# Metagenome and metabolome insights into the energy compensation and exogenous toxin degradation of gut microbiota in high-altitude rhesus macaques (*Macaca mulatta*)

**DOI:** 10.1038/s41522-023-00387-3

**Published:** 2023-04-20

**Authors:** Junsong Zhao, Yongfang Yao, Diyan Li, Wei Zhu, Hongtao Xiao, Meng Xie, Ying Xiong, Jiayun Wu, Qingyong Ni, Mingwang Zhang, Huailiang Xu

**Affiliations:** 1 College of Life Science, Sichuan Agricultural University, Ya’an, 625014 China; 2 College of Agronomy and Life Sciences, Zhaotong University, Zhaotong, 657000 China; 3 School of Pharmacy, Chengdu University, Chengdu, 610106 China; 4 College of Animal Science and Technology, Sichuan Agricultural University, Chengdu, 611130 China

**Keywords:** Microbial ecology, Metagenomics

## Abstract

There have been many reports on the genetic mechanism in rhesus macaques (RMs) for environmental adaptation to high altitudes, but the synergistic involvement of gut microbiota in this adaptation remains unclear. Here we performed fecal metagenomic and metabolomic studies on samples from high- and low-altitude populations to assess the synergistic role of gut microbiota in the adaptation of RMs to high-altitude environments. Microbiota taxonomic annotation yielded 7471 microbiota species. There were 37 bacterial species whose abundance was significantly enriched in the high-altitude populations, 16 of which were previously reported to be related to the host’s dietary digestion and energy metabolism. Further functional gene enrichment found a stronger potential for gut microbiota to synthesize energy substrate acetyl-CoA using CO_2_ and energy substrate pyruvate using oxaloacetate, as well as a stronger potential to transform acetyl-CoA to energy substrate acetate in high-altitude populations. Interestingly, there were no apparent differences between low-altitude and high-altitude populations in terms of genes enriched in the main pathways by which the microbiota consumed the three energy substrates, and none of the three energy substrates were detected in the fecal metabolites. These results strongly suggest that gut microbiota plays an important energy compensatory role that helps RMs to adapt to high-altitude environments. Further functional enrichment after metabolite source analysis indicated the abundance of metabolites related to the degradation of exogenous toxins was also significantly higher in high-altitude populations, which suggested a contributory role of gut microbiota to the degradation of exogenous toxins in wild RMs adapted to high-altitude environments.

## Introduction

The extreme cold, high ultraviolet radiation, low oxygen content, and poor food resources of high-altitude environments pose great challenges to animal survival^1,2^. The gut microbiota plays an important role in a variety of physiological activities in animals, such as cardiovascular activity, food digestion, energy metabolism, nutrient homeostasis, immune regulation, and maintenance of body temperature^3–8^. The gut microbiota has also been found to be intimately involved in host environmental adaptation^9^. Understanding the intrinsic role of the gut microbiota in the adaptation of wildlife to extreme environments at high altitudes is crucial for studying the adaptive evolution of animals.

The structural composition of the gut microbiota is the result of strong selection and co-evolution between the host and its environment, and studies in a variety of mammals (e.g., humans, ruminants, rabbits, rhesus macaques, mice, etc.) have confirmed that the gut microbiota positively contributes to the adaptation of its hosts to high-altitude environments^2,10–14^. Metabolites, such as short-chain fatty acids (SCFAs), bacteriocins, and bacterial proteins produced by gut microbiota, play important roles in host metabolic regulation, energy replenishment, and immune regulation^15–19^. Among them, the production of SCFAs is related to diet, microbiota composition, and the host^19^. The macronutrient composition of the diet determines the amount and source of fermentable substrates for the gut microbiota and is a major driver of gut microbiota structure and function^19,20^. The SCFAs produced by gut microbiota when fermenting host foods are mainly acetate, propionate, and butyrate, which mediate energy metabolism and physiological regulatory processes in the host^21,22^. There are also a few branched-chain fatty acids (BCFAs), that although in low abundance, can also mediate metabolic processes in the host^23–25^. The gut microbiota of high-altitude ruminants helps the host adapt to the high-altitude environment by reducing the emission of methane and elevating the production of volatile fatty acids^11^.

Nonhuman primates (NHPs) are the closest biological relatives of humans, originated from tropical lowlands and are primarily found in warm tropical and subtropical environments, with a few species having radiated into temperate alpine forests^26,27^. The structure and composition of gut microbiota in NHPs are closely related to factors, such as dietary composition^28,29^, habitat occupancy^30^, social interactions^30^, eco-environmental factors^31^, and health status^32,33^. The mechanisms of high-altitude adaptation in NHPs, including physiological, behavioral, and genetic, have been widely studied^34–36^. However, less is known about the role of gut microbiota in high-altitude adaptation.

The gut microbiota of NHPs responds to changes in season and differences in habitat environment^9,28^. The plasticity of the gut microbiota in Ethiopian geladas (*Theropithecus gelada*) largely provides dietary and metabolic flexibility to the host and may be a key factor in allowing them to thrive in a changing environment^37^. There have been many reports on the interaction between gut microbiota composition and environmental adaptation in nonhuman primates based on the 16S rRNA genes of the microbiota; however, studies on the interaction between the functional gene composition of gut microbiota and metabolite composition in the environmental adaptation of NHPs are largely lacking.

Rhesus macaques (RMs) are the most widely distributed small omnivorous NHPs and are currently a widely used biological model in medical and biological research given their high physiological similarity to humans^14^. In China, wild RMs are widely distributed from sea level to the Qinghai Tibetan Plateau (altitude exceeding 3000 m). Given its strong adaptability, the RM is an ideal model for investigating the adaptation mechanisms involving primate gut microbiota in the host to high-altitude environments^38^. Previous studies have also found significant differences in the composition of the gut microbiota between RMs from different habitats, with the highest diversity and the largest number of specific microbiota in high-altitude populations^9,14^. Related studies are mainly based on 16S rRNA gene sequencing and the characterization of gut microbiota composition in RMs at the genus or family level. Less is known about the functional and metabolite composition of gut microbiota, or the interactive mechanisms between gut microbiota and host adaptation to high-altitude environments. Therefore, we collected fresh fecal samples in March 2021 from four wild RM populations on the southeast edge of the Qinghai Tibetan Plateau, from Pamlin (HA population) and Xi’eluo Town (HB population) in Yajiang County at altitudes higher than 3500 m and from Baidicheng (LA population) and Simianshan (LB population) in Chongqing at altitudes lower than 1000 m. After individual identification based on microsatellite loci and gender identification based on the zinc finger protein X-linked (ZFX)/ sex-determining region of the Y-chromosome (SRY) gene, metagenomic and metabolomic data were collected from 40 individuals. Differences in taxonomic composition and functional genes of gut microbiota and differences in fecal metabolites between high-altitude populations and low-altitude populations were revealed. Metagenome and metabolome association analysis was conducted to shed new light on the synergistic mechanism of gut microbiota in the adaptation of RMs to high-altitude environments.

## Results

### Sequence statistics

We collected 40 RM fecal samples and obtained a total of 1,964,188,761 raw reads after metagenomic sequencing and 1,957,927,597 clean reads remained after quality control, accounting for 99.7% of the raw data. After elimination of the host sequence (*Macaca mulatta*), a total of 1,855,936,251 optimized reads were obtained, representing 94.49% of the raw reads. After optimizing the read assembly, 14,321,941 contigs were obtained, and 9,432,768 microbiota non-redundant gene data were obtained after gene prediction and de-redundancy.

### Differences in gut microbiota composition between RMs at high- and low-altitude

**Fig. 1:**
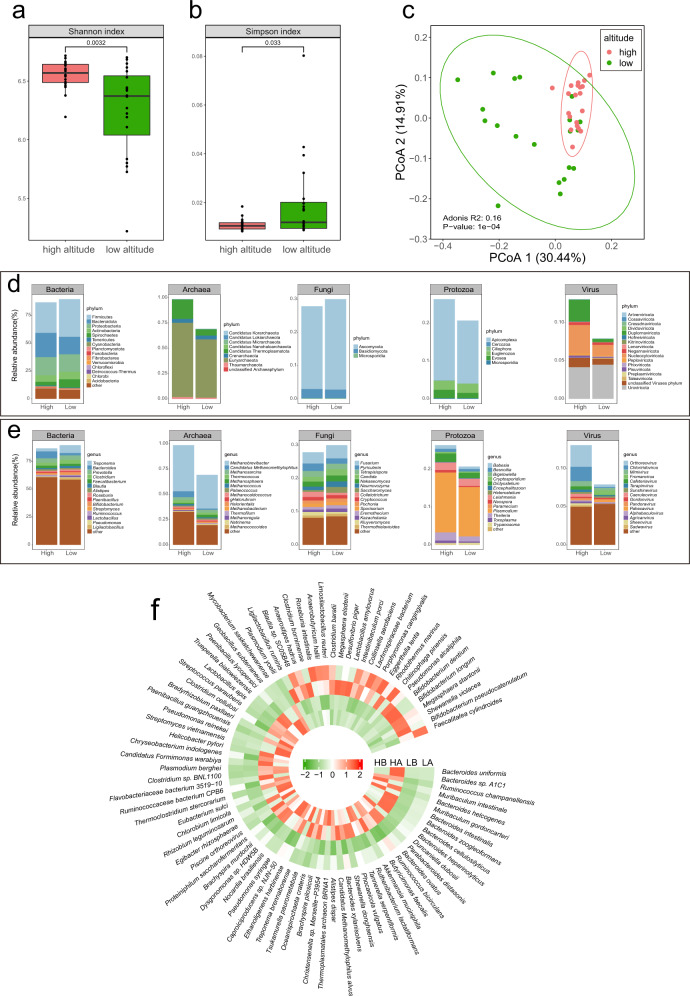
Differences in gut microbiota taxonomic composition between high-altitude populations and low-altitude populations of the wild rhesus macaque. **a**, **b** Alpha diversity differences (Shannon index, Simpson index; Welch’s *t*-test), The boxes represent 25th–75th percentiles, black lines indicate the median and whiskers extend to the maximum and minimum values within 1.5× the interquartile range, point represents the actual value of the sample. **c** Principal coordinate analysis (PCoA) of individuals in high and low altitude populations based on gut microbiota at the species level (Bray–Curtis distance; Adonis test, R^2^ = 0.16, *P* < 0.001), The ellipse borders represent the 95% confidence interval. **d** Relative abundance of gut microbiota taxa at the phylum level. **e** Relative abundance of gut microbiota taxa at the genus level. **f** Species level differential relative abundance heatmaps of the gut microbiota between high-altitude and low-altitude populations in the RMs (LEfSe analysis, LDA > 2, *P* < 0.01).

The community structure of the gut microbiota of the wild macaque populations at high altitudes differed significantly from that of the populations at low altitudes. Alpha diversity analysis of the microbiota at the species level showed that the diversity of gut microbiota of high-altitude populations was significantly higher than that of low-altitude populations (Shannon index, Simpson index, Welch’s *t*-test, *P* < 0.05; Fig. 1a, b). At the species level, principal coordinate analysis (PCoA) based on Bray–Curtis distance found that there was a significant separation between high-altitude and low-altitude populations, and the Adonis test also showed that there was a significant difference in microbiota composition between high-altitude and low-altitude populations (Adonis test, R^2^ = 0.16, *P* < 0.001; Fig. 1c). The PCoA of different genders based on the Bray–Curtis distance found that there was no significant separation between male and female individuals, and the Adonis test also showed that there was no significant difference in microbiota composition between males and females (Adonis test, R^2^ = 0.03, *P* = 0.4202, Supplementary Fig. 1). After taxonomic annotation based on the predicted nonredundant gene data by Kraken2, 7471 species were obtained (resolution at species level was 72.04 ± 2.97%), belonging to the four kingdoms. These included 6107 species of bacteria (mean ± SD, 88.14 ± 2.89%) belonging to 40 phyla, 546 families, and 1774 genera; 329 species of Archaea (0.84 ± 0.86%) belonging to 9 phyla, 56 families, and 149 genera; 808 species of viruses (0.10 ± 0.06%) belonging to 17 phyla, 101 families, and 427 genera; 79 species of fungi (0.29 ± 0.07%) belonging to 3 phyla, 27 families, and 47 genera; and 44 species of protozoa (0.23 ± 0.06%) belonging to 5 phyla, 13 families, and 18 genera. At the phylum level, bacteria were mainly dominated by Firmicutes (30.73 ± 6.38%), Bacteroidota (18.69 ± 6.31%), Proteobacteria (16.03 ± 2.54%), Actinobacteria (6.36 ± 2.22%), and Spirochaetes (5.35 ± 6.13%) (Fig. 1d). Archaea were dominated by Euryarchaeota (0.66 ± 0.85%), Candidatus Thermoplasmatota (0.13 ± 0.13%), and Crenarchaeota (0.04 ± 0.02%) (Fig. 1d). Viruses were dominated by Nucleocytoviricota (0.03 ± 0.05%) and Duplornaviricota (0.02 ± 0.02%) (Fig. 1d). Fungi were mainly dominated by Ascomycota (0.26 ± 0.06%) and Basidiomycota (0.03 ± 0.01%) (Fig. 1d). Protozoa were mainly dominated by Apicomplexa (0.19 ± 0.06%), Euglenozoa (0.02 ± 0.01%), and Evosea (0.02 ± 0.01%) (Fig. 1d). At the genus level, the bacteria were mainly dominated by *Treponema* (4.96 ± 6.18%), *Bacteroides* (4.17 ± 3.99%), *Prevotella* (3.94 ± 2.09%), *Clostridium* (3.15 ± 1.45%), *Faecalibacterium* (2.03 ± 0.87%), *Blautia* (1.48 ± 0.83%), and *Alistipes* (1.33 ± 0.43%) (Fig. 1e). Archaea were dominated by *Methanobrevibacter* (0.40 ± 0.82%), *Candidatus Methanomethylophilus* (0.04 ± 0.05%), and *Methanosarcina* (0.03 ± 0.01%) (Fig. 1e). Viruses were dominated by *Orthoreovirus* (0.02 ± 0.02%) and *Chlororidovirus* (0.01 ± 0.05%) (Fig. 1e). Fungi in eukaryotes were mainly dominated by *Fusarium* (0.04 ± 0.01%), *Pyricularia* (0.02 ± 0.01%), *Tetrapisispora* (0.02 ± 0.01%), *Candida* (0.02 ± 0.01%), and *Nakaseomyces* (0.02 ± 0.01%) (Fig. 1e). Protozoa in eukaryotes were mainly dominated by *Plasmodium* (0.14 ± 0.05%), *Dictyostelium* (0.02 ± 0.01%), *Leishmania* (0.02 ± 0.01%), and *Theileria* (0.02 ± 0.02%) (Fig. 1e). The Linear Discriminant Analysis (LDA) Effect Size (LEfSe) analysis (*P* < 0.01, LDA > 2) showed that there were significant differences in the abundance of 86 microbiota species and 82 genera between populations at high and low altitudes (Fig. 1f, Supplementary Fig. 2), including 81 bacterial species, 2 archaeal species, 2 protozoan species, and 1 viral species. Based on the further screening of gut microbiota with higher (or lower) abundance in both high-altitude populations than in both low-altitude populations revealed that 40 microbiota species were significantly more abundant in the two high-altitude populations than in the two low-altitude populations, including 37 Bacteria, 1 Archaea, and 2 Protozoa. 14 bacteria were more abundant in the low-altitude populations than in the high-altitude populations. Previously published data related to microbial function also revealed that 16 of the 37 bacteria species that were significantly more abundant in the high-altitude populations were associated with dietary digestion and energy metabolism of the host (Table 1 and Supplementary Table 1), including five that were shown to have the ability to produce acetate (*Akkermansia muciniphila*, *Candidatus Formimonas warabiya*, *Ethanoligenens harbinense*, *Ruminococcaceae bacterium CPB6*, and *Ruthenibacterium lactiformans*). Four of the 14 bacterial species with significantly higher abundance in low-altitude populations have previously been reported to be involved in dietary digestion and energy metabolism of the host (Table 1), including two that were shown to have the ability to produce butyrate (*Anaerostipes hadrus* and *Megasphaera elsdenii*).

**Table 1:** Classification of gut microbiota with significant differences (LefSe test) in abundance between high-altitude and low-altitude populations of rhesus macaques.

Classification	Bacteria					Archaea	Protozoa
	Total	DDAESM	Lm	PPODA	OOU	Total	Total
Total	51	20	2	11	18	1	2
High altitude	37	16	0	8	13	1	2
Low altitude	14	4	2	3	5	0	0

*DDAESM* Dietary digestion and energy substance metabolism refers to the associated microbiota that degrades nutrients in food and produces energy substances, *Lm* Immunomodulation refers to the microbiota associated with host immune regulation, *PPODA* Potentially pathogenic or disease associated refers to being reported as disease associated or presence of potentially pathogenic microbiota, *OOU* Other or unknown.

Meanwhile, we also analyzed the difference in gut microbiota composition between different populations with the same altitude distribution via LEfSe analysis (LDA > 3.5, *P* < 0.01). It was found that in the low-altitude environment, the abundance of *Treponema succinifaciens*, *Lactobacillus ruminis*, *Bifidobacterium adolescentis*, *Bifidobacterium angulatum*, and *Clostridium bornimense* was significantly higher in the LB population. In the high-altitude environment, the abundance of *Bacteroides A1C1* and *Bacteroides uniformis* was significantly higher in the HA population, while the abundance of *Faecalibacterium prausnitzii* was significantly higher in the HB population (Supplementary Fig. 3).

### Differences in gut microbiota functional genes between RMs at high- and low-altitude

**Fig. 2:**
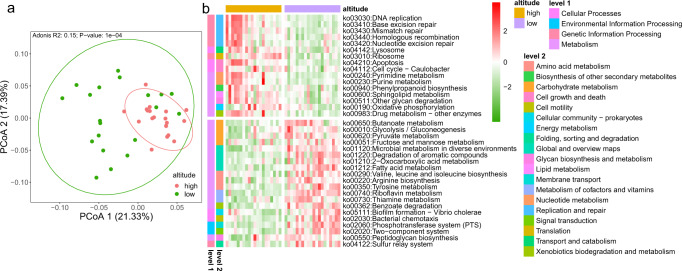
Differences in gut microbiota functional gene composition. **a** Principal coordinate analysis (PCoA) of individuals in high- and low-altitude populations based on gut microbiota gene-family abundance (Bray–Curtis distance; Adonis test, R^2^ = 0.15, *P* < 0.001), The ellipse borders represent the 95% confidence interval. **b** Heatmap showing the difference in functional enrichment rate of KEGG tertiary pathways of gut microbiota between high-altitude and low-altitude populations of RMs (LEfSe analysis, LDA > 2, *P* < 0.05).

The functionality of the gut microbiota was confirmed using metagenomics sequencing. After functional assignment based on the eggNOG database, 13,494 Kyoto Encyclopaedia of Genes and Genomes (KEGG) orthologous (KO) genes were identified. Through the PCoA based on the Bray–Curtis distance of relative abundance of the KO genes, we found that there was a separation between the high- and low-altitude populations (Adonis test, R^2^ = 0.15, *P* < 0.001; Fig. 2a). There was no significant separation of KO genes between genders (Adonis test, R^2^ = 0.02, *P* = 0.4657, Supplementary Fig. 4). KEGG functional pathway enrichment analysis found that 430 KEGG Level 3 pathways were enriched in RMs. Further, the LEfSe analysis found that there were significant differences in the enrichment of 36 KEGG Level 3 pathways between high-altitude and low-altitude populations. (LDA > 2, *P* < 0.05; Fig. 2b). Among these pathways, high-altitude populations showed significantly higher enrichment in pathways such as DNA replication (KO03030), nucleotide excision repair (KO03420), mismatch repair (KO03430), and homologous recombination (KO03440); however, low-altitude populations showed significantly higher enrichment in pathways such as tyrosine metabolism (KO00350), pyruvate metabolism (KO00620), and butanoate metabolism (KO00650).

**Fig. 3:**
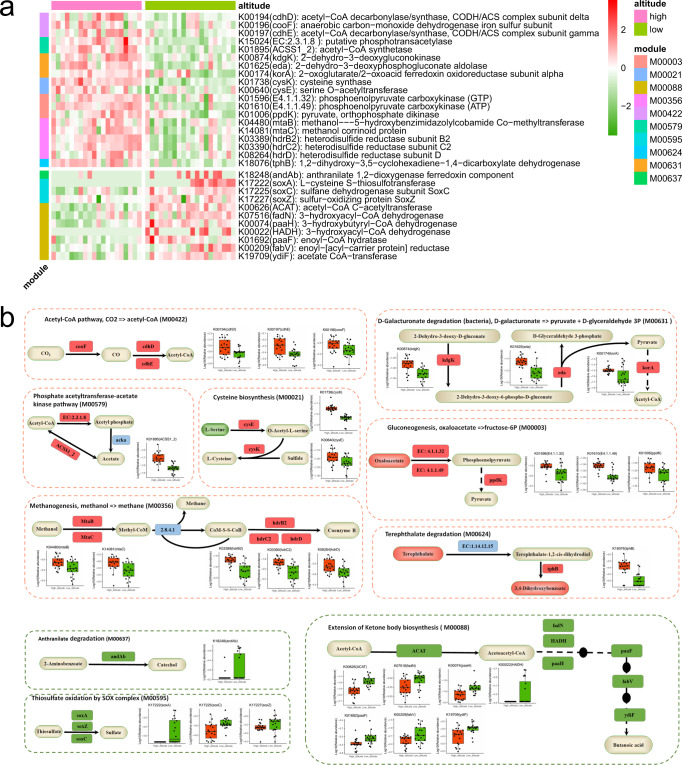
Main metabolic modules associated with the differential functional genes (T-test, *P* < 0.05) of the gut microbiota in high-altitude populations and low-altitude populations of wild RMs. **a** Heatmap of major differential genes (log10 relative abundance). **b** Schematic diagram of main modules involved in differential genes. Red indicates that relative abundance was significantly higher in high-altitude populations. Green indicates that abundance was significantly higher in low-altitude populations. The boxes represent 25th–75th percentiles, black lines indicate the median and whiskers extend to the maximum and minimum values within 1.5× the interquartile range, point represents the actual value of the sample.

Further, we identified that the abundance of 4934 KO genes differed significantly between high- and low-altitude populations through Statistical Analysis of Metagenomic Profiles (STAMP) difference analysis (Welch’s *t*-test, *P* < 0.05). Comparison of these 4934 KO genes through the KEGG Mapper – Color tool found that 320 KO genes are enriched in the “microbial metabolism in diverse environments (map01120)” pathway, 131 of which were significantly richer in high-altitude populations and 189 that were significantly richer in low-altitude populations (Supplementary Fig. 5). After screening the 320 KO genes by modules, it was found that the genes related to the CO_2_ ≥ acetyl-CoA (M00422), D-galactonate ≥ pyruvate + D-glycoraldehyde 3P (M00631), and oxaloacetate ≥ fructose-6P (M00003) modules were significantly enriched in the high-altitude population. Meanwhile, SCFA synthesis modules also differed between high-altitude and low-altitude populations, that is, synthetic acetate-related phosphate acetyltransferase-acetate kinase pathway (M00579) genes were also significantly enriched in high-altitude populations, but butyrate synthesis-related ketone body biosynthesis module (M00088) extended pathway genes were significantly enriched in low-altitude populations (Fig. 3a, b). Further, the gene enrichment of these SCFA breakdown-related modules by the microbiota themselves did not differ significantly between high- and low-altitude populations. These results suggest that the gut microbiota at high altitudes has a significantly higher potential to synthesize acetate, while that of RMs at low altitudes has a significantly higher potential to synthesize butyrate, but with no significant difference in microbiota decomposition of these substrates. In addition, we also found that high-altitude populations were significantly enriched in genes related to the cysteine biosynthesis (M00021), terephthalate degradation (M00624), and the methanol ≥ methane (M00356) modules (Fig. 3a, b). The low-altitude population was also significantly enriched in the genes related to the anthranilate degradation module (M00637) and the thiosulfate oxidation by the SOX complex module (M00595) (Fig. 3a, b).

### Differences in fecal metabolites between RMs at high- and low-altitude

In order to test whether the metabolites produced by the actual metabolic profile and related pathway effects of the gut microbiota of RMs are excreted with feces, we performed metabolite profiling of all RM fecal samples using an untargeted metabolomics approach via UPLC-MS/MS. Peak map reads were performed on the chromatographic peaks detected in the samples, where the peak area of each characteristic peak represents the relative quantification value of one metabolite. The quantification results were normalized using the total peak area, and a total of 2338 different metabolites were obtained from the alignment, of which 1550 were obtained from positive ion mode and 788 from negative ion mode (Table 2 and Supplementary Table 2). After comparing the metabolites with the Human Metabolome Database (HMDB), 878 metabolites of 158 SubClasses were annotated (Table 2 and Supplementary Table 2). These were dominated by fatty amides (percentage of the total annotated amount; mean ± SD: 26.27 ± 6.64%), fatty acids and conjugates (20.67 ± 7.68%), bile acid alcohols, alcohols, and derivatives (8.29 ± 6.54%), bilirubins (4.70 ± 2.98%), amino acids, peptides, and analogs (3.91 ± 2.51%), phenylpropane (3.29 ± 0.96%), fatty acid esters (2.80 ± 1.90%), and purines and purine derivatives (2.15 ± 3.97%) (average relative abundance >2%; Supplementary Fig. 6). Although the total abundance of the fatty acid and conjugate class metabolites did not differ significantly between the high- and low-altitude populations according to the metabolomic data, we found differences in the potential of gut microbiota to synthesize SCFAs between the high- and low-altitude populations according to the metagenomic data, with a significantly higher potential for acetate synthesis in the high-altitude populations and a significantly higher potential for butyrate synthesis in the low-altitude populations. In order to determine whether the SCFAs produced by gut microbiota are excreted with feces, we conducted further screening of the fatty acid and conjugate class metabolites and found 71 fatty acid and conjugate class metabolites, 28 of which (including elaidic acid, suberic acid, sebacic acid, 20-carboxyleukotriene B4, and erucic acid) differed significantly in abundance (Fold Change (FC) > 1.5 or FC < 0.7, Variable Importance in the Projection (VIP) > 1, *P* < 0.05) between high- and low-altitude populations. However, the abundance of seven SCFAs and conjugates with less than six carbon atoms (2-hydroxy-2-methylbutanoic acid, 3-hydroxyvaleric acid, valeric acid, citraconic acid, 2-hydroxyvaleric acid, tiglic acid, and methylsuccinic acid) did notdiffer significantly (VIP < 1 or *P* > 0.05) between high- and low-altitude populations (Supplementary Table 3). These results indicate that there were no significant differences in fecal metabolic SCFAs and conjugate abundance between the high- and low-altitude populations.

**Table 2:** Summary of metabolite identification and classification annotation results.

Classification	Number of Chromatographic peaks	Number of HMDB database annotations	Number of KEGG database annotations
Positive ion mode	1550	556	392
Negative ion mode	788	322	239
Total	2338	878	631

**Fig. 4:**
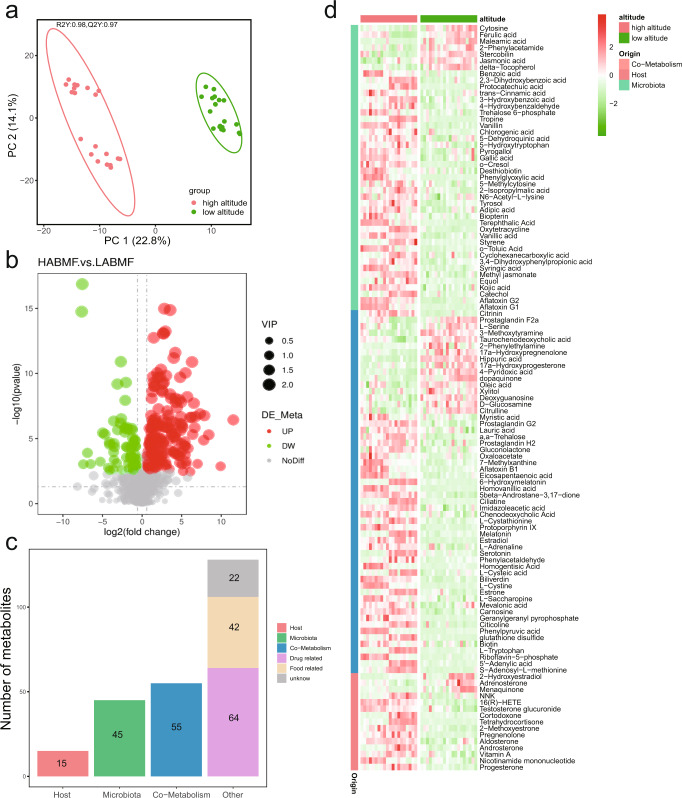
Abundance differences and source profiles of metabolites annotated by KEGG database in feces of RMs at high versus low altitude. **a** PCA analysis showed the pattern of separation between the two groups after PLS-DA analysis 200 permutation test model validation, The ellipse borders represent the 95% confidence interval. **b** Volcano map of differential metabolites between the two groups, red indicates that the relative abundance was significantly higher at high altitudes, and green indicates that the relative abundance was significantly higher at low altitudes. **c** Differential metabolite source profiles. **d** Heatmap of differential metabolite abundance from host, microbiota and co-metabolism.

After aligning these metabolites with the KEGG database to obtain 631 KEGG annotated metabolites (Table 2 and Supplementary Table 2), 243 metabolites with significant differences in abundance between high and low altitudes were identified based on multivariate analysis (FC > 1.5 or FC < 0.7, VIP > 1, *P* < 0.05). Partial least squares discriminant analysis (PLS-DA) models showed the metabolite differences between the high- and low-altitude populations according to the metabolic differences, and permutation tests indicated that the PLS-DA model did not overfit (Fig. 4a and Supplementary Fig. 7). Of the 243 metabolites with significant differences in abundancese, 180 metabolites were significantly more abundant in the high-altitude populations than in the low-altitude populations, and 63 metabolites were significantly more abundant in the low-altitude populations than in the high-altitude populations (Fig. 4b). To verify once more whether the metabolites involved in the main modules we screened by metagenome analysis were excreted with the feces, we aligned these 243 differential metabolites with the microbiota differential genes screened by metagenome analysis at the same time using the KEGG Mapper – Color tool and found that oxaloacetate in the oxaloacetate ≥ fructose-6p (M00003) module and terephthalate and 3,4-dihydroxybenzoate in the terephthalate degradation (M00624) module were significantly more abundant in the feces of populations living at high altitude. L-serine in the cysteine biosynthesis (M00021) module was significantly more abundant in feces of low-altitude populations (Fig. 3b); however, the abundance of related metabolites (acetyl-CoA, acetate, butyrate, and pyruvate) involved in the other modules did not differ significantly in the feces of populations from high and low altitudes, implying that these metabolites may be absorbed and utilized by the host to provide energy compensation.

### Differences in metabolic function based on metabolite sources

**Fig. 5:**
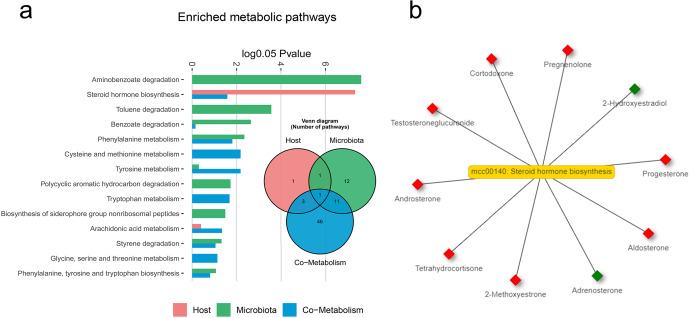
Comparison of KEGG pathways corresponding to differential metabolites of different origins. **a** Statistically significant comparisons of metabolic pathways (Hypergeometric test, log0.05 *P* value > 1); **b** Network plots obtained based on the Metorigin platform showed statistically significant steroid hormone biosynthesis (ko00140) related metabolites in the host’s own metabolism. Boxes indicate metabolites, red indicates significantly higher abundance in high-altitude populations than in low-altitude populations, and green indicates significantly higher abundance in low-altitude populations than in high-altitude populations.

**Fig. 6:**
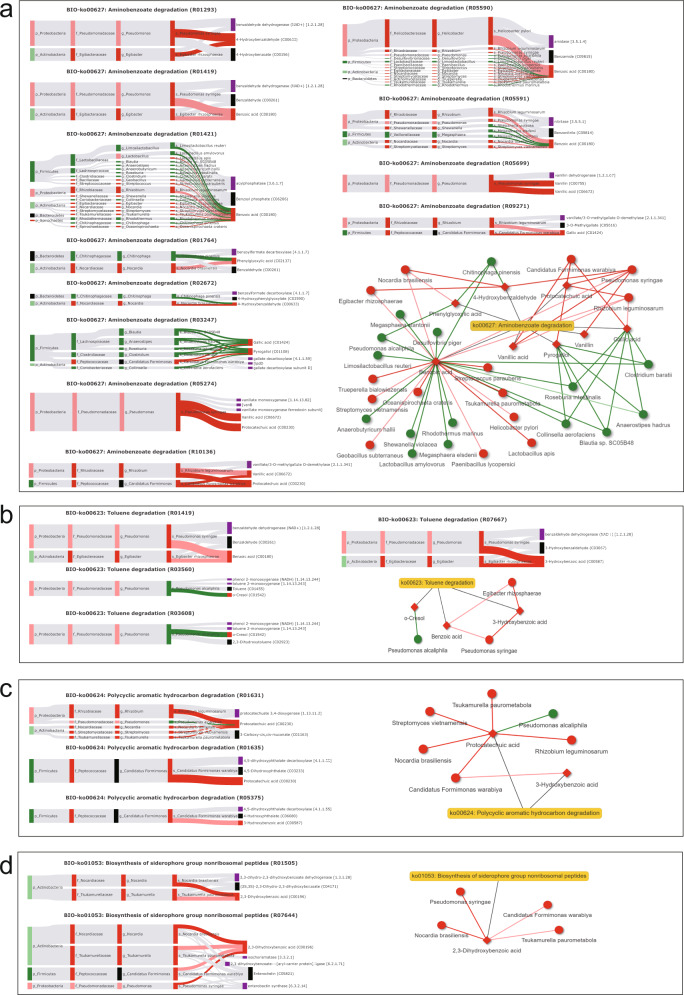
Microbiota specific metabolic pathways (Hypergeometric test, log0.05 *p*-value > 1) BIO-Sankey network diagram and network summary analysis revealed close associations between microbiota and metabolites. **a** BIO-ko00627: Aminobenzoate degradation; **b** BIO-ko00623: Toluene degradation; **c** BIO-ko00624: Polycyclic aromatic hydrocarbon degradation; **d** BIO-ko01053: Biosynthesis of siderophore group nonribosomal peptides. In the BIO-Sankey network diagram, Dark red (or green) bars indicate the microbes or metabolites that are significantly higher (or lower) in the high-altitude population (FC > 1 or FC < 1, *P* < 0.05); Light red (or green) bars indicate the microbes or metabolites that are higher (or lower) in the high-altitude population (FC > 1 or FC < 1, *P* ≥ 0.05); Black bars indicate the microbes or metabolites in the reference database; Purple bars indicate the metabolic enzymes; Dark red (or green) bands indicate significant positive (or negative) correlations (Spearman correlation test; R > or R < 0, *P* < 0.05); Light red (or green) bands indicate positive (or negative) correlations without statistical significance (Spearman correlation test; R > or R < 0, *P* ≥ 0.05); Light gray bands indicate reference relationships searched through database. In the network summary analysis diagram, the diamond and dot shapes indicate the related metabolites and microorganisms, respectively. Red (or green) nodes indicate populations at significantly higher (or lower) altitude. Connecting lines in red (or green) indicate positive (or negative) correlations between microbes and metabolites. The FC refers to the fold change, which is the ratio of the mean of all biological repeated quantitative values of each metabolite in the comparison group; *P*-value is calculated by T-test and represents the level of difference significance. Relevant analysis is completed based on MetOrigin platform.

Further searching of these 243 differential metabolites against the MetOrigin platform database for metabolite traceability revealed that the metabolites fell into 4 groups: 15 metabolites from the RMs host, 45 metabolites from gut microbiota, 55 co-metabolites from microbiota hosts, and 128 metabolites from other sources (Fig. 4c, d). The origin-based metabolic pathway enrichment analysis identified 6, 25, and 61 metabolic pathways in the host, microbiota, and shared by both respectively, 14 of which were statistically significant (Hypergeometric test, log0.05 *p* > 1; Fig. 5a). Among these, steroid hormone biosynthesis (KO00140) in the host metabolism was statistically significant, and of the 10 related metabolites, 8 were significantly more abundant in the high-altitude group than in the low-altitude group and 2 were significantly more abundant in the low-altitude group than in the high-altitude group (Fig. 5b). These results indicate that high-altitude populations have significantly higher rates of steroid hormone metabolism than low altitude populations. The MetOrigin platform was also used to carry out association analysis in combination with differential metabolites and differential microbiotas. After integrating the correlation between biology and statistics, the results showed that the specific metabolic pathways of the microbiota, including aminobenzoic acid degradation (KO00627), toluene degradation (KO00623), iron carrier non-ribosomal peptide biosynthesis (KO01053), and polycyclic aromatic hydrocarbon (PAH) degradation (KO00624) were significant. The abundance of 14 metabolites corresponding to these four pathways was significantly higher in feces of the high-altitude population, indicating that their metabolic rates were significantly higher in the high-altitude population. Subsequently, after verification by biological and statistical correlation analysis, the Biology-Sankey (BIO-Sankey) network diagram showed the correlation between microbiota and metabolites at different microbiota classification levels (Fig. 6a–d). After integration of the network diagram, it was found that 29 microbiota species were closely associated with 12 reactions of 8 metabolites in the aminobenzoic acid degradation (KO00627) pathway, of which 13 microbiota species were positively related to the 8 metabolites (Fig. 6a). Three microbiota species were closely associated with 4 reactions of 3 metabolites in the toluene degradation (KO00623) pathway, and 2 microbiotas species were positively related to 2 metabolites (Fig. 6b). 6 microbiota species are closely associated with 3 reactions of 2 metabolites in the pathway of PAH degradation (KO00624), and 5 microbiota species were positively associated with Which two metabolites (Fig. 6c). Four microbiota species were closely associated with 2 reactions of 1 metabolite in the siderophore non-ribosomal peptide biosynthesis pathway (KO01053), and 4 microbiota species were positively associated with 1 metabolite (Fig. 6d). The potential statistical relevance of the macaque gut microbiota and metabolites in these pathways was further revealed by Statistics- Sankey (STA-Sankey) network plots (Supplementary Fig. 8a–d). Notably, we found that the R01633 and R05148 responsive genes in the terephthalate degradation (M00624) module of the PAH degradation (KO00624) pathway were significantly enriched by metagenomic functional module enrichment, but based on biological database alignment, we found that only the Proteobacteria was biologically relevant to these two responses. Statistical analysis identified potential associations of multiple microbiota with the occurrence of this module, notably Proteobacteria (Supplementary Fig. 9). Furthermore, 5 of the 8 statistically significant pathways involved in microbe-host co-metabolism were associated with amino acid metabolism. After verification based on biological and statistical correlation analysis the network plot showed that a total of 17 metabolites were closely related to the 5 amino acid metabolism-related pathways, of which the abundance of 13 metabolites was significantly higher in the high-altitude populations than in the low-altitude populations (Supplementary Fig. 10), perhaps implying that high-altitude populations have higher host metabolic rates than low-altitude populations and revealing many close links between the gut microbiota and metabolites.

## Discussion

Animals living in high-altitude environments on the Qinghai Tibetan Plateau are subjected to low-pressure hypoxia, low temperature, poor food resources, low vegetation coverage, and prolonged wilting of grasses, which limit the availability of food for wildlife living in the area^1,2,39^. It has been found that multiple mammals living in high-altitude areas have adapted to the extreme environment of cold and low oxygen with a higher metabolic rate and consume more oxygen and energy to maintain normal survival compared with low-altitude animals^40,41^. Steroid hormones are important mediators of host metabolism and exert vital regulatory effects on immune regulation and reproduction^42,43^. In this study, we found that the wild RM population at high altitude had a significantly higher rate of steroid hormone biosynthesis, also implying that the high-altitude population had a higher rate of spontaneous metabolism than the low-altitude population. Furthermore, our analysis of differential metabolites associated with host-microbe co-metabolism revealed that 13 metabolites associated with cysteine and methionine metabolism (KO00270), tyrosine metabolism (KO00350), phenylalanine metabolism (KO00360), tryptophan metabolism (KO00380), and glycine, serine, and threonine metabolism (KO00260) were significantly more abundant in the high-altitude populations. The metagenomic functional analysis did not reveal any significant differences in the gene enrichment rates in these amino acid metabolic pathways between the gut microbiota of high- and low-altitude macaque populations. This suggests that high-altitude populations metabolize cysteine, methionine, tyrosine, phenylalanine, tryptophan, glycine, serine, and threonine at higher rates than low-altitude populations, which may also imply that high-altitude RM populations have a higher metabolic rate. In addition, we found that the enrichment rate of genes related to the DNA replication, base excision repair, nucleotide excision repair, mismatch repair, and homologous recombination pathways was significantly higher in high-altitude populations. This is consistent with previous studies on Tibetan chickens^44^, blind mole rats (*Spalax carmeli*)^45^, Tibetan antelope (*Pantholops hodgsonii*), and other animals living in high-altitude areas^1^, which have enhanced DNA repair capacity.

High microbiota diversity can promote the stability of the gut microbiota ecosystem and increase the rate of dietary fermentation in the host^8,13,46^. In this study, we characterized the composition of the gut microbiota of wild RMs through metagenomics, and the results showed that the diversity of the gut microbiota was significantly higher in high-altitude populations than in low-altitude populations, which was consistent with the results of 16S rRNA gene-based studies^9,14^. In particular, the differential analysis identified 16 bacteria associated with dietary digestion and metabolism of energy metabolism that were significantly more abundant in high-altitude populations than in low-altitude populations (including *Akkermansia muciniphila*, *Candidatus Formimonas warabiya*, *Clostridium cellulosi*, *Clostridium sp. BNL1100*, *Ethanoligenens harbinense*, *Proteiniphilum saccharofermentans, Ruminococcaceae bacterium CPB6*, *Ruminococcus bicirculans*, *Ruminococcus champanellensis*, and *Thermoclostridium stercorarium*), and 4 bacteria associated with dietary digestion and metabolism of energy substrates that were significantly more abundant in low-altitude populations than in high-altitude populations (*Anaerostipes hadrus*, *Clostridium bornimense*, *Megasphaera elsdenii*, and *Collinsella aerofaciens*). Previous studies have found that *A. muciniphila* is an important acetate producer that synthesizes acetate via acetyl-CoA^47^. *C. formimonas warabiya* can synthesize acetate via degradation of dichloromethane^48^, and *E. harbinense*, *R. lactatiformans*, and *R. bacterium CPB6* can synthesize acetate via the glycolytic pathway^49–51^. However, *A. hadrus* and *M. elsdenii* were confirmed to have the capacity to produce acetate^52,53^. The results on microbial composition seem to illustrate that the potential of microbiota to metabolize acetate was higher in the high-altitude population RM, whereas the potential to ferment butyrate was higher in the low altitude population. Meanwhile, previous studies have also found that *C. cellulosi*, *C. sp. BNL1100*, *R. bicirculans*, *R. champanellensis*, and *T. stereolarium* have the function of degrading cellulose and lignocellulose^54–58^, and their abundance was significantly higher in high altitude populations. *C. bornimense and A. hadrus* have the ability to degrade glycans^52,59^, and their abundance was significantly higher in low altitude populations. These results also seem to indicate that the gut microbial composition is compatible with the food availability of wild RMs. During long-term monitoring and sample collection of the HB population^60^, it was observed that the high-altitude populations consumed more food with a higher cellulose content (e.g., plant leaves) and less food with a higher sugar content (e.g., plant fruits) than low-altitude populations. When food resources are scarce in winter, RMs in high-altitude areas will eat more bark with a high cellulose content. The high abundance of microbiota associated with cellulose degradation aids macaque hosts in adapting to high-altitude environments by enhancing the degradation of high-fiber foods.

Fermentation by the gut microbiota produces SCFAs dominated by acetate, propionate, and butyrate^16^, which can be rapidly absorbed by the host gut epithelium to provide the host with energy, helping the host improve the metabolic capacity of food^19–24^. The pyruvate, propionate, butyrate, starch, sucrose, and pentose phosphate pathways are important for the fermentation of unabsorbed carbohydrates into SCFAs^25^. Meanwhile, butyrate is preferentially used as an energy source for the intestinal mucosa, propionate contributes to gluconeogenesis in the liver, and acetate has the highest concentration in blood^61^. Acetate production pathways are widely distributed in anaerobic bacteria^15^, while the production pathways of propionate, butyrate, and lactic acid are more conservative and substrate-specific^62^. Among the SCFAs produced by gut microbiota, butyrate is the most preferred source of energy in this respect, but its synthesis depends mainly on carbohydrates in the intestine^22,63^. In human gut microbiota, a variety of fermentation strategies have been developed to further generate energy. Pyruvate can be decomposed and metabolized into succinate, lactic acid, or acetyl-CoA. These intermediates can be further metabolized by hosts to produce acetate, propionate, and butyrate at the same time^23,64^. In this study, we found that high-altitude populations, acting through the m00422, m00631, and m00003 modules, can produce the energy substrates acetyl-CoA and pyruvate, mediate the energy metabolism of the host and provide energy compensation to the host. Meanwhile, the gut microbiota of RMs had a significantly higher potential to synthesize acetate at high altitudes, and a significantly higher potential to synthesize butyrate at low altitudes. However, the gene enrichment of the microbiota itself for these SCFA breakdown-related modules did not differ significantly between high- and low-altitude populations. Further, metabolite analysis did not detect these energy substrates, and the detected SCFAs did not differ significantly between the high- and low-altitude populations, indicating that these SCFAs and energy substrates are more likely to be absorbed and utilized by the host. These results are compatible with food acquisition and environmental stress in populations of wild RMs from different altitudes. Low-altitude populations are more likely to obtain foods with a high carbohydrate content and have more residual carbohydrates in their intestines than high-altitude populations; therefore, the potential to catalyze the formation of butyrate is higher as a result of the richer carbohydrate in the food of low-altitude populations. The gut microbiota at high altitudes shows high potential for acetate production. Meanwhile, pyruvate and acetyl-CoA are produced through the gut microbiota own metabolic pathways, which provide energy substrates for the host’s tricarboxylic acid cycle and fatty acid metabolic pathways. This also corresponds to the fact that the abundance of the microbiota associated with acetate production was significantly higher in the high-altitude populations. A recent study in other mammals also found that elevated acetate levels induced by altered microbiota drive hosts to seek food and energy accumulation by activating the parasympathetic nervous system to promote the secretion of insulin and gastric hunger hormones^65^. Another study in obese mice also found that oral butyrate reduced body weight largely due to increased energy expenditure and lipid oxidation^66^. This also appears to account for the higher potential for gut microbiota in high-altitude populations to directly provide energy compensation to the host through acetate synthesis and by stimulating food intake, further promoting energy accumulation. Moreover, the gut microbiota of high-altitude populations had a significantly higher abundance of genes associated with the metabolism of methanol than that of low-altitude populations. Methanol, as a by-product of microbiota metabolism, can serve as a substrate for microbiota methanogenesis and acetate^64,67^. Our results shows that the potential of microbiota in high-altitude populations to use methanol decomposition to produce energy is also higher than that in low-altitude populations, and their utilization of intestinal metabolites is more refined than that in low-altitude populations.

Wildlife gut microbiota is commonly exposed to environmental contaminants and is involved in the process of degrading environmental contaminants^68^. The gastrointestinal tract is the main route for xenobiotics to enter the body, and the gut microbiota has a high metabolic potential for xenobiotics. Gut microbiota can directly metabolize xenobiotic compounds of exogenous origin or affect the absorption, distribution, metabolism, and elimination of xenobiotics in the host, thus changing the toxicity of xenobiotics to the host^68–70^. PAHs are a broad class of organic pollutants present in the environment and toxic to humans and animals^71^. Soils act as a reservoir of PAHs mainly derived from atmospheric deposition and plant to microbiota synthesis^72,73^. Toluene is a common volatile organic compound (VOC) that is toxic to animals. Dead plant litter produces toluene, which is then transported to the soil^74–76^. In this study, we performed functional enrichment analysis of differential metabolites derived from microbiota production and found that among 4 statistically significant metabolic pathways, metabolites associated with cyclic aromatic hydrocarbon degradation and toluene degradation were significantly enriched in high-altitude populations compared with low-altitude populations. Based on biological and statistical analysis, we found that multiple responses of various bacterial communities (such as *Streptomyces vietnamensis*, *Tsukamurella paulometabola*, *Nocardia brasiliensis*, *Candidatus Formimonas warabiya*, and *Rhizobium leguminosarum*) in these two pathways play an important role,, in promoting the degradation of these exogenous toxins through R01631, R01635, and R05375 reactions in high-altitude populations. Meanwhile, our metagenomic screening based on KO genes in microbiota also revealed significantly higher *tphB* (K18076) gene richness in the polycyclic aromatic hydrocarbon degradation pathway (terephthalate degradation M00624) module that prompted the R01633 reaction to proceed. These results indicate that the degradation of PAHs, occurs at a significantly higher rate in high-altitude populations than in low-altitude populations. This means that high-altitude RMs can eat many plant roots and stems in cold seasons when there is a lack of food. As we observed during the long-term monitoring of the HB population^60^ and sample collection, high-altitude populations will dig and eat many plant roots when food resources are scarce in winter, and PAHs and toluene are widely enriched in soil and plant roots. The degradation of these exogenous toxicants by gut microbiota in high-altitude populations is important. In addition, our gut microbe-based functional enrichment analysis also revealed that high-altitude populations were significantly enriched in genes involved in the conversion of L-serine to L-cysteine in the cysteine biosynthesis (M00021) module, while related studies found that L-cysteine, an amino acid detoxification drug, is involved in cellular reduction processes and phospholipid metabolism in the liver, protects hepatocytes from damage, and promotes liver function recovery pharmacological effects^77^, implying an important role for their gut microbiota in the detoxification of the host’s own products via metabolites. These results suggest that as part of RMs adaptation to high-altitude environments, the gut microbiota contributes to hosts degradation of exogenous toxicants.

**Fig. 7:**
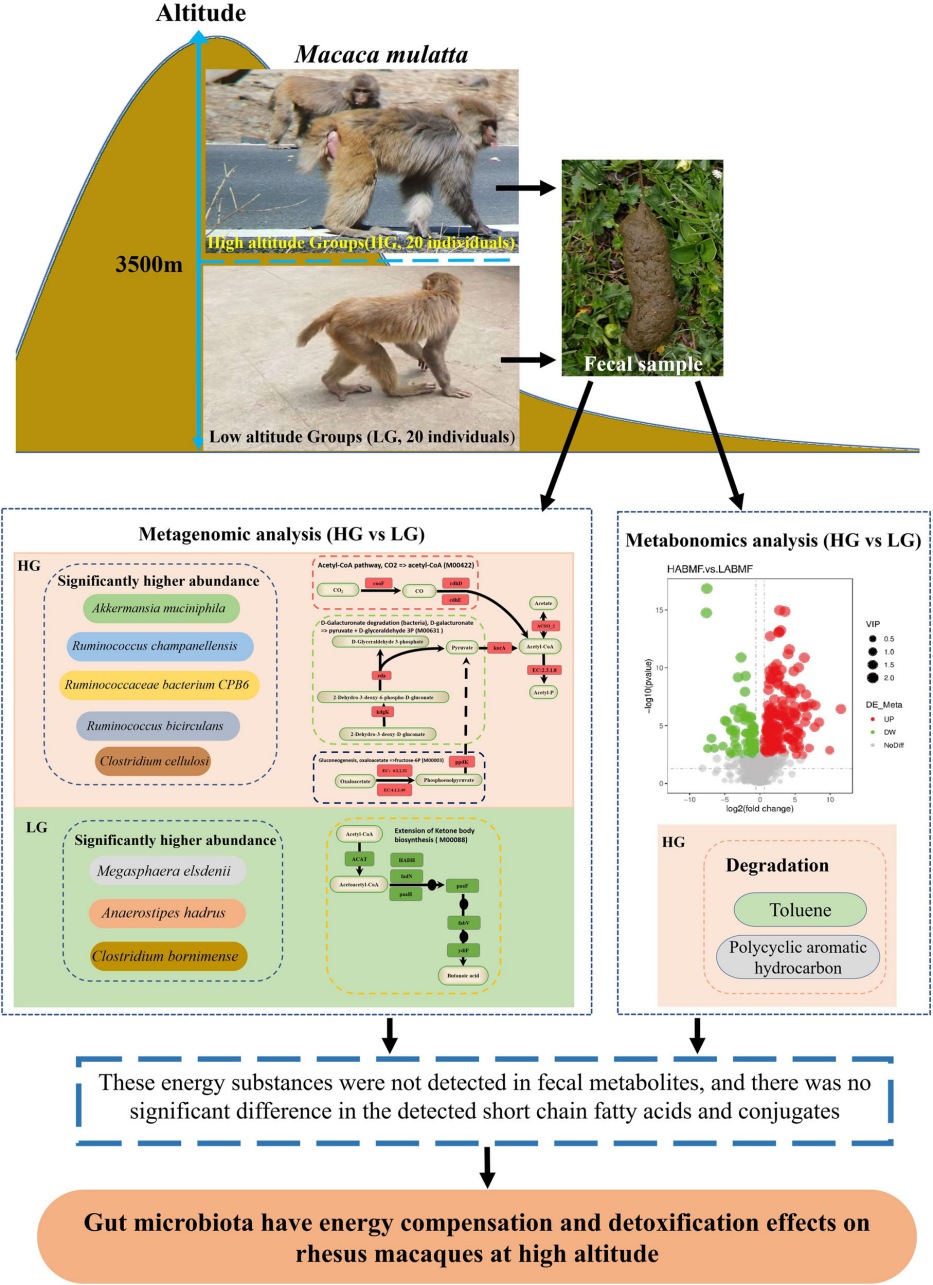
Study design and main results. cooF anaerobic carbon-monoxide dehydrogenase iron sulfur subunit, cdhD acetyl-CoA decarbonylase/synthase, CODH/ACS complex subunit delta, cdhE acetyl-CoA decarbonylase/synthase, eda 2-dehydro-3-deoxyphosphogluconate aldolase, kdgK 2-dehydro-3-deoxygluconokinase, E4.1.1.32 phosphoenolpyruvate carboxykinase, E4.1.1.49 phosphoenolpyruvate carboxykinase, ppdK pyruvate, orthophosphate dikinase, korA 2-oxoglutarate/2-oxoacid ferredoxin oxidoreductase subunit alpha, ACSS1_2 acetyl-CoA synthetase, EC:2.3.1.8 putative phosphotransacetylase. Red indicates that relative abundance was significantly higher in high-altitude populations. Green indicates that abundance was significantly higher in low-altitude populations. These macaque photos were taken by ourselves. These images and every element of these images were created by us.

In conclusion, this study revealed the synergistic involvement of the gut microbiota in the adaptation of wild RMs to high-altitude environments (Fig. 7). Wild RMs adapted to high energy demands and high-quality food deprivation in a high-altitude environment had a diverse gut microbiota. And the abundance of multiple species of bacteria associated with acetate synthesis and those associated with fiber degradation was significantly higher than in low-altitude populations. We found a stronger potential for gut microbiota to synthesize the energy substrate acetyl-CoA using CO_2_ and the energy substrate pyruvate using oxaloacetate, as well as a stronger potential to transform acetyl-CoA to the energy substrate acetate in high-altitude populations. These energy substrates provide the host with enhanced energy compensation at high altitudes. Acetate production by gut microbiota promotes host insulin and gastric ghrelin secretion, driving food behaviors seeking and further energy accumulation. Meanwhile, in high-altitude populations, the gut microbiota can effectively help the host degrade PAHs consumed when feeding on complex foods such as plant rhizomes. In addition, we revealed biological and statistical links between multiple distinct gut microbiota and metabolites, providing fundamental data for understanding the ways gut microbiota affect the host.

## Methods

### Fecal sample collection

**Fig. 8:**
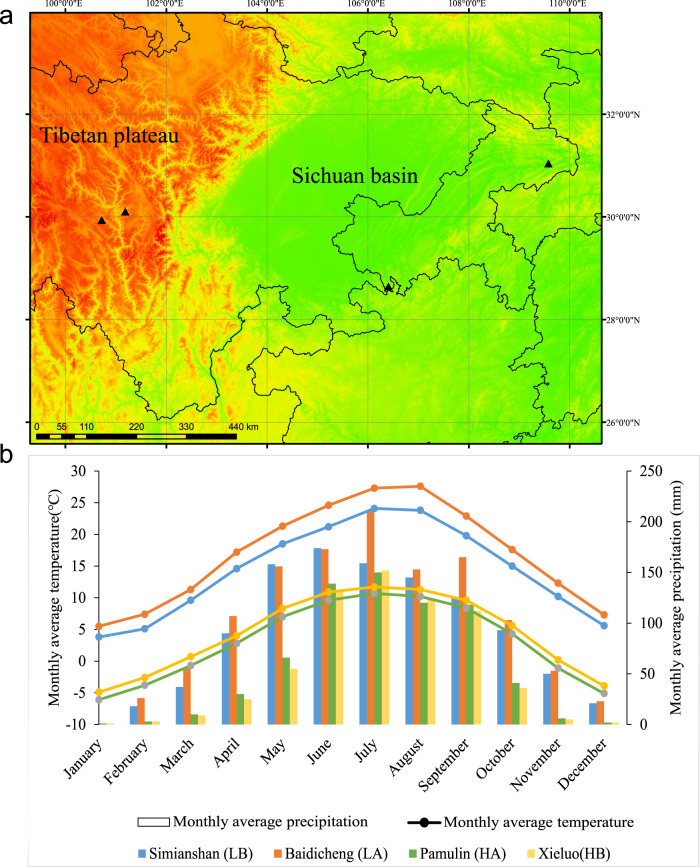
Distribution of sampling sites and climate overview. **a** Distribution of sample collection sites. **b** Monthly average temperature and precipitation data for 1970–2000 at the sampling site obtained based on WorldClim (version 2.1). The boundary data comes from the data published by China National Basic Geographic Information Center. Triangles represent sample points.

Fecal samples from RMs were collected from the wild natural habitat in March 2021. The samples were collected from Pamulin (HA; 101.182061°E, 30.101192°N) and Xi’elou Town (HB; 100.715649°E, 29.936961°N) in Yajiang County, Ganzi Prefecture, Sichuan Province at an altitude of more than 3000 m and from Baidicheng (LA; 109.577491°E, 31.040904°N) and Simianshan (LB; 106.405604°E, 28.644834°N) of the Chongqing municipality at an altitude of less than 1000 m (Fig. 8a). Yajiang County is located on the southeast edge of the Qinghai Tibet Plateau, with high-altitude climate characteristics of low temperature, low oxygen, and high ultraviolet radiation. The vegetation in the RMs habitat is mainly alpine coniferous forest, alpine hardwood forest, and alpine meadow, so it is difficult for RMs to obtain food. Simianshan and Baidicheng in Chongqing are located on the southeast edge of the Sichuan Basin, a subtropical humid monsoon climate area with a mild climate and high oxygen content. The vegetation in this habitat is mainly broad-leaved evergreens and broad-leaved deciduous trees, and it is less difficult for RMs to obtain food. Comparison of the monthly average temperature and precipitation data for the 4 sampled sites based on ArcGIS 10.0 and WorldClim’s (version 2.1)^78^ published monthly climate data for 1970–2000 (resolution 30 s) revealed that the climate of the habitats of the two high-altitude populations (Yajiang Pamulin Temple and Xi’elou Town) was similar, with the lowest average temperature in January (−6.1 °C/−4.9 °C) and the highest average in July (−10.7 °C/−11.7 °C), and precipitation mainly falling in 6–9 months, with the highest monthly precipitation of 150 mm/152 mm showing distinct high-altitude climatic features. Although separated by a wide geographic distance, the habitats of the two low-altitude populations (Baidicheng and Simianshan, Chongqing) also have similar climates, with the lowest average temperature in January (5.5 °C/3.8 °C), the highest average temperature in July/August (27.6 °C/21.2 °C), the highest monthly precipitation of 212 mm/174 mm, and significantly higher temperature and humidity than the two high-altitude population habitats (Fig. 8b). According to the observations made during sampling, the total number of RM groups sampled at the four sites ranged from 40 to 50, and all sampled individuals were considered adult individuals based on follow-up observations and comparison of stool size. After defecation by the wild RMs, fecal samples were immediately collected with sterile gloves then stored in liquid nitrogen and transported to the laboratory. The samples for DNA preparation and metabolome detection for metagenomic sequencing were taken from the center of the fecal samples under sterile conditions, while samples for DNA preparation for individual identification and gender identity were taken from the surface. After collection, the samples were transported in liquid nitrogen and stored at -80 °C until analysis. There was no direct contact with RM during fecal collection, and our study had no impact on the health or welfare of RMs.

Samples collected from the four locations were analyzed for microsatellite molecular markers for individual identification and for SRY/ZFX genes for sex determination. The partial SRY gene (364 bp) on the Y chromosome and the partial ZFX gene (183 bp) on the X chromosome were used for sex determination. The primers were designed by Primer Premier 5 based on sequences in the GenBank database (accession numbers AF284311 and XM_028842412). The six microsatellite loci used for individual identification were amplified using the primers designed in our previous study for nested PCR. The primer pairs (Supplementary Table 4) under their respective annealing temperatures were used with 1.0 μl of template DNA or the product of the first round of PCR in a 20 μl PCR mixture (95 °C for 10 min initial denaturation; 35 cycles of denaturation at 94 °C for 30 s, 45 s annealing, and 30 s extension at 72 °C; followed by final extension for 10 min at 72 °C). All PCR products were examined by electrophoresis in 1.5% agarose gels containing ethidium bromide. Capillary electrophoresis analysis was used for PCR products of microsatellite loci. Sex determination was performed with five independent experiments per sample. Ten individual samples were screened per population by a male to female ratio, and a total of 40 fresh fecal samples were subjected to subsequent metagenomic and metabolomic testing (Fig. 7, Supplementary Table 5).

### Metagenomic sequencing and quality control of raw data

DNA samples with a total content >25 ng, dispersion concentration above 500 bp, and no serious agglomeration below 500 bp were used for library preparation. The qPCR concentration of the final library was greater than 1.5 nm. There was no splice sequence, heteropeaks, or small fragments, and the library had a fragment size of 350 bp. After we completed Dnbseq-T7 platform (MGI Tech Co., Ltd. China) sequencing (Novogene Co., Ltd. China), adapters and low-quality reads in raw data were removed by Trimmomatic^79^, and potential RM sequences were removed by Bowtie2^80^ based on the RM reference genome (assembly Mmul_10; GCA_003339765.3).

### Assembly, taxonomic annotation, functional prediction, and quantification of genes

After quality control of the data obtained and the removal of host genes, mate pair assembly was performed using MEGAHIT (1.2.9)^81^, and the quality of the assembly results was assessed using QUAST (5.0.2)^82^. Gene prediction was done using Prodigal (2.6.3)^83^ with the parameter ‘-p meta’. The gene model of all genes predicted by CD-hit (4.8.1)^84^ was used to construct a non-redundant gene catalog parameter of ‘- as 0.9 - c 0.95 - G 0 - g 0 - T 0 - M 0’. SALMON (1.3.0)^85^ was used to quantify the non-redundant genes. Functional assignments of the protein sequences was done based on DIAMOND alignment against the eggNOG 5.0 database using eggNOG Mapper (2.0.0)^86^ by taking the best hit with the criterion of an E value <1e−3. The functional description of the portal gene responding to the searched sequence was the final annotation result to calculate the difference in gene composition; the next difference analysis was performed using normalized counts (TPM values). STAMP (2.1.3)^87^ was used to analyze the differences in gene composition between the low-altitude and high-altitude populations. After false discovery rate (FDR) corrections, *P* < 0.05 was used as the threshold for significance, revealing the differential genes of the gut microbiota between high- and low-altitude populations of RMs. The 8 GB MiniKraken database, pre-built using the abundance estimates of Kraken2 (2.1.1)^88^, was used to test the classification analysis based on non-redundant genes. LEfSe analysis was completed using LEfSe (1.1.2). Taxonomic composition, alpha diversity, beta diversity analysis, and visualization of all results were done using R (4.1.2). In order to investigate pathway modules known in microorganisms, we collected KEGG modules listed under “Microbial metabolism in diverse environments” (map01120)^89^.

### Metabolite profiling from stool samples

Faecal samples (100 mg) were individually ground with liquid nitrogen, and the homogenate was resuspended with prechilled 80% methanol and 0.1% formic acid using vortexing. The samples were incubated on ice for 5 min then centrifuged at 15,000 g and 4 °C for 20 min. Some of the supernatants were diluted with UPLC-MS/MS grade water to a final concentration of 53% methanol. The samples were subsequently transferred to a fresh Eppendorf tube then centrifuged at 15,000 g and 4 °C for 20 min. Finally, the supernatant was injected into the UPLC-MS/MS system^90^.

UHPLC-MS analyses were performed using a Vanquish UHPLC system (Thermo Fisher, Germany) coupled with an Orbitrap Q ExactiveTM HF mass spectrometer (Thermo Fisher, Germany) at Novogene Co., Ltd. (Beijing, China). Samples were injected onto a Hypersil gold column (100 ×2.1 mm, 1.9 μm) using a 17-min linear gradient at a flow rate of 0.2 mL/min. The eluents for the positive polarity mode were eluent A (0.1% FA in water) and eluent B (methanol). The eluents for the negative polarity mode were eluent A (5 mM ammonium acetate, pH 9.0) and eluent B (methanol). The solvent gradient was set as follows: 2% B, 1.5 min; 2%–100% B, 12.0 min; 100% B, 14.0 min; 100%–2% B, 14.1 min; and 2% B, 17 min. The Q Exactive TM HF mass spectrometer was operated in positive/negative polarity mode with a spray voltage of 3.2 kV, a capillary temperature of 320 °C, a sheath gas flow rate of 40 arb, and an aux gas flow rate of 10 arbs.

The raw data files generated by UHPLC-MS/MS were processed using Compound Discoverer 3.1 (CD3.1, Thermo Fisher) to perform peak alignment, peak picking, and quantitation for each metabolite. The main parameters were set as follows: retention time tolerance, 0.2 min; actual mass tolerance, 5 ppm; signal intensity tolerance, 30%; signal/noise ratio, 3; and minimum intensity. After that, peak intensities were normalized to the total spectral intensity. The normalized data was used to predict the molecular formula based on additive ions, molecular ion peaks, and fragment ions, then peaks were matched with the mzCloud (https://www.mzcloud.org/), mzVault, and MassList databases to obtain accurate qualitative and relative quantitative results. Statistical analyses were performed using the statistical software R (R version 3.4.3), Python (Python version 2.7.6), and CentOS (CentOS release 6.6). When the data were not normally distributed, normal transformations were attempted using the area normalization method.

### Metabolite statistical analysis

Data on the fecal metabolome were processed using the Novogene platform. The metabolites were annotated using the KEGG (https://www.genome.jp/kegg/pathway.html) and HMDB (https://hmdb.ca/metabolites) databases. Principal components analysis (PCA) and PLS-DA were performed using metaX^91^ (a flexible and comprehensive software for processing metabolomics data). We applied univariate analysis (t-test) to calculate the statistical significance (P-value). The metabolites with VIP > 1, a *P*-value <0.05, and a fold change (FC) > 1.5 or <0.7 were considered differential metabolites. Volcano plots were used to filter metabolites of interest, and were based on the log2 (FC) and -log10(P-value) of metabolites by ‘ggplot2’ in R. For clustering heat maps, the data were normalized using z-scores of the intensity areas of differential metabolites and were plotted using the ‘pheatmap’ package in R. The correlation between differential metabolites was calculated using the function ‘cor’ in R (method = Pearson). The statistical significance of correlations between differential metabolites was calculated using ‘cor.mtest’ in R. A *P*-value greater than 0.05 was considered as statistically significant, and correlation plots were plotted using the ‘corrplot’ package in R. The functions of these metabolites and metabolic pathways were studied using the KEGG database. Metabolic pathway enrichment was analyzed: when the ratio was x/n > y/N, the metabolic pathway was considered as enriched, and when the *P*-value of the metabolic pathway was <0.05, the metabolic pathway was considered as significantly enriched.

### Metabolite traceability and association analysis with microbiota

The bioinformatics analysis process on the MetOrigin platform^92^ was used to trace the source of metabolites by searching and integrating the databases of seven metabolites (KEGG, HMDB, BIGG, ChEBI, FoodDB, drug database, and toxin and toxin target database [T3DB]). Further, functional enrichment analysis was performed according to the different sources of metabolites^92^. The reference metabolic pathway of the host was from the KEGG database, and the reference metabolic pathway of the microbiota community was from more than 6,800 microbiotas in the integrated database of the MetOrigin platform. The reference metabolic pathway shared by the host and the microorganism was obtained by integrating these two pathways. Combined with the differential microbiota data, the statistical links between the differential fecal metabolites and differential microbiota were first revealed by Spearman analysis, then the KEGG database was utilized to search for microbiotas that might be involved in the associated metabolic responses and to perform associations with metabolites. Sankey network analysis was further utilized to visually demonstrate the biological and statistical links between gut microbiota and fecal metabolites^92^. Finally, network summary analysis was performed to further reveal the statistical and biological significance of correlations between microbiota and metabolites in specific functional pathways^92^. We mapped the KO genes and metabolites with significant differences in the feces of RMs at high and low altitudes together in the “microbial metabolism in diverse environments” pathway using the KEGG Mapper–Color tool (https://www.genome.jp/kegg/mapper/color.html) to reveal the residues of metabolites in feces produced by the main modules of the functioning of RMs gut microbiota.

### Reporting summary

Further information on research design is available in the Nature Portfolio Reporting Summary linked to this article.

## Supplementary information

Supplementary Information

Reporting summary

## Data Availability

The data that support the findings of this study have been deposited into the CNGB Sequence Archive (CNSA)^93^ of China National GeneBank DataBase (CNGBdb)^94^ with accession number CNP0003401.

## Code availability

Code and detailed information are available on github (https://github.com/junsongzhao-junsongzhao/Metagenome-metabolome-gut-microbiota-in-high-altitude-rhesus-macaque-Macaca-mulatta).
