# Supplementary material for: Metagenome and metabolome insights into the energy compensation and exogenous toxin degradation of gut microbiota in high-altitude rhesus macaques (Macaca mulatta)
Source: NPJ Biofilms Microbiomes. 2023 Apr 20;9:20. doi: 10.1038/s41522-023-00387-3 (PMC10119431; doi:10.1038/s41522-023-00387-3)
Supplement: Supplementary file 1 — Supplementary Information [file 41522_2023_387_MOESM1_ESM.pdf]

Metagenome and metabolome insights into the energy compensation and exogenous toxin degradation of gut microbiota in high-altitude rhesus macaques (*Macaca mulatta*)

Junsong Zhao[1, 2†], Yongfang Yao[1†], Diyan Li[3], Wei Zhu[4], Hongtao Xiao[1], Meng Xie[1], Ying Xiong[1], Jiayun Wu[1], Qingyong Ni[4], Mingwang Zhang[4], Huailiang Xu[1*]

[1]College of Life Science, Sichuan Agricultural University, Ya'an 625014, China

[2]College of Agronomy and Life Sciences, Zhaotong University, Zhaotong 657000, China

[3]School of Pharmacy, Chengdu University, Chengdu 610106, China

[4]College of Animal Science and Technology, Sichuan Agricultural University, Chengdu 611130, China

[†]These authors contributed equally to this work.

[*]Correspondence to: Huailiang Xu, College of Life Science, Sichuan Agricultural University, No. 46, Xinkang Road, Yucheng District, Ya'an, Sichuan 625014, P.R. China. Tel: +86-835-2885238. Email: xuhuail@sicau.edu.cn

10 Supplementary Figures

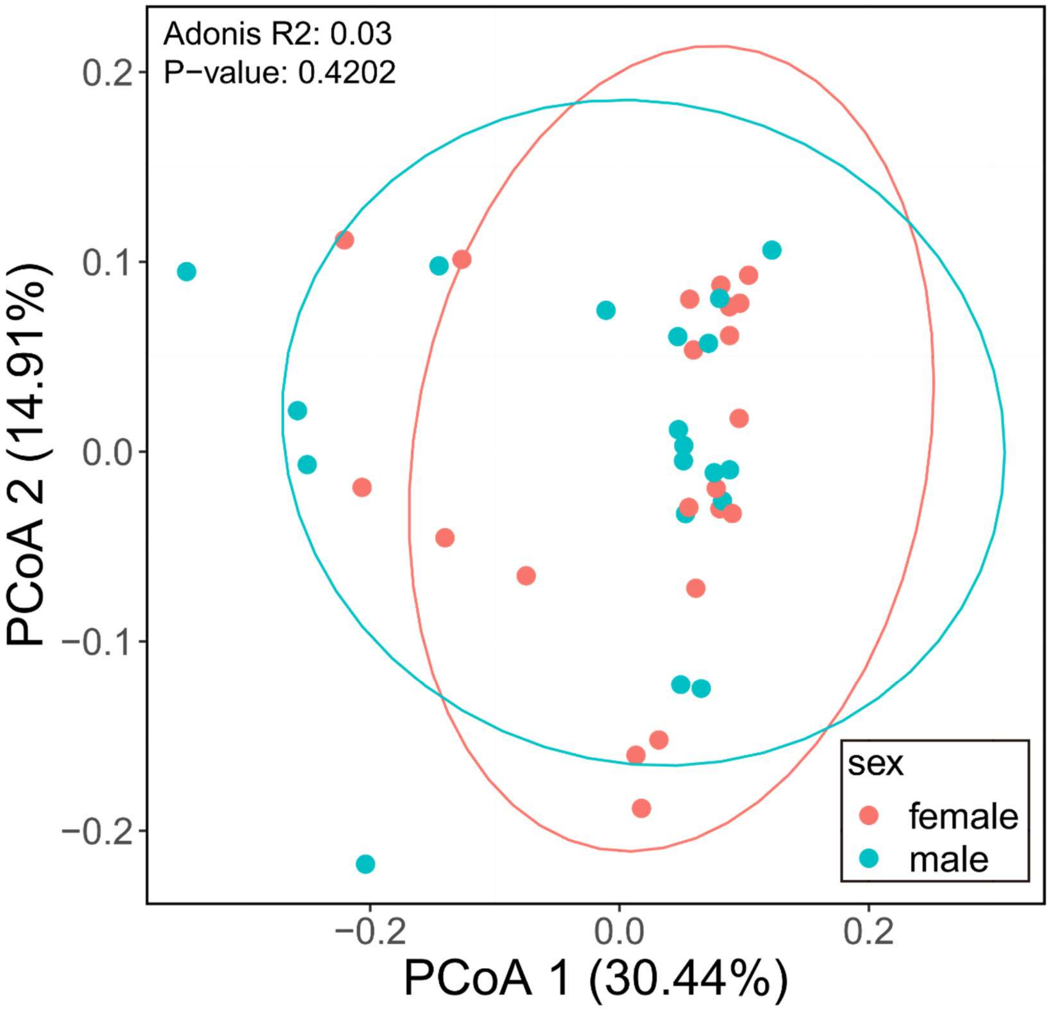

Supplementary Figure. 1 Principal coordinate analysis (PCoA) of individuals between females and males based on gut microbiota species level abundance (Bray-Curtis distance, Adonis test, *P* > 0.05). The ellipse borders represent the 95% confidence interval.

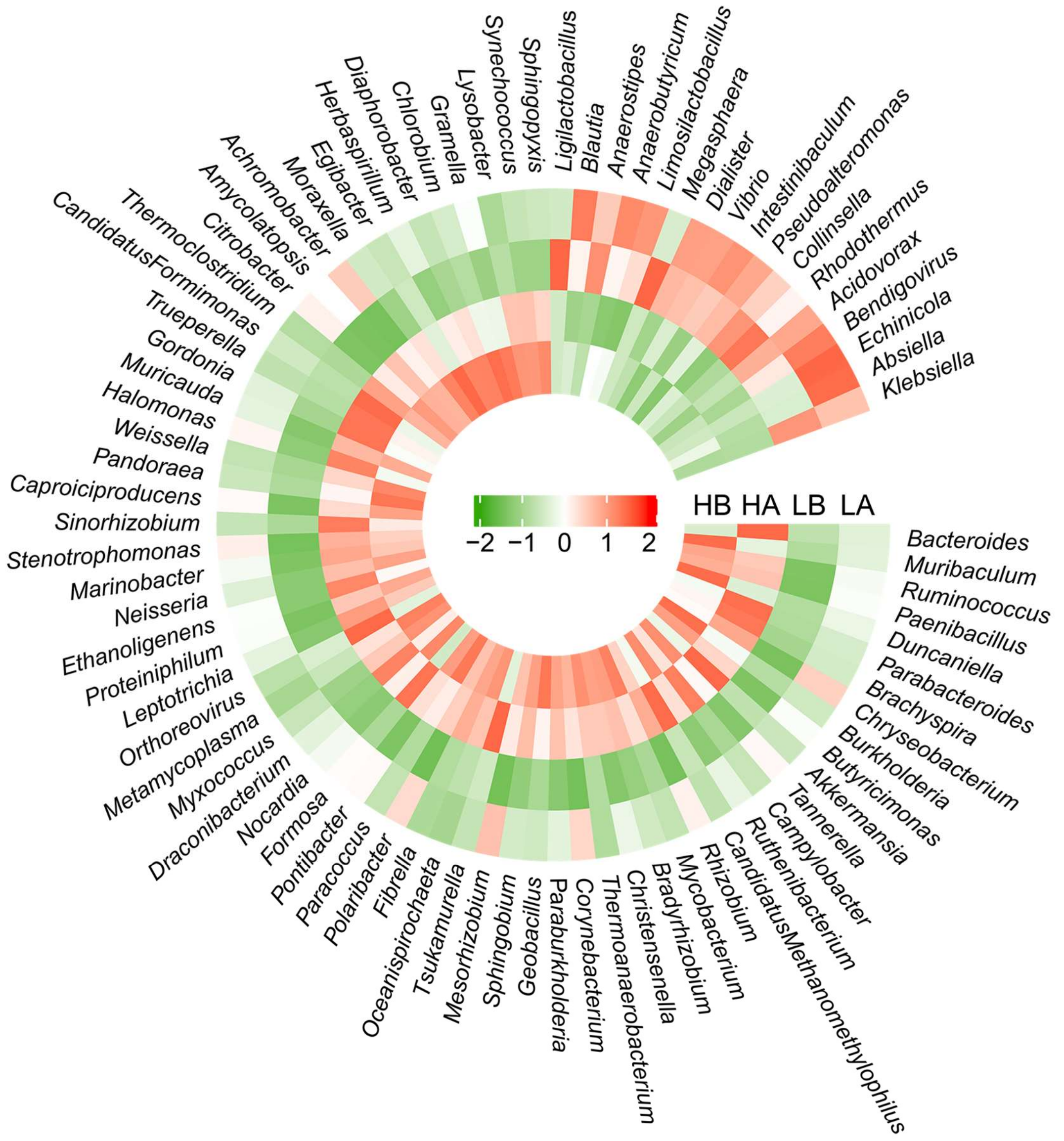

Supplementary Figure. 2 Genus level differential microbiota relative abundance heatmaps of the gut microbiota between high-altitude and low-altitude populations in the RMs (LEfSe analysis, LDA > 2, *P* < 0.01).

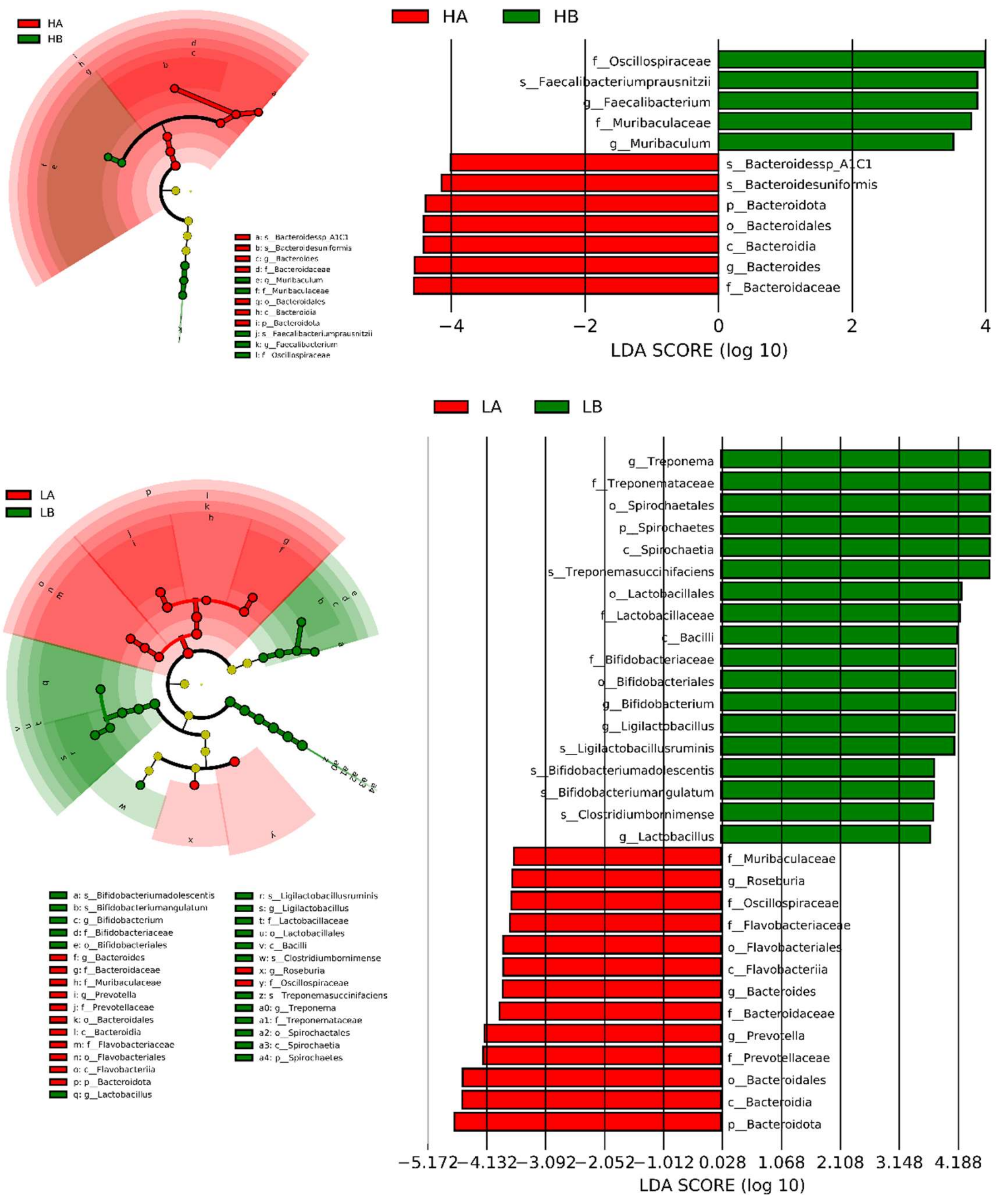

Supplementary Figure. 3 Linear discriminant analysis effect size (LEfSe) analysis of differential gut microbial composition between two different groups at the same altitude in RMs (LDA > 3.5, *P*<0.01).

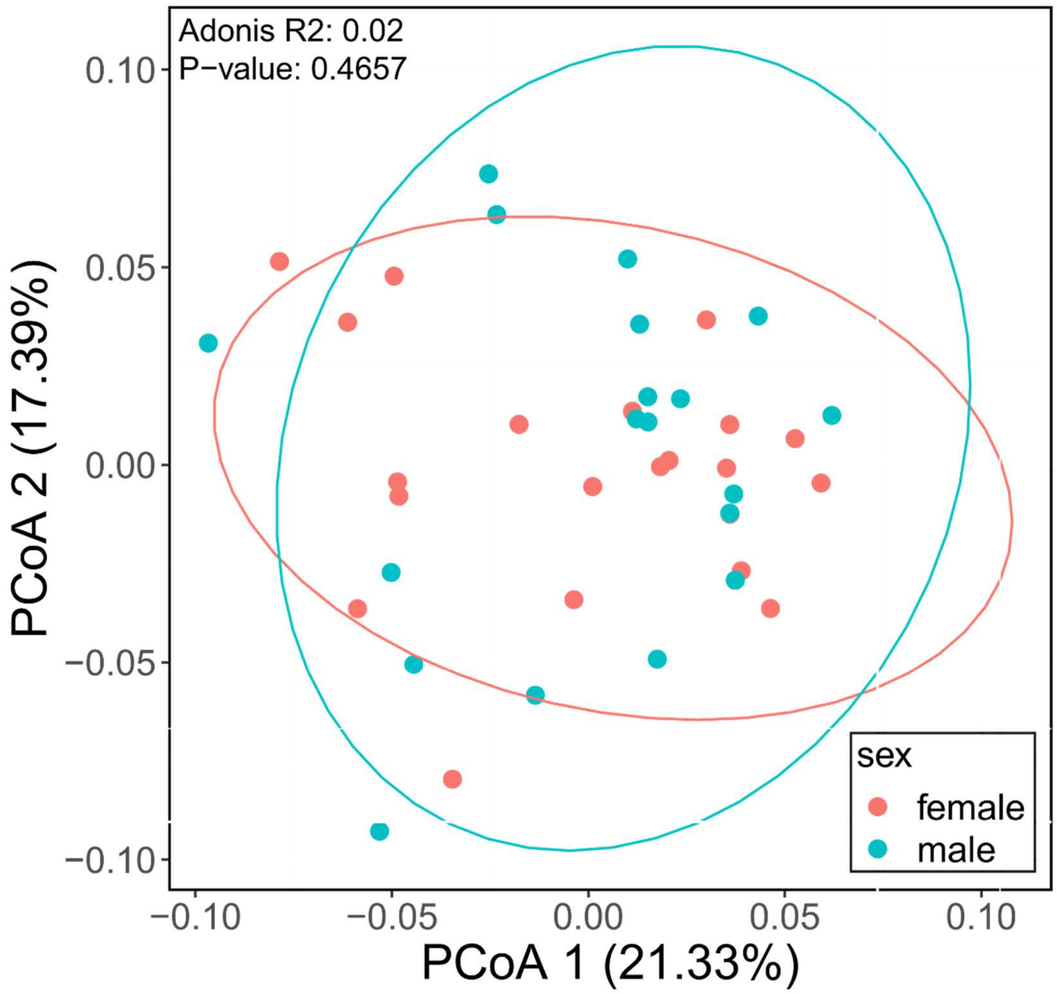

Supplementary Figure. 4 Principal coordinate analysis (PCoA) of individuals between females and males based on Ko genes (Bray-Curtis distance, Adonis test, $P > 0.05$).

Continuation of Supplementary Figure 5

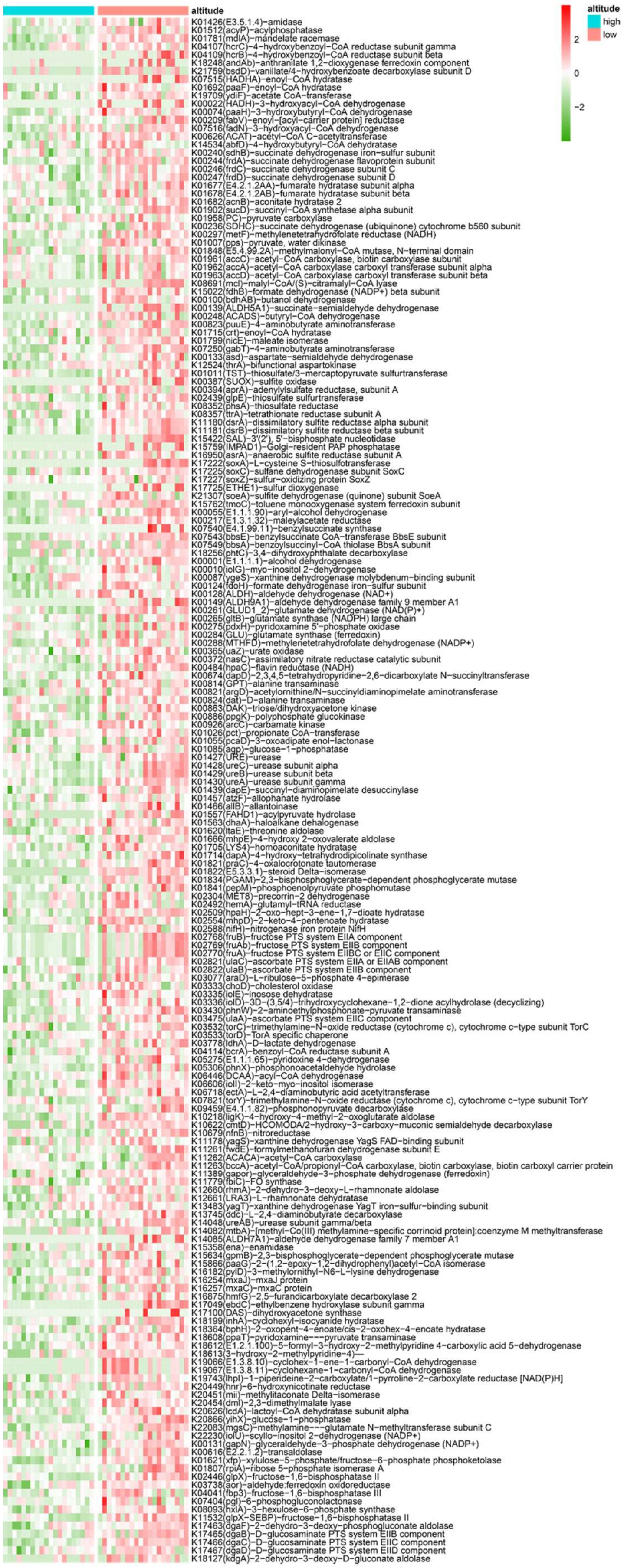

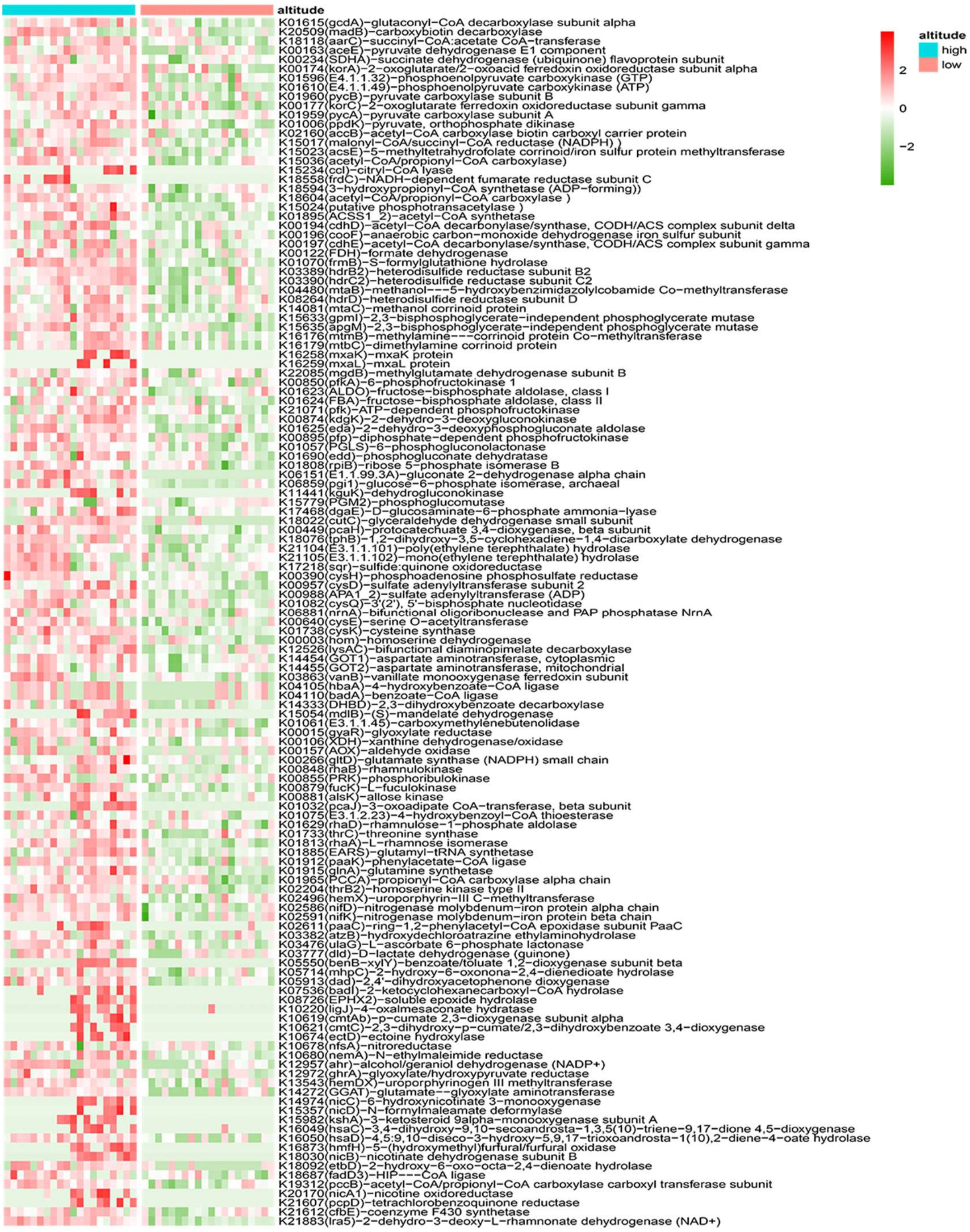

Supplementary Figure. 5 Heatmap of differential Ko gene richness between high and low altitude populations enriched in the pathway "microbial metabolism in diverse environments (map01120)".

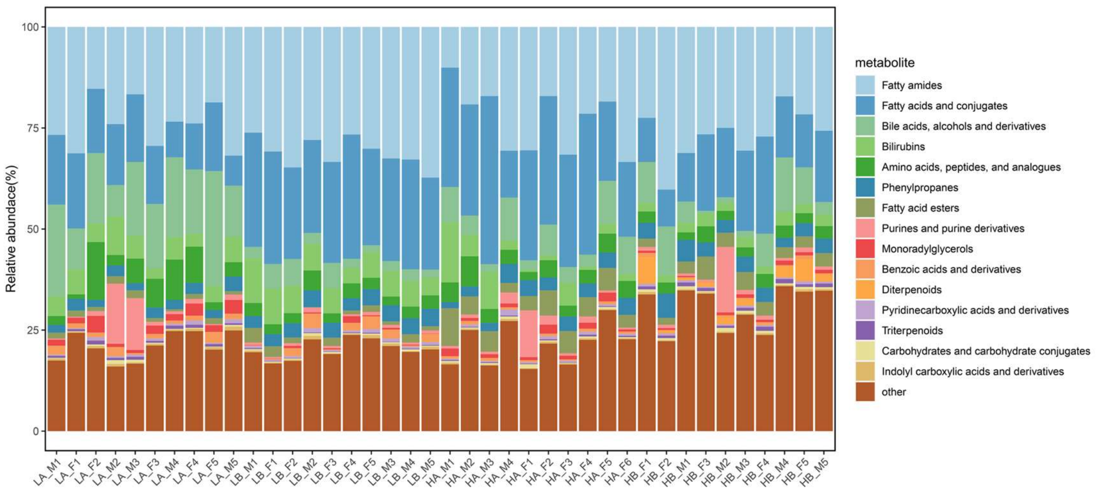

Supplementary Figure. 6 Percentage histogram of HMDB database annotations (Sub class) of RMs fecal metabolites.

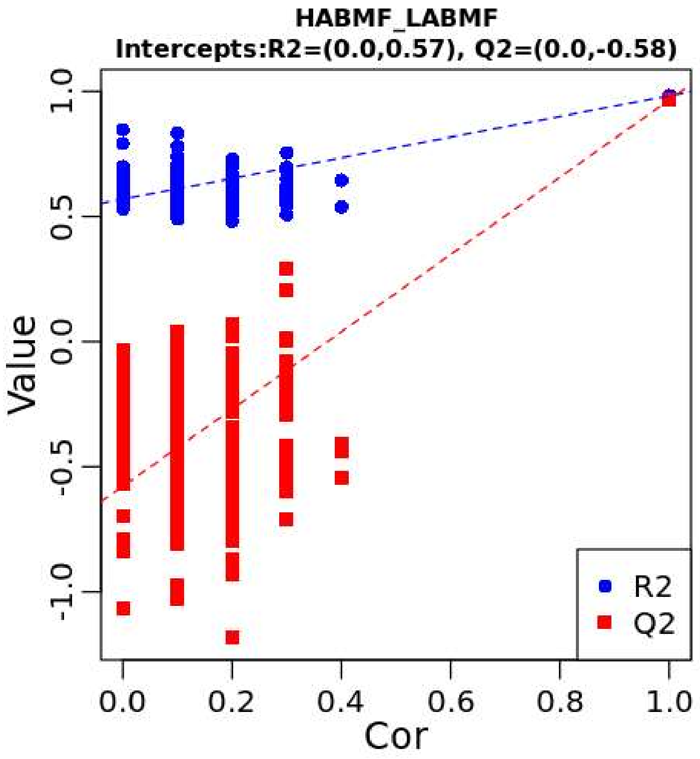

Supplementary Figure. 7 PLS-DA analysis 200 permutation test model verification.

Continuation of Supplementary Figure 8a

a

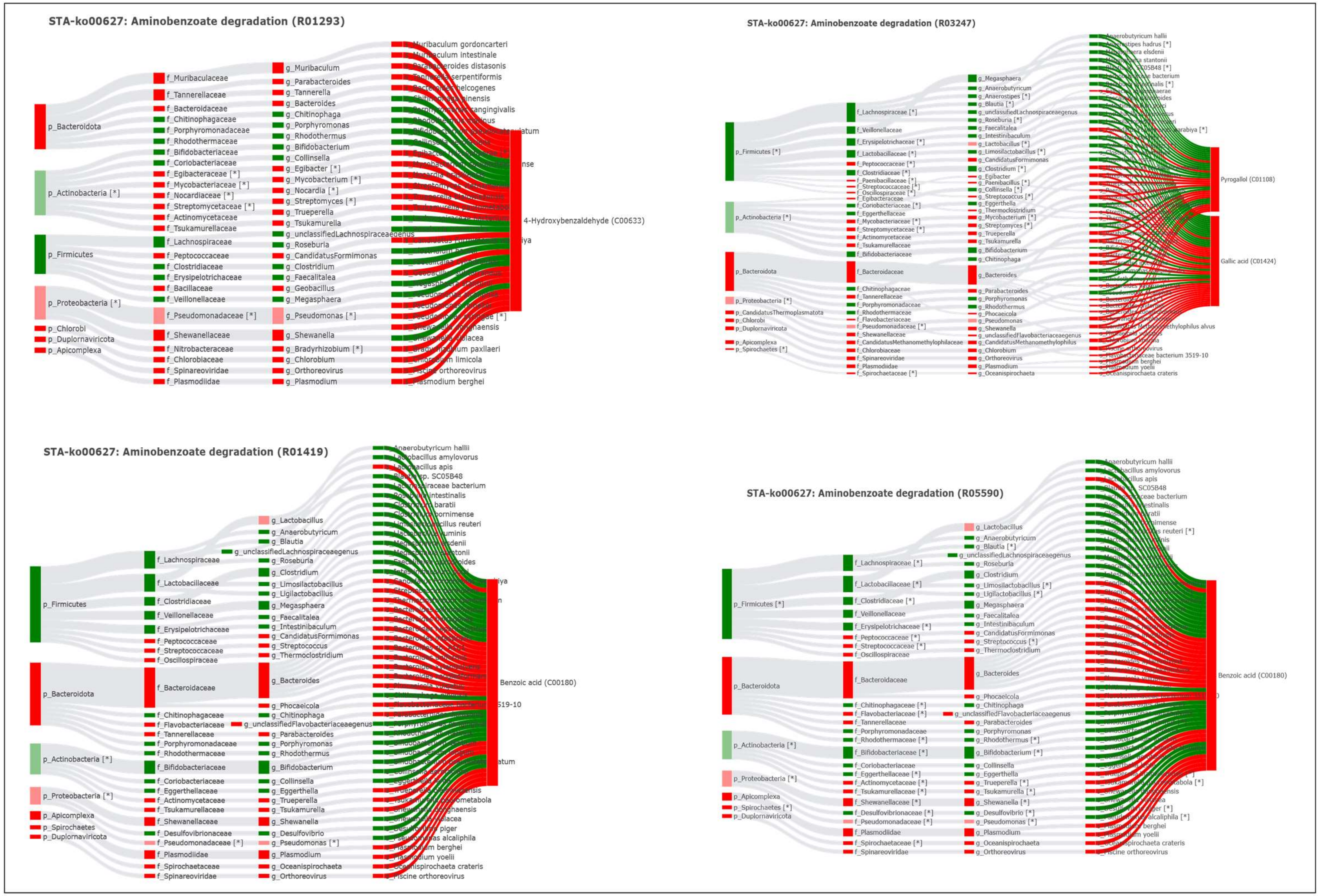

Continuation of Supplementary Figure 8a

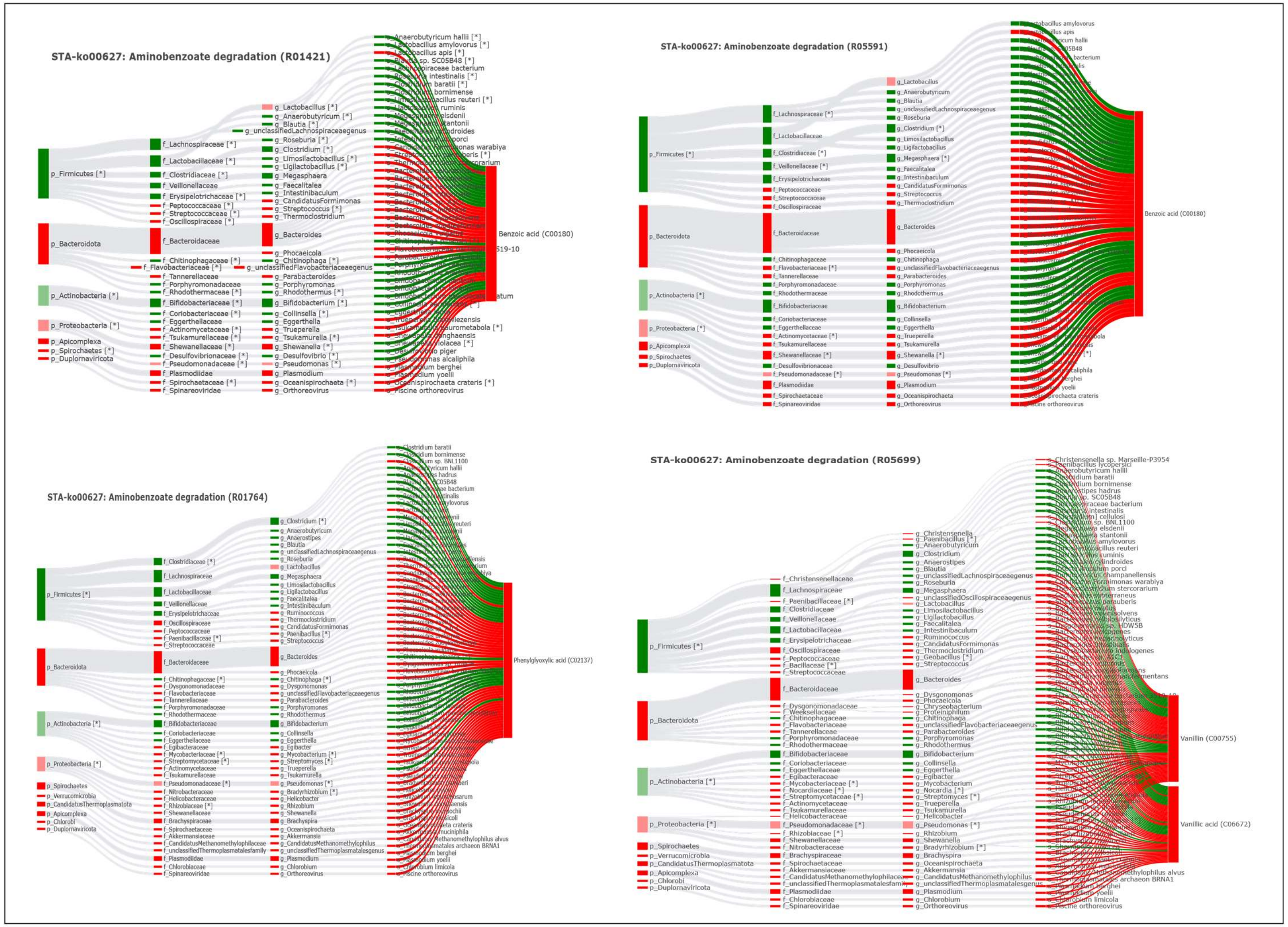

Continuation of Supplementary Figure 8a

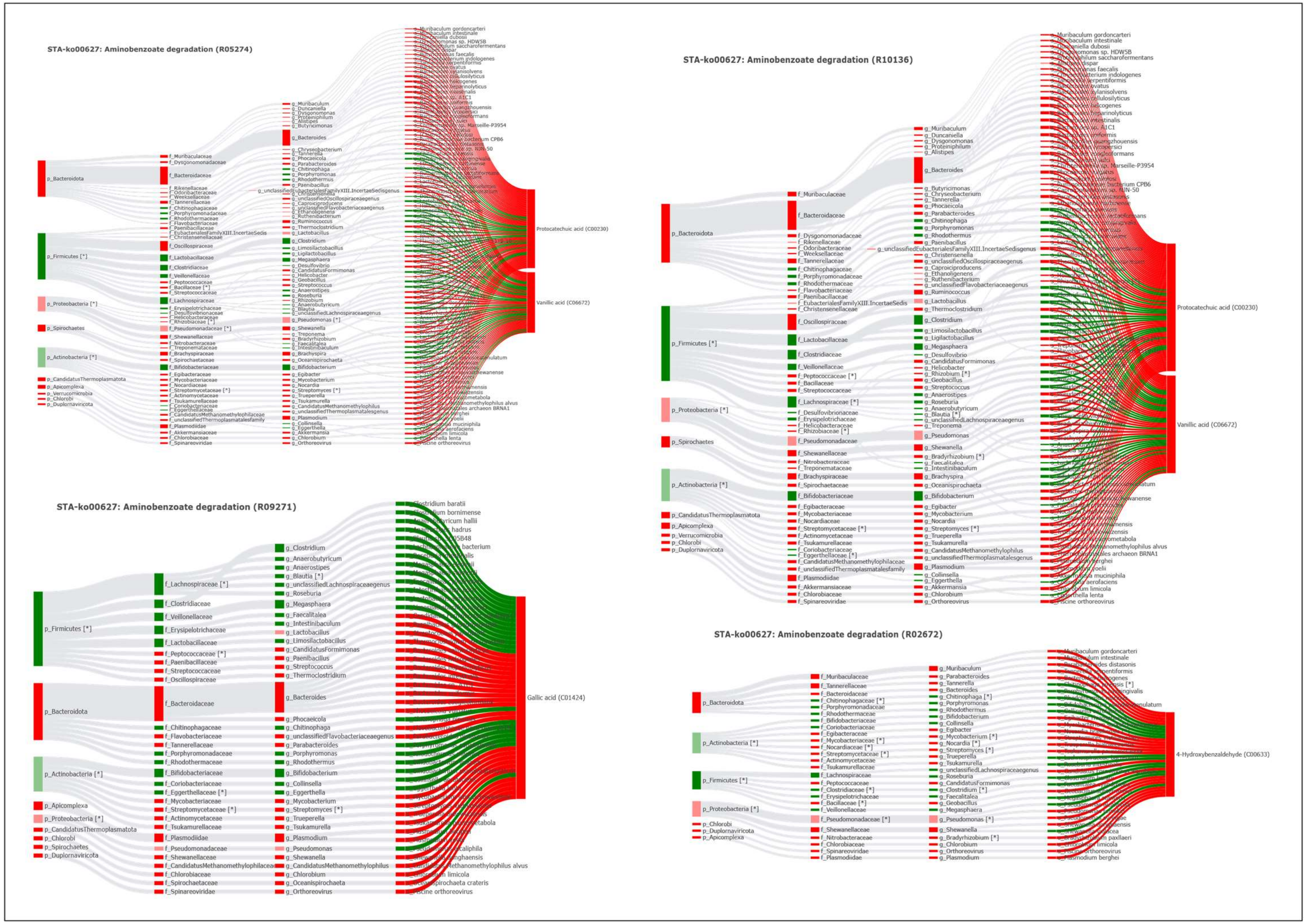

Continuation of Supplementary Figure 8

b

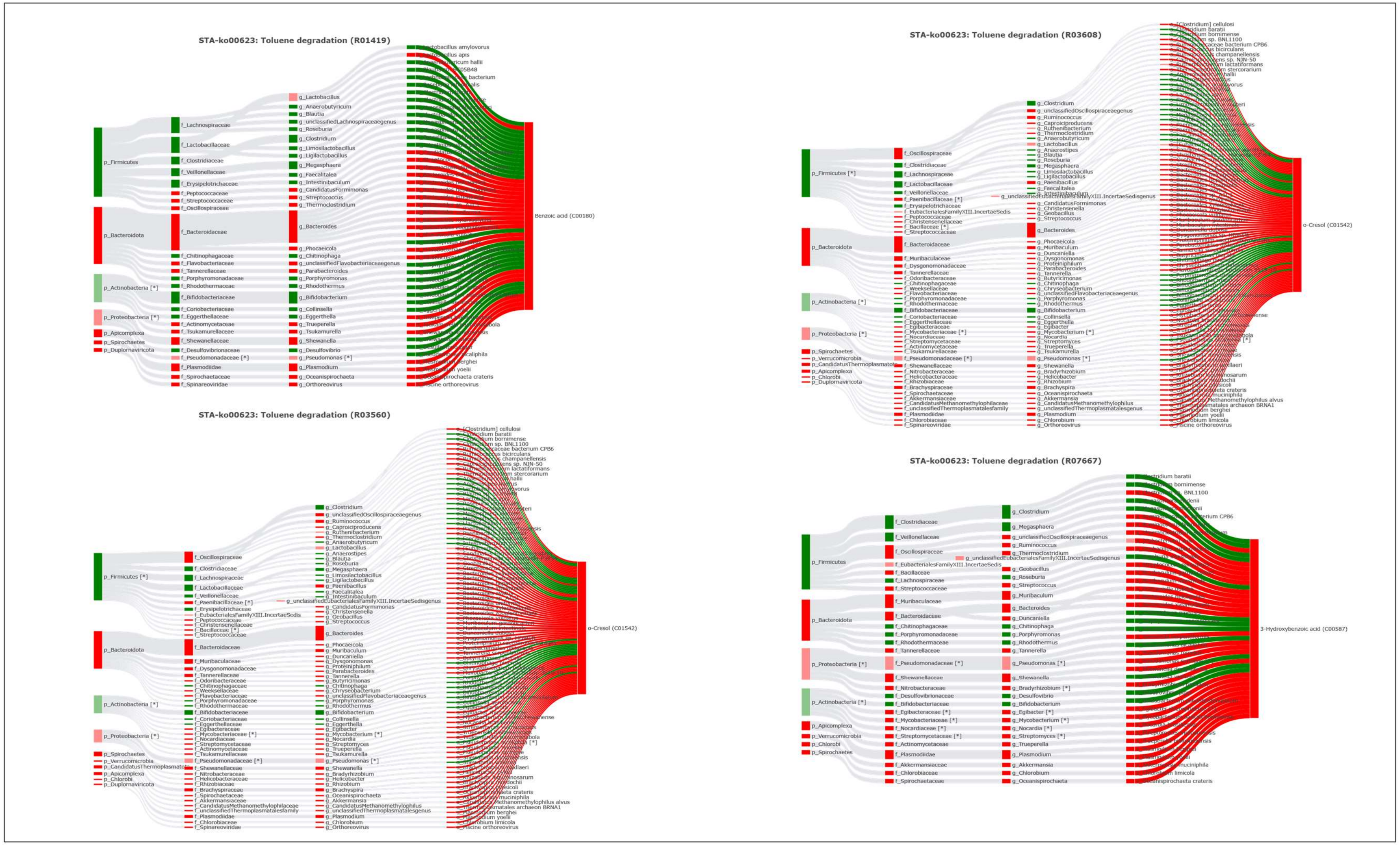

Continuation of Supplementary Figure 8

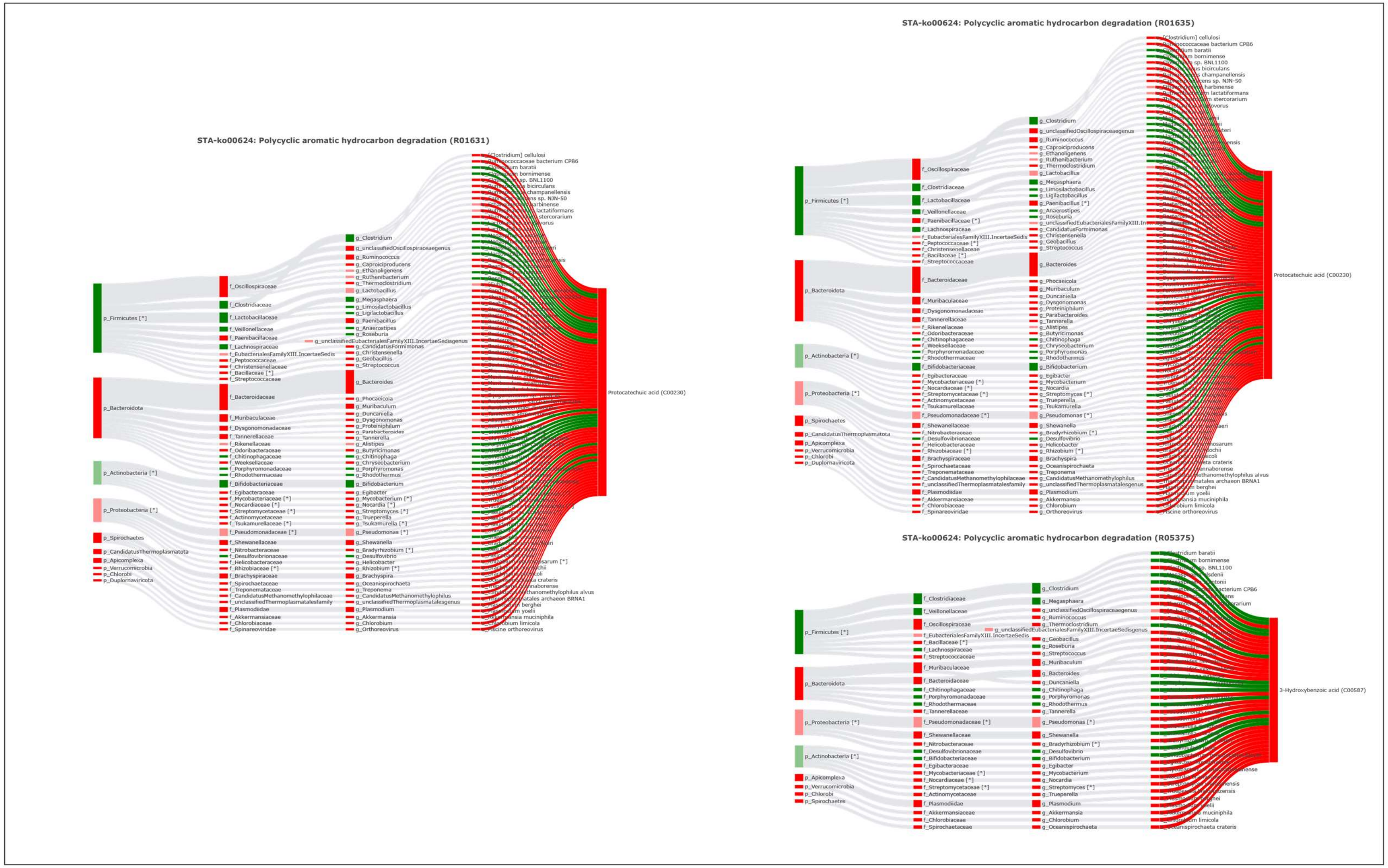

Continuation of Supplementary Figure 8

d

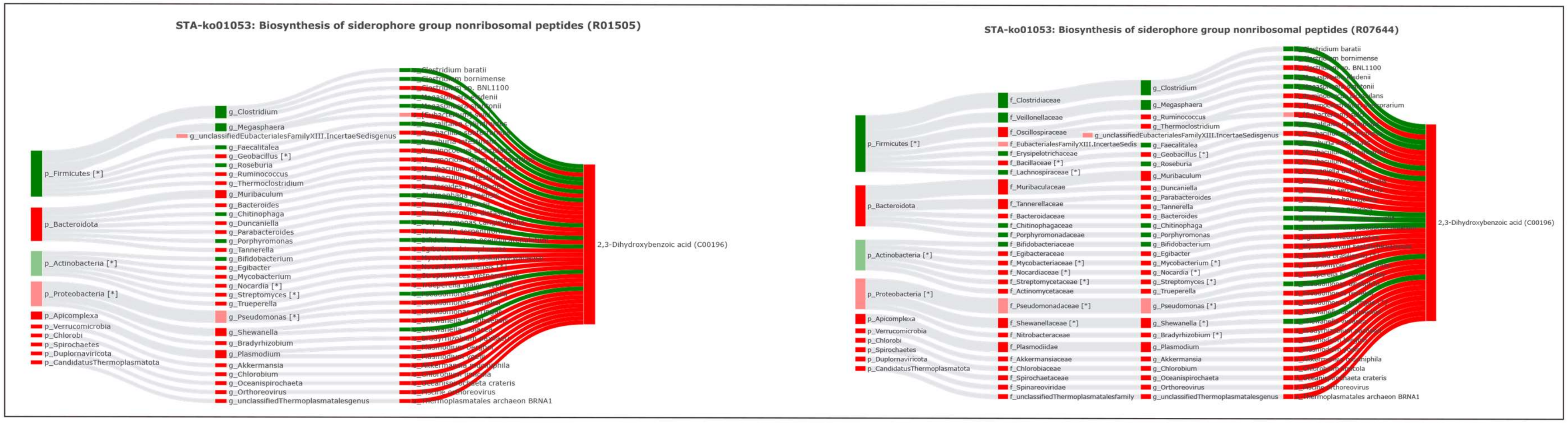

Supplementary Figure. 8 Microbiota specific metabolic pathways (Hypergeometric test, log0.05 p-value > 1) STA-Sankey network diagram analysis reveal close connections between microbiota and metabolites. (a). STA-ko00627: Aminobenzoate degradation; (b). STA-ko00623: Toluene degradation; (c). STA-ko00624: Polycyclic aromatic hydrocarbon degradation; (d). STA-ko01053: Biosynthesis of siderophore group nonribosomal peptides. The dark red (or green) bars indicate the microbes or metabolites that are significantly higher (or lower) in the high-altitude population (FC>1 or FC<1, $P < 0.05$); light red (or green) bars indicate the microbes or metabolites that are higher (or lower) in the high-altitude population (FC>1 or FC<1, $P \geq 0.05$); dark grey bars indicate the microbes or metabolites with no change (FC = 1); Dark red (or green) bands indicate significant positive (or negative) correlations (Spearman correlation test; R>or R<0, $P < 0.05$); Light red (or green) bands indicate positive (or negative) correlations without statistical significance (Spearman correlation test; R>or R<0, $P \geq 0.05$); Light gray bands indicate reference relationships searched through database. The asterisk (*) indicates an abiologically significant correlation with the metabolite. Relevant analysis is completed based on MetOrigin platform.

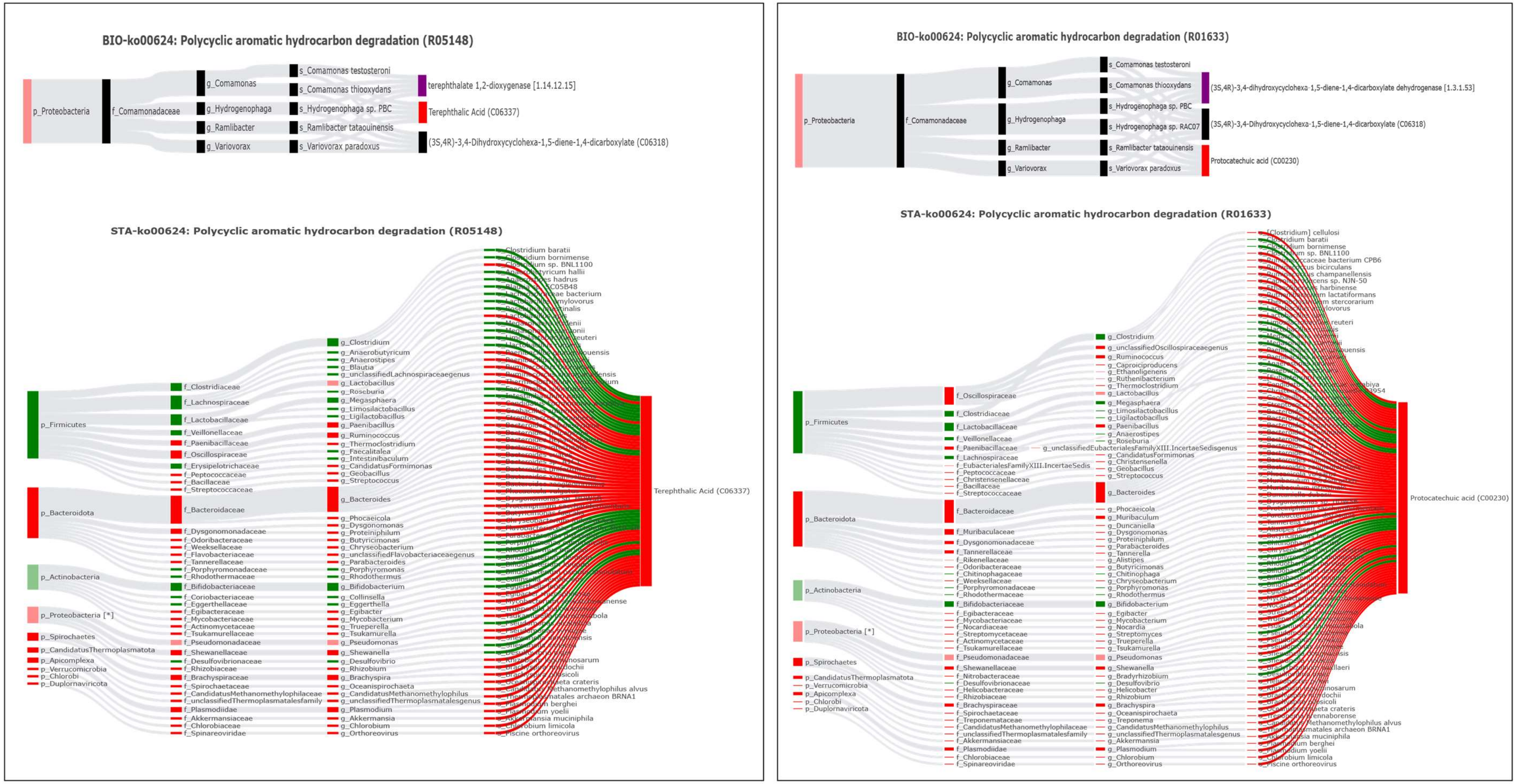

Supplementary Figure. 9 Polycyclic aromatic hydrocarbon degradation（R01548 and R01633) pathways BIO-Sankey and STA-Sankey network diagram revealed links between microbiota and metabolites. In the BIO-Sankey network diagram, Dark red bars indicate the microbes or metabolites that are significantly higher in the high-altitude population (FC>1, P < 0.05); Light red bars indicate the microbes or metabolites that are higherin the high-altitude population (FC>1, P

≥0.05); Black bars indicate the microbes or metabolites in the reference database; Purple bars indicate the metabolic enzymes; Light gray bands indicate reference relationships searched through database. In the network summary analysis diagram, the diamond and dot shapes indicate the related metabolites and microorganisms, respectively. Red (or green) nodes indicate populations at significantly higher (or lower) altitude. Connecting lines in red (or green) indicate positive (or negative) correlations between microbes and metabolites. In the STA-Sankey network diagram, Dark red (or green) bars indicate the microbes or metabolites that are significantly higher (or lower) in the high-altitude population (FC>1 or FC<1, P < 0.05); Light red (or green) bars indicate the microbes or metabolites that are higher (or lower) in the high-altitude population (FC>1 or FC<1, P ≥0.05); Dark red (or green) bands indicate significant positive (or negative) correlations (Spearman correlation test; R>or R<0, P < 0.05); Light gray bands indicate reference relationships searched through database. The FC refers to the fold change, which is the ratio of the mean of all biological repeated quantitative values of each metabolite in the comparison group; P-value is calculated by T-test and represents the level of difference significance. The asterisk (*) indicates an abiologically significant correlation with the metabolite. Relevant analysis is completed based on MetOrigin platform.

Continuation of Supplementary Figure 10

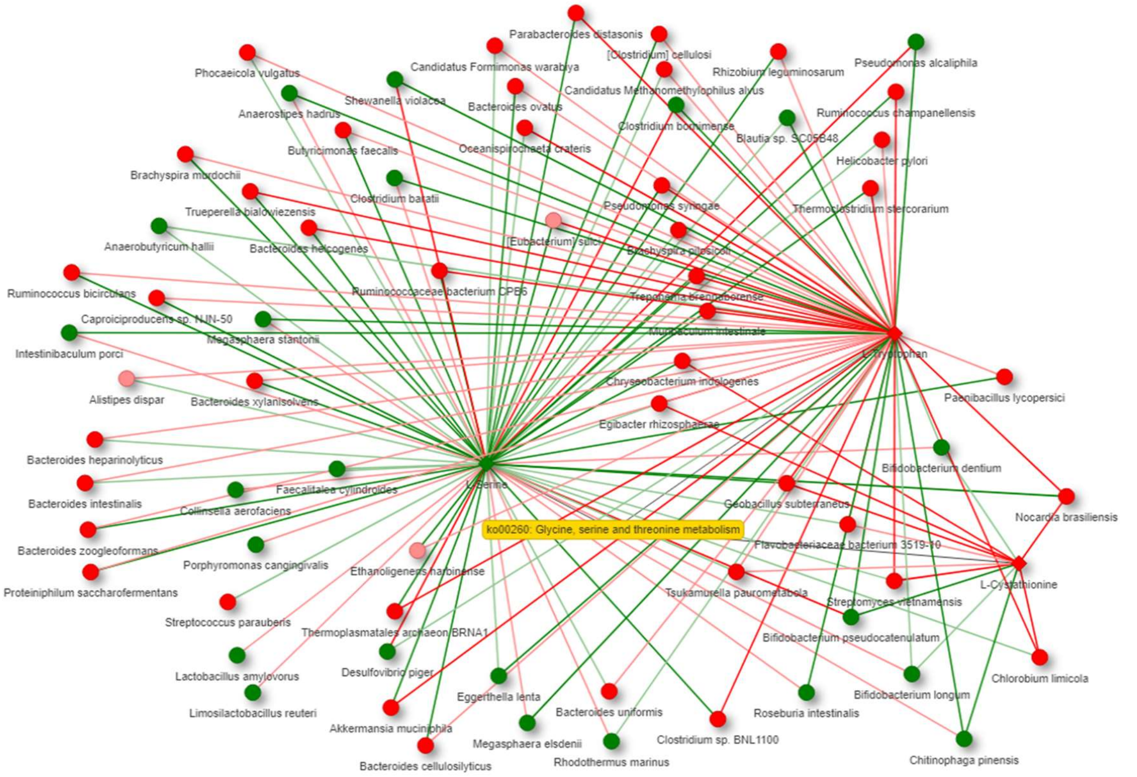

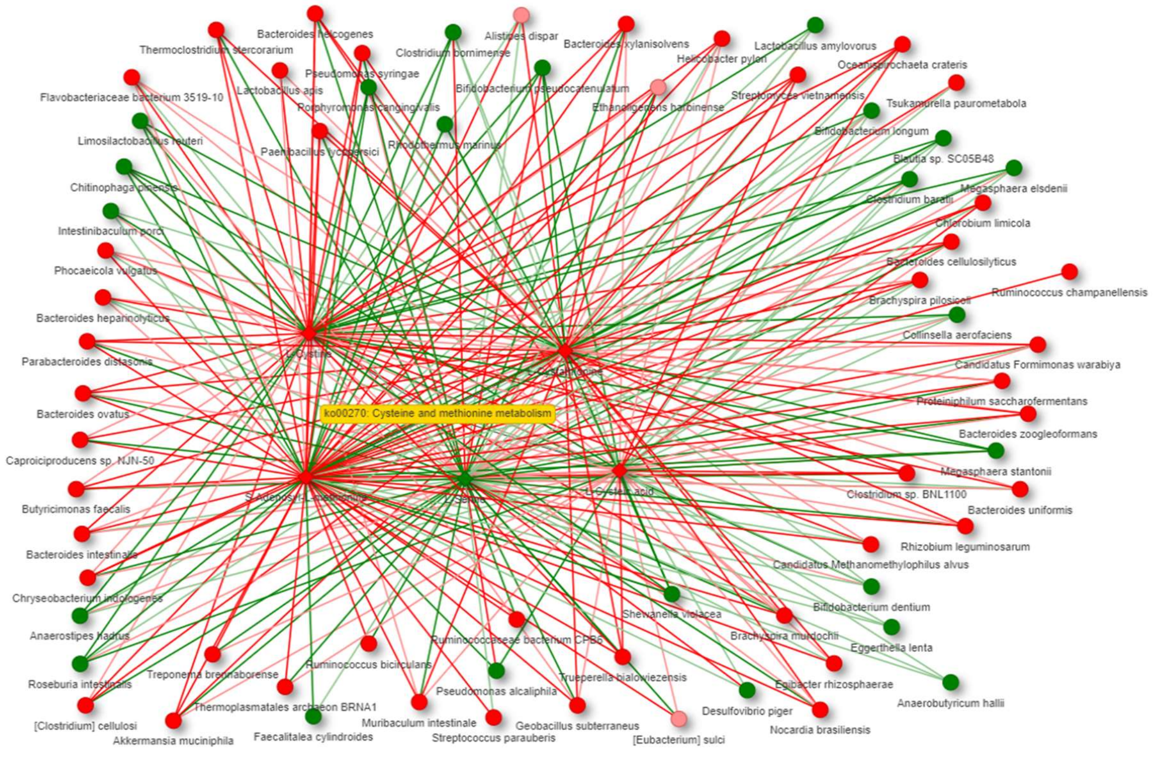

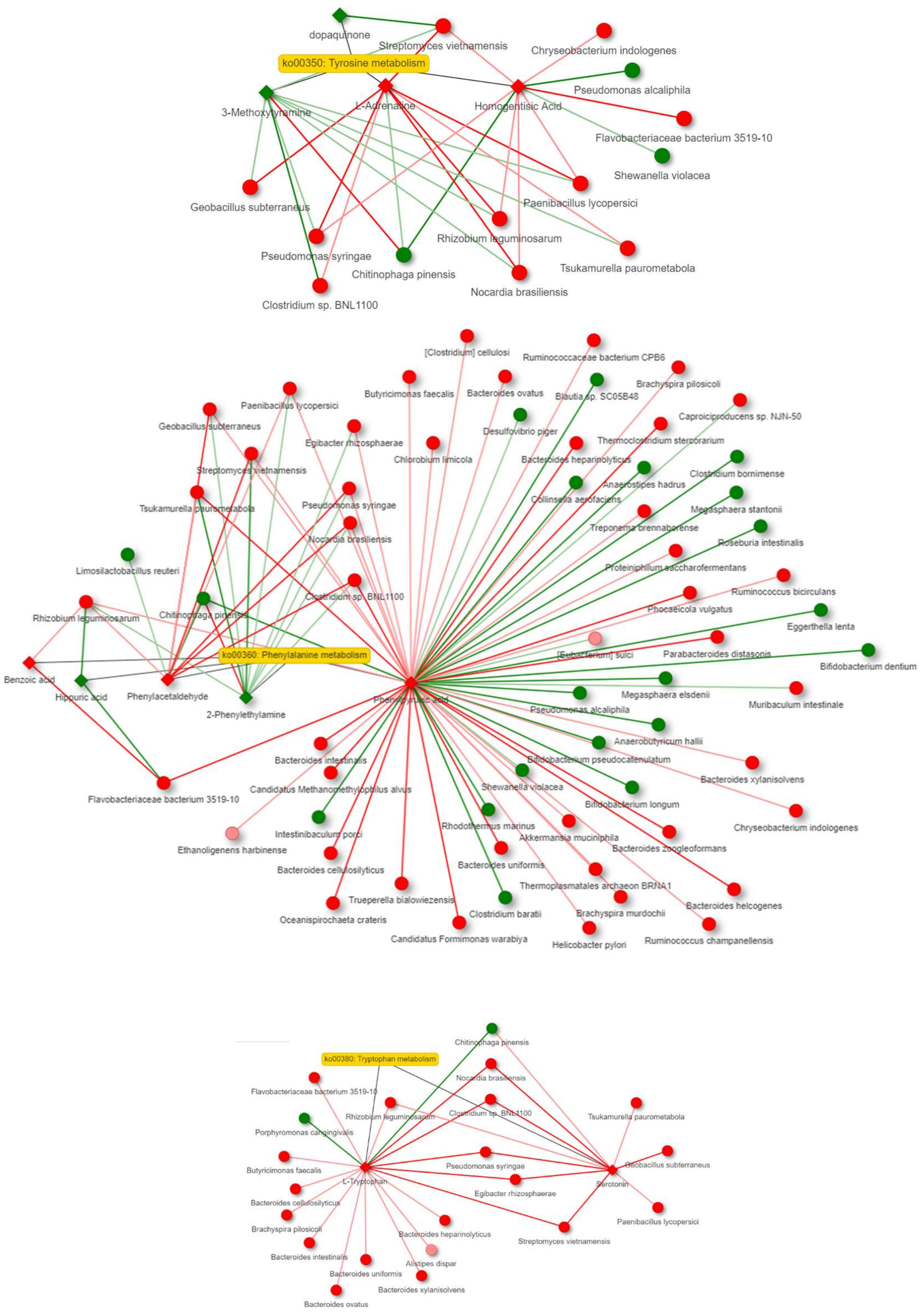

Supplementary Figure. 10 Derived from the microbial to metabolite correlation network summary analysis in microbe andhost co-metabolic pathways (Hypergeometric test, log0.05 p-value > 1). the diamond and dot shapes indicate the related metabolites and microorganisms, respectively. Red (or green) nodes indicate populations at significantly higher (or lower) altitude. Connecting lines in red (or green) indicate positive (or negative) correlations between microbes and metabolites.

Supplementary Table 1 Functional composition of gut flora based on differences between high and low altitude populations derived from Previous research report

Species	Kingdom	LDA	Populations with significantly higher abundance	Functional classification	Reference
Akkermansia muciniphila	Bacteria	3.010803116	High.altitude	Dietary digestion and energy substance metabolism	[1]
Bacteroides ovatus	Bacteria	3.551583281	High.altitude	Dietary digestion and energy substance metabolism	[2]
Bacteroides xylanisolvens	Bacteria	3.066352432	High.altitude	Dietary digestion and energy substance metabolism	[3]
Candidatus Formimonas warabiya	Bacteria	2.591048165	High.altitude	Dietary digestion and energy substance metabolism	[4]
Clostridium cellulosi	Bacteria	2.832983124	High.altitude	Dietary digestion and energy substance metabolism	[5]
Ethanoligenens harbinense	Bacteria	3.017138868	High.altitude	Dietary digestion and energy substance metabolism	[6]
Lactobacillus apis	Bacteria	2.530501426	High.altitude	Dietary digestion and energy substance metabolism	[7]
Parabacteroides distasonis	Bacteria	3.315920609	High.altitude	Dietary digestion and energy substance metabolism	[8]
Proteiniphilum saccharofermentans	Bacteria	2.823322069	High.altitude	Dietary digestion and energy substance metabolism	[9]
Pseudomonas reinekei	Bacteria	2.376256907	High.altitude	Dietary digestion and energy substance metabolism	[10]
Ruminococcaceae bacterium CPB6	Bacteria	2.859799795	High.altitude	Dietary digestion and energy substance metabolism	[11]
Ruminococcus bicirculans	Bacteria	3.487094113	High.altitude	Dietary digestion and energy substance metabolism	[12]
Ruminococcus champanellensis	Bacteria	3.7959257	High.altitude	Dietary digestion and energy substance metabolism	[13]
Ruthenibacterium lactatiformans	Bacteria	3.416319273	High.altitude	Dietary digestion and energy substance metabolism	[14]
Thermoclostridium stercorarium	Bacteria	2.675688411	High.altitude	Dietary digestion and energy substance metabolism	[15]
Clostridium sp. BNL1100	Bacteria	2.710071448	High.altitude	Dietary digestion and energy substance metabolism	[16]
Tannerella serpentiformis	Bacteria	3.128223766	High.altitude	Potentially pathogenic or disease related	[17]
Bacteroides helcogenes	Bacteria	3.54017983	High.altitude	Potentially pathogenic or disease related	[18]
Brachyspira murdochii	Bacteria	2.739349956	High.altitude	Potentially pathogenic or disease related	[19]

Continuation of Supplementary Table 1

Species	Kingdom	LDA	Populations with significantly higher abundance	Functional classification	Reference
Brachyspira pilosicoli	Bacteria	2.830071114	High.altitude	Potentially pathogenic or disease related	[20]
Chryseobacterium indologenes	Bacteria	2.620136427	High.altitude	Potentially pathogenic or disease related	[21]
Helicobacter pylori	Bacteria	2.694369456	High.altitude	Potentially pathogenic or disease related	[22]
Nocardia brasiliensis	Bacteria	2.59948869	High.altitude	Potentially pathogenic or disease related	[23]
Streptococcus parauberis	Bacteria	2.546854799	High.altitude	Potentially pathogenic or disease related	[24]
Paenibacillus lycopersici	Bacteria	2.594538466	High.altitude	Other	[25]
Anaerostipes hadrus	Bacteria	4.01534169	Low.altitude	Dietary digestion and energy substance metabolism	[26]
Clostridium bornimense	Bacteria	3.809172377	Low.altitude	Dietary digestion and energy substance metabolism	[27]
Collinsella aerofaciens	Bacteria	3.28733749	Low.altitude	Dietary digestion and energy substance metabolism	[28]
Megasphaera elsdenii	Bacteria	3.502271686	Low.altitude	Dietary digestion and energy substance metabolism	[29]
Anaerobutyricum hallii	Bacteria	3.980629913	Low.altitude	Immunomodulation	[30]
Limosilactobacillus reuteri	Bacteria	3.687253489	Low.altitude	Immunomodulation	[31]
Desulfovibrio piger	Bacteria	3.17571854	Low.altitude	Potentially pathogenic or disease related	[32]
Porphyromonas cangingivalis	Bacteria	2.954281259	Low.altitude	Potentially pathogenic or disease related	[33]
Pseudomonas alcaliphila	Bacteria	2.59058746	Low.altitude	Potentially pathogenic or disease related	[34]
Lachnospiraceae bacterium	Bacteria	3.251491374	Low.altitude	Other	[35]

REFERENCE

[1] Belzer C, de Vos WM. Microbes inside--from diversity to function: the case of Akkermansia. ISME J. 2012 Aug;6(8):1449-58.

[2] Centanni M, Bell TJ, Sims IM, Tannock GW. Preferential use of plant glycans for growth by Bacteroides ovatus. Anaerobe. 2020 Dec;66:102276.

[3]Despres J, Forano E, Lepercq P, Comtet-Marre S, Jubelin G, Yeoman CJ, Miller ME, Fields CJ, Terrapon N, Le Bourvellec C, Renard CM, Henrissat B, White BA, Mosoni P. Unraveling the pectinolytic function of Bacteroides xylanisolvens using a RNA-seq approach and mutagenesis. BMC Genomics. 2016 Feb 27;17:147.

[4]Holland SI, Ertan H, Montgomery K, Manefield MJ, Lee M. Novel dichloromethane-fermenting bacteria in the Peptococcaceae family. ISME J. 2021 Jun;15(6):1709-1721.

[5]Yang M, Zhang KD, Zhang PY, Zhou X, Ma XQ, Li FL. Synergistic Cellulose Hydrolysis Dominated by a Multi-Modular Processive Endoglucanase from Clostridium cellulosi. Front Microbiol. 2016 Jun 15;7:932.

[6]Li Z, Liu B, Cui H, Ding J, Li H, Xie G, Ren N, Xing D. The complete genome sequence of Ethanoligenens harbinense reveals the metabolic pathway of acetate-ethanol fermentation: A novel understanding of the principles of anaerobic biotechnology. Environ Int. 2019 Oct;131:105053.

[7]Ghamry M, Li L, Zhao W. A metabolomics comparison of Lactobacillus communities isolated from breast milk and camel milk and Lactobacillus apis isolated from bee gut during cereals-based fermentation vs. Lactobacillus plantarum as a reference Lebensmittel-wissenschaft + [i.e. Und] Technologie. Food Science + technology. Science + Technologie Alimentaire. 2021 Jul;146:Not Available.

[8]Wang K, Liao M, Zhou N, Bao L, Ma K, Zheng Z, Wang Y, Liu C, Wang W, Wang J, Liu SJ, Liu H. Parabacteroides distasonis Alleviates Obesity and Metabolic Dysfunctions via Production of Succinate and Secondary Bile Acids. Cell Rep. 2019 Jan 2;26(1):222-235.e5.

[9]Kabaivanova L, Hubenov V, Dimitrova L, Simeonov I, Wang H, Petrova P. Archaeal and Bacterial Content in a Two-Stage Anaerobic System for Efficient Energy Production from Agricultural Wastes. Molecules. 2022 Feb 23;27(5):1512.

[10]Priyanka P, Kinsella G, Henehan GT, Ryan BJ. Isolation, purification and characterization of a novel solvent stable lipase from Pseudomonas reinekei. Protein Expr Purif. 2019 Jan;153:121-130.

[11]Tao Y, Zhu X, Wang H, Wang Y, Li X, Jin H, Rui J. Complete genome sequence of Ruminococcaceae bacterium CPB6: A newly isolated culture for efficient n-caproic acid production from lactate. J Biotechnol. 2017 Oct 10;259:91-94.

[12]Wegmann U, Louis P, Goesmann A, Henrissat B, Duncan SH, Flint HJ. Complete genome of a new Firmicutes species belonging to the dominant human colonic microbiota ('Ruminococcus bicirculans') reveals two chromosomes and a selective capacity to utilize plant glucans. Environ Microbiol. 2014 Sep;16(9):2879-90.

[13]Ben David Y, Dassa B, Borovok I, Lamed R, Koropatkin NM, Martens EC, White BA, Bernalier-Donadille A, Duncan SH, Flint HJ, Bayer EA, Moraïs S. Ruminococcal cellulosome systems from rumen to human. Environ Microbiol. 2015 Sep;17(9):3407-26.

[14]Shkoporov AN, Chaplin AV, Shcherbakova VA, Suzina NE, Kafarskaia LI, Bozhenko VK, Efimov BA. Ruthenibacterium lactatiformans gen. nov., sp. nov., an anaerobic, lactate-producing member of the family Ruminococcaceae isolated from human faeces. Int J Syst Evol Microbiol. 2016 Aug;66(8):3041-3049.

[15]Wang N, Yan Z, Liu N, Zhang X, Xu C. Synergy of Cellulase Systems between Acetivibrio thermocellus and Thermoclostridium stercorarium in Consolidated-Bioprocessing for Cellulosic Ethanol. Microorganisms. 2022 Feb 24;10(3):502

[16]Li LL, Taghavi S, Izquierdo JA, van der Lelie D. Complete genome sequence of Clostridium sp. strain BNL1100, a cellulolytic mesophile isolated from corn stover.

J Bacteriol. 2012 Dec;194(24):6982-3.
[17]Kendlbacher FL, Bloch S, Hager-Mair FF, Bacher J, Janesch B, Thurnheer T, Andrukhov O, Schäffer C. Multispecies biofilm behavior and host interaction support the association of Tannerella serpentiformis with periodontal health. Mol Oral Microbiol. 2022 Aug 14.
[18]Pati A, Gronow S, Zeytun A, Lapidus A, Nolan M, Hammon N, Deshpande S, Cheng JF, Tapia R, Han C, Goodwin L, Pitluck S, Liolios K, Pagani I, Ivanova N, Mavromatis K, Chen A, Palaniappan K, Land M, Hauser L, Chang YJ, Jeffries CD, Detter JC, Brambilla E, Rohde M, Göker M, Woyke T, Bristow J, Eisen JA, Markowitz V, Hugenholtz P, Kyrpides NC, Klenk HP, Lucas S. Complete genome sequence of Bacteroides helcogenes type strain (P 36-108). Stand Genomic Sci. 2011 Feb 22;4(1):45-53.
[19]Jensen TK, Christensen AS, Boye M. Brachyspira murdochii colitis in pigs. Vet Pathol. 2010 Mar;47(2):334-8.
[20]Pandey A, Humbert MV, Jackson A, Passey JL, Hampson DJ, Cleary DW, La Ragione RM, Christodoulides M. Evidence of homologous recombination as a driver of diversity in Brachyspira pilosicoli. Microb Genom. 2020 Dec;6(12):mgen00047
[21]Hsieh MC, Yang SY, Liu YL, Lin CP. Chryseobacterium indologenes keratitis-A case report. Kaohsiung J Med Sci. 2020 Jul;36(7):563-564.
[22]Thorell K, Lehours P, Vale FF. Genomics of Helicobacter pylori. Helicobacter. 2017 Sep;22 Suppl 1.
[23]Mangieri NA, Guevara Nuñez D, Echavarría G, Bertona E, Castello L, Benchetrit G, De Paulis AN. Nocardiosis esporotricoide por Nocardia brasiliensis [Sporotrichoid nocardiosis by Nocardia brasiliensis]. Rev Argent Microbiol. 2021 Jan-Mar;53(1):43-47. Spanish.
[24]Huan SJKW, Tan JSW, Chin AYH. Streptococcus parauberis infection of the hand. J Hand Surg Eur Vol. 2021 Jan;46(1):83-84.
[25]Lee SA, Kim TW, Heo J, Sang MK, Song J, Kwon SW, Weon HY. Paenibacillus lycopersici sp. nov. and Paenibacillus rhizovicinus sp. nov., isolated from the rhizosphere of tomato (Solanum lycopersicum). J Microbiol. 2020 Oct;58(10):83
[26]Endo A, Tanno H, Kadowaki R, Fujii T, Tochio T. Extracellular fructooligosaccharide degradation in Anaerostipes hadrus for co-metabolism with non-fructooligosaccharide utilizers. Biochem Biophys Res Commun. 2022 Jul 12;613:81-86.
[27]Tomazetto G, Hahnke S, Koeck DE, Wibberg D, Maus I, Pühler A, Klocke M, Schlüter A. Complete genome analysis of Clostridium bornimense strain M2/40(T): A new acidogenic Clostridium species isolated from a mesophilic two-phase laboratory-scale biogas reactor. J Biotechnol. 2016 Aug 20;232:38-49.
[28]Han KI, Kim JS, Eom MK, Lee KC, Suh MK, Kim HS, Park SH, Lee JH, Kang SW, Park JE, Oh BS, Ryu SW, Yu SY, Choi SH, Lee DH, Yoon H, Kim BY, Lee JH, Lee JS. Collinsella acetigenes sp. nov., an Anaerobic Actinobacterium Isolated from Human Feces, and Emended Description of the Genus Collinsella and Collinsella aerofaciens. Curr Microbiol. 2021 Oct;78(10):3667-3673.
[29]Yoshikawa S, Araoka R, Kajihara Y, Ito T, Miyamoto H, Kodama H. Valerate production by Megasphaera elsdenii isolated from pig feces. J Biosci Bioeng. 2018 May;125(5):519-524.

[30]Kumari M, Singh P, Nataraj BH, Kokkiligadda A, Naithani H, Azmal Ali S, Behare PV, Nagpal R. Fostering next-generation probiotics in human gut by targeted dietary modulation: An emerging perspective. Food Res Int. 2021 Dec;150(Pt A):110716.

[31]Abuqwider J, Altamimi M, Mauriello G. Limosilactobacillus reuteri in Health and Disease. Microorganisms. 2022 Feb 28;10(3):522.

[32]Kushkevych I, Dordević D, Vítězová M. Toxicity of hydrogen sulfide toward sulfate-reducing bacteria Desulfovibrio piger Vib-7. Arch Microbiol. 2019 Apr;201(3):389-397.

[33]O'Flynn C, Deusch O, Darling AE, Eisen JA, Wallis C, Davis IJ, Harris SJ. O'Flynn C, Deusch O, Darling AE, Eisen JA, Wallis C, Davis IJ, Harris SJ. Comparative Genomics of the Genus Porphyromonas Identifies Adaptations for Heme Synthesis within the Prevalent Canine Oral Species Porphyromonas cangingivalis. Genome Biol Evol. 2015 Nov 13;7(12):3397-413.

[34]Faucher SP, Matthews S, Nickzad A, Vounba P, Shetty D, Bédard É, Prévost M, Déziel E, Paranjape K. Toxoflavin secreted by Pseudomonas alcaliphila inhibits the growth of Legionella pneumophila and Vermamoeba vermiformis. Water Res. 2022 Jun 1;216:118328.

[35]Guan L, Wang K, Gao Y, Li J, Yan S, Ji N, Ren C, Wang J, Zhou Y, Li B, Lu S. Biochemical and Structural Characterization of a Novel Bacterial Tannase From Lachnospiraceae bacterium in Ruminant Gastrointestinal Tract. Front Bioeng Biotechnol. 2021 Dec 15;9:806788.

Supplementary Table 2 Faecal metabolite identification and annotation profiles in RMs

Compound_ID	Name	Formula	Molecular Weight	RT [min]	Kegg_ID	HMDB_ID	m/z
Com_33635_pos	Paliperidone	C23 H27 F N4 O3	426.20101	7.547	cpd:C21516	HMDB0015396	427.20828
Com_1013_pos	3-Methylcrotonylglycine	C7 H11 N O3	157.07413	5.413	cpd:C20828	HMDB0000459	158.08127
Com_17188_neg	N7-Methylguanosine	C11 H17 N5 O5	299.12239	1.279	cpd:C20674	--	298.11517
Com_3753_neg	4-Hydroxycoumarin	C9 H6 O3	162.03218	6.523	cpd:C20414	HMDB0003654	161.02486
Com_6419_pos	Andrographolide	C20 H30 O5	350.20756	11.564	cpd:C20214	--	351.21463
Com_22436_neg	Salvinorin A	C23 H28 O8	432.18267	10.077	cpd:C20196	--	431.17557
Com_66252_pos	Strophanthidin	C23 H32 O6	404.21925	12.913	cpd:C19988	--	405.22717
Com_5320_pos	Oleandrin	C32 H48 O9	576.32873	11.287	cpd:C19987	--	577.336
Com_61672_pos	Debromohymenialdisine	C11 H11 N5 O2	245.08975	1.702	cpd:C19897	--	246.09703
Com_1_pos	Oleamide	C18 H35 N O	281.27188	15.012	cpd:C19670	HMDB0002117	282.27914
Com_19394_pos	3-Succinoylpyridine	C9 H9 N O3	197.06888	7.282	cpd:C19569	HMDB0000992	198.07596
Com_12317_pos	8-Hydroxyquinoline	C9 H7 N O	145.05324	7.777	cpd:C19434	--	146.06062
Com_1118_pos	Fumonisin B2	C34 H59 N O14	705.39393	12.094	cpd:C19242	HMDB0034703	706.401
Com_44952_pos	Fumonisin B1	C34 H59 N O15	721.38854	11.977	cpd:C19241	HMDB0034702	722.39581
Com_24_pos	Fenpropimorph	C20 H33 N O	303.25395	15.042	cpd:C18787	HMDB0037270	304.26123
Com_54437_neg	3-Hydroxypicolinic acid	C6 H5 N O3	139.02732	1.205	cpd:C18620	--	138.01996
Com_105102_pos	Colchicoside	C27 H33 N O11	569.18999	9.368	cpd:C17963	--	570.19727
Com_2216_pos	Nootkatone	C15 H22 O	218.1673	13.394	cpd:C17914	--	219.17432
Com_2050_neg	β-Muricholic acid	C24 H40 O5	408.28826	12.345	cpd:C17726	--	407.28091
Com_670_neg	Undecanoic acid	C11 H22 O2	186.16239	13.095	cpd:C17715	HMDB0000947	185.15508
Com_507_neg	Heptanoic acid	C7 H14 O2	130.09973	10.026	cpd:C17714	HMDB0000666	129.09233
Com_550_pos	Catechin	C15 H14 O6	290.0793	6.969	cpd:C17590	HMDB0002780	291.0863
Com_96159_pos	Icariin	C33 H40 O15	698.21636	1.248	cpd:C17555	--	699.22314

Continuation of Supplementary Table 2

Compound_ID	Name	Formula	Molecular Weight	RT [min]	Kegg_ID	HMDB_ID	m/z
Com_34016_neg	albiflorin	C23 H28 O11	526.16499	12.663	cpd:C17457	--	525.15771
Com_6733_pos	Shikonin	C16 H16 O5	288.09996	9.347	cpd:C17412	--	289.10733
Com_28817_pos	Neosaxitoxin	C10 H17 N7 O5	337.1159	10.991	cpd:C17208	HMDB0029369	338.12335
Com_746_pos	Oleanolic acid	C30 H48 O3	438.35054	14.464	cpd:C17148	HMDB0002364	439.35773
Com_40092_neg	Kaempferitrin	C27 H30 O14	624.16979	7.993	cpd:C16981	HMDB0037438	623.16241
Com_4828_neg	Tauroursodeoxycholic acid	C26 H45 N O6 S	499.29806	12.073	cpd:C16868	HMDB0000874	498.29065
Com_10498_pos	Alternariol	C14 H10 O5	258.05265	7.174	cpd:C16838	HMDB0030831	259.06
Com_4251_neg	Citrinin	C13 H14 O5	250.08455	6.975	cpd:C16765	HMDB0041857	249.07745
Com_32288_pos	Aflatoxin M1	C17 H12 O7	328.05809	9.595	cpd:C16756	HMDB0030479	329.06543
Com_15527_neg	Aflatoxin G1	C17 H12 O7	328.05911	7.9	cpd:C16755	HMDB0030474	327.05182
Com_4153_neg	Aflatoxin G2	C17 H14 O7	330.07462	10.301	cpd:C16754	HMDB0030475	329.06741
Com_14453_neg	L-Hydroxylysine	C6 H14 N2 O3	162.1009	1.383	cpd:C16741	--	161.09363
Com_6061_pos	3,4-Dihydroxybenzaldehyde	C7 H6 O3	138.03202	6.968	cpd:C16700	--	139.03923
Com_112_neg	4-Oxoretinol	C20 H28 O2	300.21009	13.738	cpd:C16683	HMDB0012329	299.20264
Com_80357_pos	Morphine-3-glucuronide	C23 H27 N O9	461.16906	7.58	cpd:C16643	HMDB0041936	462.17636
Com_21919_pos	3-Hydroxylidocaine	C14 H22 N2 O2	250.16791	6.998	cpd:C16560	HMDB0060655	251.17508
Com_7_neg	Pentadecanoic acid	C15 H30 O2	242.22442	14.312	cpd:C16537	HMDB0000826	241.2169
Com_3922_neg	Nonadecanoic acid	C19 H38 O2	298.28791	15.199	cpd:C16535	HMDB0000772	297.28052
Com_4126_neg	Adrenic acid	C22 H36 O2	332.27262	14.769	cpd:C16527	HMDB0002226	331.26532
Com_3353_neg	Docosapentaenoic acid	C22 H34 O2	330.25653	14.465	cpd:C16513	HMDB0001976	329.24921
Com_9107_pos	Palmitoylethanolamide	C18 H37 N O2	299.28367	15.207	cpd:C16512	HMDB0002100	300.28937
Com_26950_pos	NNK	C14 H26 N6 O6	356.18169	8.293	cpd:C16453	HMDB0011603	357.18906
Com_53323_pos	Valine	C5 H11 N O2	117.07942	9.905	cpd:C16436	HMDB0000883	118.08666

Continuation of Supplementary Table 2

Compound_ID	Name	Formula	Molecular Weight	RT [min]	Kegg_ID	HMDB_ID	m/z
Com_31584_neg	Xanthohumol	C21 H22 O5	354.14715	12.763	cpd:C16417	HMDB0037479	353.1398
Com_26645_neg	1,3,7-Trimethyluric acid	C8 H10 N4 O3	210.07583	6.338	cpd:C16361	HMDB0002123	209.06848
Com_2930_neg	7-Methylxanthine	C6 H6 N4 O2	166.04957	2.848	cpd:C16353	HMDB0001991	165.0423
Com_1560_pos	9(S)-HOTrE	C18 H30 O3	316.204	13.636	cpd:C16326	--	317.21155
Com_1886_pos	13(S)-HOTrE	C18 H30 O3	294.21984	13.247	cpd:C16316	--	295.22723
Com_4298_pos	Traumatic acid	C12 H20 O4	228.13642	11.807	cpd:C16308	HMDB0000933	229.14349
Com_28887_neg	D-Glucuronic acid	C6 H10 O7	194.04213	15.067	cpd:C16245	HMDB0000127	193.03485
Com_17997_pos	Glycitin	C22 H22 O10	468.10669	10.635	cpd:C16195	HMDB0002219	469.11414
Com_44921_pos	Angiotensin IV	C40 H54 N8 O8	774.39653	13.281	cpd:C15849	HMDB0001038	775.40381
Com_3195_pos	Hydroquinone	C6 H6 O2	110.03708	14.003	cpd:C15603	HMDB0002434	111.04436
Com_20725_neg	Purine	C5 H4 N4	120.04398	1.356	cpd:C15587	HMDB0001366	285.08612
Com_12930_neg	Catechol	C6 H6 O2	110.03708	6.934	cpd:C15571	HMDB0000957	109.02981
Com_4210_pos	N-Benzylformamide	C8 H9 N O	135.06877	2.376	cpd:C15561	--	136.07603
Com_2403_neg	Glycolithocholic acid	C26 H43 N O4	433.31976	13.406	cpd:C15557	HMDB0000698	432.31274
Com_84203_pos	16(R)-HETE	C20 H32 O3	342.2153	8.205	cpd:C14778	HMDB0004680	343.2225
Com_3654_pos	9-KODE	C18 H30 O3	294.21944	13.625	cpd:C14766	HMDB0004669	295.22672
Com_42123_pos	13-OxoODE	C18 H30 O3	316.20393	15.945	cpd:C14765	HMDB0004668	317.21112
Com_2942_pos	Isophorone	C9 H14 O	138.1048	11.015	cpd:C14743	HMDB0031195	139.11208
Com_10555_neg	5-OxoETE	C20 H30 O3	272.21447	12.523	cpd:C14732	HMDB0010217	271.20721
Com_3775_neg	Glycitein	C16 H12 O5	284.06906	12.274	cpd:C14536	HMDB0005781	283.06195
Com_14012_neg	Kojic acid	C6 H6 O4	142.02707	1.189	cpd:C14516	HMDB0032923	141.01999
Com_13090_neg	4-Hydroxybenzophenone	C13 H10 O2	198.06841	9.64	cpd:C14230	--	197.06111
Com_3006_neg	Sulfoacetic acid	C2 H4 O5 S	139.97834	1.15	cpd:C14179	--	138.97104

Continuation of Supplementary Table 2

Compound_ID	Name	Formula	Molecular Weight	RT [min]	Kegg_ID	HMDB_ID	m/z
Com_1648_neg	delta-Tocopherol	C27 H46 O2	402.35114	14.205	cpd:C14151	HMDB0002902	401.34311
Com_3887_neg	Equol	C15 H14 O3	242.09472	10.753	cpd:C14131	HMDB0141948	241.08749
Com_10391_neg	1-Naphthoic acid	C11 H8 O2	172.05293	5.257	cpd:C14091	--	171.04564
Com_70031_pos	Mevastatin	C23 H34 O5	390.24023	11.556	cpd:C13963	--	391.24796
Com_53_pos	Stearamide	C18 H37 N O	266.26122	15.385	cpd:C13846	HMDB0034146	267.26804
Com_9560_neg	Prostaglandin D3	C20 H30 O5	332.19955	10.833	cpd:C13802	HMDB0003034	331.19229
Com_15025_neg	4-Ethylphenol	C8 H10 O	122.0736	7.239	cpd:C13637	HMDB0029306	121.06636
Com_14317_neg	Bisphenol A	C15 H16 O2	228.11558	8.106	cpd:C13624	HMDB0032133	227.10829
Com_66155_pos	Estradiol Benzoate	C25 H28 O3	376.20388	11.88	cpd:C13444	--	377.21075
Com_3235_neg	Dehydrocholic acid	C24 H34 O5	402.24124	12.974	cpd:C13154	--	401.23395
Com_30817_neg	Cyclic ADP-ribose	C15 H21 N5 O13 P2	541.06054	1.12	cpd:C13050	--	540.05347
Com_85441_pos	Pantethine	C22 H42 N4 O8 S2	554.24555	9.102	cpd:C12661	HMDB0003828	555.25226
Com_8217_pos	Trifolin	C21 H20 O11	448.1012	9.758	cpd:C12626	HMDB0030864	449.10828
Com_45182_pos	Aripiprazole	C23 H27 Cl2 N3 O2	447.15294	7.157	cpd:C12564	HMDB0005042	448.16022
Com_3867_pos	Ecgonine methyl ester	C10 H17 N O3	199.12115	1.966	cpd:C12448	HMDB0006406	200.12854
Com_979_neg	Saccharin	C7 H5 N O3 S	182.99936	5.57	cpd:C12283	HMDB0029723	181.99202
Com_7896_pos	Kanosamine	C6 H13 N O5	161.06899	1.369	cpd:C12212	--	162.07622
Com_10199_pos	(-)-Epigallocatechin	C15 H14 O7	306.07423	5.481	cpd:C12136	HMDB0038361	307.0816
Com_10910_neg	Perillic acid	C10 H14 O2	166.09979	10.811	cpd:C11924	HMDB0004586	165.09251
Com_35496_neg	Gibberellin A7	C19 H22 O5	330.14748	9.084	cpd:C11867	--	329.14023
Com_7446_neg	Gibberellin A4	C19 H24 O5	332.16308	6.671	cpd:C11864	--	331.15579

Continuation of Supplementary Table 2

Compound_ID	Name	Formula	Molecular Weight	RT [min]	Kegg_ID	HMDB_ID	m/z
Com_4405_pos	Normorphine	C16 H17 N O3	271.12117	7.483	cpd:C11785	HMDB0041959	272.12881
Com_95087_pos	6-Acetylmorphine	C19 H21 N O4	327.14714	5.982	cpd:C11781	HMDB0041812	328.15442
Com_18496_pos	Levalbuterol	C13 H21 N O3	221.14167	6.645	cpd:C11770	--	222.14888
Com_106671_pos	Mupirocin	C26 H44 O9	522.28374	6.177	cpd:C11758	HMDB0014554	523.29102
Com_28572_pos	N-Ethylglycine	C4 H9 N O2	103.06362	8.32	cpd:C11735	HMDB0041945	104.0709
Com_19239_pos	1-Naphthol	C10 H8 O	144.05775	8.767	cpd:C11714	HMDB0012138	145.06499
Com_11388_pos	2-Naphthol	C10 H8 O	144.05771	8.592	cpd:C11713	HMDB0012322	145.06499
Com_25079_pos	Methyl jasmonate	C13 H20 O3	224.14152	10.874	cpd:C11512	HMDB0036583	225.14883
Com_25277_pos	Carvone	C10 H14 O	150.1047	8.785	cpd:C11383	HMDB0035824	151.11189
Com_59996_pos	Apocynin	C9 H10 O3	166.06332	8.931	cpd:C11380	--	167.07065
Com_50488_pos	Indole-3-butyric acid	C12 H13 N O2	203.09457	7.957	cpd:C11284	HMDB0002096	204.10197
Com_52927_neg	Androsterone glucuronide	C25 H38 O8	466.25806	8.381	cpd:C11135	HMDB0002829	465.25079
Com_41876_pos	Testosterone glucuronide	C25 H36 O8	464.24227	10.516	cpd:C11134	HMDB0003193	465.2496
Com_64082_pos	Diphenylamine	C12 H11 N	186.11565	10.194	cpd:C11016	HMDB0032562	187.12254
Com_44471_pos	2,6-Xylidine	C8 H11 N	121.08954	6.949	cpd:C11004	HMDB0060677	122.09692
Com_94167_pos	Carbendazim	C9 H9 N3 O2	191.06922	7.172	cpd:C10897	HMDB0031769	192.07649
Com_2928_pos	Ecgonine	C9 H15 N O3	185.10533	1.373	cpd:C10858	HMDB0006548	186.11281
Com_79217_pos	Benzoylecgonine	C16 H19 N O4	289.13405	4.3	cpd:C10847	HMDB0041836	290.14133
Com_48208_neg	Syringic acid	C9 H10 O5	198.05317	6.882	cpd:C10833	HMDB0002085	197.04588
Com_28987_pos	Veratramine	C27 H39 N O2	409.29144	13.548	cpd:C10829	--	410.29849
Com_71705_pos	Tomatidine	C27 H45 N O2	415.34511	11.808	cpd:C10826	HMDB0034731	416.35239
Com_12737_pos	Solanine	C45 H73 N O15	867.49907	10.239	cpd:C10820	HMDB0034202	868.50635
Com_23177_pos	Jervine	C27 H39 N O3	425.29092	15.261	cpd:C10811	--	426.29803

Continuation of Supplementary Table 2

Compound_ID	Name	Formula	Molecular Weight	RT [min]	Kegg_ID	HMDB_ID	m/z
Com_36447_pos	Cannabidiolic acid	C22 H30 O4	358.21204	13.506	cpd:C10784	--	359.21927
Com_1194_pos	Sparteine	C15 H26 N2	234.20978	5.697	cpd:C10783	--	235.217
Com_1409_pos	Cytisine	C11 H14 N2 O	190.1108	4.407	cpd:C10763	--	191.11801
Com_2715_neg	Anacardic acid	C22 H36 O3	348.26691	14.768	cpd:C10759	HMDB0029683	347.25958
Com_48045_pos	Oxymatrine	C15 H24 N2 O2	264.18337	3.003	cpd:C10749	--	265.19064
Com_6814_pos	Rottlerin	C30 H28 O8	516.17961	12.861	cpd:C10721	--	517.18695
Com_26705_pos	Magnolol	C18 H18 O2	266.13082	13.714	cpd:C10651	--	267.13794
Com_76174_pos	4-Methoxycinnamaldehyde	C10 H10 O2	162.0684	6.911	cpd:C10475	HMDB0032611	163.07559
Com_4943_neg	Isoferulic acid	C10 H10 O4	194.05842	5.29	cpd:C10470	HMDB0000955	193.05118
Com_4137_pos	6-Gingerol	C17 H26 O4	294.18355	12.444	cpd:C10462	HMDB0005783	295.19092
Com_12220_neg	3,4-Dihydroxyphenylpropionic acid	C9 H10 O4	182.0584	4.21	cpd:C10447	HMDB0000423	181.05109
Com_4523_pos	β-Asarone	C12 H16 O3	208.1103	13.225	cpd:C10430	--	209.11758
Com_36947_pos	trans-Anethole	C10 H12 O	148.08933	8.432	cpd:C10428	HMDB0030837	149.09622
Com_54633_pos	β-Lapachone	C15 H14 O3	242.09419	11.32	cpd:C10367	--	243.10138
Com_64272_pos	Retrorsine	C18 H25 N O6	351.16768	6.896	cpd:C10364	--	352.17508
Com_90374_pos	Monocrotaline	C16 H23 N O6	325.15288	6.348	cpd:C10350	HMDB0034363	326.16016
Com_24545_pos	Emodin	C15 H10 O5	270.05288	9.752	cpd:C10343	HMDB0035214	271.06009
Com_6372_pos	Daidzein	C15 H10 O4	254.05818	10.379	cpd:C10208	HMDB0003312	255.06548
Com_2513_neg	Picolinic acid	C6 H5 N O2	123.03241	1.787	cpd:C10164	HMDB0002243	122.02511
Com_40409_pos	Arecoline	C8 H13 N O2	155.09519	8.343	cpd:C10129	HMDB0030353	156.10246
Com_6510_pos	Nobiletin	C21 H22 O8	402.13168	12.158	cpd:C10112	HMDB0002927	403.13889
Com_47578_pos	Myricetin	C15 H10 O8	318.03766	8.767	cpd:C10107	HMDB0002755	319.04504

Continuation of Supplementary Table 2

Compound_ID	Name	Formula	Molecular Weight	RT [min]	Kegg_ID	HMDB_ID	m/z
Com_70470_pos	Morin	C15 H10 O7	302.04311	10.146	cpd:C10105	HMDB0030796	303.05054
Com_37192_neg	Isorhamnetin	C16 H12 O7	316.05909	6.462	cpd:C10084	HMDB0002655	315.05179
Com_44103_pos	Galangin	C15 H10 O5	232.09364	8.712	cpd:C10044	HMDB0029521	271.05682
Com_12218_pos	Diosmetin	C16 H12 O6	300.06337	9.817	cpd:C10038	HMDB0029676	301.07065
Com_21608_pos	Eucalyptol	C10 H18 O	154.13606	12.477	cpd:C09844	HMDB0004472	155.14325
Com_6246_pos	Pinocembrin	C15 H12 O4	256.07365	12.9	cpd:C09827	HMDB0030808	257.08127
Com_7476_pos	Cyclohexanecarboxylic acid	C7 H12 O2	128.08412	8.196	cpd:C09822	HMDB0031342	129.09131
Com_9475_pos	Benzamide	C7 H7 N O	121.05313	8.201	cpd:C09815	HMDB0004461	122.06035
Com_13433_pos	Phellamurin	C26 H30 O11	518.17335	2.459	cpd:C09808	HMDB0031876	519.18073
Com_108_neg	Neohesperidin	C28 H34 O15	610.18953	9.526	cpd:C09806	HMDB0030748	609.18176
Com_4002_pos	Naringin	C27 H32 O14	580.17999	9.311	cpd:C09789	HMDB0002927	581.18738
Com_11955_neg	Genipin	C11 H14 O5	226.08452	12.532	cpd:C09780	HMDB0035830	225.07732
Com_20241_neg	Liquiritigenin	C15 H12 O4	256.07409	9.919	cpd:C09762	--	255.06665
Com_240_pos	Hesperidin	C28 H34 O15	610.18999	9.536	cpd:C09755	HMDB0003265	611.19702
Com_9232_pos	Deoxynivalenol	C15 H20 O6	296.12651	10.057	cpd:C09747	HMDB0036156	297.1337
Com_18360_pos	T-2 Toxin	C24 H34 O9	488.20281	8.522	cpd:C09738	HMDB0036600	489.21027
Com_240_neg	alpha-Farnesene	C15 H24	204.1883	13.642	cpd:C09665	HMDB0036065	249.18649
Com_21409_pos	Diacetoxyscirpenol	C19 H26 O7	383.19438	8.324	cpd:C09662	HMDB0035104	384.20169
Com_1732_pos	Salsolinol	C10 H13 N O2	179.0948	2.266	cpd:C09642	HMDB0005199	180.10207
Com_61545_pos	Artemisinin	C15 H22 O5	564.29242	7.03	cpd:C09538	--	283.15332
Com_20950_pos	Picrotoxinin	C15 H16 O6	292.0947	9.964	cpd:C09529	--	293.10226
Com_50498_neg	Picrotin	C15 H18 O7	310.10905	2.68	cpd:C09528	--	309.10178
Com_10033_pos	Glaucine	C21 H25 N O4	355.17918	8.094	cpd:C09446	--	356.18677

Continuation of Supplementary Table 2

Compound_ID	Name	Formula	Molecular Weight	RT [min]	Kegg_ID	HMDB_ID	m/z
Com_52528_neg	Cynaropicrin	C19 H22 O6	392.14833	2.7	cpd:C09385	HMDB0036423	391.14105
Com_24739_neg	Rutarin	C20 H24 O10	470.1433	10.876	cpd:C09309	HMDB0030884	469.13629
Com_28451_pos	Rutamarin	C21 H24 O5	356.16221	12.118	cpd:C09308	HMDB0030666	357.16953
Com_21591_pos	Psoralen	C11 H6 O3	186.03185	12.913	cpd:C09305	HMDB0034272	187.03922
Com_47693_neg	Esculin	C15 H16 O9	340.08061	5.574	cpd:C09264	HMDB0030820	339.07333
Com_55517_pos	Esculetin	C9 H6 O4	178.02684	7.478	cpd:C09263	HMDB0030819	179.03416
Com_9677_pos	Mitragynine	C23 H30 N2 O4	398.21886	11.425	cpd:C09226	--	399.22617
Com_51026_pos	Gelsemine	C20 H22 N2 O2	322.16777	8.603	cpd:C09207	--	323.17508
Com_65921_pos	Triptolide	C20 H24 O6	360.15668	10.076	cpd:C09204	HMDB0038459	361.16342
Com_12431_pos	Resiniferatoxin	C37 H40 O9	628.27161	11.539	cpd:C09179	--	629.27881
Com_92691_pos	Pleuromutilin	C22 H34 O5	416.19438	8.508	cpd:C09169	--	417.20166
Com_56849_pos	Prunin	C21 H22 O10	434.12124	8.224	cpd:C09099	--	435.12857
Com_7861_pos	Cafestol	C20 H28 O3	316.20369	14.257	cpd:C09066	HMDB0035710	317.21097
Com_23313_pos	Aloesin	C19 H22 O9	376.11535	6.892	cpd:C08994	--	377.12268
Com_242_neg	Ursolic acid	C30 H48 O3	456.36097	14.605	cpd:C08988	HMDB0002395	455.35376
Com_448_pos	Diosgenin	C27 H42 O3	414.3141	15.344	cpd:C08898	--	415.32123
Com_45487_neg	Cymarin	C30 H44 O9	548.30206	10.425	cpd:C08859	--	547.2948
Com_12793_pos	Keracyanin	C27 H30 O15	594.16169	14.441	cpd:C08620	HMDB0031458	595.16852
Com_1407_pos	Betulin	C30 H50 O2	442.38178	14.928	cpd:C08618	HMDB0036838	443.38919
Com_5752_pos	Jasmonic acid	C12 H18 O3	210.1259	9.067	cpd:C08491	HMDB0032797	211.13329
Com_21339_pos	Jasmone	C11 H16 O	164.12046	10.764	cpd:C08490	HMDB0035601	165.12775
Com_31222_neg	Triacanthine	C10 H13 N5	203.11752	10.628	cpd:C08435	--	202.11023
Com_24832_pos	N6-Methyladenine	C6 H7 N5	149.07052	2.112	cpd:C08434	HMDB0002099	150.07791

Continuation of Supplementary Table 2

Compound_ID	Name	Formula	Molecular Weight	RT [min]	Kegg_ID	HMDB_ID	m/z
Com_1153_pos	Cordycepin	C10 H13 N5 O3	251.10208	3.919	cpd:C08431	--	252.10939
Com_11850_pos	Palmitoleic Acid	C16 H30 O2	276.20953	10.892	cpd:C08362	HMDB0012328	277.21686
Com_496_neg	β-D-Glucopyranuronic acid	C6 H10 O7	194.04303	1.213	cpd:C08350	--	193.03572
Com_28240_pos	Linustatin	C16 H27 N O11	431.13741	6.577	cpd:C08333	--	432.14468
Com_11838_neg	Nervonic acid	C24 H46 O2	366.3505	16.012	cpd:C08323	HMDB0002368	365.34332
Com_4182_neg	Lignoceric Acid	C24 H48 O2	368.36616	16.386	cpd:C08320	HMDB0002003	367.35898
Com_425_neg	12-Hydroxydodecanoic acid	C12 H24 O3	170.16732	11.25	cpd:C08317	HMDB0002059	169.16031
Com_3045_neg	Erucic acid	C22 H42 O2	338.31941	15.551	cpd:C08316	HMDB0002068	337.31198
Com_1677_pos	Skatole	C9 H9 N	131.07379	11.533	cpd:C08313	HMDB0000466	132.08096
Com_39866_pos	3-Methylindole	C9 H9 N	131.07394	4.457	cpd:C08313	HMDB0000466	132.08105
Com_4889_pos	Bufotenin	C12 H16 N2 O	204.12657	4.901	cpd:C08299	HMDB0041842	205.13379
Com_19264_pos	Hypoglycin A	C7 H11 N O2	124.05298	6.379	cpd:C08287	HMDB0029427	125.06018
Com_1895_neg	Docosanoic acid	C22 H44 O2	340.33491	15.905	cpd:C08281	HMDB0000944	339.32764
Com_1079_pos	Tiglic acid	C5 H8 O2	100.05265	1.404	cpd:C08279	HMDB0001470	101.05991
Com_2628_pos	Octanedioic acid	C8 H14 O4	174.08966	8.21	cpd:C08278	HMDB0000893	175.09657
Com_2179_neg	Suberic acid	C8 H14 O4	174.08957	1.76	cpd:C08278	HMDB0000893	173.08218
Com_66824_pos	Sebacic acid	C10 H18 O4	202.12067	7.149	cpd:C08277	HMDB0000792	203.12817
Com_25980_neg	Kinetin	C10 H9 N5 O	215.07985	1.4	cpd:C08272	HMDB0012245	214.07278
Com_1788_neg	Azelaic acid	C9 H16 O4	188.10524	4.381	cpd:C08261	HMDB0000784	187.09795
Com_6793_neg	Fludrocortisone acetate	C23 H31 F O6	422.21625	9.147	cpd:C08186	HMDB0002802	421.20901
Com_25782_pos	Prednisolone tebutate	C27 H38 O6	458.26688	10.774	cpd:C08182	HMDB0014998	459.2742
Com_17243_pos	Hydroxyprogesterone caproate	C27 H40 O4	428.29344	10.525	cpd:C08148	HMDB0001830	429.30109
Com_66195_pos	Oxymorphone	C17 H19 N O4	284.10498	11.444	cpd:C08019	HMDB0015323	285.11285

Continuation of Supplementary Table 2

Compound_ID	Name	Formula	Molecular Weight	RT [min]	Kegg_ID	HMDB_ID	m/z
Com_7_pos	Ursodeoxycholic acid	C24 H40 O4	392.29261	13.965	cpd:C07880	HMDB0000946	393.29846
Com_38859_pos	Naratriptan	C17 H25 N3 O2 S	297.20322	8.744	cpd:C07792	--	336.16638
Com_1249_neg	Mestranol	C21 H26 O2	310.18198	12.2	cpd:C07618	HMDB0015446	309.17465
Com_20946_neg	Rotenone	C23 H22 O6	394.14252	10.457	cpd:C07593	HMDB0034436	393.13519
Com_61364_pos	Colchicine	C22 H25 N O6	399.16868	9.801	cpd:C07592	--	400.17587
Com_54727_neg	Psilocybin	C12 H17 N2 O4 P	284.0901	1.212	cpd:C07576	--	283.08221
Com_1917_pos	Acetanilide	C8 H9 N O	135.0687	10.457	cpd:C07565	HMDB0001250	136.07613
Com_15932_pos	17alpha-Ethinyl estradiol	C20 H24 O2	296.17778	12.308	cpd:C07534	HMDB0001926	297.18509
Com_75383_pos	Amphetamine	C9 H13 N	135.10517	2.28	cpd:C07514	HMDB0014328	136.11244
Com_3_pos	Caffeine	C8 H10 N4 O2	194.0803	7.783	cpd:C07481	HMDB0001847	195.0874
Com_452_pos	Theobromine	C7 H8 N4 O2	180.06469	6.161	cpd:C07480	HMDB0002825	181.07191
Com_30377_pos	Pilocarpine	C11 H16 N2 O2	208.12111	6.829	cpd:C07474	--	209.12845
Com_37817_pos	Phenmetrazine	C11 H15 N O	177.11547	5.757	cpd:C07432	HMDB0014968	178.12263
Com_12996_neg	1,5-Anhydro-D-glucitol	C6 H12 O5	224.0902	1.244	cpd:C07326	HMDB0002712	223.08279
Com_53135_pos	Stanozolol	C21 H32 N2 O	328.24742	5.631	cpd:C07311	HMDB0003116	329.25497
Com_95976_pos	Mezlocillin	C21 H25 N5 O8 S2	267.0801	1.381	cpd:C07221	--	557.14941
Com_5329_neg	o-Toluic Acid	C8 H8 O2	136.05278	1.805	cpd:C07215	--	135.04544
Com_18059_pos	Methyltestosterone	C20 H30 O2	302.22447	15.446	cpd:C07198	HMDB0015655	303.23236
Com_14543_pos	Methyldopa	C10 H13 N O4	211.08475	1.759	cpd:C07194	HMDB0011754	212.09203
Com_5398_neg	Valproic acid	C8 H16 O2	144.11537	4.682	cpd:C07185	HMDB0000901	143.10811
Com_7007_pos	Theophylline	C7 H8 N4 O2	180.06473	7.027	cpd:C07130	HMDB0001889	181.07191
Com_9010_pos	Medroxyprogesterone	C22 H32 O3	344.23508	13.822	cpd:C07119	HMDB0001939	345.24265
Com_19561_pos	Acetophenone	C8 H8 O	120.05794	9.869	cpd:C07113	HMDB0033910	121.06526

Continuation of Supplementary Table 2

Compound_ID	Name	Formula	Molecular Weight	RT [min]	Kegg_ID	HMDB_ID	m/z
Com_1570_pos	Styrene	C8 H8	104.06293	1.989	cpd:C07083	HMDB0034240	105.07005
Com_9228_pos	Isoproterenol	C11 H17 N O3	211.12127	1.408	cpd:C07056	--	212.12802
Com_1094_neg	THC	C21 H30 O2	314.22531	14.7	cpd:C06972	--	313.2179
Com_12672_pos	dihydrotachysterol	C28 H46 O	398.35556	16.009	cpd:C06957	HMDB0015203	399.36292
Com_75943_pos	Capsaicin	C18 H27 N O3	305.1991	8.899	cpd:C06866	HMDB0002227	306.2063
Com_433_neg	Dithranol	C14 H10 O3	226.06327	7.671	cpd:C06831	--	225.05606
Com_34863_neg	Acetylcysteine	C5 H9 N O3 S	163.03081	12.427	cpd:C06809	HMDB0000574	162.02353
Com_17612_neg	Paracetamol	C8 H9 N O2	151.06376	6.738	cpd:C06804	HMDB0001859	150.05649
Com_17178_pos	Aflatoxin B1	C17 H12 O6	312.06301	11.344	cpd:C06800	HMDB0006552	313.07053
Com_1589_neg	4-Methylcatechol	C7 H8 O2	124.05283	8.761	cpd:C06730	HMDB0000873	123.04549
Com_23672_neg	Monensin	C36 H62 O11	670.42955	11.565	cpd:C06693	--	669.42163
Com_26908_neg	Vanillic acid	C8 H8 O4	168.04273	1.172	cpd:C06672	HMDB0000484	167.03545
Com_8881_neg	Oxytetracycline	C22 H24 N2 O9	460.14149	8.628	cpd:C06624	HMDB0014733	459.13434
Com_7936_pos	Cuminaldehyde	C10 H12 O	148.08918	12.682	cpd:C06577	HMDB0002214	149.09634
Com_11638_neg	Chlortetracycline	C22 H23 Cl N2 O8	478.11573	7.638	cpd:C06571	HMDB0014401	477.10843
Com_1148_neg	Genistein	C15 H10 O5	270.05322	13.249	cpd:C06563	HMDB0003217	269.04608
Com_16530_pos	Solanidine	C27 H43 N O	397.33464	10.227	cpd:C06543	HMDB0003236	398.34192
Com_9915_pos	Reserpine	C33 H40 N2 O9	608.26413	15.012	cpd:C06539	--	609.27051
Com_22101_neg	Harmaline	C13 H14 N2 O	214.11117	7.645	cpd:C06536	HMDB0030310	213.10394
Com_71970_pos	Papaverine	C20 H21 N O4	339.14738	7.842	cpd:C06533	HMDB0015245	340.15451
Com_7537_pos	Quinidine	C20 H24 N2 O2	324.18025	7.249	cpd:C06527	--	325.18753
Com_93550_pos	Quinine	C20 H24 N2 O2	324.18361	9.687	cpd:C06526	--	325.19089
Com_13156_neg	Prostaglandin F3α	C20 H32 O5	398.23163	11.397	cpd:C06476	--	397.2243

Continuation of Supplementary Table 2

Compound_ID	Name	Formula	Molecular Weight	RT [min]	Kegg_ID	HMDB_ID	m/z
Com_20962_neg	Prostaglandin F1α	C20 H36 O5	356.25706	10.262	cpd:C06475	--	355.24988
Com_4891_pos	Muramic acid	C9 H17 N O7	251.10018	1.362	cpd:C06470	HMDB0003254	252.10788
Com_84282_pos	Bialaphos	C11 H22 N3 O6 P	668.22234	7.409	cpd:C06457	--	669.22961
Com_7975_neg	Prostaglandin E3	C20 H30 O5	386.18497	10.836	cpd:C06439	HMDB0002664	385.17804
Com_2678_neg	Docosahexaenoic acid	C22 H32 O2	328.2408	14.327	cpd:C06429	HMDB0002183	327.23352
Com_466_neg	Eicosapentaenoic acid	C20 H30 O2	302.22499	14.134	cpd:C06428	HMDB0001999	301.21771
Com_214_neg	Arachidic acid	C20 H40 O2	312.3033	15.149	cpd:C06425	HMDB0002212	311.29578
Com_44494_pos	Myristic acid	C14 H28 O2	228.20893	12.908	cpd:C06424	HMDB0000806	229.21623
Com_865_neg	Caprylic acid	C8 H16 O2	144.11541	11.191	cpd:C06423	HMDB0000482	143.10806
Com_1618_pos	4-Quinolinecarboxylic acid	C10 H7 N O2	173.0479	8.554	cpd:C06414	--	174.05518
Com_88463_pos	Quinoline-4-carboxylic acid	C10 H7 N O2	173.04773	9.688	cpd:C06414	--	174.05501
Com_7539_pos	Quinoline	C9 H7 N	129.05822	10.39	cpd:C06413	HMDB0033731	130.06548
Com_20982_pos	Methyl cinnamate	C10 H10 O2	162.06836	10.409	cpd:C06358	HMDB0033833	325.14401
Com_630_neg	Terephthalic Acid	C8 H6 O4	166.02703	8.991	cpd:C06337	HMDB0002428	165.01965
Com_27299_pos	Isoquinoline	C9 H7 N	129.05823	13.726	cpd:C06323	HMDB0034244	130.06552
Com_17701_neg	Vanillyl alcohol	C8 H10 O3	154.06345	6.221	cpd:C06317	HMDB0032012	153.05618
Com_18696_neg	Biopterin	C9 H11 N5 O3	237.08546	1.223	cpd:C06313	HMDB0000468	236.07829
Com_40627_pos	Senecionine	C18 H25 N O5	335.17188	8.285	cpd:C06176	--	336.17862
Com_16485_neg	Adipic acid	C6 H10 O4	146.05832	1.089	cpd:C06104	HMDB0000448	145.05101
Com_36504_neg	Tyrosol	C8 H10 O2	138.06846	4.236	cpd:C06044	HMDB0004284	137.06119
Com_20750_pos	Thromboxane B2	C20 H34 O6	392.21778	11.648	cpd:C05963	HMDB0003252	393.22501
Com_3611_neg	Prostaglandin J2	C20 H30 O4	316.20437	12.51	cpd:C05957	HMDB0004238	315.19705
Com_13209_pos	Prostaglandin G2	C20 H32 O6	368.21819	12.18	cpd:C05956	HMDB0003235	369.22546

Continuation of Supplementary Table 2

Compound_ID	Name	Formula	Molecular Weight	RT [min]	Kegg_ID	HMDB_ID	m/z
Com_35016_pos	Prostaglandin B2	C20 H30 O4	334.21371	9.805	cpd:C05954	HMDB0004236	335.22092
Com_9383_pos	Prostaglandin A2	C20 H30 O4	316.20417	12.235	cpd:C05953	HMDB0002752	317.21161
Com_46567_pos	D-Panthenol	C9 H19 N O4	205.13129	1.883	cpd:C05944	HMDB0004231	206.1385
Com_57976_pos	Neopterin	C9 H11 N5 O4	253.08048	6.807	cpd:C05926	HMDB0000845	254.08824
Com_12203_pos	Kaempferol	C15 H10 O6	286.04791	9.76	cpd:C05903	HMDB0005801	287.05502
Com_21060_neg	Piceatannol	C14 H12 O4	244.07401	9.395	cpd:C05901	HMDB0004215	243.06676
Com_7274_pos	Coumarin	C9 H6 O2	146.03709	8.217	cpd:C05851	HMDB0001218	147.04427
Com_1629_pos	Methylimidazoleacetic acid	C6 H8 N2 O2	140.05882	1.357	cpd:C05828	HMDB0002820	141.06609
Com_4_pos	Stercobilin	C33 H46 N4 O6	594.34126	10.25	cpd:C05793	HMDB0004159	595.34821
Com_22645_pos	5-Methoxyindoleacetic acid	C11 H11 N O3	205.07377	8.216	cpd:C05660	HMDB0004096	206.08109
Com_13190_pos	5-Methoxytryptamine	C11 H14 N2 O	173.0842	6.947	cpd:C05659	HMDB0004095	174.0916
Com_15014_pos	6-Hydroxymelatonin	C13 H16 N2 O3	248.11651	4.828	cpd:C05643	HMDB0004081	249.12393
Com_10774_pos	Eriodictyol	C15 H12 O6	288.06354	10.161	cpd:C05631	HMDB0005810	289.07095
Com_49245_neg	Rutin	C27 H30 O16	610.15474	8.807	cpd:C05625	HMDB0003249	609.14746
Com_11006_neg	Melanin	C18 H10 N2 O4	318.06428	2.061	cpd:C05606	HMDB0004068	317.05701
Com_52478_pos	Phenylacetylglycine	C10 H11 N O3	193.07396	10.488	cpd:C05598	HMDB0000821	194.08141
Com_5639_pos	3-Methoxytyramine	C9 H13 N O2	167.09491	11.478	cpd:C05587	HMDB0000022	168.10242
Com_38287_neg	2,5-Dihydroxybenzaldehyde	C7 H6 O3	138.03221	7.809	cpd:C05585	HMDB0004062	137.02493
Com_7294_neg	Homovanillic acid	C9 H10 O4	182.05814	8.262	cpd:C05582	HMDB0000118	181.05072
Com_48633_pos	3,4-Dihydroxymandelic acid	C8 H8 O5	184.0374	7.344	cpd:C05580	HMDB0001866	185.04468
Com_20606_neg	indole-5,6-quinone	C8 H5 N O2	147.03225	7.995	cpd:C05579	HMDB0006779	146.02504
Com_56778_pos	Biocytin	C16 H28 N4 O4 S	372.18323	6.676	cpd:C05552	HMDB0003134	373.19086
Com_31397_pos	Penicillin G	C16 H18 N2 O4 S	334.09901	8.803	cpd:C05551	HMDB0037849	335.10638

Continuation of Supplementary Table 2

Compound_ID	Name	Formula	Molecular Weight	RT [min]	Kegg_ID	HMDB_ID	m/z
Com_52999_pos	Deoxyinosine	C10 H12 N4 O4	252.086	4.218	cpd:C05512	HMDB0000071	253.09315
Com_115_neg	Cortodoxone	C21 H30 O4	346.21479	13.79	cpd:C05488	HMDB0000015	345.20749
Com_21140_pos	Tetrahydrocortisone	C21 H32 O5	750.43391	15.22	cpd:C05470	HMDB0000903	751.44135
Com_998_neg	Taurochenodeoxycholic acid	C26 H45 N O6 S	499.29772	12.901	cpd:C05465	HMDB0000951	498.29028
Com_28753_pos	Taurodeoxycholic Acid	C26 H45 N O6 S	481.28748	13.064	cpd:C05463	HMDB0000896	482.29446
Com_10058_pos	Cholecalciferol	C27 H44 O	384.33916	14.72	cpd:C05443	HMDB0000876	385.3468
Com_11338_pos	Ergocalciferol	C28 H44 O	396.33952	13.864	cpd:C05441	HMDB0000900	397.34674
Com_7828_neg	D-Sedoheptulose 7-phosphate	C7 H15 O10 P	290.04073	1.179	cpd:C05382	HMDB0001068	289.03348
Com_60442_pos	Nicotinuric Acid	C8 H8 N2 O3	180.05353	6.957	cpd:C05380	HMDB0003269	181.06088
Com_29787_pos	Menadione	C11 H8 O2	172.05256	8.938	cpd:C05377	HMDB0001892	173.05971
Com_5948_pos	Phenethylamine	C8 H11 N	121.08946	6.214	cpd:C05332	HMDB0012275	122.09673
Com_29293_neg	2-Phenylethylamine	C8 H11 N	121.0895	5.844	cpd:C05332	HMDB0012275	120.08222
Com_17793_neg	2-Hydroxyestradiol	C18 H24 O3	288.17296	13.237	cpd:C05301	HMDB0000338	287.16562
Com_36714_pos	16α-Hydroxyestrone	C18 H22 O3	286.15664	11.379	cpd:C05300	--	287.16373
Com_9966_pos	2-Methoxyestrone	C19 H24 O3	300.17266	10.79	cpd:C05299	HMDB0000010	301.17996
Com_13340_pos	7α-Hydroxytestosterone	C19 H28 O3	304.20382	9.341	cpd:C05291	--	305.211
Com_28617_pos	Adrenosterone	C19 H24 O3	300.17232	13.306	cpd:C05285	HMDB0006772	301.17978
Com_81569_pos	Protopine	C20 H19 N O5	353.12973	7.237	cpd:C05189	HMDB0003920	354.13715
Com_25413_pos	Estriol	C18 H24 O3	288.17259	12.019	cpd:C05141	HMDB0000347	289.17987
Com_1442_pos	16α-Hydroxydehydroepiandrosterone	C19 H28 O3	321.23653	14.056	cpd:C05139	--	322.2438
Com_3732_neg	17α-Hydroxypregnenolone	C21 H32 O3	332.23566	13.774	cpd:C05138	--	331.2283
Com_34091_pos	N-Acetylhistamine	C7 H11 N3 O	153.0905	1.654	cpd:C05135	--	154.09778

Continuation of Supplementary Table 2

Compound_ID	Name	Formula	Molecular Weight	RT [min]	Kegg_ID	HMDB_ID	m/z
Com_5342_pos	Taurocholic acid	C26 H45 N O7 S	515.2925	13.361	cpd:C05122	HMDB0000036	516.29956
Com_4528_pos	Prostaglandin E1	C20 H34 O5	336.22994	12.288	cpd:C04741	HMDB0001442	337.23715
Com_19391_neg	N-Acetyl-α-D-glucosamine 1-phosphate	C8 H16 N O9 P	301.05687	1.187	cpd:C04501	--	300.04959
Com_3_neg	Deoxycholic acid	C24 H40 O4	392.29294	13.329	cpd:C04483	HMDB0000626	391.28555
Com_14216_pos	Etiocholanolone	C19 H30 O2	272.21382	14.043	cpd:C04373	HMDB0000490	273.22092
Com_14098_neg	Lithocholic acid	C24 H40 O3	376.29838	15.4	cpd:C03990	HMDB0000761	375.29111
Com_36160_pos	Linalool	C10 H18 O	154.13605	12.714	cpd:C03985	HMDB0036100	155.14328
Com_16467_pos	Piperine	C17 H19 N O3	285.13661	12.42	cpd:C03882	HMDB0029377	286.14389
Com_7700_pos	N6,N6,N6-Trimethyl-L-lysine	C9 H20 N2 O2	188.15262	1.395	cpd:C03793	HMDB0001325	189.1597
Com_16868_pos	5beta-Androstane-3,17-dione	C19 H28 O2	288.20899	11.031	cpd:C03772	HMDB0003769	289.21643
Com_5322_neg	Taurolithocholic acid 3-sulfate	C26 H45 N O8 S2	563.25949	11.861	cpd:C03642	HMDB0002580	562.25281
Com_49454_pos	N,N-Dimethylarginine	C8 H18 N4 O2	202.14275	1.332	cpd:C03626	HMDB0001539	203.14998
Com_72244_pos	NG,NG-Dimethyl-L-arginine	C8 H20 Cl2 N4 O2	274.09524	8.501	cpd:C03626	HMDB0001539	275.10245
Com_41579_pos	5-Methyl-2'-deoxycytidine	C10 H15 N3 O4	241.10574	1.301	cpd:C03592	HMDB0002224	259.13943
Com_9046_neg	Resveratrol	C14 H12 O3	228.07916	11.612	cpd:C03582	HMDB0003747	227.07187
Com_8164_neg	Ciliatine	C2 H8 N O3 P	125.02426	7.404	cpd:C03557	HMDB0011747	124.01696
Com_19365_neg	N-Acetyl-L-phenylalanine	C11 H13 N O3	207.08998	7.293	cpd:C03519	HMDB0000512	206.0829
Com_12202_pos	Limonin	C26 H30 O8	452.18185	12.912	cpd:C03514	HMDB0035921	453.18912
Com_90029_pos	Folinic acid	C20 H23 N7 O7	473.16674	7.116	cpd:C03479	HMDB0001562	474.17386
Com_43353_pos	L-Argininosuccinate	C10 H18 N4 O6	290.1222	1.297	cpd:C03406	HMDB0000052	291.12964
Com_7578_pos	L-Tyrosine methyl ester	C10 H13 N O3	195.08979	7.27	cpd:C03404	--	196.09711
Com_15101_pos	L-Tyrosinemethylester	C10 H13 N O3	195.0898	2.897	cpd:C03404	--	196.09718

Continuation of Supplementary Table 2

Compound_ID	Name	Formula	Molecular Weight	RT [min]	Kegg_ID	HMDB_ID	m/z
Com_19826_pos	L-threo-3-Phenylserine	C9 H11 N O3	181.07423	1.385	cpd:C03290	HMDB0002184	182.08162
Com_10855_pos	Deoxycorticosterone	C21 H30 O3	330.2194	9.971	cpd:C03205	HMDB0000016	331.22635
Com_20811_pos	Desoxycortone	C21 H30 O3	330.2198	14.195	cpd:C03205	HMDB0000016	331.22711
Com_2328_neg	Glycerol-3-phosphate	C3 H9 O6 P	172.01431	1.443	cpd:C03189	HMDB0000126	171.00713
Com_59481_pos	N-Acetyl-D-tryptophan	C13 H14 N2 O3	246.10029	9.183	cpd:C03137	HMDB0013713	247.10771
Com_4396_pos	Testosterone acetate	C21 H30 O3	330.21742	14.311	cpd:C03027	HMDB0062780	331.22461
Com_2357_pos	L-Palmitoylcarnitine	C23 H45 N O4	399.33536	14.064	cpd:C02990	HMDB0000222	400.34302
Com_54631_pos	Imidazoleacetic acid	C5 H6 N2 O2	126.04318	1.358	cpd:C02835	HMDB0002024	127.05067
Com_38303_pos	Cyclohexylsulfamate	C6 H13 N O3 S	179.06178	1.34	cpd:C02824	HMDB0031340	180.06906
Com_1592_pos	Hydrocortisone acetate	C23 H32 O6	404.21981	10.67	cpd:C02821	HMDB0000063	405.22687
Com_1629_neg	10-Hydroxydecanoic acid	C10 H20 O3	188.14171	9.672	cpd:C02774	--	187.1344
Com_9668_pos	N6-Acetyl-L-lysine	C8 H16 N2 O3	188.11617	1.382	cpd:C02727	HMDB0000206	189.12354
Com_26054_pos	N-Acetyl-L-leucine	C8 H15 N O3	173.10539	9.069	cpd:C02710	HMDB0011756	174.11276
Com_27627_pos	N-Formylkynurenine	C11 H12 N2 O4	236.07954	1.402	cpd:C02700	HMDB0001200	237.08688
Com_157_neg	Lauric acid	C12 H24 O2	200.17804	13.498	cpd:C02679	HMDB0000638	199.17075
Com_83_neg	Dodecanedioic acid	C12 H22 O4	230.15197	9.546	cpd:C02678	HMDB0000623	229.14447
Com_42806_pos	Ecdysterone	C27 H44 O7	480.30928	10.654	cpd:C02633	HMDB0030180	481.31677
Com_110925_pos	2,4-Dichlorophenol	C6 H4 Cl2 O	161.96357	0.518	cpd:C02625	HMDB0004811	184.95279
Com_24220_pos	3-O-Feruloylquinic acid	C17 H20 O9	368.10909	7.823	cpd:C02572	HMDB0030669	369.11636
Com_44439_pos	O-Acetyl-L-carnitine	C9 H17 N O4	203.11585	7.903	cpd:C02571	HMDB0000201	204.123
Com_88599_pos	N-Methylhydantoin	C4 H6 N2 O2	114.04334	1.43	cpd:C02565	HMDB0003646	267.04984
Com_18406_neg	Chenodeoxycholic Acid	C24 H40 O4	438.29939	11.867	cpd:C02528	HMDB0000518	437.29211
Com_51715_pos	2-Phenylacetamide	C8 H9 N O	135.06875	7.767	cpd:C02505	HMDB0010715	136.07599

Continuation of Supplementary Table 2

Compound_ID	Name	Formula	Molecular Weight	RT [min]	Kegg_ID	HMDB_ID	m/z
Com_1266_neg	2-Isopropylmalic acid	C7 H12 O5	176.06879	1.196	cpd:C02504	HMDB0000402	175.06143
Com_26836_pos	1-Methyladenosine	C11 H15 N5 O4	281.11257	5.719	cpd:C02494	HMDB0003331	282.12
Com_11993_pos	gamma-Tocopherol	C28 H48 O2	416.36537	15.553	cpd:C02483	HMDB0001492	417.37341
Com_20085_pos	Xanthurenic acid	C10 H7 N O4	205.03782	6.456	cpd:C02470	HMDB0000881	206.04526
Com_83331_pos	L-Homocitrulline	C7 H15 N3 O3	189.11147	1.351	cpd:C02427	HMDB0000679	417.1861
Com_1673_neg	Cinnamyl alcohol	C9 H10 O	134.0735	9.914	cpd:C02394	HMDB0029697	133.06618
Com_40620_pos	5-Methylcytosine	C5 H7 N3 O	125.05934	1.383	cpd:C02376	HMDB0002894	126.06619
Com_14475_neg	Prostaglandin F2β	C20 H34 O5	708.49676	16.258	cpd:C02314	--	707.48962
Com_62738_pos	L-Cystathionine	C7 H14 N2 O4 S	222.06831	9.727	cpd:C02291	HMDB0000099	223.07559
Com_34977_pos	D-Phenylalanine	C9 H11 N O2	165.07919	9.942	cpd:C02265	--	166.08647
Com_3654_neg	D-Galactosamine	C6 H14 Cl N O5	215.05653	1.445	cpd:C02262	--	214.0493
Com_9815_pos	7-Methylguanine	C6 H7 N5 O	165.06534	2.004	cpd:C02242	HMDB0006040	166.07253
Com_2101_neg	Citraconic acid	C5 H6 O4	130.02692	1.199	cpd:C02226	HMDB0000634	129.01962
Com_10091_neg	Protoporphyrin IX	C34 H34 N4 O4	562.26092	12.353	cpd:C02191	HMDB0000241	561.25366
Com_94470_pos	Methylmalonate	C4 H6 O4	118.02701	13.859	cpd:C02170	HMDB0000202	119.03422
Com_5985_pos	Glycyl-L-leucine	C8 H16 N2 O3	188.11646	6.422	cpd:C02155	HMDB0028929	189.1236
Com_1215_pos	Corticosterone	C21 H30 O4	346.21449	10.45	cpd:C02140	HMDB0001547	347.22183
Com_281_neg	Phenylglyoxylic acid	C8 H6 O3	150.03196	12.739	cpd:C02137	HMDB0001587	149.02463
Com_69294_pos	Spectinomycin	C14 H24 N2 O7	364.17833	9.94	cpd:C02078	--	365.18561
Com_8890_neg	Pseudouridine	C9 H12 N2 O6	244.06998	1.43	cpd:C02067	HMDB0000767	243.06255
Com_7213_pos	Phylloquinone	C31 H46 O2	450.35163	14.061	cpd:C02059	HMDB0015157	451.35837
Com_49880_pos	Maltotetraose	C24 H42 O21	688.20556	1.359	cpd:C02052	HMDB0001296	689.21307
Com_1449_pos	4-Hydroxyindole	C8 H7 N O	133.05288	7.696	cpd:C02040	--	134.0601

Continuation of Supplementary Table 2

Compound_ID	Name	Formula	Molecular Weight	RT [min]	Kegg_ID	HMDB_ID	m/z
Com_426_pos	Acetylcholine	C7 H15 N O2	145.11038	1.33	cpd:C01996	--	146.11758
Com_29603_pos	Pregnenolone	C21 H32 O2	316.24061	10.878	cpd:C01953	HMDB0000253	317.24802
Com_6092_pos	Okadaic acid	C44 H68 O13	786.45511	13.695	cpd:C01945	HMDB0030441	787.46246
Com_52719_pos	Homoarginine	C7 H16 N4 O2	188.12749	1.293	cpd:C01924	HMDB0000670	189.13509
Com_787_pos	Glycocholic acid	C26 H43 N O6	465.30974	12.78	cpd:C01921	HMDB0000138	466.3168
Com_33638_pos	Desthiobiotin	C10 H18 N2 O3	214.13157	9.065	cpd:C01909	HMDB0003581	215.13916
Com_11754_neg	Pyroglutamic acid	C5 H7 N O3	129.04299	1.333	cpd:C01879	HMDB0000805	128.03568
Com_16388_pos	L-Pyroglutamic acid	C5 H7 N O3	147.05349	8.541	cpd:C01879	HMDB0000267	148.06097
Com_2412_neg	4-Oxoproline	C5 H7 N O3	129.04299	1.204	cpd:C01877	--	128.03575
Com_18596_pos	Methoxsalen	C12 H8 O4	216.04258	11.561	cpd:C01864	HMDB0014693	217.04987
Com_110397_pos	Scopolamine	C17 H21 N O4	303.14665	5.668	cpd:C01851	HMDB0003573	304.15393
Com_21872_pos	Rosmarinic acid	C18 H16 O8	377.11089	7.541	cpd:C01850	HMDB0003572	378.11816
Com_14920_neg	Maltotriose	C18 H32 O16	504.16773	11.383	cpd:C01835	HMDB0001262	503.16037
Com_45327_neg	Aldosterone	C21 H28 O5	406.20192	9.35	cpd:C01780	HMDB0000037	405.19464
Com_6277_neg	Xanthosine	C10 H12 N4 O6	284.07622	3.279	cpd:C01762	HMDB0000299	283.06894
Com_6542_neg	Scopoletin	C10 H8 O4	192.04273	7.61	cpd:C01752	HMDB0034344	191.03557
Com_7856_neg	Resorcinol	C6 H6 O2	110.0371	5.546	cpd:C01751	--	109.02984
Com_16389_neg	3-(4-Hydroxyphenyl)propionic acid	C9 H10 O3	166.06339	3.999	cpd:C01744	HMDB0002199	165.05608
Com_7803_pos	Kynurenic acid	C10 H7 N O3	189.04292	10.113	cpd:C01717	HMDB0000715	190.05046
Com_2_neg	Elaidic acid	C18 H34 O2	282.25579	14.73	cpd:C01712	HMDB0000573	281.24847
Com_986_pos	3',5,7-Trihydroxy-4'-methoxyflavanone	C16 H14 O6	302.07898	9.534	cpd:C01709	HMDB0005782	303.08618

Continuation of Supplementary Table 2

Compound_ID	Name	Formula	Molecular Weight	RT [min]	Kegg_ID	HMDB_ID	m/z
Com_485_pos	Hesperetin	C16 H14 O6	302.07915	10.898	cpd:C01709	HMDB0005782	303.08627
Com_24509_neg	Gibberellic acid	C19 H22 O6	346.1423	6.776	cpd:C01699	HMDB0003559	345.13507
Com_808_neg	Calcitriol	C27 H44 O3	416.32975	14.164	cpd:C01673	HMDB0001903	415.32254
Com_49576_pos	Cadaverine	C5 H14 N2	102.11604	1.082	cpd:C01672	HMDB0002322	103.12325
Com_11805_pos	Taxifolin	C15 H12 O7	304.0582	8.798	cpd:C01617	--	305.06549
Com_62358_pos	Phlorizin	C21 H24 O10	458.11962	9.932	cpd:C01604	HMDB0036634	459.1275
Com_10043_neg	Ornithine	C5 H12 N2 O2	132.09031	1.298	cpd:C01602	HMDB0003374	131.08302
Com_9950_neg	Nonanoic acid	C9 H18 O2	158.13114	11.813	cpd:C01601	HMDB0000847	157.12386
Com_26407_pos	Melatonin	C13 H16 N2 O2	232.12145	5.975	cpd:C01598	HMDB0001389	233.12872
Com_15130_pos	Maleamic acid	C4 H5 N O3	115.04246	8.765	cpd:C01596	--	116.04961
Com_61_pos	Hippuric acid	C9 H9 N O3	179.05821	7.595	cpd:C01586	HMDB0000714	180.06538
Com_17098_neg	Hexanoic acid	C6 H12 O2	116.08402	1.318	cpd:C01585	HMDB0061883	115.07674
Com_1809_neg	Decanoic acid	C10 H20 O2	172.14676	12.602	cpd:C01571	HMDB0000511	171.13942
Com_20765_pos	Allantoin	C4 H6 N4 O3	158.04321	1.088	cpd:C01551	HMDB0000462	159.05055
Com_1003_neg	2-Furoic acid	C5 H4 O3	112.01648	1.099	cpd:C01546	HMDB0000617	111.0091
Com_8288_pos	o-Cresol	C7 H8 O	108.05757	12.841	cpd:C01542	HMDB0002055	109.06508
Com_25_neg	Stearic acid	C18 H36 O2	284.27164	15.015	cpd:C01530	HMDB0000827	283.26431
Com_17124_pos	Morphine	C17 H19 N O3	285.13693	7.269	cpd:C01516	HMDB0014440	286.14441
Com_3967_neg	Luteolin	C15 H10 O6	286.04835	9.941	cpd:C01514	HMDB0005800	285.04111
Com_6969_pos	Ferulic acid	C10 H10 O4	194.05805	9.033	cpd:C01494	HMDB0000954	195.0657
Com_50107_pos	Atropine	C17 H23 N O3	289.16792	6.875	cpd:C01479	HMDB0014712	290.1752
Com_3208_neg	4-Methylphenol	C7 H8 O	108.05779	2.724	cpd:C01468	HMDB0001858	107.05048
Com_24881_pos	Vitexin	C21 H20 O10	432.10567	8.929	cpd:C01460	--	433.11282

Continuation of Supplementary Table 2

Compound_ID	Name	Formula	Molecular Weight	RT [min]	Kegg_ID	HMDB_ID	m/z
Com_47337_pos	Ouabain	C29 H44 O12	622.24217	14.789	cpd:C01443	HMDB0015224	623.24945
Com_5424_neg	Gallic acid	C7 H6 O5	170.02195	1.082	cpd:C01424	HMDB0005807	169.01479
Com_34523_pos	Tacrolimus	C44 H69 N O12	785.45708	12.072	cpd:C01375	HMDB0015002	786.46503
Com_29825_neg	dUDP	C9 H14 N2 O11 P2	388.00784	1.162	cpd:C01346	HMDB0001000	387.00046
Com_19267_neg	D-myo-Inositol 1,4-bisphosphate	C6 H14 O12 P2	339.99656	1.086	cpd:C01220	HMDB0000968	338.98929
Com_35549_pos	Caffeic acid	C9 H8 O4	180.04236	8.42	cpd:C01197	HMDB0001964	181.04932
Com_34736_pos	17alpha-Hydroxyprogesterone	C21 H30 O3	330.21896	11.059	cpd:C01176	HMDB0000374	331.22577
Com_3426_neg	17α-Hydroxyprogesterone	C21 H30 O3	330.22018	13.521	cpd:C01176	--	329.21286
Com_83724_pos	1-Methylhistidine	C7 H11 N3 O2	169.08447	1.374	cpd:C01152	HMDB0000001	339.17596
Com_10297_neg	Pyrogallol	C6 H6 O3	126.03212	1.081	cpd:C01108	HMDB0013674	125.02481
Com_430_pos	Trehalose	C12 H22 O11	342.11651	1.353	cpd:C01083	HMDB0000975	343.12308
Com_18139_neg	α,α-Trehalose	C12 H22 O11	342.11714	8.371	cpd:C01083	--	341.10986
Com_104354_pos	4-Guanidinobutyric acid	C5 H11 N3 O2	128.05889	1.701	cpd:C01035	HMDB0003464	146.09274
Com_65597_pos	N8-Acetylspermidine	C9 H21 N3 O	187.16825	1.13	cpd:C01029	HMDB0002189	188.17552
Com_43238_pos	6-Hydroxynicotinic acid	C6 H5 N O3	139.02736	2.918	cpd:C01020	HMDB0002658	140.03465
Com_52833_pos	L-Fucose	C6 H12 O5	164.06787	1.379	cpd:C01019	HMDB0000174	165.07582
Com_49037_pos	5-Hydroxytryptophan	C11 H12 N2 O3	220.08482	8.134	cpd:C01017	HMDB0000472	221.09224
Com_12466_pos	O-Phospho-L-serine	C3 H8 N O6 P	185.00916	1.28	cpd:C01005	HMDB0001721	186.01653
Com_6361_pos	Trigonelline	C7 H7 N O2	137.04796	1.364	cpd:C01004	HMDB0000875	138.0551
Com_12130_pos	D-Ala-D-Ala	C6 H12 N2 O3	160.08509	1.361	cpd:C00993	HMDB0003459	161.09232
Com_21350_pos	N-Acetylserotonin	C12 H14 N2 O2	218.10592	7.689	cpd:C00978	HMDB0001238	219.11328
Com_1274_neg	Prostaglandin B1	C20 H32 O4	336.23067	12.368	cpd:C00959	HMDB0002982	335.22327
Com_2458_pos	Indole-3-acetic acid	C10 H9 N O2	175.06343	9.63	cpd:C00954	HMDB0000197	176.07076

Continuation of Supplementary Table 2

Compound_ID	Name	Formula	Molecular Weight	RT [min]	Kegg_ID	HMDB_ID	m/z
Com_3466_neg	Estradiol	C18 H24 O2	272.17816	9.915	cpd:C00951	HMDB0000151	271.17102
Com_24383_neg	β-Estradiol	C18 H24 O2	544.35286	13.329	cpd:C00951	--	543.34558
Com_1356_neg	5-Dehydroquinic acid	C7 H10 O6	190.04821	1.085	cpd:C00944	HMDB0012710	189.04094
Com_53935_pos	cGMP	C10 H12 N5 O7 P	345.04735	1.42	cpd:C00942	HMDB0001314	346.05463
Com_13379_pos	Porphobilinogen	C10 H14 N2 O4	226.09517	2.156	cpd:C00931	HMDB0000245	227.10251
Com_63516_pos	3-Methyladenine	C6 H7 N5	149.07064	5.823	cpd:C00913	HMDB0011600	150.07788
Com_440_pos	Pantothenic acid	C9 H17 N O5	219.11084	6.261	cpd:C00864	HMDB0000210	220.11787
Com_31640_pos	Formononetin	C16 H12 O4	268.07356	11.627	cpd:C00858	HMDB0005808	269.0809
Com_6281_pos	Chlorogenic acid	C16 H18 O9	354.09539	7.435	cpd:C00852	HMDB0003164	355.10287
Com_11887_pos	4-Pyridoxic acid	C8 H9 N O4	183.05345	3.541	cpd:C00847	HMDB0000017	184.06071
Com_55216_pos	Sphinganine	C18 H39 N O2	301.29794	15.343	cpd:C00836	HMDB0000269	302.30496
Com_15271_pos	Pantetheine	C11 H22 N2 O4 S	278.13074	8.044	cpd:C00831	HMDB0003426	279.13806
Com_4341_pos	Menaquinone	C31 H40 O2	444.30409	15.308	cpd:C00828	HMDB0001892	445.31152
Com_35180_neg	dopaquinone	C9 H9 N O4	195.05358	8.483	cpd:C00822	HMDB0001229	194.0463
Com_61384_pos	D-Glucarate	C6 H10 O8	210.03714	6.809	cpd:C00818	HMDB0000663	211.04448
Com_73197_pos	Biochanin A	C16 H12 O5	284.07164	6.967	cpd:C00814	HMDB0002338	285.07892
Com_7414_neg	Salicylic acid	C7 H6 O3	138.03209	5.943	cpd:C00805	HMDB0000840	137.02477
Com_580_neg	Valeric acid	C5 H10 O2	102.0683	3.837	cpd:C00803	HMDB0000892	101.06096
Com_1563_pos	Creatinine	C4 H7 N3 O	113.05912	1.308	cpd:C00791	HMDB0000562	114.0664
Com_9235_pos	L-Adrenaline	C9 H13 N O3	183.0896	1.395	cpd:C00788	HMDB0000068	201.12341
Com_5350_pos	Urocanic acid	C6 H6 N2 O2	138.04319	1.385	cpd:C00785	HMDB0000301	139.05051
Com_101_pos	Serotonin	C10 H12 N2 O	176.09505	3.449	cpd:C00780	HMDB0000259	177.10243
Com_12526_neg	Phloretin	C15 H14 O5	274.08439	10.743	cpd:C00774	HMDB0003306	273.07693

Continuation of Supplementary Table 2

Compound_ID	Name	Formula	Molecular Weight	RT [min]	Kegg_ID	HMDB_ID	m/z
Com_10679_pos	Flavanone	C15 H12 O2	224.08369	9.6	cpd:C00766	--	225.09096
Com_2662_neg	D-Proline	C5 H9 N O2	115.06371	1.379	cpd:C00763	HMDB0003411	114.05643
Com_21052_pos	Cortisone	C21 H28 O5	360.19124	12.072	cpd:C00762	HMDB0002802	361.19846
Com_35111_pos	Berberine	C20 H17 N O4	335.11593	9.171	cpd:C00757	HMDB0003409	336.12289
Com_26451_pos	Vanillin	C8 H8 O3	170.05815	6.68	cpd:C00755	HMDB0012308	171.06534
Com_2771_pos	(S)-Nicotine	C10 H14 N2	162.11595	2.01	cpd:C00745	HMDB0001934	163.12317
Com_25643_pos	Nicotine	C10 H14 N2	162.11595	1.372	cpd:C00745	HMDB0001934	163.12317
Com_36623_pos	Cortisol	C21 H30 O5	724.41772	13.678	cpd:C00735	HMDB0000063	725.42499
Com_19678_pos	Hydrocortisone	C21 H30 O5	362.20921	14.001	cpd:C00735	HMDB0000063	363.21664
Com_168_pos	Tropine	C8 H15 N O	141.11565	1.966	cpd:C00729	--	142.12283
Com_9390_neg	Lipoic acid	C8 H14 O2 S2	160.03756	1.205	cpd:C00725	HMDB0001451	159.03035
Com_362_pos	Oleic acid	C18 H34 O2	282.25616	14.346	cpd:C00712	HMDB0000207	283.26312
Com_13943_neg	Prostaglandin D2	C20 H32 O5	334.215	9.798	cpd:C00696	HMDB0001403	333.20761
Com_2053_neg	Cholic acid	C24 H40 O5	408.28824	12.51	cpd:C00695	HMDB0000619	407.28091
Com_35039_neg	Trehalose 6-phosphate	C12 H23 O14 P	422.06904	1.087	cpd:C00689	HMDB0001124	421.06177
Com_3933_neg	gamma-Glutamylcysteine	C8 H14 N2 O5 S	250.06339	12.37	cpd:C00669	HMDB0001049	249.05618
Com_43240_pos	4-Hydroxymandelonitrile	C8 H7 N O2	149.0482	7.969	cpd:C00650	--	150.0555
Com_13803_neg	Prostaglandin F2alpha	C20 H34 O5	354.24129	13.026	cpd:C00639	HMDB0010199	353.23404
Com_10048_pos	Prostaglandin F2α	C20 H34 O5	336.22773	13.688	cpd:C00639	--	337.23499
Com_987_neg	4-Hydroxybenzaldehyde	C7 H6 O2	122.0372	7.849	cpd:C00633	HMDB0011718	121.02997
Com_340_neg	Gentisic acid	C7 H6 O4	154.02697	2.321	cpd:C00628	HMDB0000152	153.01961
Com_25035_neg	Phenylacetaldehyde	C8 H8 O	120.05785	6.715	cpd:C00601	HMDB0041610	119.05063
Com_10696_neg	3-Hydroxybenzoic acid	C7 H6 O3	138.03212	1.89	cpd:C00587	HMDB0002466	137.02489

Continuation of Supplementary Table 2

Compound_ID	Name	Formula	Molecular Weight	RT [min]	Kegg_ID	HMDB_ID	m/z
Com_43287_neg	cAMP	C10 H12 N5 O6 P	329.05336	4.859	cpd:C00575	HMDB0000058	328.04608
Com_18833_pos	4-Aminobenzoic acid	C7 H7 N O2	137.04801	5.438	cpd:C00568	HMDB0004992	138.05515
Com_7065_pos	2'-Deoxyadenosine	C10 H13 N5 O3	251.10207	3.265	cpd:C00559	HMDB0000101	252.10957
Com_107016_pos	Deoxyadenosine	C10 H13 N5 O3	251.10234	8.123	cpd:C00559	HMDB0000050	252.10962
Com_32565_pos	Noradrenaline	C8 H11 N O3	169.07434	10.14	cpd:C00547	HMDB0037685	361.13806
Com_7654_pos	Homogentisic Acid	C8 H8 O4	168.04258	12.023	cpd:C00544	HMDB0001336	169.0493
Com_15854_pos	Testosterone	C19 H28 O2	288.20911	14.6	cpd:C00535	HMDB0002833	289.21643
Com_22650_pos	Pyridoxamine	C8 H12 N2 O2	168.09012	1.297	cpd:C00534	HMDB0001431	169.09746
Com_44908_neg	2-Deoxyuridine	C9 H12 N2 O5	228.07518	1.204	cpd:C00526	HMDB0000012	273.07339
Com_27190_pos	Androsterone	C19 H30 O2	272.21403	15.274	cpd:C00523	HMDB0000031	273.22137
Com_65_neg	Naringenin	C15 H12 O5	272.06906	10.565	cpd:C00509	HMDB0002670	271.06186
Com_26149_neg	L-Cysteic acid	C3 H7 N O5 S	169.00483	1.204	cpd:C00506	HMDB0002757	167.99759
Com_55675_pos	Folic acid	C19 H19 N7 O6	441.14246	1.395	cpd:C00504	HMDB0000121	442.1503
Com_58579_pos	Biliverdin	C33 H34 N4 O6	582.24779	11.781	cpd:C00500	HMDB0001008	583.25507
Com_11538_neg	L-Cystine	C6 H12 N2 O4 S2	240.02755	6.06	cpd:C00491	HMDB0000192	239.02025
Com_63327_pos	Glutaric Acid	C5 H8 O4	132.04257	1.348	cpd:C00489	HMDB0000661	133.04951
Com_11002_neg	Bilirubin	C33 H36 N4 O6	584.26481	12.819	cpd:C00486	HMDB0000054	583.25745
Com_1304_pos	Tyramine	C8 H11 N O	137.08439	3.46	cpd:C00483	HMDB0000306	138.09164
Com_42311_pos	Cytidine	C9 H13 N3 O5	486.16681	10.434	cpd:C00475	HMDB0000089	487.17389
Com_20261_pos	Vitamin A	C20 H30 O	286.22905	14.178	cpd:C00473	HMDB0000305	287.23633
Com_4987_neg	Estrone	C18 H22 O2	316.16797	11.946	cpd:C00468	HMDB0000145	315.16089
Com_2140_pos	Indole	C8 H7 N	117.05818	10.459	cpd:C00463	HMDB0035924	118.06546
Com_18549_neg	Nicotinamide mononucleotide	C11 H15 N2 O8 P	334.05457	1.06	cpd:C00455	HMDB0000229	333.04715

Continuation of Supplementary Table 2

Compound_ID	Name	Formula	Molecular Weight	RT [min]	Kegg_ID	HMDB_ID	m/z
Com_9061_pos	D-threo-Isocitric acid	C6 H8 O7	192.02697	1.587	cpd:C00451	HMDB0001874	193.03401
Com_14025_pos	L-Saccharopine	C11 H20 N2 O6	276.13194	1.376	cpd:C00449	HMDB0000279	277.13889
Com_10214_neg	Sedoheptulose 1,7-bisphosphate	C7 H16 O13 P2	370.005	1.443	cpd:C00447	--	368.99783
Com_17594_pos	N-Acetylornithine	C7 H14 N2 O3	174.10059	2.069	cpd:C00437	HMDB0003357	175.10793
Com_1685_neg	5-Aminovaleric acid	C5 H11 N O2	117.07937	1.436	cpd:C00431	HMDB0003355	116.07212
Com_12920_neg	5-Aminopentanoate	C5 H11 N O2	117.07941	7.975	cpd:C00431	HMDB0003355	116.07214
Com_9062_neg	Prostaglandin H2	C20 H32 O5	352.22563	12.152	cpd:C00427	HMDB0001381	351.21814
Com_30888_neg	trans-Cinnamic acid	C9 H8 O2	148.05299	10.045	cpd:C00423	HMDB0000930	147.04572
Com_7943_neg	Mevalonic acid	C6 H12 O4	148.07399	1.394	cpd:C00418	HMDB0000227	147.06682
Com_4735_pos	Progesterone	C21 H30 O2	314.22489	10.933	cpd:C00410	HMDB0001830	315.23227
Com_10_pos	Pipecolic acid	C6 H11 N O2	129.079	1.385	cpd:C00408	HMDB0000716	130.0862
Com_4525_neg	Quercetin	C15 H10 O7	302.04319	11.241	cpd:C00389	HMDB0005794	301.03589
Com_51153_pos	Histamine	C5 H9 N3	111.08004	1.088	cpd:C00388	HMDB0000870	112.08732
Com_3624_neg	Guanosine	C10 H13 N5 O5	283.09207	3.512	cpd:C00387	HMDB0000133	282.08456
Com_21244_neg	Carnosine	C9 H14 N4 O3	226.10566	1.403	cpd:C00386	HMDB0000033	225.09853
Com_553_pos	Xanthine	C5 H4 N4 O2	152.03372	2.13	cpd:C00385	HMDB0000292	153.04082
Com_11041_pos	Cytosine	C4 H5 N3 O	111.04346	1.305	cpd:C00380	HMDB0000630	112.05078
Com_24884_pos	Xylitol	C5 H12 O5	152.06978	1.359	cpd:C00379	HMDB0002917	153.07713
Com_24285_pos	Thiamine	C12 H16 N4 O S	264.10428	1.302	cpd:C00378	HMDB0000235	265.11191
Com_8421_neg	Uric acid	C5 H4 N4 O3	168.02882	1.362	cpd:C00366	HMDB0000289	167.02147
Com_38293_neg	dUMP	C9 H13 N2 O8 P	308.04163	1.74	cpd:C00365	HMDB0001409	307.03424
Com_52933_pos	dAMP	C10 H14 N5 O6 P	331.06909	7.03	cpd:C00360	HMDB0000905	332.07654
Com_54723_pos	L-Dopa	C9 H11 N O4	197.06878	6.796	cpd:C00355	HMDB0000181	198.0762

Continuation of Supplementary Table 2

Compound_ID	Name	Formula	Molecular Weight	RT [min]	Kegg_ID	HMDB_ID	m/z
Com_37381_neg	Levodopa	C9 H11 N O4	197.06935	1.21	cpd:C00355	HMDB0000181	196.06181
Com_17166_neg	D-Fructose 1,6-bisphosphate	C6 H14 O12 P2	339.99686	1.369	cpd:C00354	HMDB0001058	338.9895
Com_7129_neg	Geranylgeranyl pyrophosphate	C20 H36 O7 P2	450.19332	11.106	cpd:C00353	HMDB0004486	449.18625
Com_10927_pos	D-Glucosamine 6-phosphate	C6 H14 N O8 P	259.04565	1.343	cpd:C00352	HMDB0001254	260.05313
Com_25181_neg	Deoxyguanosine	C10 H13 N5 O4	267.0961	1.379	cpd:C00330	HMDB0000085	266.08887
Com_8940_pos	D-Glucosamine	C6 H13 N O5	179.07946	1.347	cpd:C00329	HMDB0001514	180.08577
Com_28470_pos	L-Kynurenine	C10 H12 N2 O3	208.08458	1.394	cpd:C00328	HMDB0000684	209.0919
Com_12314_neg	Citrulline	C6 H13 N3 O3	175.09617	1.294	cpd:C00327	HMDB0000904	174.08882
Com_7910_neg	L-Carnitine	C7 H15 N O3	161.10559	1.398	cpd:C00318	HMDB0000062	160.09853
Com_4135_pos	Pyridoxine	C8 H11 N O3	169.07416	2.116	cpd:C00314	HMDB0000239	170.08141
Com_42647_neg	Citicoline	C14 H26 N4 O11 P2	488.10927	11.062	cpd:C00307	HMDB0001413	487.10199
Com_44979_pos	Kanamycin	C18 H36 N4 O11	506.21607	7.59	cpd:C00304	--	507.22357
Com_18203_neg	Glutamine	C5 H10 N2 O3	146.06952	6.646	cpd:C00303	HMDB0000641	145.06226
Com_47558_neg	Adenosine diphosphate ribose	C15 H23 N5 O14 P2	559.07069	1.093	cpd:C00301	HMDB0001178	558.06342
Com_1576_pos	Creatine	C4 H9 N3 O2	131.06979	1.338	cpd:C00300	HMDB0000064	132.07697
Com_2829_neg	Uridine	C9 H12 N2 O6	244.06992	2.224	cpd:C00299	HMDB0000296	243.06259
Com_20038_pos	Quinic acid	C7 H12 O6	192.06319	1.369	cpd:C00296	HMDB0003072	193.07047
Com_8764_pos	Inosine	C10 H12 N4 O5	268.08071	3.359	cpd:C00294	HMDB0000195	269.08832
Com_11410_pos	Androstenedione	C19 H26 O2	286.19334	10.433	cpd:C00280	HMDB0000053	287.20056
Com_26093_pos	D-Erythrose 4-phosphate	C4 H9 O7 P	200.00858	1.208	cpd:C00279	HMDB0001321	201.01567
Com_1149_neg	D-Mannose 6-phosphate	C6 H13 O9 P	260.0302	1.173	cpd:C00275	HMDB0001078	259.02298

Continuation of Supplementary Table 2

Compound_ID	Name	Formula	Molecular Weight	RT [min]	Kegg_ID	HMDB_ID	m/z
Com_26866_neg	N-Acetylneuraminic acid	C11 H19 N O9	309.10666	1.229	cpd:C00270	HMDB0000230	308.0993
Com_1027_pos	Hypoxanthine	C5 H4 N4 O	136.03874	1.534	cpd:C00262	HMDB0000157	137.04601
Com_14935_neg	Riboflavin	C17 H20 N4 O6	376.13914	8.225	cpd:C00255	HMDB0001520	375.13156
Com_8878_pos	Vitamin B2	C17 H20 N4 O6	376.137	13.274	cpd:C00255	HMDB0000244	377.14429
Com_295_pos	Nicotinic acid	C6 H5 N O2	123.03226	1.869	cpd:C00253	HMDB0001488	124.03952
Com_57778_pos	Pyridoxal	C8 H9 N O3	167.05859	7.498	cpd:C00250	HMDB0001545	168.0659
Com_9_neg	Palmitic acid	C16 H32 O2	256.24018	14.604	cpd:C00249	HMDB0000220	255.23283
Com_23021_neg	lipoamide	C8 H15 N O S2	205.05897	1.162	cpd:C00248	HMDB0000962	204.05135
Com_88188_pos	Taurine	C2 H7 N O3 S	125.01485	1.327	cpd:C00245	HMDB0000251	126.02228
Com_2413_pos	Guanine	C5 H5 N5 O	151.0497	1.65	cpd:C00242	HMDB0000132	152.05685
Com_10784_neg	dCMP	C9 H14 N3 O7 P	307.05712	2.294	cpd:C00239	HMDB0001202	306.04984
Com_21097_pos	Protocatechuic acid	C7 H6 O4	154.0271	7.09	cpd:C00230	HMDB0001856	155.03458
Com_30366_neg	acetyl phosphate	C2 H5 O5 P	139.98791	1.185	cpd:C00227	HMDB0001494	138.98067
Com_337_neg	Arachidonic acid	C20 H32 O2	304.24186	14.311	cpd:C00219	HMDB0001043	303.23502
Com_5624_neg	Thymidine	C10 H14 N2 O5	242.09076	5.592	cpd:C00214	HMDB0000273	241.08344
Com_3323_pos	Adenosine	C10 H13 N5 O4	267.09715	2.606	cpd:C00212	HMDB0000050	268.10437
Com_8867_neg	Gluconolactone	C6 H10 O6	178.04819	1.284	cpd:C00198	HMDB0000150	177.04099
Com_22870_neg	2,3-Dihydroxybenzoic acid	C7 H6 O4	154.02694	1.376	cpd:C00196	HMDB0000397	153.01961
Com_13355_pos	L-Threonine	C4 H9 N O3	119.05863	1.381	cpd:C00188	HMDB0000167	120.06585
Com_36967_pos	Cholesterol	C27 H46 O	386.35514	15.361	cpd:C00187	HMDB0000067	387.36243
Com_1064_pos	L-Valine	C5 H11 N O2	117.07923	1.439	cpd:C00183	HMDB0000883	118.08654
Com_642_neg	Benzoic acid	C7 H6 O2	122.03711	2.707	cpd:C00180	HMDB0004461	121.02979
Com_10459_pos	Thymine	C5 H6 N2 O2	126.04326	5.617	cpd:C00178	HMDB0000262	127.05059

Continuation of Supplementary Table 2

Compound_ID	Name	Formula	Molecular Weight	RT [min]	Kegg_ID	HMDB_ID	m/z
Com_3115_neg	Phenylpyruvic acid	C9 H8 O3	164.04773	11.552	cpd:C00166	HMDB0000205	163.04054
Com_14317_pos	acetoacetate	C4 H6 O3	102.03178	1.328	cpd:C00164	HMDB0000060	103.03923
Com_21091_pos	4-Hydroxybenzoic acid	C7 H6 O3	138.03189	8.424	cpd:C00156	HMDB0000500	139.03915
Com_14410_pos	Nicotinamide	C6 H6 N2 O	122.04834	1.659	cpd:C00153	HMDB0001406	123.05562
Com_57868_pos	L-Asparagine	C4 H8 N2 O3	132.05375	1.337	cpd:C00152	HMDB0000168	133.06117
Com_809_neg	Adenine	C5 H5 N5	135.05485	3.474	cpd:C00147	HMDB0000034	134.04755
Com_14858_pos	Guanosine monophosphate	C10 H14 N5 O8 P	363.05713	1.406	cpd:C00144	HMDB0001397	364.06454
Com_7652_neg	3-Methyl-2-oxobutanoic acid	C5 H8 O3	116.04764	1.216	cpd:C00141	HMDB0000019	115.04044
Com_1955_pos	Inositol	C6 H12 O6	180.06359	1.271	cpd:C00137	HMDB0000211	219.02676
Com_77913_pos	L-Histidine	C6 H9 N3 O2	155.06963	6.479	cpd:C00135	HMDB0000177	156.0769
Com_17747_neg	dATP	C10 H16 N5 O12 P3	490.99869	1.402	cpd:C00131	HMDB0001532	489.99139
Com_53317_pos	Inosine 5'-Monophosphate	C10 H13 N4 O8 P	348.04691	1.39	cpd:C00130	HMDB0000175	349.05402
Com_37945_pos	glutathione disulfide	C20 H32 N6 O12 S2	612.14918	7.553	cpd:C00127	HMDB0003337	613.15649
Com_6485_neg	Fumaric acid	C4 H4 O4	116.01132	1.163	cpd:C00122	HMDB0000134	115.004
Com_14736_neg	Biotin	C10 H16 N2 O3 S	244.0886	5.736	cpd:C00120	HMDB0000030	243.08125
Com_11954_neg	Glyceraldehyde 3-phosphate	C3 H7 O6 P	169.99858	1.23	cpd:C00118	--	168.99118
Com_1465_pos	Choline	C5 H13 N O	103.09987	1.273	cpd:C00114	HMDB0000097	104.10706
Com_36120_neg	CDP	C9 H15 N3 O11 P2	403.01906	1.1	cpd:C00112	HMDB0001546	402.01178
Com_81574_pos	Anthranilic acid	C7 H7 N O2	137.04711	15.076	cpd:C00108	HMDB0001123	297.08344
Com_1341_neg	Uracil	C4 H4 N2 O2	112.02757	1.745	cpd:C00106	HMDB0000300	111.02028
Com_23911_pos	UMP	C9 H13 N2 O9 P	324.03548	1.41	cpd:C00105	HMDB0000288	325.04279

Continuation of Supplementary Table 2

Compound_ID	Name	Formula	Molecular Weight	RT [min]	Kegg_ID	HMDB_ID	m/z
Com_1110_pos	L-Tyrosine	C9 H11 N O3	181.07416	2.312	cpd:C00082	HMDB0000158	182.08134
Com_309_pos	L-Phenylalanine	C9 H11 N O2	165.07906	5.511	cpd:C00079	HMDB0000159	166.08609
Com_7452_pos	L-Tryptophan	C11 H12 N2 O2	204.09027	5.315	cpd:C00078	HMDB0000929	205.0974
Com_591_pos	L-Ornithine	C5 H12 N2 O2	132.09014	1.179	cpd:C00077	HMDB0000214	133.09741
Com_2598_pos	Methionine	C5 H11 N O2 S	149.05133	1.674	cpd:C00073	HMDB0000696	150.05862
Com_2943_pos	L-Ascorbate	C6 H8 O6	176.03246	2.575	cpd:C00072	HMDB0000044	177.03943
Com_195_neg	Ascorbic acid	C6 H8 O6	176.03223	1.099	cpd:C00072	HMDB0000044	175.0247
Com_42659_neg	TPP	C12 H19 Cl N4 O7 P2 S	460.014	1.02	cpd:C00068	HMDB0001372	459.0061
Com_12504_neg	L-Serine	C3 H7 N O3	105.04305	1.25	cpd:C00065	HMDB0000187	104.0358
Com_45157_pos	Riboflavin-5-phosphate	C17 H21 N4 O9 P	456.10376	11.468	cpd:C00061	HMDB0001520	457.11078
Com_12772_neg	Cytidine-5'-monophosphate	C9 H14 N3 O8 P	323.05248	1.329	cpd:C00055	HMDB0000095	322.04526
Com_35950_pos	Glutathione	C10 H17 N3 O6 S	307.08506	14.488	cpd:C00051	HMDB0000125	308.09128
Com_2085_neg	L-Aspartic acid	C4 H7 N O4	133.03789	1.207	cpd:C00049	HMDB0000191	132.03062
Com_624_neg	Succinic acid	C4 H6 O4	118.02692	1.169	cpd:C00042	HMDB0000254	117.01959
Com_28008_neg	Oxaloacetate	C4 H4 O5	132.00617	1.084	cpd:C00036	HMDB0000223	130.99896
Com_27527_neg	alpha-Ketoglutaric acid	C5 H6 O5	146.02193	1.186	cpd:C00026	HMDB0000208	145.01451
Com_687_pos	L-Glutamic acid	C5 H9 N O4	147.05335	1.305	cpd:C00025	HMDB0000148	148.06059
Com_36805_neg	5'-Adenylic acid	C10 H14 N5 O7 P	347.06478	6.218	cpd:C00020	HMDB0011617	346.05756
Com_13963_neg	Adenosine 5'-monophosphate	C10 H14 N5 O7 P	347.06366	1.434	cpd:C00020	HMDB0000045	346.05627
Com_6056_neg	S-Adenosyl-L-methionine	C15 H22 N6 O5 S	398.13725	8.948	cpd:C00019	HMDB0001185	397.12991
Com_30182_neg	Flavin adenine dinucleotide	C27 H33 N9 O15 P2	785.15961	6.912	cpd:C00016	HMDB0001248	784.15253

Continuation of Supplementary Table 2

Compound_ID	Name	Formula	Molecular Weight	RT [min]	Kegg_ID	HMDB_ID	m/z
Com_31890_pos	alpha-Benzylsuccinic acid	C11 H12 O4	208.07351	9.032	--	HMDB0142179	209.08109
Com_110844_pos	1,7-bis(4-hydroxyphenyl)heptan-3-one	C19 H22 O3	280.14742	10.151	--	HMDB0138216	281.15469
Com_3131_pos	1,7-bis(4-hydroxyphenyl)-5-methoxyheptan-3-one	C20 H24 O4	350.14716	6.885	--	HMDB0138162	351.15442
Com_54201_pos	4-hydroxy-3-(3-methylbut-2-en-1-yl)benzoic acid	C12 H14 O3	206.0944	11.405	--	HMDB0135898	207.10181
Com_1094_pos	2-Hydroxycinnamic acid	C9 H8 O3	164.04764	2.383	--	HMDB0134028	165.05495
Com_2828_pos	8-(1,2-dihydroxy-3-methylbut-3-en-1-yl)-7-methoxy-2H-chromen-2-one	C15 H16 O5	276.09971	10.177	--	HMDB0132703	277.10709
Com_8301_neg	7-hydroxy-3-phenyl-4H-chromen-4-one	C15 H10 O3	238.06112	1.744	--	HMDB0130492	237.05379
Com_18150_neg	3,4,5-trihydroxycyclohex-1-ene-1-carboxylic acid	C7 H10 O5	174.05326	1.732	--	HMDB0130150	173.04601
Com_5081_pos	Isorhapontigenin	C15 H14 O4	258.08904	12.21	--	HMDB0128522	259.09628
Com_3300_pos	3,5-Dimethoxybenzoic acid	C9 H10 O4	182.05814	10.896	--	HMDB0127495	183.06537
Com_12700_pos	Arachidonic acid methyl ester	C21 H34 O2	318.25598	12.514	--	HMDB0062594	319.26328
Com_6947_neg	13Z,16Z-Docosadienoic Acid	C22 H40 O2	336.30377	15.258	--	HMDB0062219	335.29657
Com_12660_pos	Noroxymorphone	C16 H17 N O4	287.11576	10.711	--	HMDB0061073	288.12308
Com_50514_pos	N-Desmethyltramadol	C15 H23 N O2	267.18341	11.87	--	HMDB0061007	268.1907
Com_8070_neg	Acetildenafil	C25 H34 N6 O3	466.27075	14.092	--	HMDB0060932	465.26367
Com_14730_pos	N-Acetyl-5-aminosalicylic acid	C9 H9 N O4	195.05345	7.087	--	HMDB0060602	196.06076

Continuation of Supplementary Table 2

Compound_ID	Name	Formula	Molecular Weight	RT [min]	Kegg_ID	HMDB_ID	m/z
Com_98892_pos	Norbuprenorphine	C25 H35 N O4	413.2572	9.18	--	HMDB0060546	414.26416
Com_58047_pos	Norverapamil	C26 H36 N2 O4	440.26391	6.835	--	HMDB0060540	441.271
Com_13811_pos	13,14-dihydro-15-keto-PGD2	C20 H32 O5	352.22392	14.179	--	HMDB0060042	353.2312
Com_13927_pos	3-hydroxy-3-methylpentanedioic acid	C6 H10 O5	184.037	12.324	--	HMDB0059737	185.04428
Com_1893_neg	3-Hydroxy-3-methylglutaric acid	C6 H10 O5	162.05331	1.318	--	HMDB0059737	161.04614
Com_37923_pos	Ritalinic acid	C13 H17 N O2	219.12625	7.562	--	HMDB0042008	220.13365
Com_44665_pos	Noroxycodone	C17 H19 N O4	301.13143	6.514	--	HMDB0041960	302.13898
Com_40025_pos	N-Acetyldopamine	C10 H13 N O3	173.10552	8.833	--	HMDB0041943	174.1127
Com_20414_neg	2-Butoxyacetic acid	C6 H12 O3	132.07902	1.191	--	HMDB0041844	131.07173
Com_60291_pos	7-Aminoflunitrazepam	C16 H14 F N3 O	283.11054	10.573	--	HMDB0041818	284.1181
Com_42402_pos	2-(3,4-dimethoxyphenyl)ethanamine	C10 H15 N O2	181.11022	1.368	--	HMDB0041806	182.11748
Com_64576_pos	Lactitol	C12 H24 O11	344.13177	1.293	--	HMDB0040937	345.13995
Com_14857_neg	Dihydroroseoside	C19 H32 O8	388.21055	8.346	--	HMDB0040614	387.20328
Com_2511_pos	Isopropyl myristate	C17 H34 O2	287.28253	13.195	--	HMDB0040392	288.29004
Com_73101_pos	Ascorbyl palmitate	C22 H38 O7	431.28887	12.228	--	HMDB0039883	432.29611
Com_25400_pos	Neodiosmin	C28 H32 O15	608.17496	9.677	--	HMDB0039856	609.18219
Com_13049_pos	Eicosapentaenoic acid ethyl ester	C22 H34 O2	330.25601	14.456	--	HMDB0039530	331.26312
Com_61643_pos	Trolox	C14 H18 O4	228.13648	11.509	--	HMDB0038804	229.14359
Com_16547_pos	Dinophysistoxin-2	C44 H68 O13	782.48321	12.52	--	HMDB0038517	783.49023

Continuation of Supplementary Table 2

Compound_ID	Name	Formula	Molecular Weight	RT [min]	Kegg_ID	HMDB_ID	m/z
Com_8636_pos	Ergosterol peroxide	C28 H44 O3	428.33178	14.042	--	HMDB0037941	429.33835
Com_1967_pos	p-Mentha-1,3,8-triene	C10 H14	134.10972	9.286	--	HMDB0037013	135.11699
Com_9749_pos	Oxaceprol	C7 H11 N O4	173.06893	1.392	--	HMDB0036576	174.07616
Com_17641_pos	4-Deacetylneosolaniol	C17 H24 O7	322.14186	12.847	--	HMDB0036158	323.14914
Com_1774_neg	Corchorifatty acid F	C18 H32 O5	328.2255	10.939	--	HMDB0035919	327.21814
Com_20692_pos	Perillartine	C10 H15 N O	165.11564	9.431	--	HMDB0035652	166.12305
Com_55_pos	(+)-ar-Turmerone	C15 H20 O	216.15148	13.073	--	HMDB0035612	217.15869
Com_730_pos	Kahweol	C20 H26 O3	314.18846	12.451	--	HMDB0035602	315.19574
Com_16893_pos	T-2 Triol	C20 H30 O7	404.18141	11.415	--	HMDB0035396	405.18872
Com_42_pos	Cryptotanshinone	C19 H20 O3	296.14162	13.557	--	HMDB0035220	297.14865
Com_8488_pos	Ethyl oleate	C20 H38 O2	328.29795	14.914	--	HMDB0034451	329.30487
Com_13423_pos	Sedanolide	C12 H18 O2	176.12026	10.203	--	HMDB0034450	177.1275
Com_57430_pos	Withanolide A	C28 H38 O6	470.26994	11.435	--	HMDB0034415	471.27759
Com_24947_pos	gamma-Glutamylmethionine	C10 H18 N2 O5 S	278.09344	5.46	--	HMDB0034367	279.10095
Com_36818_pos	myricetin 3-O-beta-D-galactopyranoside	C21 H20 O13	480.09063	8.728	--	HMDB0034358	481.09738
Com_27408_pos	(2E)-3-(3,4-dimethoxyphenyl)prop-2-enoic acid	C11 H12 O4	190.06309	8.73	--	HMDB0034315	191.0703
Com_12557_pos	Muscone	C16 H30 O	238.22967	12.613	--	HMDB0034181	239.23703
Com_90671_pos	Isoeugenyl acetate	C12 H14 O3	206.09438	13.866	--	HMDB0034135	207.10164
Com_37109_pos	cis-Resveratrol	C14 H12 O3	228.07876	8.033	--	HMDB0034118	229.08615
Com_1594_neg	D-Raffinose	C18 H32 O16	504.16772	10	--	HMDB0034072	503.16049

Continuation of Supplementary Table 2

Compound_ID	Name	Formula	Molecular Weight	RT [min]	Kegg_ID	HMDB_ID	m/z
Com_49596_pos	4-Ethoxybenzaldehyde	C9 H10 O2	150.06843	14.597	--	HMDB0033970	151.0757
Com_19_neg	Lauric acid ethyl ester	C14 H28 O2	228.20887	14.12	--	HMDB0033788	227.20139
Com_25230_neg	Sulfaquinoxaline	C14 H12 N4 O2 S	300.07068	8.529	--	HMDB0033139	299.06345
Com_635_pos	6-Methylquinoline	C10 H9 N	143.07372	7.008	--	HMDB0033115	144.08099
Com_4060_pos	4-Methyl-5-thiazoleethanol	C6 H9 N O S	143.04088	5.116	--	HMDB0032985	144.04805
Com_28023_pos	1-(4-Methoxyphenyl)-2-propanone	C10 H12 O2	164.0838	8.476	--	HMDB0032891	165.09132
Com_14121_neg	Sinapinic acid	C11 H12 O5	224.06902	6.078	--	HMDB0032616	223.06183
Com_23950_neg	3-Anisic acid	C8 H8 O3	152.04774	9.485	--	HMDB0032606	151.04047
Com_13038_neg	2-Anisic acid	C8 H8 O3	152.04782	2.945	--	HMDB0032604	151.04054
Com_23682_neg	Butylparaben	C11 H14 O3	194.09476	12.067	--	HMDB0032575	193.08748
Com_6594_neg	Propylparaben	C10 H12 O3	180.079	7.515	--	HMDB0032574	179.07169
Com_14942_pos	4'-Methoxyacetophenone	C9 H10 O2	150.06857	15.306	--	HMDB0032570	151.07584
Com_23991_pos	1,3-Diphenylacetone	C15 H14 O	210.10452	13.026	--	HMDB0032561	211.11179
Com_7646_pos	2,4-Dimethylbenzaldehyde	C9 H10 O	134.07337	12.083	--	HMDB0032142	135.08066
Com_64315_pos	Veratrole	C8 H10 O2	138.06847	10.166	--	HMDB0032139	139.07578
Com_6955_pos	trans-Cinnamaldehyde	C9 H8 O	132.05781	9.59	--	HMDB0032072	133.06508
Com_34893_pos	N-Acetylhistidine	C8 H11 N3 O3	197.07974	1.376	--	HMDB0032055	198.08693
Com_35667_pos	4-Ethylbenzaldehyde	C9 H10 O	134.07343	10.297	--	HMDB0032024	135.08061
Com_2046_pos	Celestolide	C17 H24 O	244.18264	13.114	--	HMDB0031867	245.18983
Com_24516_pos	2-Isobutyl-3-methoxypyrazine	C9 H14 N2 O	149.08416	8.637	--	HMDB0031860	150.09137
Com_86238_pos	4-Guanidinobutanoic acid	C5 H11 N3 O2	145.08515	7.806	--	HMDB0031842	146.09242
Com_9154_pos	Methyl dihydrojasmonate	C13 H22 O3	226.15673	12.04	--	HMDB0031740	227.16386

Continuation of Supplementary Table 2

Compound_ID	Name	Formula	Molecular Weight	RT [min]	Kegg_ID	HMDB_ID	m/z
Com_1853_pos	9,10-Dihome	C18 H34 O4	314.24596	13.39	--	HMDB0031679	315.25327
Com_7265_pos	4-Phenyl-3-buten-2-one	C10 H10 O	146.07356	4.833	--	HMDB0031617	147.08087
Com_6650_pos	3-Methoxybenzaldehyde	C8 H8 O2	136.05277	11.202	--	HMDB0031459	137.05998
Com_10574_pos	Cyclohexaneacetic acid	C8 H14 O2	142.09976	10.312	--	HMDB0031403	143.10706
Com_200_neg	Cyclamic acid	C6 H13 N O3 S	179.06161	6.858	--	HMDB0031340	178.05434
Com_27878_pos	6-Pentyl-2H-pyran-2-one	C10 H14 O2	166.09969	11.775	--	HMDB0031085	167.10687
Com_22257_pos	Avocadyne 1-acetate	C19 H34 O4	366.23926	13.575	--	HMDB0031048	349.23563
Com_19031_neg	6,7-Dihydroxycoumarin	C9 H6 O4	178.02735	9.187	--	HMDB0030819	177.01982
Com_43921_pos	2-(2,4-dihydroxyphenyl)-3,5,7-trihydroxy-4H-chromen-4-one	C15 H10 O7	302.04259	12.19	--	HMDB0030796	303.04987
Com_48410_neg	4,5-Dicaffeoylquinic acid	C25 H24 O12	516.12772	7.553	--	HMDB0030707	515.12036
Com_34818_neg	Epicatechin-3-o-gallate	C22 H18 O10	442.09111	6.759	--	HMDB0030661	441.08383
Com_25995_neg	1-Caffeoylquinic Acid	C16 H18 O9	354.09581	9.276	--	HMDB0030652	353.08859
Com_28002_pos	Neohesperidin dihydrochalcone	C28 H36 O15	612.21439	10.146	--	HMDB0030542	613.22137
Com_27633_neg	2,5-Dimethylphenol	C8 H10 O	122.07352	9.023	--	HMDB0030540	121.06625
Com_7215_pos	Tangeritin	C20 H20 O7	372.12088	12.653	--	HMDB0030539	373.12802
Com_55638_pos	Virginiamycin	C28 H35 N3 O7	525.24383	10.128	--	HMDB0030520	526.25116
Com_38096_pos	Narasin	C43 H72 O11	382.24672	12.64	--	HMDB0030448	803.45612
Com_87528_pos	Roquefortine C	C22 H23 N5 O2	389.18334	7.025	--	HMDB0030381	390.19061
Com_84032_pos	3-(3,4,5-trimethoxyphenyl)propanoic acid	C12 H16 O5	262.08384	9.824	--	HMDB0030254	263.09106
Com_31095_pos	Sucrose octaacetate	C28 H38 O19	700.18461	1.391	--	HMDB0029893	701.19177

Continuation of Supplementary Table 2

Compound_ID	Name	Formula	Molecular Weight	RT [min]	Kegg_ID	HMDB_ID	m/z
Com_11983_neg	Sorbitan monooleate	C24 H44 O6	428.31481	12.445	--	HMDB0029886	427.3071
Com_61320_pos	Enrofloxacin	C19 H22 F N3 O3	381.15302	6.17	--	HMDB0029861	382.16016
Com_17988_neg	Dantron	C14 H8 O4	240.04286	5.248	--	HMDB0029752	239.03552
Com_9152_pos	Methyl indole-3-acetate	C11 H11 N O2	189.0791	10.388	--	HMDB0029738	190.08655
Com_28276_pos	4-Methoxybenzaldehyde	C8 H8 O2	136.05282	8.41	--	HMDB0029686	137.06017
Com_32169_neg	2,4-Dihydroxybenzoic acid	C7 H6 O4	154.02691	7.492	--	HMDB0029666	153.01961
Com_17491_neg	2,4,6-Trihydroxyacetophenone	C8 H8 O4	168.04277	9.109	--	HMDB0029644	167.03554
Com_13429_pos	Azetidine-2-carboxylic acid	C4 H7 N O2	101.04781	1.32	--	HMDB0029615	102.05517
Com_3906_pos	3-Acetyl-2,5-dimethylfuran	C8 H10 O2	138.06845	7.49	--	HMDB0029563	139.07564
Com_36422_pos	3,5,7-trihydroxy-2-phenyl-4H-chromen-4-one	C15 H10 O5	270.05285	12.078	--	HMDB0029521	271.06009
Com_37693_pos	4',7-Dihydroxyflavanone	C15 H12 O4	256.07347	10.051	--	HMDB0029519	257.0809
Com_92730_pos	Dihydrokawain	C14 H16 O3	232.11008	7.508	--	HMDB0029504	233.11736
Com_19338_pos	Maduramicin	C47 H80 O17	938.52375	13.425	--	HMDB0029453	939.53094
Com_5682_pos	Val-Ser	C8 H16 N2 O4	204.11113	1.405	--	HMDB0029136	205.11855
Com_2826_pos	Valylproline	C10 H18 N2 O3	214.13202	5.588	--	HMDB0029135	215.13939
Com_64416_pos	Tyr-Tyr	C18 H20 N2 O5	344.13686	6.468	--	HMDB0029117	345.14417
Com_35938_neg	Tyrosylalanine	C12 H16 N2 O4	252.11532	8.319	--	HMDB0029098	251.10805
Com_54629_pos	Thr-Leu	C10 H20 N2 O4	232.1415	6.578	--	HMDB0029065	233.14877
Com_40852_pos	Gly-Tyr	C11 H14 N2 O4	238.09526	4.296	--	HMDB0028853	239.10243
Com_11464_pos	Gly-Phe	C11 H14 N2 O3	222.10056	6.94	--	HMDB0028848	223.10764
Com_11110_neg	Gly-Ile	C8 H16 N2 O3	188.1163	2.958	--	HMDB0028844	187.10902
Com_95027_pos	Glu-Thr	C9 H16 N2 O6	248.09982	1.543	--	HMDB0028829	249.1071

Continuation of Supplementary Table 2

Compound_ID	Name	Formula	Molecular Weight	RT [min]	Kegg_ID	HMDB_ID	m/z
Com_53506_pos	Alanyltyrosine	C12 H16 N2 O4	252.11108	5.85	--	HMDB0028699	253.11853
Com_9527_pos	L-Alanyl-L-proline	C8 H14 N2 O3	186.10056	1.409	--	HMDB0028695	187.10799
Com_7233_pos	L-Alanyl-L-Lysine	C9 H19 N3 O3	217.14246	1.406	--	HMDB0028692	218.14973
Com_44194_pos	Ala-Leu	C9 H18 N2 O3	202.13205	6.612	--	HMDB0028691	203.1395
Com_5485_pos	Ala-Ile	C9 H18 N2 O3	202.13214	6.324	--	HMDB0028690	203.1393
Com_6917_pos	Ala-Gln	C8 H15 N3 O4	217.1061	1.351	--	HMDB0028685	218.11337
Com_12615_neg	Mycophenolic acid	C17 H20 O6	320.12674	7.075	--	HMDB0015159	319.11945
Com_2903_pos	Latanoprost	C26 H40 O5	432.28818	11.148	--	HMDB0014792	433.29529
Com_20970_pos	Mesalamine	C7 H7 N O3	153.04303	7.089	--	HMDB0014389	154.05023
Com_26618_neg	Diflorasone	C22 H28 F2 O5	410.18865	9.288	--	HMDB0014368	409.18079
Com_10637_neg	O-Desmethylnaproxen	C13 H12 O3	216.0766	8.746	--	HMDB0013989	215.06937
Com_7897_pos	2,6-Di-tert-butyl-1,4-benzoquinone	C14 H20 O2	220.14641	11.469	--	HMDB0013817	221.15367
Com_56508_pos	DL-Norvaline	C5 H11 N O2	117.0793	10.596	--	HMDB0013716	118.08658
Com_10065_neg	3,5-Dihydroxybenzoic acid	C7 H6 O4	154.02707	7.113	--	HMDB0013677	153.01978
Com_15452_pos	2,6-Dihydroxybenzoic acid	C7 H6 O4	154.02704	4.855	--	HMDB0013676	155.03444
Com_23996_pos	2-Arachidonyl Glycerol ether	C23 H40 O3	386.2803	14.935	--	HMDB0013657	387.28745
Com_5157_pos	O-Arachidonoyl ethanolamine	C22 H37 N O2	347.28017	14.953	--	HMDB0013655	348.28751
Com_43863_pos	2-(14,15-Epoxyeicosatrienoyl) glycerol	C23 H38 O5	394.27021	13.171	--	HMDB0013651	395.27744
Com_724_pos	DL-Tryptophan	C11 H12 N2 O2	204.08997	6.846	--	HMDB0013609	205.09726
Com_1592_neg	Phe-Phe	C18 H20 N2 O3	312.14796	10.052	--	HMDB0013302	311.14124
Com_22031_neg	Monobutyl phthalate	C12 H14 O4	222.08963	6.972	--	HMDB0013247	221.08232

Continuation of Supplementary Table 2

Compound_ID	Name	Formula	Molecular Weight	RT [min]	Kegg_ID	HMDB_ID	m/z
Com_21961_pos	1-Pyrenol	C16 H10 O	218.07333	8.959	--	HMDB0013139	219.08067
Com_17425_pos	Stearoyl Ethanolamide	C20 H41 N O2	327.31393	13.424	--	HMDB0013078	328.32123
Com_7013_neg	Prostaglandin H1	C20 H34 O5	336.23053	11.42	--	HMDB0013041	335.22327
Com_3831_neg	6-Hydroxycaproic acid	C6 H12 O3	132.07901	1.84	--	HMDB0012843	131.0717
Com_16_pos	Hexadecanamide	C16 H33 N O	255.25626	14.892	--	HMDB0012273	256.26349
Com_832_pos	Linoleoyl ethanolamide	C20 H37 N O2	323.28273	14.636	--	HMDB0012252	324.28983
Com_9_pos	N-Acetylvaline	C7 H13 N O3	159.09003	1.384	--	HMDB0011757	160.09726
Com_1472_neg	3-(3-Methoxyphenyl)propionic acid	C10 H12 O3	180.07917	9.923	--	HMDB0011751	179.07193
Com_11850_neg	2-Phenylpropionic acid	C9 H10 O2	150.06866	10.988	--	HMDB0011743	149.06139
Com_30874_pos	gamma-Glutamyltyrosine	C14 H18 N2 O6	310.11638	5.92	--	HMDB0011741	311.1239
Com_79923_pos	gamma-Glutamylglutamic acid	C10 H16 N2 O7	276.09453	1.716	--	HMDB0011737	277.10181
Com_9034_pos	Bicine	C6 H13 N O4	163.08478	1.305	--	HMDB0011727	164.09206
Com_10457_neg	Cinnamoylglycine	C11 H11 N O3	205.07449	8.432	--	HMDB0011621	204.06726
Com_575_pos	All-Trans-13,14-Dihydroretinol	C20 H32 O	288.2456	13.951	--	HMDB0011618	289.2529
Com_418_pos	Monoolein	C21 H40 O4	356.29309	15.109	--	HMDB0011567	357.30023
Com_46_pos	Glycerol 1-hexadecanoate	C19 H38 O4	330.27711	14.15	--	HMDB0011564	331.28436
Com_521_pos	Leucylproline	C11 H20 N2 O3	228.14762	7.056	--	HMDB0011175	229.15477
Com_7124_pos	Gamma-Glu-Leu	C11 H20 N2 O5	260.13727	6.967	--	HMDB0011171	261.14478
Com_5330_pos	gamma-Glutamylleucine	C11 H20 N2 O5	260.1373	7.703	--	HMDB0011171	261.14499
Com_7214_neg	cis-2-Decenoic acid	C10 H18 O2	170.13116	10.491	--	HMDB0010726	169.12392
Com_32653_neg	1,2-Dipalmitoylphosphatidylglycerol	C38 H75 O10 P	722.51234	16.456	--	HMDB0010570	721.5047

Continuation of Supplementary Table 2

Compound_ID	Name	Formula	Molecular Weight	RT [min]	Kegg_ID	HMDB_ID	m/z
Com_508_pos	(+/-)11(12)-EET	C20 H32 O3	302.22463	14.186	--	HMDB0010409	303.23187
Com_60408_pos	1-Palmitoyl-Sn-Glycero-3-Phosphocholine	C24 H50 N O7 P	495.33254	15.158	--	HMDB0010382	496.33981
Com_2966_pos	8,15-Dihete	C20 H32 O4	336.23024	9.571	--	HMDB0010219	337.23752
Com_30451_pos	Nicotinate ribonucleoside	C11 H13 N O6	255.07432	1.396	--	HMDB0006809	256.082
Com_17830_pos	Coenzyme Q2	C19 H26 O4	318.18358	11.852	--	HMDB0006709	319.19086
Com_2686_pos	Proline-hydroxyproline	C10 H16 N2 O4	228.11105	1.406	--	HMDB0006695	229.11813
Com_8759_pos	Heptadecanoic Acid	C17 H34 O2	292.24019	13.383	--	HMDB0006497	293.24747
Com_1306_pos	N-Phenylacetylglutamine	C13 H16 N2 O4	264.11126	7.792	--	HMDB0006344	265.11859
Com_866_neg	16-Hydroxyhexadecanoic acid	C16 H32 O3	226.23028	14.271	--	HMDB0006294	225.22247
Com_20507_pos	4-Hydroxyretinoic Acid	C20 H28 O3	316.20352	14.141	--	HMDB0006254	317.21097
Com_2533_pos	Isotretinoin	C20 H28 O2	300.20909	14.031	--	HMDB0006219	301.21643
Com_35371_pos	Troxerutin	C33 H42 O19	780.18165	15.581	--	HMDB0006083	781.18933
Com_75772_pos	Pregnanetriol	C21 H36 O3	694.51067	16.134	--	HMDB0006070	695.51794
Com_1608_neg	20-Carboxy-Leukotriene B4	C20 H30 O6	366.20584	13.847	--	HMDB0006059	365.19858
Com_33612_pos	2-Hydroxyphenylalanine	C9 H11 N O3	181.07397	6.014	--	HMDB0006050	182.08125
Com_11406_neg	DL-o-Tyrosine	C9 H11 N O3	181.07438	8.045	--	HMDB0006050	180.06718
Com_8681_pos	Mesterolone	C20 H32 O2	304.24045	13.559	--	HMDB0006036	305.24802
Com_18784_pos	11-Oxoetiocholanolone	C19 H28 O3	286.19329	9.341	--	HMDB0006031	287.20065
Com_29991_pos	N-Acetyl-L-glutamine	C7 H12 N2 O4	188.07976	1.38	--	HMDB0006029	189.08693
Com_30382_pos	Mevalonolactone	C6 H10 O3	130.06348	8.029	--	HMDB0006024	131.07089
Com_1773_neg	20-Hydroxy-(5Z,8Z,11Z,14Z)-eicosatetraenoic acid	C20 H32 O3	320.23548	13.582	--	HMDB0005998	319.22821

Continuation of Supplementary Table 2

Compound_ID	Name	Formula	Molecular Weight	RT [min]	Kegg_ID	HMDB_ID	m/z
Com_27995_pos	N4-Acetylcytidine	C11 H15 N3 O6	285.09573	1.386	--	HMDB0005923	286.10312
Com_445_pos	7-hydroxy-3-(4-methoxyphenyl)-4H-chromen-4-one	C16 H12 O4	268.07353	12.158	--	HMDB0005808	269.08081
Com_20846_neg	Thromboxane B3	C20 H32 O6	368.22364	12.84	--	HMDB0005099	367.21649
Com_80902_pos	10-Nitrolinoleate	C18 H31 N O4	307.21466	8.008	--	HMDB0005049	308.22192
Com_66750_pos	Escitalopram	C20 H21 F N2 O	324.16832	7.706	--	HMDB0005038	325.17572
Com_64079_pos	Simvastatin	C25 H38 O5	418.27275	13.118	--	HMDB0005007	419.28003
Com_79724_pos	N2,N2-Dimethylguanosine	C12 H17 N5 O5	311.12282	6.288	--	HMDB0004824	312.1301
Com_9981_neg	4-Hydroxy-3-methylbenzoic acid	C8 H8 O3	152.04775	9.775	--	HMDB0004815	151.04037
Com_11220_neg	2,5-Furandicarboxylic acid	C6 H4 O5	156.00627	1.1	--	HMDB0004812	154.99881
Com_1911_pos	9-Oxo-ODE	C18 H30 O3	294.21964	13.086	--	HMDB0004669	295.22678
Com_777_neg	3b,7b-Dihydroxy-5-androsten-17-one	C19 H28 O3	304.20459	10.396	--	HMDB0004624	303.19733
Com_25230_pos	2'-O-Methyladenosine	C11 H15 N5 O4	281.1123	5.367	--	HMDB0004326	282.11954
Com_1878_pos	Acetyl-N-formyl-5-methoxykynurenamine	C13 H16 N2 O4	264.11548	9.284	--	HMDB0004259	265.12268
Com_3157_pos	DL-Panthenol	C9 H19 N O4	205.13131	1.398	--	HMDB0004231	188.1281
Com_44928_pos	1,4-Dihydro-1-Methyl-4-Oxo-3-Pyridinecarboxamide	C7 H8 N2 O2	152.05871	1.394	--	HMDB0004194	153.06621
Com_1353_pos	2-(Formylamino)Benzoic Acid	C8 H7 N O3	165.04272	9.47	--	HMDB0004089	166.04985
Com_2786_pos	Metanephrine	C10 H15 N O3	197.10533	1.414	--	HMDB0004063	198.11237

Continuation of Supplementary Table 2

Compound_ID	Name	Formula	Molecular Weight	RT [min]	Kegg_ID	HMDB_ID	m/z
Com_23172_pos	DL-Metanephrine	C10 H15 N O3	197.10521	8.404	--	HMDB0004063	198.11272
Com_24500_pos	5,6-dihydroxyindole	C8 H7 N O2	149.04811	8.49	--	HMDB0004058	150.05531
Com_836_pos	13-HPODE	C18 H32 O4	312.23039	13.01	--	HMDB0003871	313.23782
Com_61046_pos	3-Amino-3-(4-hydroxyphenyl)propionic acid	C9 H11 N O3	181.07392	6.438	--	HMDB0003831	182.08156
Com_34378_neg	Protectin D1	C22 H32 O4	396.20579	10.923	--	HMDB0003689	395.19876
Com_8986_pos	4-Acetamidobutyric Acid	C6 H11 N O3	145.07425	1.31	--	HMDB0003681	146.08133
Com_30370_pos	3-Amino-4-methylpentanoic acid	C6 H13 N O2	131.09455	2.054	--	HMDB0003640	132.10182
Com_40191_pos	D-(-)-Salicin	C13 H18 O7	303.13214	2.334	--	HMDB0003546	304.13962
Com_13136_pos	3-(2-Hydroxyethyl)indole	C10 H11 N O	161.08432	7.711	--	HMDB0003447	162.09181
Com_20510_pos	Tryptophanol	C11 H14 N2 O	190.11046	3.563	--	HMDB0003447	191.11774
Com_6110_neg	Boldione	C19 H24 O2	284.17814	11.387	--	HMDB0003422	283.16986
Com_85392_pos	DL-Serine	C3 H7 N O3	105.04308	1.272	--	HMDB0003406	106.05042
Com_24740_neg	2-Phosphoglyceric acid	C3 H7 O7 P	185.9932	1.126	--	HMDB0003391	166.97549
Com_29140_neg	1-Methylguanine	C6 H7 N5 O	165.06547	5.771	--	HMDB0003282	164.05824
Com_18245_pos	Epitestosterone glucuronide	C25 H36 O8	464.24027	11.912	--	HMDB0003193	465.24768
Com_1315_neg	8Z,11Z,14Z-Eicosatrienoic acid	C20 H34 O2	306.25644	14.562	--	HMDB0002925	305.24902
Com_31373_pos	2,3-Dinor-TXB2	C18 H30 O6	342.20265	9.544	--	HMDB0002904	343.20944
Com_3115_pos	6-Keto-prostaglandin f1alpha	C20 H34 O6	370.23595	12.35	--	HMDB0002886	371.24332
Com_8512_neg	Testosterone sulfate	C19 H28 O5 S	368.1663	11.391	--	HMDB0002833	367.15903
Com_7442_neg	Docosatrienoic acid	C22 H38 O2	334.28799	14.987	--	HMDB0002823	333.2807

Continuation of Supplementary Table 2

Compound_ID	Name	Formula	Molecular Weight	RT [min]	Kegg_ID	HMDB_ID	m/z
Com_48394_pos	1-Oleoyl-Sn-Glycero-3-Phosphocholine	C26 H52 N O7 P	521.34613	12.572	--	HMDB0002815	522.35352
Com_34661_pos	Guggulsterone	C21 H28 O2	312.20895	14.116	--	HMDB0002726	313.21622
Com_7042_pos	19-Nortestosterone	C18 H26 O2	274.19349	12.809	--	HMDB0002725	275.2009
Com_873_neg	3,3-Dimethylglutaric acid	C7 H12 O4	160.07398	1.322	--	HMDB0002441	159.06667
Com_13997_pos	Maslinic acid	C30 H48 O4	966.69321	13.394	--	HMDB0002392	967.70032
Com_1271_neg	Tretinoin	C20 H28 O2	300.20956	14.138	--	HMDB0002369	299.20206
Com_30805_pos	Phenylpropiolic acid	C9 H6 O2	146.03713	2.41	--	HMDB0002359	147.0444
Com_1180_pos	N-(5-Aminopentyl)acetamide	C7 H16 N2 O	144.12657	1.895	--	HMDB0002284	145.1339
Com_5566_neg	2-Hydroxymyristic acid	C14 H28 O3	244.20417	12.167	--	HMDB0002261	243.19684
Com_6496_pos	cis-gondoic acid	C20 H38 O2	310.28727	14.794	--	HMDB0002231	311.29443
Com_16705_pos	2-Phenylglycine	C8 H9 N O2	151.06361	2.116	--	HMDB0002210	152.07101
Com_56393_pos	(S)-Equol	C15 H14 O3	242.09436	11.079	--	HMDB0002209	243.10173
Com_10207_neg	3-Hydroxydecanoic acid	C10 H20 O3	188.14168	11.782	--	HMDB0002203	187.13432
Com_42203_neg	(S)-beta-Aminoisobutyric Acid	C4 H9 N O2	103.0636	6.292	--	HMDB0002166	102.05632
Com_6229_neg	1,3-Dimethyluracil	C6 H8 N2 O2	140.05908	1.389	--	HMDB0002144	139.0518
Com_911_pos	Palmitoyl ethanolamide	C18 H37 N O2	299.28264	14.835	--	HMDB0002100	300.2897
Com_17276_neg	3-Methoxyphenylacetic acid	C9 H10 O3	166.06342	10.254	--	HMDB0002072	165.05612
Com_13835_neg	4-Hydroxy-2-Oxoglutaric Acid	C5 H6 O6	162.01686	1.087	--	HMDB0002070	207.01501
Com_31746_pos	5-Phenylvaleric Acid	C11 H14 O2	178.0995	10.639	--	HMDB0002043	179.10658
Com_47450_pos	4-Methoxycinnamic Acid	C10 H10 O3	178.06287	11.832	--	HMDB0002040	179.07014
Com_2890_pos	Ureidoisobutyric Acid	C5 H10 N2 O3	146.06947	1.314	--	HMDB0002031	147.07664
Com_17774_pos	Coenzyme Q1	C14 H18 O4	250.12064	15.306	--	HMDB0002012	251.1281

Continuation of Supplementary Table 2

Compound_ID	Name	Formula	Molecular Weight	RT [min]	Kegg_ID	HMDB_ID	m/z
Com_12756_pos	L-Methionine sulfoxide	C5 H11 N O3 S	165.04617	1.309	--	HMDB0002005	166.05351
Com_56_neg	cis-5,8,11,14,17-Eicosapentaenoic acid	C20 H30 O2	302.22491	14.595	--	HMDB0001999	301.21756
Com_41563_neg	2-Hydroxy-2-methylbutanoic acid	C5 H10 O3	118.06344	7.812	--	HMDB0001987	117.05616
Com_55780_pos	Dextromethorphan hydrobromide	C18 H25 N O	271.18954	9.66	--	HMDB0001920	272.19696
Com_203_neg	19(R)-Hydroxy-prostaglandin E2	C20 H32 O6	350.20979	11.859	--	HMDB0001908	349.20251
Com_8210_pos	5-Methoxysalicylic acid	C8 H8 O4	168.04249	6.277	--	HMDB0001868	169.04959
Com_26584_pos	P-Aminohippuric Acid	C9 H10 N2 O3	194.06902	1.386	--	HMDB0001867	195.07675
Com_5195_neg	2-Ketohexanoic acid	C6 H10 O3	130.06337	2.491	--	HMDB0001864	129.05605
Com_4801_neg	2-Hydroxyvaleric acid	C5 H10 O3	118.06343	1.408	--	HMDB0001863	117.05611
Com_55867_pos	1,3-Dimethyluric Acid	C7 H8 N4 O3	196.05977	6.767	--	HMDB0001857	197.06715
Com_92287_pos	5-Hydroxytryptophol	C10 H11 N O2	177.07939	8.224	--	HMDB0001855	178.08667
Com_1433_neg	Methylsuccinic acid	C5 H8 O4	132.04267	1.138	--	HMDB0001844	131.03554
Com_14913_neg	3-Coumaric acid	C9 H8 O3	164.04779	11.799	--	HMDB0001713	163.04048
Com_5442_neg	2-Hydroxycaproic acid	C6 H12 O3	132.07905	3.715	--	HMDB0001624	131.07178
Com_17394_pos	Glucose 1-phosphate	C6 H13 O9 P	260.0295	1.337	--	HMDB0001586	261.03671
Com_1786_neg	Thymidine 3',5'-cyclic monophosphate	C10 H13 N2 O7 P	304.0437	1.073	--	HMDB0001570	349.04202
Com_59125_pos	1-Methylguanosine	C11 H15 N5 O5	297.10711	5.355	--	HMDB0001563	298.11407
Com_5315_neg	D-Ribose-1-phosphate	C5 H11 O8 P	230.01949	1.183	--	HMDB0001489	229.0121

Continuation of Supplementary Table 2

Compound_ID	Name	Formula	Molecular Weight	RT [min]	Kegg_ID	HMDB_ID	m/z
Com_38290_pos	3-Hydroxyanthranilic Acid	C7 H7 N O3	153.04269	1.383	--	HMDB0001476	154.05031
Com_9578_neg	(+)-alpha-Lipoic acid	C8 H14 O2 S2	206.04314	1.358	--	HMDB0001451	205.03564
Com_55506_neg	Guanosine-5'-monophosphate	C10 H14 N5 O8 P	363.05888	2.313	--	HMDB0001397	362.05136
Com_10548_pos	Diaminopimelic acid	C7 H14 N2 O4	190.09531	1.357	--	HMDB0001370	191.10272
Com_51272_pos	Trimethyllysine	C9 H21 N2 O2	189.15986	13.377	--	HMDB0001325	190.16695
Com_42153_neg	Thymidine 5'-diphosphate	C10 H16 N2 O11 P2	402.02321	1.056	--	HMDB0001274	401.01593
Com_46598_pos	5,6-Dihydroxyindole-2-Carboxylic Acid	C9 H7 N O4	193.03752	6.914	--	HMDB0001253	194.04448
Com_19915_pos	S-Adenosyl-methionine	C22 H30 N6 O8 S2	570.15398	13.406	--	HMDB0001185	571.16125
Com_18547_pos	5'-Deoxy-5'-(Methylthio)Adenosine	C11 H15 N5 O3 S	297.0897	6.957	--	HMDB0001173	298.09698
Com_4064_pos	N-Acetyl-DL-glutamic acid	C7 H11 N O5	189.064	2.288	--	HMDB0001138	190.07137
Com_6052_pos	N-Acetylglutamic acid	C7 H11 N O5	189.06395	1.882	--	HMDB0001138	190.07137
Com_1082_neg	N-Acetylmannosamine	C8 H15 N O6	221.09045	1.375	--	HMDB0001129	220.08315
Com_27835_pos	Cotinine	C10 H12 N2 O	176.095	2.399	--	HMDB0001046	177.10228
Com_753_pos	L-Threonic acid-1,4-lactone	C4 H6 O4	118.02824	1.656	--	HMDB0000940	119.03547
Com_120_neg	Tridecylic acid	C13 H26 O2	214.19366	13.844	--	HMDB0000910	213.18631
Com_46897_pos	2'-Deoxyadenosine-5'-monophosphate	C10 H14 N5 O6 P	331.06709	1.465	--	HMDB0000905	332.07523
Com_61011_pos	Taurodeoxycholic acid sodium salt	C26 H44 N Na O6 S	521.27928	10.916	--	HMDB0000896	522.28656
Com_46944_pos	Undecanedioic acid	C11 H20 O4	216.13681	7.821	--	HMDB0000888	217.14401
Com_78178_pos	5-Methyluridine	C10 H14 N2 O6	258.0851	7.786	--	HMDB0000884	259.09238
Com_757_neg	Tetradecanedioic acid	C14 H26 O4	258.18361	11.038	--	HMDB0000872	257.1763

Continuation of Supplementary Table 2

Compound_ID	Name	Formula	Molecular Weight	RT [min]	Kegg_ID	HMDB_ID	m/z
Com_1535_pos	N-Acetyl-L-tyrosine	C11 H13 N O4	223.08459	8.199	--	HMDB0000866	224.09178
Com_11140_neg	2-Hydroxyhippuric acid	C9 H9 N O4	195.05368	6.737	--	HMDB0000840	194.0464
Com_9854_neg	N-Glycolylneuraminic acid	C11 H19 N O10	279.09604	1.207	--	HMDB0000833	278.08865
Com_2365_neg	Capryloylglycine	C10 H19 N O3	201.13707	9.041	--	HMDB0000832	200.12982
Com_53219_pos	Propionylcarnitine	C10 H19 N O4	217.13184	3.066	--	HMDB0000824	218.13911
Com_4573_pos	Propionyl-L-carnitine	C10 H19 N O4	217.13174	7.546	--	HMDB0000824	218.13907
Com_2820_neg	N-Acetylaspartic acid	C6 H9 N O5	175.04842	1.156	--	HMDB0000812	174.04109
Com_45215_neg	3-Phosphoglyceric acid	C3 H8 Na O6 P	193.99599	1.21	--	HMDB0000807	192.989
Com_30927_neg	N-Propionylglycine	C5 H9 N O3	131.0586	1.159	--	HMDB0000783	130.0513
Com_25123_neg	N-Acetyl-D-galactosamine 4-sulfate	C8 H15 N O9 S	301.04738	1.257	--	HMDB0000781	300.0401
Com_15016_neg	N-Acetylalanine	C5 H9 N O3	131.05863	1.393	--	HMDB0000766	130.05136
Com_15270_neg	D-(-)-Mannitol	C6 H14 O6	182.07768	1.853	--	HMDB0000765	181.0704
Com_70_neg	Hydrocinnamic acid	C9 H10 O2	150.06815	7.994	--	HMDB0000764	149.06076
Com_255_neg	5-Hydroxyindole-3-acetic acid	C10 H9 N O3	191.05872	5.928	--	HMDB0000763	190.05141
Com_1880_neg	DL-Malic acid	C4 H6 O5	134.02189	1.157	--	HMDB0000744	133.01454
Com_76333_pos	3-Indoleacrylic acid	C11 H9 N O2	187.06331	9.476	--	HMDB0000734	188.07059
Com_11207_pos	Glycylproline	C7 H12 N2 O3	172.08495	1.389	--	HMDB0000721	173.0921
Com_2818_neg	Hexadecanedioic acid	C16 H30 O4	286.21497	12.209	--	HMDB0000712	285.20755
Com_2362_neg	Glycoursodeoxycholic acid	C26 H43 N O5	449.3151	12.821	--	HMDB0000708	448.30774
Com_12306_neg	4-Hydroxyphenylpyruvic acid	C9 H8 O4	180.04269	5.969	--	HMDB0000707	179.03545
Com_33015_neg	hydroxyphenylpyruvate	C9 H7 O4	179.03494	5.257	--	HMDB0000707	178.02766
Com_66926_pos	Hexanoylcarnitine	C13 H25 N O4	259.17828	8.241	--	HMDB0000705	260.18561
Com_3818_neg	Hexanoylglycine	C8 H15 N O3	173.10566	6.289	--	HMDB0000701	172.09837

Continuation of Supplementary Table 2

Compound_ID	Name	Formula	Molecular Weight	RT [min]	Kegg_ID	HMDB_ID	m/z
Com_28484_pos	L-(-)-Methionine	C5 H11 N O2 S	189.04268	11.71	--	HMDB0000696	190.04959
Com_9107_neg	4-Methylvaleric Acid	C6 H12 O2	116.08399	7.656	--	HMDB0000689	115.07678
Com_54461_pos	Indoxylsulfuric acid	C8 H7 N O4 S	213.00956	6.613	--	HMDB0000682	214.01688
Com_48107_neg	3-Indoxyl sulphate	C8 H7 N O4 S	213.01015	6.959	--	HMDB0000682	212.00287
Com_34220_pos	Indole-3-lactic acid	C11 H11 N O3	205.07383	9.147	--	HMDB0000671	206.08104
Com_44201_neg	2-Hydroxyisocaproic Acid	C6 H12 O3	132.07905	7.506	--	HMDB0000665	131.07178
Com_1432_neg	cholesteryl sulfate	C27 H46 O4 S	466.31275	15.278	--	HMDB0000653	465.30539
Com_19235_pos	Decanoylcarnitine	C17 H33 N O4	315.2412	10.479	--	HMDB0000651	316.24863
Com_6517_pos	Epitestosterone	C19 H28 O2	288.20893	13.083	--	HMDB0000628	289.21628
Com_37573_neg	Gluconic acid	C6 H12 O7	196.05986	4.906	--	HMDB0000625	195.05258
Com_36080_pos	Docosanamide	C22 H45 N O	339.35031	16.53	--	HMDB0000583	340.358
Com_5062_neg	D-(+)-Arabitol	C5 H12 O5	152.06894	1.319	--	HMDB0000568	151.0616
Com_15488_pos	D-3-Phenyllactic acid	C9 H10 O3	166.06315	8.789	--	HMDB0000563	355.11554
Com_2051_neg	L-(-)-3-Phenyllactic acid	C9 H10 O3	166.06343	5.948	--	HMDB0000563	165.05623
Com_14735_neg	(3-Methoxy-4-hydroxyphenyl)ethylene glycol sulfate	C9 H12 O7 S	264.03091	5.253	--	HMDB0000559	263.02365
Com_2421_pos	3-Methyladipic acid	C7 H12 O4	160.07394	8.212	--	HMDB0000555	161.08119
Com_8612_pos	4-Phenylbutyric acid	C10 H12 O2	164.08407	15.346	--	HMDB0000543	165.09145
Com_42722_neg	N-Acetylglycine	C4 H7 N O3	117.043	1.219	--	HMDB0000532	116.03561
Com_6973_neg	3-Hydroxyvaleric acid	C5 H10 O3	118.06335	1.701	--	HMDB0000531	117.05607
Com_83465_pos	L-arginine	C6 H14 N4 O2	174.1118	8.742	--	HMDB0000517	175.11908
Com_6557_neg	Capric acid	C10 H20 O2	172.14685	14.534	--	HMDB0000511	171.13959
Com_1599_pos	7-Ketocholesterol	C27 H44 O2	400.33458	14.192	--	HMDB0000501	401.34201

Continuation of Supplementary Table 2

Compound_ID	Name	Formula	Molecular Weight	RT [min]	Kegg_ID	HMDB_ID	m/z
Com_11413_pos	L-5-Hydroxytryptophan	C11 H12 N2 O3	220.085	5.842	--	HMDB0000472	221.09232
Com_22_pos	7-Ketolithocholic acid	C24 H38 O4	390.27719	13.253	--	HMDB0000467	391.28452
Com_90784_pos	5-Hydroxylysine	C6 H15 Cl N2 O3	198.07708	1.228	--	HMDB0000450	199.08467
Com_37780_neg	2-Furoylglycine	C7 H7 N O4	169.03799	5.5	--	HMDB0000439	168.0307
Com_7746_pos	3-(3,4-dihydroxyphenyl)propanoic acid	C9 H10 O4	164.04766	8.206	--	HMDB0000423	165.05511
Com_1634_neg	2-Methylpentanedioic acid	C6 H10 O4	146.05834	1.345	--	HMDB0000422	145.05112
Com_26885_pos	2-Methoxyestradiol	C19 H26 O3	302.18848	14.443	--	HMDB0000405	303.19589
Com_31402_neg	2-Isopropylmalate	C7 H12 O5	176.0688	1.948	--	HMDB0000402	175.06152
Com_1442_neg	trans-2-Butene-1,4-dicarboxylic Acid	C6 H8 O4	144.04273	1.087	--	HMDB0000393	143.03539
Com_960_pos	7-Ketodeoxycholic acid	C24 H38 O5	406.27195	12.973	--	HMDB0000391	407.27942
Com_10523_pos	Allolithocholic acid	C24 H40 O3	376.29783	12.796	--	HMDB0000381	377.30515
Com_11009_pos	2-Methylbutyroylcarnitine	C12 H23 N O4	245.16272	9.614	--	HMDB0000378	246.1705
Com_545_neg	Beta-Muricholic acid	C24 H40 O5	408.28754	13.883	--	HMDB0000364	407.27966
Com_15879_neg	3-Hydroxybutyric acid	C4 H8 O3	104.04774	10.004	--	HMDB0000357	103.04046
Com_8626_neg	3-Hydroxysebacic acid	C10 H18 O5	218.11587	2.661	--	HMDB0000350	217.10864
Com_34332_pos	Isohomovanillic acid	C9 H10 O4	182.05811	7.432	--	HMDB0000333	183.06525
Com_33964_neg	Uridine 5'-Diphospho-N-Acetylgalactosamine	C17 H27 N3 O17 P2	607.08296	1.377	--	HMDB0000304	606.07568
Com_106084_pos	2,6-Dihydroxypurine	C5 H4 N4 O2	152.03391	3.916	--	HMDB0000292	153.04118
Com_2196_pos	N-acetyl-D-glucosamine	C8 H15 N O6	221.08961	1.357	--	HMDB0000215	222.09669
Com_35323_pos	Acetyl-L-carnitine	C9 H17 N O4	203.11568	1.78	--	HMDB0000201	204.12294
Com_75671_pos	2-(1H-indol-3-yl)acetic acid	C10 H9 N O2	175.0636	7.241	--	HMDB0000197	176.07088
Com_4247_neg	L-lysine	C6 H14 N2 O2	146.10597	1.654	--	HMDB0000182	145.09868

Continuation of Supplementary Table 2

Compound_ID	Name	Formula	Molecular Weight	RT [min]	Kegg_ID	HMDB_ID	m/z
Com_10588_pos	Threonine	C4 H9 N O3	119.05853	1.3	--	HMDB0000167	120.06583
Com_2145_pos	D-(+)-Maltose	C12 H22 O11	364.09825	1.333	--	HMDB0000163	365.10608
Com_57867_pos	Glutamic acid	C5 H9 N O4	147.05361	6.435	--	HMDB0000148	148.06079
Com_38608_pos	Choline bitartrate	C9 H19 N O7	253.11719	1.451	--	HMDB0000097	254.12436
Com_3830_neg	2'-Deoxyinosine	C10 H12 N4 O4	252.08648	4.164	--	HMDB0000071	251.07927
Com_53807_pos	Epinephrine bitartrate	C13 H19 N O9	333.10525	1.388	--	HMDB0000068	334.11237
Com_35627_pos	Epinephrine	C9 H13 N O3	183.0897	7.709	--	HMDB0000068	184.09698
Com_27544_neg	Argininosuccinic acid	C10 H18 N4 O6	254.15086	10.121	--	HMDB0000052	289.12024
Com_31771_neg	3-Ureidopropionic acid	C4 H8 N2 O3	132.05394	1.241	--	HMDB0000026	131.04666
Com_592_pos	D-(+)-Proline	C5 H9 N O2	115.06354	1.365	--	--	116.07072
Com_1301_pos	2-Amino-1,3-octadecanediol	C18 H39 N O2	301.2983	13.393	--	--	302.30557
Com_1414_pos	L(-)-Carnitine	C7 H15 N O3	161.10535	1.296	--	--	162.11247
Com_1795_pos	L-(+)-Citrulline	C6 H13 N3 O3	175.09584	1.298	--	--	176.10298
Com_4960_pos	DL-Lysine	C6 H14 N2 O2	146.10578	1.127	--	--	147.11304
Com_6463_pos	Ethyl paraben	C9 H10 O3	166.06334	7.485	--	--	167.07054
Com_7767_pos	Quercetin-3β-D-glucoside	C21 H20 O12	464.09617	9.283	--	--	465.10318
Com_10675_pos	N,N-Bis(2-hydroxyethyl)formamide	C5 H11 N O3	133.07416	1.304	--	--	134.08142
Com_12656_pos	Di(2-ethylhexyl) phthalate	C24 H38 O4	390.27713	15.315	--	--	391.28461
Com_14308_pos	17α-Ethynylestradiol	C20 H24 O2	296.17761	12.131	--	--	297.18491
Com_15916_pos	N1-tetrahydrofuran-2-ylmethyl-2-cyanoacetamide	C8 H12 N2 O2	168.0902	5.672	--	--	169.09752
Com_16078_pos	Cytidine 5'-monophosphate (hydrate)	C9 H14 N3 O8 P	323.05151	1.377	--	--	324.05902
Com_20598_pos	(R)-Equol	C15 H14 O3	242.09434	10.752	--	--	243.10173

Continuation of Supplementary Table 2

Compound_ID	Name	Formula	Molecular Weight	RT [min]	Kegg_ID	HMDB_ID	m/z
Com_24359_pos	3-(3-methylbut-2-en-1-yl)-3H-purin-6-amine	C10 H13 N5	203.11713	9.101	--	--	204.12471
Com_37205_pos	4-Methylumbelliferone hydrate	C10 H8 O3	176.04755	7.823	--	--	177.05486
Com_53945_pos	2-Isopropylaniline	C9 H13 N	135.10528	7.883	--	--	136.11261
Com_77157_pos	DL-Arginine	C6 H14 N4 O2	174.11173	1.899	--	--	175.119
Com_1405_pos	Glycerophospho-N-palmitoyl ethanolamine	C21 H44 N O7 P	453.28591	14.699	--	--	454.29361
Com_4589_pos	3'-Adenosine monophosphate (3'-AMP)	C10 H14 N5 O7 P	347.06274	2.01	--	--	348.07034
Com_9741_pos	Sorbic acid	C6 H8 O2	112.05265	6.883	--	--	113.05994
Com_19416_pos	Adenosine 3'5'-cyclic monophosphate	C10 H12 N5 O6 P	329.05226	2.133	--	--	330.05945
Com_34777_pos	α-Aspartylphenylalanine	C13 H16 N2 O5	280.10605	7.155	--	--	281.11346
Com_12_pos	D-(+)-Pipecolinic acid	C6 H11 N O2	112.05245	1.411	--	--	130.08627
Com_13_pos	Oleoyl ethylamide	C20 H39 N O	309.30324	15.484	--	--	310.31052
Com_58_pos	Tanshinone IIA	C19 H18 O3	294.12604	14.011	--	--	295.13309
Com_218_pos	1H-indene-3-carboxamide	C10 H9 N O	159.06855	4.449	--	--	160.07576
Com_226_pos	AL 8810 Methyl ester	C25 H33 F O4	438.22353	11.134	--	--	439.23022
Com_235_pos	2-Linoleoyl glycerol	C21 H38 O4	336.26414	14.773	--	--	337.27124
Com_310_pos	PC (16:2e/2:0)	C26 H50 N O7 P	519.3337	14.436	--	--	520.34088
Com_413_pos	Prostaglandin E2-1-glyceryl ester	C23 H38 O7	408.24948	12.878	--	--	409.25681
Com_419_pos	3-(4-methoxyanilino)-1-(4-methoxyphenyl)prop-2-ene-1-thione	C17 H17 N O2 S	299.09824	11.915	--	--	300.10547
Com_442_pos	7-(2-hydroxypropan-2-yl)-1,4a-dimethyl-decahydronaphthalen-1-ol	C15 H28 O2	222.19864	14.184	--	--	223.20592
Com_462_pos	3-Acetoxyurs-12-en-23-oic acid	C32 H50 O4	498.36902	13.953	--	--	499.37628

Continuation of Supplementary Table 2

Compound_ID	Name	Formula	Molecular Weight	RT [min]	Kegg_ID	HMDB_ID	m/z
Com_483_pos	(2R)-5-hydroxy-7-methoxy-2-phenyl-3,4-dihydro-2H-1-benzopyran-4-one	C16 H14 O4	270.08949	12.783	--	--	271.09677
Com_623_pos	DL-Stachydrine	C7 H13 N O2	143.09489	1.384	--	--	144.10205
Com_709_pos	2-Oxindole	C8 H7 N O	133.05287	8.929	--	--	134.06012
Com_914_pos	PC (16:1e/2:0)	C26 H52 N O7 P	499.36717	14.756	--	--	500.37469
Com_964_pos	methyl 6-{[4-(trifluoromethyl)anilino]carbonyl}nicotinate	C15 H11 F3 N2 O3	324.07505	12.563	--	--	325.08243
Com_1018_pos	tetranor-12(R)-HETE	C16 H26 O3	248.17782	12.156	--	--	249.18483
Com_1045_pos	5-[(10Z)-14-(3,5-dihydroxyphenyl)tetradec-10-en-1-yl]benzene-1,3-diol	C26 H36 O4	412.25924	13.66	--	--	413.26651
Com_1133_pos	N-Tetradecanamide	C14 H29 N O	227.22511	14.376	--	--	228.23238
Com_1166_pos	2-[(3S)-1-(4-Chlorobenzyl)-3-pyrrolidinyl]-1-methyl-1H-benzimidazole	C19 H20 Cl N3	325.13188	7.04	--	--	326.13895
Com_1218_pos	N-(5-acetamidopentyl)acetamide	C9 H18 N2 O2	186.1372	6.601	--	--	187.14417
Com_1316_pos	5,7-dihydroxy-6-methoxy-2-phenyl-3,4-dihydro-2H-1-benzopyran-4-one	C16 H14 O5	286.08417	11.418	--	--	287.09131
Com_1330_pos	LDGTS 16:3	C26 H45 N O6	467.32689	13.633	--	--	468.33441
Com_1332_pos	1-oxo-2,3-dihydro-1H-inden-4-yl benzoate	C16 H12 O3	252.07869	10.654	--	--	253.08591
Com_1450_pos	Ciprostene	C22 H36 O4	346.25115	14.385	--	--	347.25854
Com_1485_pos	1,7,8-trihydroxy-3-methyl-1,2,3,4,7,12-hexahydrotetraphen-12-one	C19 H18 O4	310.12071	12.384	--	--	311.12793
Com_1517_pos	9-Oxo-10(E),12(E)-octadecadienoic acid	C18 H30 O3	294.21797	13.798	--	--	295.22522
Com_1589_pos	5-phenyl-3-(phenylmethylene)furan-2(3H)-one	C17 H12 O2	248.08388	9.598	--	--	249.09126

Continuation of Supplementary Table 2

Compound_ID	Name	Formula	Molecular Weight	RT [min]	Kegg_ID	HMDB_ID	m/z
Com_1693_pos	8,8-dimethyl-2H,8H-pyrano[3,2-g]chromen-2-one	C14 H12 O3	246.08952	12.914	--	--	247.09676
Com_1764_pos	11-Deoxy prostaglandin F1α	C20 H36 O4	362.24355	14.56	--	--	363.25067
Com_1807_pos	ethyl 4-[(6-methyl-3-pyridazinyl)oxy]benzoate	C14 H14 N2 O3	258.10109	7.337	--	--	259.10815
Com_2001_pos	ACar 18:1	C25 H48 N O4	425.35085	13.671	--	--	426.35791
Com_2153_pos	LDGTS 18:2	C28 H51 N O6	497.37277	14.406	--	--	498.38013
Com_2212_pos	ethyl 5-[(2,1,3-benzoxadiazol-4-ylsulfonyl)amino]-2-piperidinobenzoate	C20 H22 N4 O5 S	452.11166	11.32	--	--	453.11935
Com_2564_pos	PC (18:2e/2:0)	C28 H54 N O7 P	547.36396	14.994	--	--	548.37103
Com_2592_pos	(3beta,9xi)-3-(beta-D-Glucopyranosyloxy)-14-hydroxycard-20(22)-enolide	C29 H44 O9	536.29728	14.161	--	--	537.30487
Com_2665_pos	5α-Pregnan-3,20-dione	C21 H32 O2	316.24046	11.229	--	--	317.2478
Com_2752_pos	1,1-Dimethyl-2-oxopropyl N-[2-(2-pyridyl)ethyl]carbamate	C13 H18 N2 O3	250.13159	7.141	--	--	251.13869
Com_2866_pos	IPK	C17 H32 N4 O4	338.23196	9.582	--	--	339.23947
Com_2892_pos	D-(+)-Camphor	C10 H16 O	152.12033	14.346	--	--	153.12791
Com_3069_pos	tert-Butyl N-[1-(aminocarbonyl)-3-methylbutyl]carbamate	C11 H22 N2 O3	230.16321	6.53	--	--	231.17046
Com_3155_pos	Ethyl 3-acetyl-4-oxopentanoate	C9 H14 O4	186.08965	9.185	--	--	187.09683
Com_3316_pos	5,7-dihydroxy-3,8-dimethoxy-2-phenyl-4H-chromen-4-one	C17 H14 O6	314.07923	10.23	--	--	315.08673
Com_3334_pos	D-Sphingosine	C18 H37 N O2	299.28241	13.309	--	--	300.28958
Com_3432_pos	4-(Diethylamino)salicylaldehyde	C11 H15 N O2	193.1108	2.19	--	--	194.11813

Continuation of Supplementary Table 2

Compound_ID	Name	Formula	Molecular Weight	RT [min]	Kegg_ID	HMDB_ID	m/z
Com_3635_pos	1,2-dihydroxyheptadec-16-yn-4-yl acetate	C19 H34 O4	348.22834	13.817	--	--	349.23575
Com_3702_pos	delta9-THC-d3	C21 H27 [2]H3 O2	335.2545	14.205	--	--	336.26175
Com_3757_pos	Lysopc 18:2	C26 H50 N O7 P	519.33427	14.792	--	--	520.34137
Com_3787_pos	LysoPE 18:0	C23 H48 N O7 P	481.31789	14.419	--	--	482.3251
Com_3827_pos	LPE 18:2	C23 H44 N O7 P	477.28654	14.396	--	--	478.29385
Com_3866_pos	FMH	C20 H27 N5 O4 S	433.18281	4.924	--	--	434.18997
Com_3936_pos	3-amino-2-phenyl-2H-pyrazolo[4,3-c]pyridine-4,6-diol	C12 H10 N4 O2	242.08067	10.074	--	--	243.08809
Com_3959_pos	4-methyl-6-phenyl-5,6-dihydro-2H-pyran-2-one	C12 H12 O2	188.08401	8.232	--	--	189.09132
Com_4068_pos	(±)8-HEPE	C20 H30 O3	300.20921	15.143	--	--	301.21619
Com_4071_pos	RNK	C16 H32 N8 O5	398.23753	11.984	--	--	399.24478
Com_4256_pos	11(Z),14(Z),17(Z)-Eicosatrienoic acid	C20 H34 O2	306.2538	14.377	--	--	307.26102
Com_4308_pos	RMH	C17 H30 N8 O4 S	464.1931	9.832	--	--	465.20038
Com_4400_pos	Tetranor-12R-HETE	C16 H26 O3	248.17771	14.465	--	--	249.185
Com_4566_pos	4-(2,3-dihydro-1,4-benzodioxin-6-yl)butanoic acid	C12 H14 O4	204.07881	9.59	--	--	205.08629
Com_4794_pos	N-[2-chloro-6-(trifluoromethoxy)phenyl]-2,2-dimethylpropanamide	C12 H13 Cl F3 N O2	295.05914	1.258	--	--	296.06628
Com_4912_pos	N-isopropyl-N'-(2-oxoazepan-3-yl)urea	C10 H19 N3 O2	213.14801	7.589	--	--	214.15521
Com_5001_pos	7-(1-pyrrolidinyl)pyrimido[4,5-d]pyrimidin-4-amine	C10 H12 N6	216.11148	1.82	--	--	217.11876
Com_5032_pos	2-[(3S)-1-(2-Methylbenzyl)-3-pyrrolidinyl]-1,3-benzothiazole	C19 H20 N2 S	308.13779	3.068	--	--	309.14508

Continuation of Supplementary Table 2

Compound_ID	Name	Formula	Molecular Weight	RT [min]	Kegg_ID	HMDB_ID	m/z
Com_5094_pos	Sphingosine (d18:1)	C18 H37 N O2	321.2647	14.826	--	--	322.27197
Com_5095_pos	N-(2-oxo-3-azepanyl)benzenesulfonamide	C12 H16 N2 O3 S	268.09175	7.778	--	--	269.099
Com_5194_pos	1-[4-hydroxy-3-(3-methylbut-2-en-1-yl)phenyl]ethan-1-one	C13 H16 O2	204.11528	14.356	--	--	205.12285
Com_5325_pos	(3R)-8-hydroxy-3-(4-hydroxyphenyl)-3,4-dihydro-1H-2-benzopyran-1-one	C15 H12 O4	256.07387	9.866	--	--	257.08136
Com_5352_pos	2-(3,4-dihydroxyphenyl)acetamide	C8 H9 N O3	167.05854	6.377	--	--	168.06593
Com_5457_pos	QNK	C15 H28 N6 O6	370.19674	10.698	--	--	371.20398
Com_5541_pos	QPH	C16 H24 N6 O5	380.18364	11.595	--	--	381.19067
Com_5696_pos	8-Isoprostaglandin F1α	C20 H36 O5	338.24369	13.53	--	--	339.25101
Com_5720_pos	(2E)-3-phenyl-N-(2-phenylethyl)prop-2-enamide	C17 H17 N O	251.13099	12.094	--	--	252.13838
Com_5868_pos	3-hydroxy-3,4-bis[(4-hydroxy-3-methoxyphenyl)methyl]oxolan-2-one	C20 H22 O7	356.1263	9.026	--	--	357.13382
Com_5912_pos	6-benzyl-4-oxo-1,4-dihydropyridine-3-carboxamide	C13 H12 N2 O2	250.07445	12.314	--	--	251.08173
Com_5973_pos	1,4-dihydroxyheptadec-16-en-2-yl acetate	C19 H36 O4	328.26105	14.525	--	--	329.26889
Com_5989_pos	octadec-9-ynoic acid	C18 H32 O2	262.22983	14.464	--	--	263.23715
Com_6003_pos	LDGTS 18:4	C28 H47 N O6	493.34126	13.893	--	--	494.34818
Com_6163_pos	N1-(2,3-dihydro-1,4-benzodioxin-2-ylmethyl)-2,2-dimethylpropanamide	C14 H19 N O3	249.13574	5.258	--	--	250.14302
Com_6164_pos	3-(propan-2-yl)-octahydropyrrolo[1,2-a]pyrazine-1,4-dione	C10 H16 N2 O2	196.12153	7.701	--	--	197.12906

Continuation of Supplementary Table 2

Compound_ID	Name	Formula	Molecular Weight	RT [min]	Kegg_ID	HMDB_ID	m/z
Com_6184_pos	4-(tert-butyl)phenyl 3,5-dimethylisoxazole-4-carboxylate	C16 H19 N O3	273.13676	7.526	--	--	274.1442
Com_6210_pos	1-(4-hydroxyphenyl)-2-phenylethan-1-one	C14 H12 O2	212.08367	8.336	--	--	213.09109
Com_6348_pos	DPK	C15 H26 N4 O6	179.09353	11.699	--	--	359.19434
Com_6423_pos	2-(2-acetyl-3,5-dihydroxyphenyl)acetic acid	C10 H10 O5	192.04243	14.777	--	--	193.0498
Com_6492_pos	2-(tert-butyl)-6,7-dimethoxy-4H-3,1-benzoxazin-4-one	C14 H17 N O4	263.11636	6.504	--	--	264.12396
Com_6687_pos	4-(anilinomethylidene)-3-methyl-4,5-dihydroisoxazol-5-one	C11 H10 N2 O2	202.07471	8.833	--	--	203.082
Com_6722_pos	1-(3,4-dimethoxyphenyl)ethan-1-one oxime	C10 H13 N O3	177.07911	10.077	--	--	178.08618
Com_6869_pos	ethyl 2-{[(hexylamino)carbonyl]amino}-3-phenylpropanoate	C18 H28 N2 O3	320.21047	8.653	--	--	321.2178
Com_6883_pos	15-Deoxy-Δ12,14-prostaglandin A1	C20 H30 O3	318.21949	12.803	--	--	319.22702
Com_6997_pos	1-(4-nitrophenyl)piperidine	C11 H14 N2 O2	206.10542	1.367	--	--	207.1127
Com_7025_pos	3-[(2-phenyl-1H-imidazol-4-yl)methylene]-1,3-dihydro-2H-indol-2-one	C18 H13 N3 O	287.10031	1.38	--	--	288.1077
Com_7100_pos	(11E,15Z)-9,10,13-trihydroxyoctadeca-11,15-dienoic acid	C18 H32 O5	350.20782	11.832	--	--	351.21463
Com_7112_pos	3-(4-hydroxy-3-methoxyphenyl)propanoic acid	C10 H12 O4	196.07377	8.623	--	--	197.08087
Com_7214_pos	6,6-dimethyl-4-piperidino-5,6-dihydro-2H-thiine-2-thione	C12 H19 N S2	241.09483	1.404	--	--	242.10193
Com_7246_pos	ethyl 5-hydroxy-4-oxo-4H-chromene-2-carboxylate	C12 H10 O5	234.05317	9.353	--	--	235.06049

Continuation of Supplementary Table 2

Compound_ID	Name	Formula	Molecular Weight	RT [min]	Kegg_ID	HMDB_ID	m/z
Com_7252_pos	2-oxa-4-azatetracyclo[6.3.1.1~6,10~.0~1,5~]tridecan-3-one	C11 H15 N O2	193.11073	6.4	--	--	194.11795
Com_7304_pos	indoline-2-carboxylic acid	C9 H9 N O2	163.0635	8.197	--	--	164.07036
Com_7319_pos	15-deoxy-Δ12,14-Prostaglandin J2-biotin	C35 H54 N4 O4 S	305.20501	12.913	--	--	649.37317
Com_7355_pos	19(R)-Hydroxy prostaglandin E1	C20 H34 O6	352.22472	12.617	--	--	353.23227
Com_7416_pos	Testosterone undecanoate	C30 H48 O3	439.33426	15.11	--	--	440.34344
Com_7434_pos	Lysopg 18:1	C24 H47 O9 P	510.29646	12.464	--	--	511.30374
Com_7439_pos	1-Methyl-2-[(3S)-1-(2-methylbenzyl)-3-pyrrolidinyl]-1H-benzimidazole	C20 H23 N3	305.18533	10.823	--	--	306.19266
Com_7485_pos	PNK	C15 H27 N5 O5	357.20584	7.599	--	--	358.2128
Com_7586_pos	LPC 14:0	C22 H46 N O7 P	467.30167	14.022	--	--	468.30902
Com_7840_pos	MMK	C16 H32 N4 O4 S2	390.17452	1.301	--	--	391.18173
Com_8232_pos	3',4'-Dihydroxyphenylacetone	C9 H10 O3	166.06326	1.946	--	--	167.07054
Com_8289_pos	4-ethoxy-6,7-dimethoxyquinazoline	C12 H14 N2 O3	234.10072	6.434	--	--	235.10812
Com_8344_pos	5,6-dimethoxy-2-(2-methoxyphenyl)-4H-chromen-4-one	C18 H16 O5	294.08936	10.644	--	--	295.09662
Com_8429_pos	Dihomo-γ-linolenoyl ethanolamide	C22 H39 N O2	349.29503	15.114	--	--	350.30231
Com_8449_pos	15-Deoxy-Δ12,14-prostaglandin J2-2-glycerol ester	C23 H34 O5	390.23855	14.008	--	--	391.246
Com_8510_pos	3-(dimethylamino)-1-[4-(phenylsulfonyl)phenyl]prop-2-en-1-one	C17 H17 N O3 S	315.09339	2.493	--	--	316.10077

Continuation of Supplementary Table 2

Compound_ID	Name	Formula	Molecular Weight	RT [min]	Kegg_ID	HMDB_ID	m/z
Com_8645_pos	3-Methoxy prostaglandin F1α	C21 H38 O6	368.25611	12.231	--	--	369.26352
Com_8650_pos	Valerophenone	C11 H14 O	162.10479	9.136	--	--	163.11224
Com_8653_pos	Proscillaridin A	C30 H42 O8	508.30478	13.22	--	--	509.31229
Com_8752_pos	ACar 20:3	C27 H48 N O4	449.35079	13.46	--	--	450.35855
Com_8781_pos	Cer-NS (d18:2/16:1)	C34 H63 N O3	551.49163	14.981	--	--	552.49896
Com_8800_pos	N'-(2-chlorobenzoyl)-N,N-dimethylhydrazonoformamide	C10 H12 Cl N3 O	225.06369	1.404	--	--	226.07103
Com_8883_pos	3-{[(3S)-3-(1,3-Benzothiazol-2-yl)-1-pyrrolidinyl]methyl}benzonitrile	C19 H17 N3 S	319.10925	2.416	--	--	320.11685
Com_9063_pos	1-(4-bromophenyl)-2-phenylethan-1-one	C14 H11 Br O	223.12064	1.401	--	--	224.12811
Com_9430_pos	LDGTS 16:2	C26 H47 N O6	469.34133	13.923	--	--	470.3486
Com_9437_pos	N-(4-methoxy-1-methyl-1H-indazol-3-yl)-5-methyl-3-isoxazolecarboxamide	C14 H14 N4 O3	286.10675	8.568	--	--	287.11453
Com_9449_pos	PC (14:0e/3:0)	C25 H52 N O7 P	509.34942	14.914	--	--	510.35645
Com_9514_pos	Tanespimycin	C31 H43 N3 O8	626.3282	12.658	--	--	627.33588
Com_9535_pos	10-methoxy-4H-benzo[4,5]cyclohepta[b]thiophen-4-one	C14 H10 O2 S	242.04258	12.357	--	--	243.04982
Com_9544_pos	N-Stearoyl taurine	C20 H41 N O4 S	408.30305	14.774	--	--	409.30997
Com_9599_pos	Sinapyl aldehyde	C11 H12 O4	208.07354	10.643	--	--	209.08105
Com_9630_pos	2-[3-(4-pyridyl)-1H-1,2,4-triazol-5-yl]pyridine	C12 H9 N5	223.08469	6.744	--	--	224.09204
Com_9759_pos	N-Methylisoleucine	C7 H15 N O2	145.11049	6.962	--	--	146.11769
Com_9886_pos	2-[({4-[3,5-di(tert-butyl)-1H-pyrazol-1-yl]phenyl}imino)methyl]phenol	C24 H29 N3 O	353.24368	12.286	--	--	354.25089

Continuation of Supplementary Table 2

Compound_ID	Name	Formula	Molecular Weight	RT [min]	Kegg_ID	HMDB_ID	m/z
Com_9969_pos	4-hydroxy-5,8-dimethylquinoline-3-carboxylic acid	C12 H11 N O3	217.07439	6.049	--	--	218.08163
Com_9998_pos	ABL 127	C17 H20 N2 O5	354.10825	8.362	--	--	355.11554
Com_10080_pos	2-(2-amino-3-methylbutanamido)-3-phenylpropanoic acid	C14 H20 N2 O3	264.14783	7.536	--	--	265.1553
Com_10193_pos	3-Methoxycinnamic acid	C10 H10 O3	160.05278	12.973	--	--	161.06006
Com_10307_pos	EQH	C16 H24 N6 O7	412.17131	11.017	--	--	413.17877
Com_10319_pos	Glycocholic acid hydrate	C26 H45 N O7	483.32131	12.865	--	--	484.32858
Com_10373_pos	cyclohexyl{4-[4-nitro-2-(1H-pyrrol-1-yl)phenyl]piperazino}methanone	C21 H26 N4 O3	382.19666	10.678	--	--	383.20401
Com_10489_pos	isobutyl 1-cyclohexyl-4-oxo-1,4-dihydroquinoline-3-carboxylate	C20 H25 N O3	327.18383	7.185	--	--	328.19125
Com_10492_pos	1,7-bis(3,4-dihydroxyphenyl)heptan-3-one	C19 H22 O5	312.13651	12.728	--	--	313.14392
Com_10499_pos	2,4-dihydroxyheptadec-16-en-1-yl acetate	C19 H36 O4	350.24431	13.842	--	--	351.25232
Com_10551_pos	Ingenol-3-angelate	C25 H34 O6	430.23685	13.09	--	--	431.24332
Com_10602_pos	SNK	C13 H25 N5 O6	347.17676	10.039	--	--	348.1842
Com_10619_pos	Prolylleucine	C11 H20 N2 O3	228.1474	1.974	--	--	229.15471
Com_10647_pos	N2-(2-cyano-4,6-difluorophenyl)thiophene-2-carboxamide	C12 H6 F2 N2 O S	264.01557	1.086	--	--	265.02283
Com_10777_pos	AMH	C14 H23 N5 O4 S	379.12651	7.798	--	--	380.13385
Com_10790_pos	(+/-)5(6)-EET	C20 H32 O3	320.23527	11.944	--	--	321.24246
Com_10821_pos	2-[(3S)-1-(2-Chlorobenzyl)-3-pyrrolidinyl]-5-methyl-1,3,4-oxadiazole	C14 H16 Cl N3 O	277.09524	7.959	--	--	278.10272

Continuation of Supplementary Table 2

Compound_ID	Name	Formula	Molecular Weight	RT [min]	Kegg_ID	HMDB_ID	m/z
Com_10835_pos	3-(2-Naphthyl)-D-Alanine	C13 H13 N O2	215.09492	8.897	--	--	216.10205
Com_10935_pos	2-Amino-1,3,4-octadecanetriol	C18 H39 N O3	317.29313	12.22	--	--	318.30011
Com_10945_pos	Sodium cholate	C24 H39 Na O5	430.26987	14.001	--	--	431.27682
Com_11017_pos	Cyclopentyl fentanyl-d5	C25 H27 [2]H5 N2 O	182.12993	14.465	--	--	382.29333
Com_11033_pos	6-(trifluoromethoxy)quinolin-4-ol	C10 H6 F3 N O2	229.03506	6.098	--	--	230.0423
Com_11057_pos	3-methoxy-2-phenyl-4H-furo[2,3-h]chromen-4-one	C18 H12 O4	292.07341	9.379	--	--	293.08047
Com_11074_pos	3-hydroxy-1,5-diphenylpentan-1-one	C17 H18 O2	276.11121	8.951	--	--	277.11874
Com_11192_pos	FQH	C20 H26 N6 O5	412.18909	13.625	--	--	413.19635
Com_11226_pos	N-Acetyl-aspartic acid	C6 H9 N O5	175.04822	1.506	--	--	176.05557
Com_11232_pos	(12Z)-9,10,11-trihydroxyoctadec-12-enoic acid	C18 H34 O5	352.2229	12.51	--	--	353.23035
Com_11283_pos	1-(4-methyl-2-morpholino-1,3-thiazol-5-yl)ethan-1-one	C10 H14 N2 O2 S	226.08273	7.931	--	--	227.0899
Com_11361_pos	3-Acetyl-11-keto-β-boswellic acid	C32 H48 O5	512.34916	12.773	--	--	513.3562
Com_11380_pos	1,4-dihydroxy-1,4-dimethyl-7-(propan-2-ylidene)-decahydroazulen-6-one	C15 H24 O3	234.16191	9.658	--	--	235.16904
Com_11386_pos	DL-α-Aminocaprylic acid	C8 H17 N O2	159.12615	4.245	--	--	160.13342
Com_11493_pos	3-amino-4-(propylamino)cyclobut-3-ene-1,2-dione	C7 H10 N2 O2	154.07453	3.651	--	--	155.0818
Com_11511_pos	PC (18:4e/2:0)	C28 H50 N O7 P	543.33366	14.397	--	--	544.34113
Com_11512_pos	13,14-Dihydro prostaglandin E1	C20 H36 O5	338.24581	14.174	--	--	339.25339

Continuation of Supplementary Table 2

Compound_ID	Name	Formula	Molecular Weight	RT [min]	Kegg_ID	HMDB_ID	m/z
Com_11578_pos	(6E,10E)-3,7,11,15-tetramethylhexadeca-1,6,10,14-tetraene-3,5,9-triol	C20 H34 O3	339.27741	14.57	--	--	340.28467
Com_11672_pos	5-[5-(ethylsulfonyl)-2-hydroxyanilino]-5-oxopentanoic acid	C13 H17 N O6 S	297.06734	10.408	--	--	298.07462
Com_11907_pos	PE (15:0/15:0)	C35 H70 N O8 P	663.4885	15.795	--	--	664.49664
Com_11942_pos	Verrucarol	C15 H22 O4	266.15185	9.556	--	--	267.15884
Com_12077_pos	Arachidonoyl amide	C20 H33 N O	303.25632	14.327	--	--	304.2637
Com_12340_pos	C-6 NBD ceramide	C30 H49 N5 O6	557.35147	15.078	--	--	558.35876
Com_12420_pos	1-(7-methoxy-2-oxo-2H-chromen-8-yl)-3-methyl-2-oxobutyl acetate	C17 H18 O6	335.1345	9.93	--	--	336.14182
Com_12540_pos	Lysopc 17:0	C25 H52 N O7 P	509.35024	15.208	--	--	510.35751
Com_12584_pos	2-(acetylamino)-3-[4-(acetylamino)phenyl]acrylic acid	C13 H14 N2 O4	262.09553	8.138	--	--	263.10315
Com_12623_pos	3-hydroxy-4-methoxy-9H-xanthen-9-one	C14 H10 O4	242.05812	10.011	--	--	243.06544
Com_12688_pos	Arachidonoyl serinol	C23 H39 N O3	377.29304	14.149	--	--	378.30096
Com_12718_pos	Fmoc-L-Isoleucine	C21 H23 N O4	353.16254	10.906	--	--	354.16946
Com_13032_pos	Dehydroepiandrosterone (DHEA)	C19 H28 O2	270.19823	15.34	--	--	271.20551
Com_13033_pos	3-(3,4-Dihydroxyphenyl)-2-Methylalanine	C10 H13 N O4	211.08445	2.115	--	--	212.09181
Com_13046_pos	Y-L-Glutamyl-L-glutamic acid	C10 H16 N2 O7	276.09554	1.385	--	--	277.10318
Com_13169_pos	α-Lactose	C12 H22 O11	359.14231	1.336	--	--	360.14954
Com_13446_pos	RMK	C17 H35 N7 O4 S	433.24726	6.921	--	--	434.25467
Com_13548_pos	N-Acetyl-D-lactosamine	C14 H25 N O11	383.14085	7.28	--	--	384.14841

Continuation of Supplementary Table 2

Compound_ID	Name	Formula	Molecular Weight	RT [min]	Kegg_ID	HMDB_ID	m/z
Com_13726_pos	ethyl 5-methoxy-2-methyl-1-phenyl-1H-indole-3-carboxylate	C19 H19 N O3	309.13711	7.679	--	--	310.1449
Com_13779_pos	Norfenefrine	C8 H11 N O2	153.0794	7.278	--	--	154.08673
Com_13805_pos	2-Benzyl-5-[(3S)-1-isopropyl-3-pyrrolidinyl]-1,3,4-oxadiazole	C16 H21 N3 O	271.16425	1.371	--	--	272.17163
Com_13807_pos	8-(2,3-dihydroxy-3-methylbutyl)-7-methoxy-2H-chromen-2-one	C15 H18 O5	260.10303	9.019	--	--	261.11032
Com_14037_pos	ACar 20:1	C27 H52 N O4	453.3819	13.962	--	--	454.38895
Com_14156_pos	5-[(8Z,11Z)-pentadeca-8,11-dien-1-yl]benzene-1,3-diol	C21 H32 O2	316.24037	13.538	--	--	317.24756
Com_14163_pos	19(R)-HETE	C20 H32 O3	320.23564	11.638	--	--	321.24286
Com_14228_pos	4-oxododecanedioic acid	C12 H20 O5	226.11831	9.048	--	--	227.1255
Com_14351_pos	4-{3-[(3,4-dihydroxyphenyl)methyl]-2-methylbutyl}benzene-1,2-diol	C18 H22 O4	324.13403	9.507	--	--	325.14139
Com_14454_pos	2-Arachidonoyl glycerol	C23 H38 O4	378.2772	14.675	--	--	379.28394
Com_14593_pos	2-(2,6-dimethoxyphenyl)-5,6-dimethoxy-4H-chromen-4-one	C19 H18 O6	342.11029	12.176	--	--	343.11746
Com_14621_pos	Cer-NDS (d17:0/15:0)	C32 H65 N O3	511.49738	15.271	--	--	512.50476
Com_14706_pos	Lagochilin	C20 H36 O5	356.2564	11.642	--	--	357.26373
Com_14843_pos	Lysine Butyrate	C10 H22 N2 O4	234.15844	1.335	--	--	235.1655
Com_14887_pos	DKK	C16 H31 N5 O6	389.22538	12.381	--	--	390.23138
Com_15038_pos	2-(acetyloxy)-3-amino-1-[1,2-di(acetyloxy)ethyl]-3-oxopropyl acetate	C13 H19 N O9	315.09296	3.302	--	--	316.10025

Continuation of Supplementary Table 2

Compound_ID	Name	Formula	Molecular Weight	RT [min]	Kegg_ID	HMDB_ID	m/z
Com_15096_pos	Boc-beta-cyano-L-alanine	C9 H14 N2 O4	214.09526	1.381	--	--	215.10252
Com_15261_pos	8-Aminooctanoic acid	C8 H17 N O2	159.12635	5.165	--	--	160.13368
Com_15477_pos	5-(6-hydroxy-6-methyloctyl)-2,5-dihydrofuran-2-one	C13 H22 O3	208.1462	9.575	--	--	209.1534
Com_15521_pos	(±)5(6)-DiHET	C20 H34 O4	355.27294	13.59	--	--	356.28061
Com_15522_pos	Cer-NDS (d18:0/16:0)	C34 H69 N O3	539.52891	16.208	--	--	540.53644
Com_15697_pos	(-)-Caryophyllene oxide	C15 H24 O	220.18296	13.512	--	--	221.1904
Com_15713_pos	PC (14:1e/2:0)	C24 H48 N O7 P	493.31806	14.234	--	--	494.32578
Com_15859_pos	1-(4-methoxyphenyl)propane-1,2-diol	C10 H14 O3	164.08401	9.122	--	--	165.09142
Com_15872_pos	N1-(1H-indol-4-yl)cyclohexane-1-carboxamide	C15 H18 N2 O	280.09251	10.978	--	--	281.09973
Com_15921_pos	3,5-di(2-furylmethylidene)tetrahydro-2H-pyran-4-one	C15 H12 O4	256.07353	9.205	--	--	257.0813
Com_15939_pos	N'2-(2-furylcarbonyl)-3-chloro-4-methylthiophene-2-carbohydrazide	C11 H9 Cl N2 O3 S	284.00118	2.173	--	--	285.00876
Com_16127_pos	11-Deoxy prostaglandin F1β	C20 H36 O4	322.25092	13.667	--	--	323.258
Com_16136_pos	(5S)-5-hydroxy-1,7-diphenylheptan-3-one	C19 H22 O2	282.16212	10.789	--	--	283.1694
Com_16144_pos	1,3-dimethyl 2,4-bis(4-hydroxyphenyl)cyclobutane-1,3-dicarboxylate	C20 H20 O6	356.12355	7.913	--	--	357.13104
Com_16618_pos	Linolelaidic Acid (C18:2N6T)	C18 H32 O2	280.24028	13.542	--	--	281.24734
Com_16834_pos	LNK	C16 H31 N5 O5	373.23686	8.858	--	--	374.24417
Com_16953_pos	4-(2,3-dihydro-1H-indol-1-yl)-1-phenyl-1H-pyrazolo[3,4-d]pyrimidine	C19 H15 N5	313.13157	7.521	--	--	314.13889

Continuation of Supplementary Table 2

Compound_ID	Name	Formula	Molecular Weight	RT [min]	Kegg_ID	HMDB_ID	m/z
Com_17177_pos	1-[(3,5-dimethylisoxazol-4-yl)sulfonyl]piperidine	C10 H16 N2 O3 S	244.08494	5.997	--	--	245.09215
Com_17264_pos	SQH	C14 H22 N6 O6	348.1791	9.653	--	--	349.18579
Com_17322_pos	LDGTS 20:4	C30 H51 N O6	521.37234	14.362	--	--	522.37946
Com_17402_pos	5-[(E)-2-(3,5-dihydroxyphenyl)ethenyl]-2-methoxybenzene-1,3-diol	C15 H14 O5	274.08428	7.893	--	--	275.09167
Com_17438_pos	6-(7-methyloctyl)-1H,3H,4H,6H-furo[3,4-c]furan-1-one	C15 H24 O3	252.17245	11.935	--	--	253.17981
Com_17477_pos	2-(2-hydroxy-3-methylbutanamido)-4-methylpentanoic acid	C11 H21 N O4	231.14725	8.726	--	--	232.15454
Com_17644_pos	3-[2-(3-Hydroxyphenyl)ethyl]-5-methoxyphenol	C15 H16 O3	244.11032	11.844	--	--	245.11763
Com_17664_pos	7-[(3,3-dimethyloxiran-2-yl)methoxy]-6-methoxy-2H-chromen-2-one	C15 H16 O5	276.09954	11.455	--	--	277.10709
Com_17761_pos	N-[1-(4-methoxy-2-oxo-2H-pyran-6-yl)-2-methylbutyl]acetamide	C13 H19 N O4	253.13196	2.885	--	--	254.13924
Com_17790_pos	PC (14:0e/2:0)	C24 H50 N O7 P	495.34014	14.864	--	--	496.34698
Com_17812_pos	N1-(4-oxo-2-phenyl-4H-chromen-3-yl)acetamide	C17 H13 N O3	279.08972	10.633	--	--	280.09702
Com_17980_pos	7-Hydroxycoumarine	C9 H6 O3	162.03214	8.099	--	--	163.03952
Com_18083_pos	(1R,2R)-trans-N-Boc-1,2-cyclohexanediamine	C11 H22 N2 O2	214.16833	8.341	--	--	215.17561
Com_18107_pos	4-oxo-4-[(1-phenylethyl)amino]but-2-enoic acid	C12 H13 N O3	236.11646	5.643	--	--	237.12379

Continuation of Supplementary Table 2

Compound_ID	Name	Formula	Molecular Weight	RT [min]	Kegg_ID	HMDB_ID	m/z
Com_18460_pos	dodecanohydrazide	C12 H26 N2 O	214.20466	9.69	--	--	215.21207
Com_18599_pos	N-[4-chloro-2-(2-chlorobenzoyl)phenyl]-2-(4-methylpiperidino)acetamide	C21 H22 Cl2 N2 O2	404.11139	11.922	--	--	405.11877
Com_18676_pos	3-(1,3-benzoxazol-2-ylthio)-5,5-dimethylcyclohex-2-en-1-one	C15 H15 N O2 S	273.085	2.211	--	--	274.09253
Com_18702_pos	6-(Methylthio)purine	C6 H6 N4 S	188.011	7.877	--	--	189.01825
Com_18734_pos	3-(3,4-dimethylphenyl)-3,4-dihydro-1,2,3-benzotriazin-4-one	C15 H13 N3 O	268.13104	13.072	--	--	269.13815
Com_19204_pos	4-[1,2,4]triazolo[4,3-b]pyridazin-6-ylthiomorpholine	C9 H11 N5 S	221.07232	1.406	--	--	222.07965
Com_19415_pos	VLK	C17 H34 N4 O4	358.25814	6.662	--	--	359.26575
Com_19487_pos	6 β-Hydroxycortisol	C21 H30 O6	378.20401	9.579	--	--	379.21136
Com_19516_pos	Stiripentol	C14 H18 O3	216.11525	11.185	--	--	217.12276
Com_19534_pos	N-Benzylethanolamine	C9 H13 N O	151.10015	11.508	--	--	152.10742
Com_19679_pos	(S)-Bromoenol lactone	C16 H13 Br O2	333.03268	7.693	--	--	334.03995
Com_19716_pos	LDGTS 16:1	C26 H49 N O6	471.35705	14.237	--	--	472.36432
Com_19725_pos	8(R)-Hydroxy-(5Z,9E,11Z,14Z)-eicosatetraenoic acid	C20 H32 O3	320.23495	13.606	--	--	321.2424
Com_19830_pos	4-[1-(fur-2-oyl)pyrazol-5-yl]-5-methyl-1-phenylpyrazole	C18 H14 N4 O2	318.11089	14.108	--	--	319.11819
Com_19932_pos	6-methyl-5-nitroquinoline	C10 H8 N2 O2	188.05864	10.083	--	--	189.06616
Com_20129_pos	PC (7:0/8:0)	C23 H46 N O8 P	495.29709	11.636	--	--	496.3042
Com_20172_pos	1-(2-furyl)pentane-1,4-dione	C9 H10 O3	188.04739	8.541	--	--	189.05481

Continuation of Supplementary Table 2

Compound_ID	Name	Formula	Molecular Weight	RT [min]	Kegg_ID	HMDB_ID	m/z
Com_20250_pos	5α-Tetrahydrocortisol	C21 H34 O5	754.4649	12.828	--	--	755.47205
Com_20274_pos	N-Lauroylsarcosine	C15 H29 N O3	288.24538	14.602	--	--	289.25262
Com_20300_pos	ACar 18:0	C25 H50 N O4	427.36692	13.882	--	--	428.37378
Com_20314_pos	VNK	C15 H29 N5 O5	341.206	2.704	--	--	342.21329
Com_20564_pos	Methanandamide	C23 H39 N O2	361.29751	14.663	--	--	362.30478
Com_20575_pos	N-cyclohexyl-N-methyl-6-quinoxalinecarboxamide	C16 H19 N3 O	291.13147	1.364	--	--	292.13852
Com_20627_pos	2-[(3S)-1-(1H-Indol-3-ylmethyl)-3-pyrrolidinyl]-1H-benzimidazole	C20 H20 N4	316.16563	13.177	--	--	317.17279
Com_20668_pos	Irganox 259	C40 H62 O6	660.43696	14.606	--	--	661.44434
Com_20690_pos	4-Hydroxypropranolol	C16 H21 N O3	275.1524	6.445	--	--	276.15982
Com_20871_pos	1-(6-methyl-3-pyridyl)ethan-1-one O1-ethyloxime hydrochloride	C10 H14 N2 O	178.11066	2.291	--	--	179.11784
Com_20924_pos	DLK	C16 H30 N4 O6	374.21642	2.592	--	--	375.22369
Com_20933_pos	LLK	C18 H36 N4 O4	186.13696	5.816	--	--	187.14436
Com_20999_pos	(2S)-2-(2-hydroxypropan-2-yl)-2H,3H,7H-furo[3,2-g]chromen-7-one	C14 H14 O4	246.08954	11.795	--	--	247.09683
Com_21044_pos	dimethyl 4-(acetylamino)cyclohex-3-ene-1,3-dicarboxylate	C12 H17 N O5	255.11077	9.507	--	--	256.11826
Com_21137_pos	Carbaprostacyclin	C21 H34 O4	332.23525	11.305	--	--	333.2424
Com_21293_pos	PLK	C17 H32 N4 O4	356.2421	6.759	--	--	357.24948
Com_21416_pos	RKK	C18 H38 N8 O4	215.15244	5.177	--	--	216.15977

Continuation of Supplementary Table 2

Compound_ID	Name	Formula	Molecular Weight	RT [min]	Kegg_ID	HMDB_ID	m/z
Com_21424_pos	4-decyl-3-hydroxy-5-oxooxolane-2,3-dicarboxylic acid	C16 H26 O7	347.19463	8.434	--	--	348.2019
Com_21496_pos	3-benzyl-4-hydroxy-5-(4-hydroxyphenyl)-2,5-dihydrofuran-2-one	C17 H14 O4	264.07877	9.187	--	--	265.08609
Com_21693_pos	methyl 1-methyl-1,2,5,6-tetrahydropyridine-3-carboxylate hydrobromide	C8 H13 N O2	155.09469	1.64	--	--	156.10197
Com_21748_pos	Gemifloxacin	C18 H20 F N5 O4	389.14995	6.518	--	--	390.15701
Com_21924_pos	N-Desmethyl sildenafil	C21 H28 N6 O4 S	438.20064	9.333	--	--	439.20798
Com_22029_pos	ethyl 1-(3-nitro-2-thienyl)piperidine-4-carboxylate	C12 H16 N2 O4 S	266.07016	8.188	--	--	267.07761
Com_22220_pos	TLK	C16 H32 N4 O5	161.14036	1.392	--	--	361.24332
Com_22262_pos	PNH	C15 H22 N6 O5	366.16496	9.847	--	--	367.17224
Com_22309_pos	(±)11(12)-EET	C20 H32 O3	302.22479	12.774	--	--	303.23199
Com_22310_pos	3,6-dibenzyl-1,2-dihydro-1,2,4,5-tetraazine	C16 H16 N4	264.13371	7.856	--	--	265.14099
Com_22503_pos	PPK	C16 H28 N4 O4	170.10569	1.403	--	--	171.11314
Com_22551_pos	AKB48 N-(4-hydroxypentyl) metabolite	C23 H31 N3 O2	381.23728	7.721	--	--	382.24445
Com_22689_pos	5-(2-chloro-6-fluorobenzyl)-6-methyl-2-morpholinopyrimidin-4-ol	C16 H17 Cl F N3 O2	337.09811	9.027	--	--	338.10535
Com_23010_pos	6-(4-phenylpiperazino)hexanoic acid hydrochloride	C16 H24 N2 O2	276.18421	6.605	--	--	277.19165
Com_23054_pos	1-[(4-chlorobenzyl)sulfonyl]azepane	C13 H18 Cl N O2 S	345.088	1.404	--	--	346.09518
Com_23055_pos	N1-(4-chlorobenzylidene)piperidin-1-amine	C12 H15 Cl N2	222.09297	1.366	--	--	223.10068

Continuation of Supplementary Table 2

Compound_ID	Name	Formula	Molecular Weight	RT [min]	Kegg_ID	HMDB_ID	m/z
Com_23120_pos	Citral	C10 H16 O	152.12038	10.259	--	--	153.12769
Com_23201_pos	2-[(3S)-1-Benzyl-3-pyrrolidinyl]-1,3-benzothiazole	C18 H18 N2 S	294.12183	5.953	--	--	295.12958
Com_23579_pos	(2,6-dimethylpiperidino)(3,4,5-trimethoxyphenyl)methanone	C17 H25 N O4	307.17851	7.803	--	--	308.18588
Com_23608_pos	IMK	C17 H34 N4 O4 S	372.22577	9.466	--	--	373.23352
Com_23713_pos	(9cis)-Retinal	C20 H28 O	284.21423	12.88	--	--	285.22165
Com_23749_pos	16-Heptadecyne-1,2,4-triol	C17 H32 O3	306.21947	12.616	--	--	307.22675
Com_23910_pos	ILK	C18 H36 N4 O4	372.27358	7.972	--	--	373.28146
Com_23950_pos	1,2,10-Trimethoxy-5,6,6a,7-tetrahydro-4H-dibenzo[de,g]quinolin-9-ol	C19 H21 N O4	327.14749	7.385	--	--	328.15503
Com_24028_pos	(4-nitrophenyl)(2,3,4,5,6-pentamethylphenyl)methanone	C18 H19 N O3	297.13698	6.816	--	--	298.14444
Com_24117_pos	(2R,3S,4S,5R,6R)-2-(hydroxymethyl)-6-(2-phenylethoxy)oxane-3,4,5-triol	C14 H20 O6	306.10839	9.527	--	--	307.1156
Com_24156_pos	Monolaurin	C15 H30 O4	312.17274	12.274	--	--	313.18002
Com_24191_pos	N-Desmethylclomipramine	C18 H21 Cl N2	300.14295	1.397	--	--	301.15018
Com_24201_pos	N-(2-hydroxyphenyl)acetamide	C8 H9 N O2	173.04783	4.49	--	--	174.05524
Com_24246_pos	N1-[4-(2,5-dimethyl-1H-pyrrol-1-yl)benzylidene]-4-chloroaniline	C19 H17 Cl N2	308.10516	11.931	--	--	309.11246
Com_24258_pos	2-(4,4-diphenyl-1-piperidinobuta-1,3-dienyl)phenyl acetate	C29 H29 N O2	423.22625	5.835	--	--	424.2337

Continuation of Supplementary Table 2

Compound_ID	Name	Formula	Molecular Weight	RT [min]	Kegg_ID	HMDB_ID	m/z
Com_24491_pos	3-hydroxy-N-(1-hydroxy-4-methylpentan-2-yl)-5-oxo-6-phenylhexanamide	C18 H27 N O4	359.15123	6.522	--	--	360.15848
Com_24502_pos	1-benzyl-3-butyl-4-hydroxy-6-phenylpyridin-2(1H)-one	C22 H23 N O2	333.16847	7.7	--	--	334.17581
Com_24518_pos	2-(4-aminophenoxy)isophthalonitrile	C14 H9 N3 O	235.07231	1.229	--	--	236.07961
Com_24659_pos	Ethyl protocatechuate	C9 H10 O4	182.05812	6.663	--	--	183.06506
Com_24669_pos	ACar 20:2	C27 H50 N O4	469.37734	13.794	--	--	470.38431
Com_24689_pos	2,3,4-Trihydroxybenzoic acid	C7 H6 O5	170.02176	12.903	--	--	171.02884
Com_24734_pos	N1,N1-diethyl-4-[2-(4-bromophenyl)diaz-1-enyl]aniline	C16 H18 Br N3	331.06662	3.303	--	--	332.07391
Com_24755_pos	GPH	C13 H19 N5 O4	347.1003	7.412	--	--	348.10773
Com_25012_pos	2-(3,5-dimethyl-1H-pyrazol-4-yl)-5-methoxybenzoic acid	C13 H14 N2 O3	246.10023	7.62	--	--	247.10754
Com_25016_pos	(4E)-1,7-bis(3,4-dihydroxyphenyl)hept-4-en-3-one	C19 H20 O5	345.15869	12.211	--	--	346.16525
Com_25290_pos	16,16-Dimethyl prostaglandin A2	C22 H34 O4	384.22894	14.462	--	--	385.23721
Com_25318_pos	2-[5-(2-hydroxypropyl)oxolan-2-yl]propanoic acid	C10 H18 O4	224.105	10.764	--	--	225.11227
Com_25579_pos	Radicinin	C12 H12 O5	236.06572	7.596	--	--	237.0732
Com_25872_pos	FKK	C21 H35 N5 O4	403.25817	12.509	--	--	404.26553
Com_25928_pos	APH	C14 H21 N5 O4	361.11534	7.819	--	--	362.12259
Com_26073_pos	Anandamide (AEA)	C22 H37 N O2	347.282	14.429	--	--	348.28931
Com_26192_pos	β-Cortolone	C21 H34 O5	348.23005	10.26	--	--	349.237

Continuation of Supplementary Table 2

Compound_ID	Name	Formula	Molecular Weight	RT [min]	Kegg_ID	HMDB_ID	m/z
Com_26193_pos	HexCer-NS (d18:1/16:1)	C40 H75 N O8	697.54973	15.275	--	--	698.55737
Com_26200_pos	7-(2-aminophenyl)heptanoic acid	C13 H19 N O2	243.12192	2.389	--	--	244.12924
Com_26294_pos	4-oxo-4-[(pyridin-4-ylmethyl)amino]but-2-enoic acid	C10 H10 N2 O3	206.06942	7.185	--	--	207.07669
Com_26383_pos	C-8 Ceramide-1-phosphate	C26 H52 N O6 P	505.35378	14.886	--	--	506.36118
Com_26511_pos	6β-Naltrexol	C20 H25 N O4	343.17406	7.657	--	--	344.18164
Com_26588_pos	MPH	C16 H25 N5 O4 S	405.14243	6.71	--	--	406.14984
Com_26691_pos	ethyl 5-(2-methoxy-2-oxoethoxy)-2-phenyl-1-benzofuran-3-carboxylate	C20 H18 O6	354.11024	9.497	--	--	355.11746
Com_26822_pos	SNH	C13 H20 N6 O6	378.12149	8.524	--	--	379.12885
Com_26952_pos	N-(3-chloro-2-methylphenyl)-N'-(3-methoxypropyl)thiourea	C12 H17 Cl N2 O S	272.07749	7.381	--	--	273.08481
Com_26972_pos	(+/-)5(6)-EET Ethanolamide	C22 H37 N O3	345.27032	14.55	--	--	346.27795
Com_26994_pos	LDGTS 20:5	C30 H49 N O6	519.35703	14.138	--	--	520.3642
Com_27120_pos	N(6)-OH-Me-Adenosine	C11 H15 N5 O5	297.10376	6.276	--	--	298.11124
Com_27141_pos	SPH	C14 H21 N5 O5	321.14242	1.377	--	--	322.14917
Com_27211_pos	1-(4-butylphenyl)-3-(dimethylamino)propan-1-one hydrochloride	C15 H23 N O	233.18157	8.516	--	--	234.18892
Com_27618_pos	2-(4-methylphenyl)-5-(3,4,5-trimethoxyphenyl)-2H-1,2,3,4-tetraazole	C17 H18 N4 O3	652.27937	8.864	--	--	327.14709
Com_27655_pos	N-(3-methylphenyl)-2-(3-pyridinyl)-1-pyrrolidinecarboxamide	C17 H19 N3 O	281.15278	11.904	--	--	282.1601
Com_27845_pos	3-[(5-nitropyridin-2-yl)oxy]-1H-indazole	C12 H8 N4 O3	234.07414	1.921	--	--	235.08157

Continuation of Supplementary Table 2

Compound_ID	Name	Formula	Molecular Weight	RT [min]	Kegg_ID	HMDB_ID	m/z
Com_27936_pos	5-methoxy-8,8-dimethyl-2-phenyl-4H,8H-pyrano[2,3-h]chromen-4-one	C21 H18 O4	334.11688	5.899	--	--	335.12424
Com_28294_pos	2-hydroxy-3,6-diphenylcyclohexyl acetate	C20 H22 O3	332.13742	7.21	--	--	333.14471
Com_28482_pos	4-methyl-5-oxo-2-pentyl-2,5-dihydrofuran-3-carboxylic acid	C11 H16 O4	212.10515	7.332	--	--	213.11252
Com_28488_pos	1-(3-ethyl-2,4-dihydroxy-6-methoxyphenyl)butan-1-one	C13 H18 O4	238.12065	9.202	--	--	239.12782
Com_28561_pos	TKK	C16 H33 N5 O5	375.2407	8.145	--	--	376.24799
Com_28563_pos	Zeatin-7-N-glucoside	C16 H23 N5 O6	381.16351	6.109	--	--	382.1709
Com_28721_pos	2-Methoxyresorcinol	C7 H8 O3	140.04771	12.933	--	--	141.05499
Com_29021_pos	16H-dinaphtho[2,1-d:1,2-g][1,3]dioxocine	C22 H16 O2	312.11455	8.226	--	--	313.12198
Com_29148_pos	4'-(Imidazol-1-yl)acetophenone	C11 H10 N2 O	186.07953	9.538	--	--	187.08688
Com_29256_pos	N-butyl-N'-[5-(tert-butyl)-1,3,4-thiadiazol-2-yl]urea	C11 H20 N4 O S	278.11366	8.356	--	--	279.12109
Com_29440_pos	PLH	C17 H27 N5 O4	347.19426	6.926	--	--	348.20154
Com_29636_pos	α-Lapachone	C15 H14 O3	264.07213	5.615	--	--	265.07977
Com_29772_pos	5-Methoxyindole-3-Carbaldehyde	C10 H9 N O2	175.06323	1.39	--	--	176.07053
Com_29986_pos	1,6-dihydroxy-3-methoxy-8-methyl-9H-xanthen-9-one	C15 H12 O5	272.06831	13.416	--	--	273.07556
Com_30223_pos	2,3-dihydroxypropyl 12-methyltridecanoate	C17 H34 O4	284.23524	16.082	--	--	285.24265
Com_30303_pos	2-{[2-(4-methylpiperazino)phenyl]methylene}hydrazine-1-carbothioamide	C13 H19 N5 S	299.11594	7.021	--	--	300.12326

Continuation of Supplementary Table 2

Compound_ID	Name	Formula	Molecular Weight	RT [min]	Kegg_ID	HMDB_ID	m/z
Com_30645_pos	N-(p-Coumaroyl) serotonin	C19 H18 N2 O3	666.24386	11.742	--	--	667.25153
Com_30666_pos	2-acetamido-3-(4-methoxyphenyl)propanoic acid	C12 H15 N O4	237.10009	10.872	--	--	238.10736
Com_30920_pos	JWH-018 N-(3-methylbutyl) isomer	C24 H23 N O	341.18393	7.966	--	--	342.19125
Com_31055_pos	N-{[(2R,4S,5R)-5-Ethyl-1-azabicyclo[2.2.2]oct-2-yl]methyl}-2-furamide	C15 H22 N2 O2	262.16818	5.753	--	--	263.17542
Com_31114_pos	5-Methyl-8-nitro-3-spirocyclohexyl-2,3,4,5-tetrahydro-1H-2-benzazepine	C16 H22 N2 O2	274.17135	11.203	--	--	275.17874
Com_31218_pos	5,8-dihydroxy-10-methyl-5,8,9,10-tetrahydro-2H-oxecin-2-one	C10 H14 O4	180.07879	13.5	--	--	181.08595
Com_31232_pos	MLK	C17 H34 N4 O4 S	372.21877	7.723	--	--	373.22598
Com_31235_pos	5-(1-azepanyl)-2,4(1H,3H)-pyrimidinedione	C10 H15 N3 O2	209.1165	6.833	--	--	210.12384
Com_31294_pos	3-hydroxy-4-(3-hydroxyphenyl)-1,2-dihydroquinolin-2-one	C15 H11 N O3	253.07359	12.04	--	--	254.08087
Com_31331_pos	3-(3-pyridinyl)propanoic acid	C8 H9 N O2	151.06356	5.475	--	--	152.07089
Com_31456_pos	δ-Valerolactam	C5 H9 N O	117.07914	5.587	--	--	100.07587
Com_31484_pos	(1E,4Z,6E)-5-hydroxy-1,7-bis(4-hydroxyphenyl)hepta-1,4,6-trien-3-one	C19 H16 O4	326.11611	12.169	--	--	327.12283
Com_31633_pos	Lenalidomide	C13 H13 N3 O3	276.11855	10.529	--	--	277.12582
Com_31760_pos	XLR11 N-(4-hydroxypentyl) metabolite-d5	C21 H23 [2]H5 F N O2	328.26179	15.168	--	--	329.2691
Com_31794_pos	Sildenafil N-oxide	C22 H30 N6 O5 S	490.19505	6.27	--	--	491.20239

Continuation of Supplementary Table 2

Compound_ID	Name	Formula	Molecular Weight	RT [min]	Kegg_ID	HMDB_ID	m/z
Com_31838_pos	2-[(3S)-1-(4-Fluorobenzyl)-3-pyrrolidinyl]-5-methyl-1H-benzimidazole	C19 H20 F N3	309.16068	5.757	--	--	310.16821
Com_31891_pos	2-{[2-oxo-2-(3-pyridylamino)ethyl]thio}acetic acid	C9 H10 N2 O3 S	226.04126	10.163	--	--	227.04861
Com_31918_pos	5-fluoro AB-PINACA N-(4-hydroxypentyl) metabolite	C18 H25 F N4 O3	386.17081	12.571	--	--	387.17813
Com_31988_pos	(2R,3S,4S,5R,6S)-2-(hydroxymethyl)-6-phenoxyoxane-3,4,5-triol	C12 H16 O6	273.12097	1.384	--	--	274.12848
Com_32035_pos	1-allyl-4,5-diphenyl-2-(2-thienyl)-1H-imidazole	C22 H18 N2 S	152.08173	9.003	--	--	343.12662
Com_32183_pos	3-{[(3S)-3-(1,3-Benzoxazol-2-yl)-1-pyrrolidinyl]methyl}benzonitrile	C19 H17 N3 O	606.27304	7.949	--	--	304.1438
Com_32188_pos	6-methoxy-2,3,4,9-tetrahydro-1H-beta-carbolin-1-one	C12 H12 N2 O2	216.09022	6.325	--	--	217.09761
Com_32373_pos	2-morpholino-1-phenyl-1-ethanol	C12 H17 N O2	189.11515	7.671	--	--	190.12244
Com_32432_pos	ERH	C17 H28 N8 O6	220.11004	13.693	--	--	221.1174
Com_32450_pos	4,6,8-trihydroxy-7-methoxy-3-methyl-3,4-dihydro-1H-2-benzopyran-1-one	C11 H12 O6	240.06286	6.105	--	--	241.07021
Com_32549_pos	Leu-Pro	C11 H21 Cl N2 O3	264.12435	11.233	--	--	546.28253
Com_32847_pos	JWH 412 N-(5-hydroxypentyl) metabolite	C24 H22 F N O2	375.16844	6.226	--	--	376.17584
Com_32867_pos	alprazolam-d5	C17 H8 [2]H5 Cl N4	313.11603	1.389	--	--	314.12479
Com_33044_pos	Sarpogrelate	C24 H31 N O6	429.21903	12.31	--	--	430.22607
Com_33170_pos	FPH	C20 H25 N5 O4	437.14769	9.667	--	--	438.15497

Continuation of Supplementary Table 2

Compound_ID	Name	Formula	Molecular Weight	RT [min]	Kegg_ID	HMDB_ID	m/z
Com_33213_pos	N'-[6-(tert-butyl)thieno[3,2-d]pyrimidin-4-yl]-4-methylbenzohydrazide	C18 H20 N4 O S	340.13143	13.687	--	--	341.13895
Com_33249_pos	4-(2,3-dihydro-1,4-benzodioxin-6-yl)-1,2-diphenylbut-2-ene-1,4-dione	C24 H18 O4	392.09438	5.815	--	--	393.10165
Com_33464_pos	O-Aceyl-L-Serine	C5 H9 N O4	147.05337	10.703	--	--	148.06061
Com_33536_pos	TPH	C15 H23 N5 O5	353.16603	7.89	--	--	354.1734
Com_33680_pos	4-(3,4-dimethoxyphenyl)-3-methyl-1H-pyrazol-5-amine	C12 H15 N3 O2	233.11657	7.885	--	--	234.1236
Com_33738_pos	6??-Hydroxytestosterone	C19 H28 O3	304.20386	11.21	--	--	305.21118
Com_33847_pos	N-{4-[(2R,3R)-3-(Hydroxymethyl)-5-oxo-2-morpholinyl]phenyl}acetamide	C13 H16 N2 O4	246.10098	5.769	--	--	247.10825
Com_33888_pos	N-(8-methyl-8-azabicyclo[3.2.1]oct-3-yl)-4-nitrobenzamide	C15 H19 N3 O3	289.13826	1.299	--	--	290.14554
Com_33920_pos	3-pentadecyl-4,5,6,7-tetrahydrobenzo[d]isoxazol-4-one oxime	C22 H38 N2 O2	362.29382	14.817	--	--	363.30087
Com_33972_pos	Bicyclo Prostaglandin E2	C20 H30 O4	356.19648	11.9	--	--	357.20337
Com_34059_pos	(2R)-2-[(2R,5S)-5-[(2S)-2-hydroxybutyl]oxolan-2-yl]propanoic acid	C11 H20 O4	198.12558	8.872	--	--	199.133
Com_34192_pos	2-[2-(5-bromo-2-pyridyl)diaz-1-enyl]-5-(diethylamino)phenol	C15 H17 Br N4 O	348.06096	9.209	--	--	349.06839
Com_34208_pos	2-[(carboxymethyl)(methyl)amino]-5-methoxybenzoic acid	C11 H13 N O5	239.07923	6.765	--	--	240.08678
Com_34216_pos	Leucine-enkephalin	C28 H37 N5 O7	555.26893	9.658	--	--	556.27631

Continuation of Supplementary Table 2

Compound_ID	Name	Formula	Molecular Weight	RT [min]	Kegg_ID	HMDB_ID	m/z
Com_34426_pos	MNK	C15 H29 N5 O5 S	409.19417	1.42	--	--	410.20132
Com_34506_pos	AMK	C14 H28 N4 O4 S	348.17987	7.459	--	--	349.18716
Com_34746_pos	2-[(5-chloro-3-pyridyl)oxy]-5-(1H-pyrrol-1-yl)pyridine	C14 H10 Cl N3 O	271.04903	6.943	--	--	272.0563
Com_34814_pos	Baohuoside I	C27 H30 O10	514.18474	12.954	--	--	515.19202
Com_34881_pos	4-(methylthio)-6-phenyl-2-(3-pyridyl)pyrimidine-5-carbonitrile	C17 H12 N4 S	304.07689	1.334	--	--	305.08398
Com_34975_pos	2-(3,4-dihydroxyphenyl)-3,5,7-trihydroxy-6-methyl-4H-chromen-4-one	C16 H12 O7	316.05833	11.403	--	--	317.06558
Com_35291_pos	N-(9-oxodecyl)acetamide	C12 H23 N O2	213.17305	10.039	--	--	214.18042
Com_35320_pos	5-fluoro AKB48 N-(4-hydroxypentyl) metabolite	C23 H30 F N3 O2	437.19589	10.923	--	--	438.2034
Com_35334_pos	16,16-Dimethyl prostaglandin A1	C22 H36 O4	346.24886	13.616	--	--	347.25613
Com_35369_pos	N6-Me-dA	C11 H15 N5 O3	265.11791	5.827	--	--	266.12497
Com_35474_pos	(2E,4E)-N-(2-methylpropyl)deca-2,4-dienamide	C14 H25 N O	241.20412	12.623	--	--	242.21106
Com_35528_pos	2-{[(4,5-dimethoxy-2-nitrophenethyl)imino]methyl}phenol	C17 H18 N2 O5	330.122	9.757	--	--	331.12955
Com_35605_pos	DMH	C15 H23 N5 O6 S	201.17298	6.951	--	--	202.18045
Com_36050_pos	methyl 2-[(2-acetyl-3-oxo-1-butenyl)amino]acetate	C9 H13 N O4	199.08466	6.232	--	--	200.09204
Com_36109_pos	Caprolactam	C6 H11 N O	113.08434	7.597	--	--	114.09137
Com_36162_pos	SKK	C15 H31 N5 O5	339.24933	12.766	--	--	362.23856

Continuation of Supplementary Table 2

Compound_ID	Name	Formula	Molecular Weight	RT [min]	Kegg_ID	HMDB_ID	m/z
Com_36192_pos	3-(5-phenyl-1,3-oxazol-2-yl)-4-(trifluoromethyl)pyridine	C15 H9 F3 N2 O	290.06253	4.958	--	--	291.06992
Com_36255_pos	(2E)-1-(2-hydroxy-3,4,5,6-tetramethoxyphenyl)-3-phenylprop-2-en-1-one	C19 H20 O6	326.11544	9.499	--	--	327.12244
Com_36481_pos	JWH-250 N-(5-hydroxypentyl) metabolite	C22 H25 N O3	351.17872	7.051	--	--	352.18558
Com_36513_pos	N-(1,3-benzodioxol-5-ylmethyl)-6-morpholinonicotinamide	C18 H19 N3 O4	341.1372	8.817	--	--	342.14447
Com_36727_pos	methyl isoquinoline-3-carboxylate	C11 H9 N O2	187.06092	5.535	--	--	188.06824
Com_37000_pos	YNH	C19 H24 N6 O6	410.19418	9.288	--	--	411.20044
Com_37014_pos	4-(1H-pyrazol-1-yl)-N,N-bis(2-pyridinylmethyl)benzenesulfonamide	C21 H19 N5 O2 S	405.12461	1.309	--	--	406.13202
Com_37025_pos	4-[4-chloro-2-nitro-5-(1H-pyrrol-1-yl)phenyl]morpholine	C14 H14 Cl N3 O3	307.07268	5.342	--	--	308.07983
Com_37068_pos	4-(4-hydroxy-3,5-dimethylphenyl)-2-methylphthalazin-1(2H)-one	C17 H16 N2 O2	280.12114	8.357	--	--	281.12833
Com_37481_pos	Evernic acid	C17 H16 O7	332.08987	12.772	--	--	333.09714
Com_37514_pos	2-{[2-(3-cyano-2-pyridinyl)hydrazino]carbonyl}benzoic acid	C14 H10 N4 O3	282.07403	7.843	--	--	283.08148
Com_37585_pos	Sunitinib	C22 H27 F N4 O2	420.19209	11.914	--	--	421.19937
Com_37635_pos	5,6-dimethyl-4-oxo-4H-pyran-2-carboxylic acid	C8 H8 O4	168.04256	7.042	--	--	169.04999
Com_37735_pos	α-Linolenoyl ethanolamide	C20 H35 N O2	321.26691	10.9	--	--	322.27408
Com_37971_pos	7-{[(2E)-3,7-dimethylocta-2,6-dien-1-yl]oxy}-2H-chromen-2-one	C19 H22 O3	320.13832	8.292	--	--	321.14621

Continuation of Supplementary Table 2

Compound_ID	Name	Formula	Molecular Weight	RT [min]	Kegg_ID	HMDB_ID	m/z
Com_38027_pos	11β-Prostaglandin F2α	C20 H34 O5	376.22088	8.027	--	--	377.22842
Com_38081_pos	Tramadol N-Oxide	C16 H25 N O3	279.18327	8.127	--	--	280.19067
Com_38167_pos	4,7,8-trimethoxyfuro[2,3-b]quinoline	C14 H13 N O4	259.08777	8.988	--	--	260.09506
Com_38255_pos	O-7460	C25 H48 F O5 P	500.29587	13.265	--	--	501.30414
Com_38283_pos	DGTS (16:0/18:3)	C44 H79 N O7	733.58818	15.923	--	--	734.59546
Com_38299_pos	Cer-NS (d19:3/18:2)	C37 H65 N O3	571.49679	15.389	--	--	572.50415
Com_38345_pos	4-oxo-5-phenylpentanoic acid	C11 H12 O3	174.06841	8.854	--	--	175.07568
Com_38351_pos	HPK	C17 H28 N6 O4	380.20961	11.298	--	--	381.21634
Com_38733_pos	LysoPC 12:1	C20 H36 N O7 P	433.22099	13.689	--	--	434.22836
Com_38844_pos	APK	C14 H26 N4 O4	356.20557	6.832	--	--	357.21326
Com_38863_pos	N-(2,4-Dimethylphenyl)formamide	C9 H11 N O	149.0844	2.155	--	--	150.09181
Com_38928_pos	2-{4-[3-(trifluoromethyl)-1H-pyrazol-1-yl]phenyl}isoindoline-1,3-dione	C18 H10 F3 N3 O2	357.06728	11.564	--	--	358.07449
Com_39009_pos	1-morpholino-3-(4-nitrophenoxy)propan-2-ol	C13 H18 N2 O5	282.12136	4.816	--	--	283.12857
Com_39066_pos	1-(5,7-dichloro-2,3,4,4a-tetrahydro-1H-xanthen-4-yl)pyrrolidine	C17 H19 Cl2 N O	323.08622	6.892	--	--	324.09375
Com_39129_pos	2-(carboxymethoxy)-4-methoxybenzoic acid	C10 H10 O6	248.02942	6.804	--	--	249.03668
Com_39224_pos	3,4-dihydroxy-4-(4-methoxyphenyl)-1,2,3,4-tetrahydroquinolin-2-one	C16 H15 N O4	285.10024	6.485	--	--	286.10767
Com_39328_pos	LysoPC 10:0	C18 H38 N O7 P	411.23884	12.72	--	--	412.24625
Com_39438_pos	Flunitrazepam-d3	C16 H9 [2]H3 F N3 O3	316.10622	7.304	--	--	317.11359
Com_39547_pos	PC (7:0/7:0)	C22 H44 N O8 P	481.28798	14.367	--	--	482.29498

Continuation of Supplementary Table 2

Compound_ID	Name	Formula	Molecular Weight	RT [min]	Kegg_ID	HMDB_ID	m/z
Com_39695_pos	Adipamide	C6 H12 N2 O2	144.09	1.289	--	--	167.07927
Com_39791_pos	(1R,2S,3R,4R)-3-(Isobutylamino)-4-(3-pyridinyl)-1,2-cyclopentanediol	C14 H22 N2 O2	272.1532	12.945	--	--	562.34082
Com_39838_pos	N,N-dimethyl-9H-purin-6-amine	C7 H9 N5	163.0861	5.107	--	--	164.09335
Com_39849_pos	HNK	C16 H27 N7 O5	379.19932	6.544	--	--	380.20731
Com_39955_pos	Meperidine-d5	C15 H16 [2]H5 N O2	252.18764	13.836	--	--	253.1949
Com_39995_pos	N1-cyclooctyl-4-hydroxy-1-piperidinecarbothioamide	C14 H26 N2 O S	292.15368	6.473	--	--	293.1611
Com_40077_pos	FLK	C21 H34 N4 O4	406.25813	6.517	--	--	407.26544
Com_40078_pos	N-[2-(1,5-dimethyl-4-nitro-1H-pyrazol-3-yl)vinyl]-N,N-dimethylamine	C9 H14 N4 O2	210.11182	6.088	--	--	211.11923
Com_40367_pos	4-(4-chlorophenoxy)-3,5-dimethyl-1H-pyrazole	C11 H11 Cl N2 O	222.05266	9.735	--	--	223.05974
Com_40598_pos	Octyl hydrogen phthalate	C16 H22 O4	278.15041	6.856	--	--	279.15768
Com_40634_pos	diethyl 3-amino-6-methylthieno[2,3-b]pyridine-2,5-dicarboxylate	C14 H16 N2 O4 S	308.0853	8.715	--	--	309.09195
Com_40752_pos	WLH	C23 H30 N6 O4	436.22063	8.126	--	--	437.22791
Com_40838_pos	9-(2,3-Dihydroxy-3-methylbutoxy)-7H-furo[3,2-g]chromen-7-one	C16 H16 O6	304.09294	8.879	--	--	305.10022
Com_40995_pos	1-[1,1'-biphenyl]-4-yl-2-(2-methyl-4-nitro-1H-imidazol-1-yl)-1-ethanol	C18 H17 N3 O3	361.08315	1.383	--	--	362.09
Com_41166_pos	N2-tetrahydrofuran-2-ylmethyl-4-(4-fluorophenyl)-1,3-thiazol-2-amine	C14 H15 F N2 O S	278.09002	5.859	--	--	279.09723

Continuation of Supplementary Table 2

Compound_ID	Name	Formula	Molecular Weight	RT [min]	Kegg_ID	HMDB_ID	m/z
Com_41257_pos	4-[4-phenyl-3,6-dihydro-1(2H)-pyridinyl]butanoic acid hydrochloride	C15 H19 N O2	267.12603	15.275	--	--	268.13315
Com_41381_pos	2-[(3S)-1-(3,4-Difluorobenzyl)-3-pyrrolidinyl]-1,3-benzoxazole	C18 H16 F2 N2 O	314.12702	9.388	--	--	315.13422
Com_41392_pos	Indole-3-acrylic acid	C11 H9 N O2	187.06332	10.342	--	--	188.07053
Com_41614_pos	(+/-)5(6)-DiHET	C20 H34 O4	355.27205	10.994	--	--	356.27939
Com_41740_pos	N'-[(2,4-dihydroxyphenyl)methylene]-4-methylbenzenesulfonohydrazide	C14 H14 N2 O4 S	295.11718	5.881	--	--	296.12463
Com_41806_pos	4-chloro-N~1~-(4-pyridinyl)-1,2-benzenediamine	C11 H10 Cl N3	219.05301	10.219	--	--	220.06027
Com_41807_pos	SLK	C15 H30 N4 O5	346.221	2.082	--	--	347.22839
Com_41886_pos	3-hydroxy-2-[5-nitro-2-(1-pyrrolidinyl)benzyl]propanenitrile	C14 H17 N3 O3	275.12691	6.858	--	--	276.13412
Com_42093_pos	8-chloro-6-(trifluoromethyl)imidazo[1,2-a]pyridine-2-carbohydrazide	C9 H6 Cl F3 N4 O	278.01289	2.131	--	--	279.02014
Com_42282_pos	5-acetyl-2,6-dimethyl-1,2,3,4-tetrahydropyridin-4-one	C9 H13 N O2	149.08431	5.076	--	--	150.09152
Com_42373_pos	trans,trans-2,4-Heptadienal	C7 H10 O	110.07343	12.107	--	--	111.0807
Com_42433_pos	Cer-NS (d14:2/16:1)	C30 H55 N O3	455.43661	16.095	--	--	456.44189
Com_42800_pos	2-(3,4-dimethoxyphenyl)-N-(4-morpholinophenyl)acetamide	C20 H24 N2 O4	356.17286	8.754	--	--	357.17993

Continuation of Supplementary Table 2

Compound_ID	Name	Formula	Molecular Weight	RT [min]	Kegg_ID	HMDB_ID	m/z
Com_42814_pos	dimethyl N~1~,N~4~-dicyanotetrahydropyrazine-1,4-dicarbimidothioate	C10 H14 N6 S2	282.07144	6.161	--	--	283.07886
Com_42838_pos	N-Acetyl-DL-phenylalanine	C11 H13 N O3	207.0898	10.311	--	--	208.09669
Com_42856_pos	Methyl 3-indolyacetate	C11 H11 N O2	189.07943	6.769	--	--	190.08673
Com_42993_pos	ELK	C17 H32 N4 O6	388.23142	2.215	--	--	389.23856
Com_43305_pos	Luotonin A	C18 H11 N3 O	285.08937	1.974	--	--	286.09665
Com_43437_pos	(S)-AL 8810	C24 H31 F O4	384.21634	11.606	--	--	385.2229
Com_43499_pos	Lysopc 20:0	C28 H58 N O7 P	551.39733	15.334	--	--	552.4046
Com_43639_pos	PE (10:0/16:4)	C31 H54 N O8 P	599.35972	15.618	--	--	600.36707
Com_43675_pos	PC (14:1e/8:0)	C30 H60 N O7 P	577.41199	15.914	--	--	578.41956
Com_43696_pos	4-[4-(1-phenyl-1H-1,2,4-triazol-5-yl)-1,3-thiazol-2-yl]pyridine	C16 H11 N5 S	305.07337	3.296	--	--	306.08057
Com_43760_pos	1-(3-phenylpropanoyl)-4-piperidinecarboxylic acid	C15 H19 N O3	261.13212	1.299	--	--	262.13898
Com_43771_pos	Lidocaine N-oxide	C14 H22 N2 O2	250.16776	1.365	--	--	251.17503
Com_43850_pos	1-adamantyl(piperidino)methanone	C16 H25 N O	247.19359	13.241	--	--	248.20085
Com_44003_pos	Glu-Val-Phe	C19 H27 N3 O6	393.18957	8.521	--	--	394.19705
Com_44087_pos	ANH	C13 H20 N6 O5	170.07321	10.164	--	--	171.08051
Com_44359_pos	1-(3-methoxy-2-nitrostyryl)pyrrolidine	C13 H16 N2 O3	248.11579	6.188	--	--	249.12323
Com_44467_pos	4-[(2-thienylmethylidene)amino]benzoic acid	C12 H9 N O2 S	231.03545	12.219	--	--	232.04272
Com_44762_pos	N-(2-quinolinyl)-2,3-dihydro-1-benzofuran-6-carboxamide	C18 H14 N2 O2	290.10456	10.969	--	--	291.11258

Continuation of Supplementary Table 2

Compound_ID	Name	Formula	Molecular Weight	RT [min]	Kegg_ID	HMDB_ID	m/z
Com_44858_pos	JWH 018 N-(4,5-epoxypentyl) analog	C24 H21 N O2	355.16303	6.076	--	--	356.17038
Com_44909_pos	2-phenyl-4H-furo[2,3-h]chromen-4-one	C17 H10 O3	262.06313	9.213	--	--	263.0704
Com_44935_pos	(1E)-5-hydroxy-1,7-diphenylhept-1-en-3-one	C19 H20 O2	264.17098	11.058	--	--	303.13419
Com_45053_pos	5-[(2-hydroxybenzylidene)amino]-2-(2-methoxyethoxy)benzoic acid	C17 H17 N O5	315.11392	5.396	--	--	316.12146
Com_45250_pos	Dl-3-Hydroxy-kynurenine	C10 H12 N2 O4	224.07964	7.15	--	--	225.08693
Com_45702_pos	2-Amino-6-methylmercaptopurine	C6 H7 N5 S	181.0422	12.119	--	--	182.04932
Com_45713_pos	6,7,8-trimethoxy-2-(2-phenoxy-3-pyridyl)-4H-3,1-benzoxazin-4-one	C22 H18 N2 O6	406.11095	5.783	--	--	407.11771
Com_45731_pos	4-(3,4-dihydro-2H-1,5-benzodioxepin-7-ylamino)-4-oxobutanoic acid	C13 H15 N O5	247.08205	9.258	--	--	248.08882
Com_45738_pos	3-hydroxy-2-octylpentanedioic acid	C13 H24 O5	220.16782	7.95	--	--	221.1749
Com_45794_pos	4-methoxy-9-(3-methylbut-2-en-1-yl)-7H-furo[3,2-g]chromen-7-one	C17 H16 O4	284.10472	11.333	--	--	285.11185
Com_45940_pos	3-[(4-chlorophenyl)thio]-1-phenylprop-2-en-1-one	C15 H11 Cl O S	296.00093	2.661	--	--	297.00821
Com_45999_pos	2,4-dimethyl-2,3-dihydrochromeno[4,3-c]pyrazol-3-one	C12 H10 N2 O2	214.07445	7.932	--	--	215.08185
Com_46068_pos	Sodium [dodecanoyl(methyl)amino]acetate	C15 H29 N O3	271.2146	10.949	--	--	272.22186
Com_46069_pos	5-phenyl-2,3-dihydro-1H-1,4-benzodiazepin-2-one	C15 H12 N2 O	258.07391	6.511	--	--	259.08124
Com_46082_pos	Cnidioside A	C17 H20 O9	390.09499	10.643	--	--	391.10226
Com_46235_pos	N-Acetyl-DL-tryptophan	C13 H14 N2 O3	246.10058	7.969	--	--	247.10788

Continuation of Supplementary Table 2

Compound_ID	Name	Formula	Molecular Weight	RT [min]	Kegg_ID	HMDB_ID	m/z
Com_46359_pos	Bisphenol TMC	C21 H26 O2	327.21583	6.824	--	--	328.22308
Com_46619_pos	7-chloro-2-methyl-3-(4-pyridylmethyl)-3,4-dihydroquinazolin-4-one	C15 H12 Cl N3 O	285.06469	7.392	--	--	286.07205
Com_46689_pos	Ribulose-5-phosphate	C5 H11 O8 P	230.01927	1.361	--	--	231.02655
Com_46917_pos	HexCer-NDS (d18:0/15:0)	C39 H77 N O8	687.5685	15.071	--	--	688.57629
Com_46922_pos	GNH	C12 H18 N6 O5	326.13555	8.834	--	--	327.14297
Com_46994_pos	5-(hydroxymethyl)-4-methoxy-2,5-dihydrofuran-2-one	C6 H8 O4	166.02435	1.176	--	--	167.03159
Com_47119_pos	KMH	C17 H30 N6 O4 S	396.19152	14	--	--	397.19839
Com_47165_pos	4-OHE1-1-N3Ade	C23 H25 N5 O3	419.19467	7.525	--	--	420.20193
Com_47170_pos	benzaldehyde 1-[4-(3-pyridyl)pyrimidin-2-yl]hydrazone	C16 H13 N5	275.1191	5.254	--	--	276.1265
Com_47416_pos	Pro-Leu	C11 H20 N2 O3	228.14784	9.579	--	--	229.15433
Com_47528_pos	FNK	C19 H29 N5 O5	389.20475	6.685	--	--	390.21194
Com_47534_pos	5-(4-benzylpiperazino)-2,4(1H,3H)-pyrimidinedione	C15 H18 N4 O2	286.13921	7.734	--	--	287.14648
Com_47580_pos	DL-α-Tocopherol	C29 H50 O2	430.38135	15.142	--	--	431.38919
Com_47873_pos	Cer-NS (d14:1/18:1)	C32 H61 N O3	507.46681	16.653	--	--	508.4744
Com_47906_pos	4-amino-2-(2,4-dimethoxyanilino)-5-pyrimidinecarbonitrile	C13 H13 N5 O2	271.1054	7.816	--	--	272.11252
Com_48270_pos	Artesunate	C19 H28 O8	406.16095	8.838	--	--	407.16809
Com_48317_pos	Epinastine	C16 H15 N3	249.12247	8.078	--	--	250.1299
Com_48367_pos	SM (d26:1/12:0)	C43 H87 N2 O6 P	758.6434	14.324	--	--	759.64954

Continuation of Supplementary Table 2

Compound_ID	Name	Formula	Molecular Weight	RT [min]	Kegg_ID	HMDB_ID	m/z
Com_48694_pos	8,8-dimethyl-2-phenyl-4H,8H-pyrano[2,3-h]chromen-4-one	C20 H16 O3	304.1059	6.761	--	--	305.11313
Com_48753_pos	6-methyl-7-nitro-2,3-dihydro-1,4-benzodioxine	C9 H9 N O4	177.04275	6.59	--	--	178.05002
Com_48972_pos	6-Aminonicotinamide	C6 H7 N3 O	137.05915	6.161	--	--	138.06644
Com_49131_pos	3-[(4-hydroxyphenyl)methyl]-octahydropyrrolo[1,2-a]pyrazine-1,4-dione	C14 H16 N2 O3	260.11625	7.23	--	--	261.1232
Com_49242_pos	1-[5-(2-phenyleth-1-ynyl)-2-thienyl]ethan-1-one oxime	C14 H11 N O S	241.05805	1.505	--	--	242.06532
Com_49244_pos	6-phenyl-1,2,3,4-tetrahydro-2,5-benzodiazocin-1-one	C16 H14 N2 O	288.06359	12.4	--	--	289.07095
Com_49293_pos	(2R,3S,4S,5R,6R)-2-(hydroxymethyl)-6-(propan-2-yloxy)oxane-3,4,5-triol	C9 H18 O6	244.09241	6.497	--	--	245.09972
Com_49328_pos	2-cyano-2-(2,3-dihydro-1H-inden-1-yliden)acetamide	C12 H10 N2 O	198.07958	7.702	--	--	199.08708
Com_49462_pos	3-(3,4-dimethoxyphenyl)-1-(2-hydroxy-4,6-dimethoxyphenyl)propan-1-one	C19 H22 O6	368.12453	15.116	--	--	369.13181
Com_49683_pos	mesityl (4-methylphenyl) sulfone	C16 H18 O2 S	274.09944	12.378	--	--	275.10687
Com_49806_pos	(5E)-7-methylidene-10-oxo-4-(propan-2-yl)undec-5-enoic acid	C15 H24 O3	274.15459	10.764	--	--	275.16187
Com_50007_pos	2-{2-oxo-2H,8H,9H-furo[2,3-h]chromen-8-yl}propan-2-yl acetate	C16 H16 O5	310.07979	8.832	--	--	311.08707
Com_50100_pos	PD 0200347	C10 H17 N3 O2	211.13227	6.884	--	--	212.13956
Com_50177_pos	2,3-di(acetyloxy)-3-cyano-1-[1,2-di(acetyloxy)ethyl]propyl acetate	C16 H21 N O10	365.13132	6.901	--	--	366.13907

Continuation of Supplementary Table 2

Compound_ID	Name	Formula	Molecular Weight	RT [min]	Kegg_ID	HMDB_ID	m/z
Com_50312_pos	2,2-dimethyl-6,7-di[(4-nitrobenzyl)oxy]chroman-4-one	C25 H22 N2 O8	478.13856	2.267	--	--	479.14606
Com_50323_pos	2-[(7-methylthieno[3,2-d]pyrimidin-4-yl)thio]ethanohydrazide	C9 H10 N4 O S2	276.01129	9.089	--	--	277.01859
Com_50401_pos	3-ethyl-4-hydroxy-1-methyl-1,2-dihydroquinolin-2-one	C12 H13 N O2	203.09472	7.345	--	--	204.10197
Com_50413_pos	17beta-Trenbolone	C18 H22 O2	270.15761	6.766	--	--	271.16489
Com_50925_pos	N-METHYL (-)EPHEDRINE	C11 H17 N O	179.13071	2.714	--	--	180.13799
Com_51171_pos	Tauro-alpha-Muricholic acid sodium salt	C26 H44 N Na O7 S	537.27563	14.434	--	--	538.28217
Com_51212_pos	GLK	C14 H28 N4 O4	316.21105	2.229	--	--	317.21814
Com_51271_pos	2-chloro-N-[4-(4-methylpiperazino)phenyl]benzamide	C18 H20 Cl N3 O	329.12987	6.021	--	--	330.13724
Com_51622_pos	Clinafloxacin	C17 H17 Cl F N3 O3	347.08253	1.299	--	--	348.08981
Com_51660_pos	3-{[(hexylamino)carbonyl]amino}-3-phenylpropanoic acid	C16 H24 N2 O3	292.17866	8.959	--	--	293.18573
Com_51727_pos	Lumefantrine	C30 H32 Cl3 N O	509.14644	8.918	--	--	510.15359
Com_51948_pos	2-chloro-6-(1,4-thiazinan-4-yl)benzonitrile	C11 H11 Cl N2 S	260.01536	10.53	--	--	261.02264
Com_52015_pos	HPH	C17 H23 N7 O4	371.17278	9.195	--	--	372.18005
Com_52387_pos	(2E,4E)-N-(2-methylpropyl)dodeca-2,4-dienamide	C16 H29 N O	251.22475	13.782	--	--	252.23203
Com_52410_pos	4,7-diphenyl[1,10]phenanthroline	C24 H16 N2	332.12587	7.846	--	--	333.13342
Com_52521_pos	4-(1-adamantyl)-2-methyl-1,3-thiazole	C14 H19 N S	233.12634	2.032	--	--	234.13377

Continuation of Supplementary Table 2

Compound_ID	Name	Formula	Molecular Weight	RT [min]	Kegg_ID	HMDB_ID	m/z
Com_52598_pos	FMK	C20 H32 N4 O4 S	406.19829	8.754	--	--	407.20557
Com_52897_pos	methyl 3,4,5-trihydroxycyclohex-1-ene-1-carboxylate	C8 H12 O5	210.05284	8.181	--	--	211.05997
Com_52904_pos	1-{[5-(4-chlorophenyl)-4H-1,2,4-triazol-3-yl]thio}acetone	C11 H10 Cl N3 O S	267.02	8.868	--	--	268.02716
Com_52919_pos	2-(4-methylphenyl)-2-oxoethyl thiocyanate	C10 H9 N O S	191.04046	7.091	--	--	192.04778
Com_53004_pos	Cer-NS (d14:1/18:2)	C32 H59 N O3	505.45054	16.23	--	--	506.45761
Com_53234_pos	Uracil 1-beta-D-arabinofuranoside	C9 H12 N2 O6	244.06905	2.228	--	--	245.07635
Com_53396_pos	6-(3-hydroxybutan-2-yl)-5-(hydroxymethyl)-4-methoxy-2H-pyran-2-one	C11 H16 O5	210.08904	8.335	--	--	211.09636
Com_53584_pos	N-Carbamyl-L-glutamicacid	C6 H10 N2 O5	190.0587	1.294	--	--	191.06584
Com_53674_pos	4-(4-methoxyphenyl)-6-pyridin-4-yl-1,3,5-triazin-2(3H)-one	C15 H12 N4 O2	280.09196	8.5	--	--	281.09888
Com_53726_pos	2-[2-(benzylsulfanyl)-4-phenyl-1H-imidazol-1-yl]-1-phenyl-1-ethanone	C24 H20 N2 O S	384.13805	10.143	--	--	385.14532
Com_53783_pos	7-hydroxy-5-methoxy-2-phenyl-3,4-dihydro-2H-1-benzopyran-4-one	C16 H14 O4	146.03573	12.188	--	--	293.07874
Com_54017_pos	FNH	C19 H24 N6 O5	454.12886	9.158	--	--	455.13611
Com_54078_pos	5-methyl-N'-[3-(trifluoromethyl)benzoyl]-3-isoxazolecarbohydrazide	C13 H10 F3 N3 O3	335.0464	7.763	--	--	336.05368
Com_54132_pos	1-(4-hydroxyphenyl)propane-1,2-diol	C9 H12 O3	190.06334	11.202	--	--	191.07092
Com_54248_pos	Azilsartan	C25 H20 N4 O5	478.12665	10.222	--	--	479.13446
Com_54253_pos	PC (18:1/20:5)	C46 H80 N O8 P	805.56493	16.383	--	--	806.5722
Com_54330_pos	Diethyleneglycol diacetate	C8 H14 O5	190.08421	9.476	--	--	191.09131

Continuation of Supplementary Table 2

Compound_ID	Name	Formula	Molecular Weight	RT [min]	Kegg_ID	HMDB_ID	m/z
Com_54421_pos	Sitagliptin	C16 H15 F6 N5 O	814.24627	11.05	--	--	815.25305
Com_54427_pos	KQH	C17 H29 N7 O5	393.21493	5.817	--	--	394.22235
Com_54503_pos	Cer-NS (d14:1/16:1)	C30 H57 N O3	479.43494	16.305	--	--	480.44168
Com_54675_pos	SDB-006 N-phenyl analog	C20 H22 N2 O	306.1692	8.079	--	--	307.17636
Com_54730_pos	1-[2-(2,5-dimethyl-1H-pyrrol-1-yl)-4-nitrophenyl]-1H-imidazole	C15 H14 N4 O2	260.1258	7.85	--	--	283.11505
Com_54779_pos	N-gamma-Acetyl-N-2-Formyl-5-Methoxykynurenamine	C13 H16 N2 O4	264.11067	5.536	--	--	265.11792
Com_54977_pos	(2E)-6-hydroxy-2-methyl-6-(4-methylphenyl)hept-2-enoic acid	C15 H20 O3	270.12122	6.983	--	--	271.12833
Com_55064_pos	JNJ-1661010	C19 H19 N5 O S	365.13192	1.353	--	--	366.13879
Com_55068_pos	3-[3-(beta-D-Glucopyranosyloxy)-2-hydroxyphenyl]propanoic acid	C15 H20 O9	344.11036	6.518	--	--	345.11758
Com_55205_pos	TNK	C14 H27 N5 O6	361.19208	6.679	--	--	362.19937
Com_55332_pos	dodecyl [(tetrahydrofuran-2-ylmethyl)amino]methanedithioate	C18 H35 N O S2	345.21387	6.507	--	--	346.22119
Com_55477_pos	6-[2-(2H-1,3-benzodioxol-5-yl)ethyl]-4-methoxy-2H-pyran-2-one	C15 H14 O5	274.08585	10.127	--	--	297.07507
Com_55803_pos	WPH	C22 H26 N6 O4	460.18419	9.969	--	--	461.19147
Com_55818_pos	Melicopidine	C17 H15 N O5	335.08034	9.765	--	--	336.08762
Com_55826_pos	Zolpidem-d6	C19 H15 [2]H6 N3 O	313.20249	9.112	--	--	314.20996
Com_55870_pos	3-[1-(phenylsulfonyl)-2-pyrrolidinyl]pyridine	C15 H16 N2 O2 S	288.0888	16.737	--	--	289.09583

Continuation of Supplementary Table 2

Compound_ID	Name	Formula	Molecular Weight	RT [min]	Kegg_ID	HMDB_ID	m/z
Com_56254_pos	N1-isopropyl-2-(phenylthio)benzamide	C16 H17 N O S	293.08779	8.169	--	--	294.09485
Com_56531_pos	4-[2-(4-chlorophenyl)diaz-1-enyl]-2-methyl-6-(piperidinomethyl)phenol	C19 H22 Cl N3 O	343.14193	11.185	--	--	344.14926
Com_56604_pos	Kinetin 9-riboside	C15 H17 N5 O5	347.12317	5.41	--	--	348.13077
Com_56613_pos	5-chloro-6-(trifluoromethyl)-1,3-dihydro-2H-benzimidazole-2-thione	C8 H4 Cl F3 N2 S	251.97191	8.113	--	--	252.97914
Com_57104_pos	YQH	C20 H26 N6 O6	446.19186	8.211	--	--	447.19864
Com_57114_pos	1-methyl-3,5-di(1-naphthylmethylidene)piperidin-4-one	C28 H23 N O	389.17975	8.853	--	--	390.18692
Com_57143_pos	Milbemycin A3 oxime	C31 H43 N O7	541.29991	10.702	--	--	542.30719
Com_57290_pos	6-[(4-methylphenyl)sulfonyl]-6-azabicyclo[3.2.1]octane	C14 H19 N O2 S	303.07198	1.248	--	--	304.07935
Com_57356_pos	N1-(2,4-dimethylphenyl)-2-(2,4-dichlorophenoxy)propanamide	C17 H17 Cl2 N O2	337.06182	7.737	--	--	338.06909
Com_57446_pos	5-[(benzoyloxy)methyl]-4,5,6-trihydroxycyclohex-2-en-1-yl benzoate	C21 H20 O7	406.10536	12.302	--	--	407.1127
Com_57915_pos	4-(allyloxy)-1,2-dihydroquinolin-2-one	C12 H11 N O2	201.0791	7.981	--	--	202.08649
Com_58081_pos	3-(2-methylpropyl)-octahydropyrrolo[1,2-a]pyrazine-1,4-dione	C11 H18 N2 O2	232.12145	8.631	--	--	233.12877
Com_58139_pos	N1-[2-(2-pyridyl)ethyl]-2-aminobenzamide	C14 H15 N3 O	263.10145	3.128	--	--	264.10876
Com_58291_pos	8-[(4-phenethyl-1,4-diazepan-1-yl)sulfonyl]quinoline	C22 H25 N3 O2 S	395.16866	6.747	--	--	396.17471
Com_58368_pos	5-S-cysteinyldopa	C12 H16 N2 O6 S	316.07205	15.106	--	--	317.07932
Com_58483_pos	YMH	C20 H27 N5 O5 S	471.16493	8.465	--	--	472.17239

Continuation of Supplementary Table 2

Compound_ID	Name	Formula	Molecular Weight	RT [min]	Kegg_ID	HMDB_ID	m/z
Com_58686_pos	DRH	C16 H26 N8 O6	448.18235	7.296	--	--	449.18961
Com_58789_pos	3,4,10,11-tetramethoxy-7,8,12b,13-tetrahydro-5H-6-azatetraphene	C21 H25 N O4	355.17843	11.585	--	--	356.18585
Com_59164_pos	methyl 2,5-dimethyl-1H-pyrrole-3-carboxylate	C8 H11 N O2	153.07935	5.694	--	--	154.08662
Com_59238_pos	(4-methoxyphenyl)(4-nitrophenyl)methanone	C14 H11 N O4	275.07934	9.171	--	--	276.08655
Com_59485_pos	6β-Oxycodol N-oxide	C18 H23 N O5	333.15747	8.812	--	--	334.16486
Com_59833_pos	4-[2-(3-pyridyl)-4-quinolyl]morpholine	C18 H17 N3 O	291.13327	7.337	--	--	292.14056
Com_59878_pos	HQH	C17 H24 N8 O5	460.18525	6.801	--	--	461.19342
Com_60590_pos	trans-Clovamide	C18 H17 N O7	359.10377	2.105	--	--	360.11105
Com_60676_pos	Milbemycin A4 oxime	C32 H45 N O7	577.2957	10.251	--	--	578.30298
Com_60754_pos	QMK	C16 H31 N5 O5 S	405.20012	6.373	--	--	406.2074
Com_60805_pos	N-(1-adamantyl)-N'-(2-chlorobenzyl)thiourea	C18 H23 Cl N2 S	334.12761	5.963	--	--	335.13498
Com_60845_pos	EPK	C16 H28 N4 O6	394.18485	7.021	--	--	395.19162
Com_60892_pos	3-aminobenzo[4,5]imidazo[2,1-b][1,3]thiazole-2-carbonitrile	C10 H6 N4 S	214.02767	8.312	--	--	215.03516
Com_60918_pos	7-alpha-carboxy-17-alpha-carboxyethylandrostan lactone phenyl ester	C28 H38 O5	454.27343	10.989	--	--	455.28085
Com_61022_pos	(±)13-HpODE	C18 H32 O4	334.21448	10.127	--	--	335.22171
Com_61142_pos	1-(4-benzylpiperazino)-2-(pyridin-2-ylamino)propan-1-one	C19 H24 N4 O	340.17332	7.915	--	--	341.18039
Com_61271_pos	ethyl 4-amino-2-(methylsulfanyl)-1,3-thiazole-5-carboxylate	C7 H10 N2 O2 S2	218.01923	7.843	--	--	219.02652
Com_61555_pos	4-(4-methoxyphenyl)-2-(methylthio)pyrimidine	C12 H12 N2 O S	232.06223	1.088	--	--	233.06935

Continuation of Supplementary Table 2

Compound_ID	Name	Formula	Molecular Weight	RT [min]	Kegg_ID	HMDB_ID	m/z
Com_61610_pos	N-(1-benzylpiperidin-4-yl)-5-methoxy-1H-indole-2-carboxamide	C22 H25 N3 O2	363.1887	2.01	--	--	364.19601
Com_61641_pos	Obscurolide A1	C15 H17 N O5	313.0925	8.574	--	--	314.09961
Com_61780_pos	SMK	C14 H28 N4 O5 S	364.17528	9.762	--	--	365.18262
Com_62109_pos	WKK	C23 H36 N6 O4	460.27803	7.762	--	--	461.28546
Com_62429_pos	N-(1-benzyl-4-piperidinyl)-4-(1H-pyrazol-1-yl)benzamide	C22 H24 N4 O	398.15156	6.578	--	--	399.15875
Com_62557_pos	4-(4-methylphenyl)-2-phenyl-1,3-thiazole	C16 H13 N S	229.09495	6.784	--	--	230.10219
Com_62788_pos	RNH	C16 H27 N9 O5	447.19749	6.15	--	--	448.20486
Com_62817_pos	D-δ-Tocopherol	C27 H46 O2	402.34998	16.224	--	--	403.35721
Com_62933_pos	1-ethyl 4-(2-oxo-1,2-diphenylethyl) succinate	C20 H20 O5	362.11085	7.738	--	--	363.11798
Com_63128_pos	(3S,9aS)-3-benzyl-octahydro-1H-pyrido[1,2-a]pyrazin-1-one	C15 H20 N2 O	266.13811	6.848	--	--	267.14532
Com_63226_pos	2-piperidinobenzoic acid	C12 H15 N O2	205.11034	6.99	--	--	206.11758
Com_63508_pos	VMK	C16 H32 N4 O4 S	358.20815	7.726	--	--	359.21542
Com_63557_pos	4-(benzoylamino)-3-hydroxybutanoic acid	C11 H13 N O4	223.08448	7.327	--	--	224.09181
Com_63698_pos	3-(3-morpholinopropyl)-2-(2-pyridinyl)-2,3-dihydro-4(1H)-quinazolinone	C20 H24 N4 O2	352.18722	9.632	--	--	353.19556
Com_63790_pos	trans-3-Indoleacrylic acid	C11 H9 N O2	187.06705	7.007	--	--	188.07407
Com_63828_pos	CYM-5442	C23 H27 N3 O4	409.20063	9.923	--	--	410.20795
Com_63921_pos	N'3-(2-thienylmethylene)-5-(1-hexynyl)-3-pyridinecarbohydrazide	C17 H17 N3 O S	311.1154	1.72	--	--	312.12299
Com_63927_pos	dimethyl 2-(3-nitro-2-pyridyl)malonate	C10 H10 N2 O6	254.05552	7.592	--	--	255.06265

Continuation of Supplementary Table 2

Compound_ID	Name	Formula	Molecular Weight	RT [min]	Kegg_ID	HMDB_ID	m/z
Com_64210_pos	Oxohongdenafil	C25 H32 N6 O4	480.24859	9.537	--	--	481.25586
Com_64355_pos	5-Fluoro-2-[(3S)-1-(2-fluorobenzyl)-3-pyrrolidinyl]-1H-benzimidazole	C18 H17 F2 N3	626.28379	6.151	--	--	314.14917
Com_64945_pos	2-(4-chlorophenyl)-5-(4-phenylbuta-1,3-dienyl)-2H-1,2,3,4-tetraazole	C17 H13 Cl N4	308.0739	6.901	--	--	309.08124
Com_65025_pos	2-{[5-(ethylsulfonyl)-2-hydroxyanilino]carbonyl}benzoic acid	C16 H15 N O6 S	331.05113	9.063	--	--	332.05841
Com_65464_pos	N-(4-piperidinophenyl)-2-thiophenecarboxamide	C16 H18 N2 O S	324.06901	7.791	--	--	325.07614
Com_65789_pos	J147	C18 H17 F3 N2 O2	350.12584	8.596	--	--	351.13312
Com_65879_pos	[4-(7-chloroquinolin-4-yl)piperazino](phenyl)methanone	C20 H18 Cl N3 O	313.15446	10.025	--	--	352.11761
Com_65907_pos	2-Thio-acetyl MAGE	C21 H42 O3 S	396.26259	7.486	--	--	397.26987
Com_66042_pos	2,5-bis(4-hydroxy-3-methoxyphenyl)-3,4-dimethyloxolan-3-ol	C20 H24 O6	382.14213	13.345	--	--	383.14926
Com_66136_pos	LMH	C17 H29 N5 O4 S	421.17381	7.781	--	--	422.18079
Com_66375_pos	5-oxo-3-phenyl-5-(2-quinolinylamino)pentanoic acid	C20 H18 N2 O3	356.10842	8.872	--	--	357.11591
Com_66438_pos	4-fluoro-N-(4-piperidinophenyl)benzenesulfonamide	C17 H19 F N2 O2 S	334.11533	7.253	--	--	335.12234
Com_66515_pos	6-methoxy-2-phenyl-3,4-dihydro-2H-1-benzopyran-4-one	C16 H14 O3	254.0939	9.19	--	--	255.1013
Com_66695_pos	N1-[4-(acetylamino)phenyl]-2,2-dimethylcyclopropane-1-carboxamide	C14 H18 N2 O2	246.13541	6.904	--	--	247.14268

Continuation of Supplementary Table 2

Compound_ID	Name	Formula	Molecular Weight	RT [min]	Kegg_ID	HMDB_ID	m/z
Com_66755_pos	1-(3-acetyl-2,4,6-trihydroxyphenyl)ethan-1-one	C10 H10 O5	210.05289	6.457	--	--	211.06027
Com_66936_pos	AKB48 N-(4-fluorobenzyl) analog	C25 H26 F N3 O	403.2075	1.38	--	--	404.21484
Com_66950_pos	N-(2,3-dihydro-1,4-benzodioxin-6-yl)-N'-(1H-indol-5-yl)thiourea	C17 H15 N3 O2 S	325.09096	8.448	--	--	326.09824
Com_66965_pos	PC (14:0e/20:2)	C42 H82 N O7 P	743.58342	16.048	--	--	744.5907
Com_67349_pos	(2E)-N-(4-acetamidobutyl)-3-(4-hydroxy-3-methoxyphenyl)prop-2-enamide	C16 H22 N2 O4	306.15806	8.743	--	--	307.16544
Com_67439_pos	DL-2-(acetylamino)-3-phenylpropanoic acid	C11 H13 N O3	189.07898	7.415	--	--	190.0862
Com_67444_pos	N,5-diphenyl-1,3,4-oxadiazole-2-carboxamide	C15 H11 N3 O2	265.08057	10.607	--	--	266.08783
Com_68216_pos	3,4-dihydro-4-oxo-2H-1,3-benzoxazine-2-spiro-4'-(1'-ethylpiperidine)	C20 H26 N2 O6 S	422.15238	11.273	--	--	423.15915
Com_68349_pos	Tetranor-12(S)-HETE	C16 H26 O3	288.16863	6.344	--	--	289.17606
Com_68368_pos	Solvent orange 2	C17 H14 N2 O	262.11061	11.821	--	--	263.11774
Com_68434_pos	[4-(2-methoxyphenyl)piperazino](4-methylphenyl)methanone	C19 H22 N2 O2	310.16689	9.107	--	--	311.17416
Com_68564_pos	ethyl 3-hydroxy-4,6-dimethoxy-2-oxoindoline-3-carboxylate	C13 H15 N O6	281.08969	6.436	--	--	282.09683
Com_68722_pos	4-OHE2-1-N3Ade	C23 H27 N5 O3	421.20975	6.276	--	--	422.21713
Com_69007_pos	(4-methoxyphenyl)(4-phenyl-3-quinolinyl)methanone	C23 H17 N O2	339.13146	6.438	--	--	340.13873
Com_69054_pos	4-methoxy-9-(2-methylbut-3-en-2-yl)-7H-furo[3,2-g]chromen-7-one	C17 H16 O4	284.10461	11.192	--	--	285.11191
Com_69205_pos	ethyl 3-oxo-5,6-diphenyl-2,3-dihydropyridazine-4-carboxylate	C19 H16 N2 O3	358.06859	11.946	--	--	359.07587

Continuation of Supplementary Table 2

Compound_ID	Name	Formula	Molecular Weight	RT [min]	Kegg_ID	HMDB_ID	m/z
Com_69426_pos	1H-indol-3-yl(pyridin-2-yl)methanol	C14 H12 N2 O	206.08428	12.227	--	--	207.09157
Com_69866_pos	para-methoxy-Butyryl fentanyl-d7	C24 H25 [2]H7 N2 O2	387.29326	15.982	--	--	388.30054
Com_70241_pos	3-(1-benzylpiperidin-4-yl)-3H-[1,2,3]triazolo[4,5-b]pyridine	C17 H19 N5	293.16265	6.194	--	--	294.16992
Com_70257_pos	INK	C16 H31 N5 O5	373.2318	2.033	--	--	374.23926
Com_70436_pos	GPK	C13 H24 N4 O4	322.16414	6.49	--	--	323.17142
Com_70524_pos	3-hydroxyquinuclidine-3-carbonitrile hydrochloride	C8 H12 N2 O	152.09556	6.34	--	--	153.10289
Com_70557_pos	3-bromo-9-(piperidinomethyl)-9H-carbazole	C18 H19 Br N2	342.24114	13.39	--	--	343.2486
Com_70661_pos	2-{[methyl(2,3,4,5,6-pentahydroxyhexyl)amino]methylidene}malononitrile	C11 H17 N3 O5	271.11623	2.471	--	--	272.1235
Com_71196_pos	EMH	C16 H25 N5 O6 S	397.14833	5.868	--	--	398.15503
Com_71302_pos	N1-[(2,6-dimethylmorpholino)carbonyl]-3-chloro-4-fluorobenzamide	C14 H16 Cl F N2 O3	314.08508	6.807	--	--	315.09247
Com_71335_pos	EKK	C17 H33 N5 O6	403.24338	6.669	--	--	404.25079
Com_71542_pos	TQH	C15 H24 N6 O6	384.17598	8.87	--	--	385.18326
Com_71878_pos	methyl 5-[(5-phenylthieno[2,3-d]pyrimidin-4-yl)oxy]-2-furoate	C18 H12 N2 O4 S	352.05816	10.081	--	--	353.06546
Com_71915_pos	4-morpholino-3-nitrobenzene-1-sulfonamide	C10 H13 N3 O5 S	269.04649	5.591	--	--	270.0538
Com_72117_pos	4,6-dimethyl-2,7-diphenyl-3,7-dihydro-2H-pyrazolo[3,4-b]pyridin-3-one	C20 H17 N3 O	315.14326	2.157	--	--	316.15048
Com_72282_pos	4-benzylidene-2-phenyltetrahydroisoxazole-3,5-dione	C16 H11 N O3	265.07349	8.26	--	--	266.08078

Continuation of Supplementary Table 2

Compound_ID	Name	Formula	Molecular Weight	RT [min]	Kegg_ID	HMDB_ID	m/z
Com_72299_pos	7-(3,4-dihydroxyphenyl)-5-hydroxy-1-(4-hydroxyphenyl)heptan-3-one	C19 H22 O5	352.13115	12.758	--	--	353.13843
Com_72412_pos	N1-(1-benzyl-4-piperidyl)-4-chlorobenzene-1-sulfonamide	C18 H21 Cl N2 O2 S	364.09498	1.414	--	--	365.10168
Com_72414_pos	Fluprostenol	C23 H29 F3 O6	458.19262	11.827	--	--	459.19968
Com_72431_pos	R-1 Methanandamide phosphate	C23 H40 N O5 P	463.24362	5.801	--	--	464.25089
Com_72622_pos	VPH	C16 H25 N5 O4	351.19054	3.433	--	--	352.19781
Com_72723_pos	4-butyl-4-(hydroxymethyl)-1,2-diphenylpyrazolidine-3,5-dione	C20 H22 N2 O3	360.14622	7.205	--	--	361.15378
Com_73680_pos	2-(cyclopropylcarbonyl)-3-(4-fluoroanilino)acrylonitrile	C13 H11 F N2 O	268.03736	8.785	--	--	269.04437
Com_73802_pos	N-(1,3-benzodioxol-5-ylmethyl)-N'-(1-benzothiophen-3-yl)urea	C17 H14 N2 O3 S	364.02428	6.255	--	--	365.03156
Com_74155_pos	Desmethylmianserin	C17 H18 N2	250.14274	5.024	--	--	251.15005
Com_74452_pos	7-(2-thienyl)[1,2,4]triazolo[4,3-a]pyrimidine	C9 H6 N4 S	224.01618	5.347	--	--	225.02345
Com_74807_pos	2-[(2-amino-6-methylpyrimidin-4-yl)thio]-4,6-dimethylnicotinonitrile	C13 H13 N5 S	249.10377	7.836	--	--	250.11119
Com_74843_pos	N-(4-fluorophenyl)-N'-(2-piperidinophenyl)urea	C18 H20 F N3 O	335.14015	2.193	--	--	336.14743
Com_74961_pos	farensyl diphosphate	C15 H37 N3 O7 P2	433.21042	11.639	--	--	434.21713
Com_74978_pos	1-(3,4-dimethylphenyl)-3-piperidinopyrrolidine-2,5-dione	C17 H22 N2 O2	286.16397	4.931	--	--	287.17126

Continuation of Supplementary Table 2

Compound_ID	Name	Formula	Molecular Weight	RT [min]	Kegg_ID	HMDB_ID	m/z
Com_75048_pos	3-(3-chlorophenyl)-4-phenyl-1,2,4-oxadiazol-5(4H)-one	C14 H9 Cl N2 O2	289.06166	3.636	--	--	290.06894
Com_75116_pos	3-(tert-butyl)-1-methyl-N-(6-quinoxalinyl)-1H-pyrazole-5-carboxamide	C17 H19 N5 O	309.15755	8.816	--	--	310.16483
Com_75165_pos	4-oxo-4-(5,6,7,8-tetrahydronaphthalen-1-ylamino)but-2-enoic acid	C14 H15 N O3	245.10516	6.596	--	--	246.11252
Com_75519_pos	4-(4-methylbenzyl)-1lambda~6~,4-thiazinane-1,1-dione	C12 H17 N O2 S	277.05858	8.67	--	--	278.06586
Com_75664_pos	5-Fluoro-2-[(3S)-1-(2-methylbenzyl)-3-pyrrolidinyl]-1H-benzimidazole	C19 H20 F N3	309.1688	8.036	--	--	310.17612
Com_75821_pos	EMK	C16 H30 N4 O6 S	388.17414	8.489	--	--	389.18152
Com_76020_pos	3-hydroxy-3-(2-pyridylmethyl)indolin-2-one	C14 H12 N2 O2	240.08991	8.47	--	--	241.09731
Com_76230_pos	N-[3-(1H-imidazol-1-yl)propyl]-5-methoxy-1H-indole-2-carboxamide	C16 H18 N4 O2	596.28089	5.713	--	--	299.14761
Com_76569_pos	5-hydroxy-6,7-dimethoxy-2-phenyl-4H-chromen-4-one	C17 H14 O5	280.07692	9.403	--	--	281.0842
Com_76670_pos	6,7,8-trimethoxy-2-[3-(trifluoromethyl)phenyl]-4H-3,1-benzoxazin-4-one	C18 H14 F3 N O5	381.08248	7.308	--	--	382.08975
Com_76688_pos	N1-(2-pyridyl)-2-(3,5-difluorophenyl)acetamide	C13 H10 F2 N2 O	248.07728	6.124	--	--	249.08453
Com_76732_pos	N,N'-Di-2-naphthyl-p-phenylenediamine	C26 H20 N2	360.15604	10.155	--	--	361.16373
Com_77125_pos	Dehydroaripiprazole	C23 H25 Cl2 N3 O2	445.1378	8.481	--	--	446.14508

Continuation of Supplementary Table 2

Compound_ID	Name	Formula	Molecular Weight	RT [min]	Kegg_ID	HMDB_ID	m/z
Com_77299_pos	4-(5H-thieno[3',2':5,6]thiino[4,3-d]pyrimidin-2-yl)benzamide	C16 H11 N3 O S2	325.03011	7.064	--	--	326.03738
Com_77371_pos	5-benzhydryl-1-(phenylsulfonyl)-1H-pyrazole	C22 H18 N2 O2 S	396.08242	8.324	--	--	397.08994
Com_77406_pos	3,4-dihydro-2H-benzo[4,5]imidazo[2,1-b][1,3]thiazin-3-ol	C10 H10 N2 O S	206.05176	8.424	--	--	207.05904
Com_78060_pos	IRH	C18 H32 N8 O4	446.2429	9.954	--	--	447.25015
Com_78439_pos	2-morpholinophenyl 2-thiophenecarboxylate	C15 H15 N O3 S	289.07851	3.98	--	--	290.08578
Com_78540_pos	2-hydroxy-6-[(8Z,11Z)-pentadeca-8,11,14-trien-1-yl]benzoic acid	C22 H30 O3	359.24581	7.247	--	--	360.25296
Com_79212_pos	INH	C16 H26 N6 O5	404.17344	7.364	--	--	405.18094
Com_79214_pos	VLH	C17 H29 N5 O4	367.22168	7.617	--	--	368.22885
Com_79410_pos	3-(dimethylamino)-1-(5-methyl-3-phenyl-4-isoxazolyl)-2-propen-1-one	C15 H16 N2 O2	278.10124	2.157	--	--	279.10852
Com_79772_pos	di{4-[(2-hydroxyethyl)(methyl)amino]phenyl}methanone	C19 H24 N2 O3	328.17803	9.491	--	--	329.18567
Com_80722_pos	1-(3,4-dihydroxyphenyl)-7-(4-hydroxyphenyl)heptan-3-one	C19 H22 O4	336.13303	9.533	--	--	337.14029
Com_81357_pos	6-methyl-4-(morpholinomethyl)-2H-chromen-2-one	C15 H17 N O3	259.12392	5.712	--	--	260.1312
Com_82651_pos	Avobenzone	C20 H22 O3	332.13691	7.776	--	--	333.14398
Com_82821_pos	Bisphenol M	C24 H26 O2	726.43734	6.63	--	--	364.22583
Com_83146_pos	2-[(butylamino)(imino)methyl]-1-oxohydrazinium-1-olate	C5 H12 N4 O2	182.07928	1.377	--	--	183.0863

Continuation of Supplementary Table 2

Compound_ID	Name	Formula	Molecular Weight	RT [min]	Kegg_ID	HMDB_ID	m/z
Com_83388_pos	N1-[4-(aminosulfonyl)phenyl]-2,2-dimethylpropanamide	C11 H16 N2 O3 S	256.08473	7.125	--	--	257.09201
Com_83448_pos	5-(2,5-dihydroxyhexyl)oxolan-2-one	C10 H18 O4	224.1046	7.371	--	--	225.1116
Com_83574_pos	3-phenyl-5-(1,2,3-thiadiazol-4-yl)-1,2,4-oxadiazole	C10 H6 N4 O S	252.00851	9.031	--	--	253.01532
Com_83707_pos	N-{4-[4-(3-chlorophenyl)piperazino]butyl}-4-methoxybenzamide	C22 H28 Cl N3 O2	401.18444	8.494	--	--	402.19128
Com_84110_pos	U-47700-d6	C17 H19 [2]H6 Cl N2 O	334.15349	10.098	--	--	335.16074
Com_85067_pos	Didodecyl-3,3-thiodipropionate (DLTDP)	C30 H58 O4 S	514.40152	15.989	--	--	515.40839
Com_85071_pos	ethyl 2-(tert-butyl)-3-chloro-5-cyano-6-morpholinoisonicotinate	C17 H22 Cl N3 O3	351.13454	6.071	--	--	352.14182
Com_85150_pos	NPH	C15 H22 N6 O5	366.16115	7.332	--	--	367.16827
Com_85593_pos	1,2-di(3,4-dimethoxyphenyl)diaz-1-ene	C16 H18 N2 O4	302.12612	4.349	--	--	303.13339
Com_85851_pos	1-[3-(3,4-dichlorophenoxy)-2-hydroxypropyl]piperidine-4-carboxamide	C15 H20 Cl2 N2 O3	324.09657	14.211	--	--	325.10367
Com_86095_pos	Taurochenodeoxycholic Acid (sodium salt)	C26 H45 N O6 S	481.27936	9.142	--	--	482.28683
Com_86129_pos	HKK	C18 H33 N7 O4	393.25175	5.569	--	--	394.25906
Com_86606_pos	N'3-(3,4,5-trimethoxybenzylidene)pyridine-3-carbohydrazide	C16 H17 N3 O4	315.12197	6.689	--	--	316.12961
Com_86608_pos	Thromboxane B2-biotin	C35 H60 N4 O7 S	702.40038	14.08	--	--	703.40765
Com_86699_pos	N6-Isopentenyladenosine	C15 H21 N5 O4	335.15666	10.256	--	--	336.16394
Com_87092_pos	(-)-Deguelin	C23 H22 O6	356.17939	9.119	--	--	395.14255

Continuation of Supplementary Table 2

Compound_ID	Name	Formula	Molecular Weight	RT [min]	Kegg_ID	HMDB_ID	m/z
Com_87842_pos	N-[4-(benzyloxy)phenyl]-N'-[2-chloro-6-(4-methoxyphenoxy)benzyl]urea	C28 H25 Cl N2 O4	488.15691	10.703	--	--	489.16418
Com_87969_pos	2-(4-methoxyphenyl)-3-(1H-pyrazol-4-yl)acrylonitrile	C13 H11 N3 O	247.06931	9.676	--	--	248.07652
Com_88116_pos	DY131	C18 H21 N3 O2	311.16396	7.822	--	--	312.17123
Com_88430_pos	1-[(3S)-3-(1,3-Benzoxazol-2-yl)-1-pyrrolidinyl]-3-methoxy-1-propanone	C15 H18 N2 O3	296.11108	8.973	--	--	297.1185
Com_88540_pos	2,6-di(2-thienylmethylidene)cyclohexan-1-one	C16 H14 O S2	286.04527	9.17	--	--	287.05255
Com_88618_pos	L-(-)-alpha-Amino-epsilon-Caprolactam	C6 H12 N2 O	128.09531	2.542	--	--	129.10258
Com_88755_pos	4-methoxy-N-[2-(4-methoxyphenoxy)-5-(trifluoromethyl)phenyl]benzamide	C22 H18 F3 N O4	417.12071	6.288	--	--	418.12753
Com_88842_pos	H-Gly-Pro-OH	C7 H12 N2 O3	172.08525	2.659	--	--	173.09253
Com_89033_pos	ADBICA N-pentanoic acid metabolite	C20 H27 N3 O4	411.15029	7.542	--	--	412.15756
Com_89414_pos	3,3-dimethyl-5-oxo-5-(2-piperidinoanilino)pentanoic acid	C18 H26 N2 O3	318.19448	7.498	--	--	319.20193
Com_89591_pos	3-(5,7-dimethoxy-4-oxo-4H-chromen-2-yl)propanoic acid	C14 H14 O6	278.07694	8.045	--	--	279.08459
Com_89727_pos	Abametapir	C12 H12 N2	184.10036	8.328	--	--	185.10764
Com_90021_pos	2-[(3S)-1-(2-Fluorobenzyl)-3-pyrrolidinyl]-1,3-benzothiazole	C18 H17 F N2 S	312.11067	6.985	--	--	313.11795
Com_90104_pos	4-[1-(dimethylamino)ethylidene]-2-phenyl-1,3-oxazol-5(4H)-one	C13 H14 N2 O2	252.09006	10.583	--	--	253.09734

Continuation of Supplementary Table 2

Compound_ID	Name	Formula	Molecular Weight	RT [min]	Kegg_ID	HMDB_ID	m/z
Com_90641_pos	4-methoxy-6-[2-(4-methoxyphenyl)ethyl]-2H-pyran-2-one	C15 H16 O4	298.06276	7.193	--	--	299.07004
Com_90790_pos	1-(2,4-difluorobenzoyl)-4-piperidinecarboxylic acid	C13 H13 F2 N O3	269.08882	5.631	--	--	270.09573
Com_90858_pos	4-acetyl-4-(ethoxycarbonyl)heptanedioic acid	C12 H18 O7	296.08771	6.605	--	--	297.09494
Com_91307_pos	4-methyl-3-(methylthio)-5-undecyl-4H-1,2,4-triazole	C15 H29 N3 S	321.15822	10.672	--	--	322.1655
Com_91363_pos	1-hydroxy-1-(4-methoxyphenyl)propan-2-yl 4-methoxybenzoate	C18 H20 O5	338.11424	9.508	--	--	339.12152
Com_91546_pos	N-(2-hydroxy-2-phenylethyl)-N'-(2-thienyl)urea	C13 H14 N2 O2 S	262.08247	8.27	--	--	263.08994
Com_92367_pos	FRH	C21 H30 N8 O4	480.22088	6.462	--	--	481.22821
Com_93350_pos	PC (13:0/13:0)	C34 H68 N O8 P	649.46947	16.613	--	--	650.47675
Com_93582_pos	Prostaglandin F2α-1-glyceryl ester	C23 H40 O7	450.25932	7.025	--	--	451.26645
Com_93786_pos	YMK	C20 H32 N4 O5 S	422.1913	6.774	--	--	423.19858
Com_94008_pos	NQH	C15 H23 N7 O6	397.17374	6.553	--	--	398.18094
Com_94771_pos	1,3-dipyridin-3-ylpropane-1,3-dione	C13 H10 N2 O2	226.07475	8.662	--	--	227.08203
Com_94878_pos	4-(4-methoxyphenyl)-4,5-dihydropyrrolo[1,2-a]quinoxaline	C18 H16 N2 O	276.12233	6.992	--	--	277.12961
Com_94989_pos	4-(pentyloxy)benzene-1-carbohydrazide	C12 H18 N2 O2	222.14061	13.864	--	--	223.14789
Com_95212_pos	YLH	C21 H29 N5 O5	215.60865	5.871	--	--	216.61572
Com_96248_pos	KKK	C18 H38 N6 O4	201.14764	6.608	--	--	202.15486
Com_96455_pos	Ip7G	C16 H23 N5 O5	365.17034	1.362	--	--	383.20416
Com_96666_pos	6-anilino-1,3-dimethyl-1,2,3,4-tetrahydropyrimidine-2,4-dione	C12 H13 N3 O2	231.10074	7.506	--	--	232.10806
Com_97098_pos	DNK	C14 H25 N5 O7	375.17061	6.026	--	--	376.17789

Continuation of Supplementary Table 2

Compound_ID	Name	Formula	Molecular Weight	RT [min]	Kegg_ID	HMDB_ID	m/z
Com_97659_pos	methyl 6-[(4-chlorophenyl)thio]-5-nitronicotinate	C13 H9 Cl N2 O4 S	323.99581	2.594	--	--	325.0032
Com_98073_pos	3-(4-ethoxyphenyl)-2-(tetrahydro-1H-pyrrol-1-ylcarbonyl)acrylonitrile	C16 H18 N2 O2	270.13234	5.364	--	--	271.13962
Com_98485_pos	Dl-2-Amino-3-phosphonopropionic acid	C3 H8 N O5 P	169.01417	1.26	--	--	170.02167
Com_98885_pos	KNK	C16 H32 N6 O5	388.24368	5.797	--	--	389.25055
Com_98927_pos	Octadeca-11E,13E,15Z-trienoic acid	C18 H30 O2	278.2242	0.55	--	--	279.23129
Com_99972_pos	N-(1-benzothiophen-2-yl)-N'-(2-methylphenyl)urea	C16 H14 N2 O S	304.06951	7.529	--	--	305.07678
Com_100009_pos	N5-(2-chloro-6-phenoxybenzyl)-1H-1,2,4-triazole-3,5-diamine	C15 H14 Cl N5 O	337.07412	7.676	--	--	338.0813
Com_100472_pos	MAM2201 N-(5-chloropentyl) analog	C25 H24 Cl N O	389.1621	6.216	--	--	390.16937
Com_101781_pos	2-methyl-2,3,4,5-tetrahydro-1,5-benzoxazepin-4-one	C10 H11 N O2	159.06861	8.138	--	--	160.07588
Com_101931_pos	PDMP	C23 H38 N2 O3	390.29308	16.244	--	--	391.30035
Com_102140_pos	WLK	C23 H35 N5 O4	427.25532	6.464	--	--	428.26245
Com_102988_pos	YLK	C21 H34 N4 O5	422.25221	5.578	--	--	423.25919
Com_103012_pos	N-butyl-2-methyl-5-(piperidinosulfonyl)-3-furamide	C15 H24 N2 O4 S	328.1501	8.112	--	--	329.15738
Com_103040_pos	N-(2-morpholinophenyl)-2-furamide	C15 H16 N2 O3	272.11553	6.09	--	--	273.1228
Com_103916_pos	2-[(7-methyl-2,3-dihydro-1H-inden-4-yl)oxy]pyridin-3-amine	C15 H16 N2 O	240.12164	1.123	--	--	241.12881
Com_104017_pos	N alpha-Acetyl-L-Arginine	C8 H16 N4 O3	216.12319	1.39	--	--	471.20953
Com_104646_pos	IMH	C17 H29 N5 O4 S	399.18839	7.987	--	--	400.1954
Com_105203_pos	2-[4-(tert-butyl)-2-chlorophenoxy]-3-nitropyridine	C15 H15 Cl N2 O3	306.07869	6.136	--	--	307.08597

Continuation of Supplementary Table 2

Compound_ID	Name	Formula	Molecular Weight	RT [min]	Kegg_ID	HMDB_ID	m/z
Com_106007_pos	2,4-diaziran-1-yl-6-(1-phenyl-1H-pyrrol-2-yl)-1,3,5-triazine	C17 H16 N6	304.14208	7.709	--	--	305.14935
Com_106480_pos	Amlexanox	C16 H14 N2 O4	298.09569	10.552	--	--	299.10297
Com_106584_pos	ALK	C15 H30 N4 O4	165.11315	2.268	--	--	166.12071
Com_107005_pos	N-[(4-hydroxy-3-methoxyphenyl)methyl]-8-methylnonanamide	C18 H29 N O3	329.19869	16.089	--	--	330.20596
Com_107414_pos	Benzamidine Hydrochloride Hydrate	C7 H11 Cl N2 O	174.05584	7.402	--	--	175.06308
Com_107571_pos	MJN110	C22 H21 Cl2 N3 O4	461.09557	5.196	--	--	462.10284
Com_109489_pos	N4-(4-chloro-2,5-dimethoxyphenyl)morpholine-4-carbothioamide	C13 H17 Cl N2 O3 S	338.04576	6.736	--	--	339.05304
Com_209_pos	MAG (18:3)	C21 H36 O4	352.26079	13.243	--	--	353.26788
Com_269_pos	MAG (18:2)	C21 H38 O4	354.27663	13.22	--	--	355.28445
Com_284_pos	(+/-)12(13)-DiHOME	C18 H34 O4	296.23527	12.276	--	--	297.24252
Com_512_pos	9-HpOTrE	C18 H30 O4	310.21476	13.181	--	--	311.22226
Com_671_pos	LPC 16:0	C24 H50 N O7 P	495.33316	14.761	--	--	496.3407
Com_841_pos	Oleoyl ethanolamide	C20 H39 N O2	325.29822	14.962	--	--	326.30545
Com_1119_pos	LPE 15:0	C20 H42 N O7 P	439.27057	14.261	--	--	440.27786
Com_1527_pos	18-β-Glycyrrhetinic acid	C30 H46 O4	470.34057	13.799	--	--	471.34796
Com_1600_pos	N-Oleoyl Glycine	C20 H37 N O3	339.27744	14.325	--	--	340.28491
Com_1627_pos	LPE 14:1	C19 H38 N O7 P	423.23902	14.025	--	--	424.24753
Com_1940_pos	Asp-Glu	C9 H14 N2 O7	262.07977	1.362	--	--	263.08701
Com_2471_pos	Prostaglandin K2	C20 H30 O5	332.19667	13.607	--	--	333.2041

Continuation of Supplementary Table 2

Compound_ID	Name	Formula	Molecular Weight	RT [min]	Kegg_ID	HMDB_ID	m/z
Com_2613_pos	5α-Dihydrotestosterone	C19 H30 O2	290.22443	15.058	--	--	291.2319
Com_2650_pos	Cyclosporine A	C62 H111 N11 O12	1201.84375	14.356	--	--	1202.85156
Com_2888_pos	15-OxoEDE	C20 H34 O3	322.25104	15.116	--	--	323.2583
Com_2952_pos	LPE 18:1	C23 H46 N O7 P	479.30203	14.816	--	--	480.30969
Com_2953_pos	Ethyl chrysanthemumate	C12 H20 O2	196.14673	13.277	--	--	197.15387
Com_3210_pos	7Z, 10Z, 13Z, 16Z, 19Z-docosapentaenoic acid	C22 H34 O2	330.25505	14.673	--	--	331.26233
Com_3528_pos	4-Pregnen-17alpha,20alpha-Diol-3-One	C21 H32 O3	332.23519	10.529	--	--	333.24252
Com_3577_pos	Delta-Tridecalactone	C13 H24 O2	212.17784	14.77	--	--	213.18486
Com_3875_pos	Ergosta-5,7,9(11),22-Tetraen-3-beta-Ol	C28 H42 O	394.32389	13.738	--	--	395.33124
Com_3964_pos	trans-2-Methyl-2-pentenoic Acid	C6 H10 O2	114.06816	10.853	--	--	115.0756
Com_4039_pos	Spiculisporic Acid	C17 H28 O6	328.18867	11.165	--	--	329.19595
Com_4427_pos	Mag (18:1)	C21 H40 O4	356.29335	14.443	--	--	357.30063
Com_4588_pos	12,13-EODE	C18 H32 O3	296.23567	15.122	--	--	297.24197
Com_4696_pos	Bz-RS-ISer(3-Ph)-Ome	C17 H17 N O4	299.11601	9.387	--	--	300.12332
Com_4788_pos	L-Glutamic acid monosodium salt	C5 H8 N Na O4	169.03523	1.301	--	--	170.04256
Com_5092_pos	(5-L-Glutamyl)-L-Amino Acid	C8 H14 N2 O5	218.0904	1.385	--	--	219.09789
Com_5640_pos	beta-Estradiol 17-Acetate	C20 H26 O3	314.18864	13.09	--	--	315.19586
Com_6679_pos	NSI-189	C22 H30 N4 O	366.24096	10.811	--	--	367.24844
Com_6858_pos	LPE 16:1	C21 H42 N O7 P	413.31454	14.225	--	--	414.32162
Com_6939_pos	Cer-NDS (d18:0/15:0)	C33 H67 N O3	525.51335	15.646	--	--	526.52039
Com_6971_pos	Kynurenic acid O-hexside	C16 H17 N O8	351.09526	9.607	--	--	352.10254
Com_7148_pos	11-dehydro Thromboxane B2	C20 H32 O6	368.22014	12.174	--	--	369.22742

Continuation of Supplementary Table 2

Compound_ID	Name	Formula	Molecular Weight	RT [min]	Kegg_ID	HMDB_ID	m/z
Com_7167_pos	23-Norcholic acid	C23 H38 O5	394.27227	11.649	--	--	412.30612
Com_7209_pos	MAG (18:4)	C21 H34 O4	350.24592	10.935	--	--	351.25311
Com_7491_pos	13,14-dihydro-15-keto-tetranor Prostaglandin E2	C16 H26 O5	280.16806	9.912	--	--	281.17551
Com_7664_pos	7-Hydroxy-4-chromone	C9 H6 O3	162.03206	9.065	--	--	163.03934
Com_7698_pos	tetranor-PGFM	C16 H26 O7	312.15505	10.203	--	--	313.16251
Com_7880_pos	2-oxo-2H-chromene-3-carboxylic acid	C10 H6 O4	190.02681	6.798	--	--	191.03398
Com_7984_pos	LPE 17:0	C22 H46 N O7 P	467.30179	14.822	--	--	468.30966
Com_8030_pos	Royal jelly acid	C10 H18 O3	186.12569	8.73	--	--	187.13319
Com_8108_pos	LPC 15:0	C23 H48 N O7 P	481.32143	14.317	--	--	482.3287
Com_8128_pos	LPC 18:3	C26 H48 N O7 P	517.31761	14.134	--	--	518.32495
Com_8234_pos	3,14-dihydro-15-keto-tetranor Prostaglandin E2	C16 H26 O5	320.16019	12.206	--	--	321.16733
Com_8647_pos	N-acetyl-L-ornithine	C7 H14 N2 O3	174.1006	1.391	--	--	175.10782
Com_8841_pos	LPE 18:0	C23 H48 N O7 P	232.14552	15.166	--	--	233.15234
Com_8918_pos	Lysopc 20:4	C28 H50 N O7 P	543.33109	14.862	--	--	544.33844
Com_9442_pos	Lysopc 18:3	C26 H48 N O7 P	517.31474	14.797	--	--	518.32202
Com_9939_pos	LPE 15:1	C20 H40 N O7 P	437.25449	14.272	--	--	438.26163
Com_10579_pos	(+/-)-Equol	C15 H14 O3	242.09421	9.665	--	--	243.1015
Com_10862_pos	Asp-Phe methyl ester	C14 H18 N2 O5	294.12187	7.878	--	--	295.12921
Com_10919_pos	Deoxycorticosterone 21-glucoside	C27 H40 O8	492.27467	6.579	--	--	493.28183
Com_11796_pos	N-lactoyl-phenylalanine	C12 H15 N O4	237.10002	9.053	--	--	238.10748
Com_12085_pos	L-Leucyl-L-alanine Hydrate	C9 H18 N2 O3	202.13115	5.832	--	--	203.13838
Com_12290_pos	Lysope 16:0	C21 H44 N O7 P	453.28604	14.321	--	--	454.29346
Com_12753_pos	(+/-)-Cannabichromeorcin	C17 H22 O2	258.16184	12.985	--	--	259.16919

Continuation of Supplementary Table 2

Compound_ID	Name	Formula	Molecular Weight	RT [min]	Kegg_ID	HMDB_ID	m/z
Com_13701_pos	Lysopc 16:2 (2N Isomer)	C24 H46 N O7 P	491.30183	14.421	--	--	492.30905
Com_14069_pos	Corey Lactone Diol	C8 H12 O4	172.07384	7.129	--	--	173.08124
Com_14665_pos	Methyl EudesMate	C11 H14 O5	226.08407	9.044	--	--	227.09145
Com_15125_pos	4-fluoro-N-[4-(4-methylpiperazino)phenyl]benzenesulfonamide	C17 H20 F N3 O2 S	349.12766	10.05	--	--	350.13507
Com_15204_pos	L-beta-Imidazolelactic acid	C6 H8 N2 O3	156.05275	1.35	--	--	351.06879
Com_15905_pos	6,8-di(tert-butyl)-4-oxo-4H-chromene-2-carboxylic acid	C18 H22 O4	302.15205	11.839	--	--	303.15958
Com_15922_pos	Lysope 18:1	C23 H46 N O7 P	479.30236	14.222	--	--	480.30981
Com_16183_pos	Trenbolone acetate	C20 H24 O3	312.17204	7.948	--	--	313.17932
Com_16503_pos	Tetrahydroaldosterone	C21 H32 O5	364.22412	14.095	--	--	365.23218
Com_18019_pos	Lysops 22:6	C28 H44 N O9 P	569.27288	13.895	--	--	570.28052
Com_18352_pos	Thromoboxane B1	C20 H36 O6	394.2336	12.239	--	--	395.24066
Com_18543_pos	DL-3,4-Dihydroxyphenyl glycol	C8 H10 O4	170.05828	7.972	--	--	171.06519
Com_19035_pos	3-Nitro-L-Tyrosine	C9 H10 N2 O5	226.05866	1.381	--	--	227.0659
Com_19904_pos	5-[(Benzoyloxy)methyl]-4,5,6-trihydroxy-2-cyclohexen-1-yl benzoate	C21 H20 O7	384.12102	11.773	--	--	385.12823
Com_22097_pos	Homo-Gamma-Linolenic Acid (C20:3)	C20 H34 O2	306.25607	12.426	--	--	307.26373
Com_22235_pos	2-{[(3-eth-1-ynylphenyl)imino]methyl}-4-nitrophenol	C15 H10 N2 O3	266.06901	10.829	--	--	267.07614
Com_23134_pos	(2E)-4-Hydroxy-3,7-dimethyl-2,6-octadien-1-yl beta-D-glucopyranoside	C16 H28 O7	349.21065	6.162	--	--	350.21793
Com_23580_pos	13,14-dihydro-15-keto Prostaglandin A2	C20 H30 O4	356.19642	12.791	--	--	357.20346

Continuation of Supplementary Table 2

Compound_ID	Name	Formula	Molecular Weight	RT [min]	Kegg_ID	HMDB_ID	m/z
Com_24075_pos	1-benzyl-3-(2-methylphenyl)-3,7-dihydro-1H-purine-2,6-dione	C19 H16 N4 O2	332.12631	12.958	--	--	333.13367
Com_24534_pos	13,14-dihydro-15-keto-tetranor Prostaglandin D2	C16 H26 O5	280.16688	9.531	--	--	281.17429
Com_24540_pos	cis-7-Hexadecenoic Acid	C16 H30 O2	254.22445	14.271	--	--	255.23152
Com_26019_pos	lithocholic acid 3-sulfate sodium salt	C24 H38 O6 S Na2	500.21682	11.129	--	--	501.224
Com_26283_pos	D-2-Aminoadipic acid	C6 H11 N O4	161.06893	2.249	--	--	162.0762
Com_27610_pos	N-{5-[(dimethylamino)sulfonyl]-2-methylphenyl}cyclohexanecarboxamide	C16 H24 N2 O3 S	324.15461	10.402	--	--	649.31531
Com_28815_pos	N-(2,5-diethoxy-4-morpholinophenyl)-4-methoxybenzenesulfonamide	C21 H28 N2 O6 S	414.18934	9.585	--	--	415.19644
Com_29357_pos	S-(5-Adenosy)-L-Homocysteine	C14 H20 N6 O5 S	384.12099	10.215	--	--	385.12827
Com_30888_pos	4-Butylresorcinol	C10 H14 O2	166.09964	8.677	--	--	167.10699
Com_31243_pos	Lysope 14:0	C19 H40 N O7 P	425.25443	13.699	--	--	426.26187
Com_31298_pos	S-(Methyl)Glutathione	C11 H19 N3 O6 S	321.10051	10.095	--	--	322.10773
Com_31903_pos	Calcium D-Panthotenate	C18 H32 Ca N2 O10	476.16958	8.77	--	--	499.15875
Com_31934_pos	5,6-dimethyl-3-[5-(trifluoromethyl)pyridin-2-yl]-1,2,4-triazine	C11 H9 F3 N4	254.07661	6.706	--	--	255.08408
Com_33767_pos	2-deoxyglucose-6-phosphate	C6 H13 O8 P	244.03487	8.306	--	--	245.04243
Com_36340_pos	LSD-d3	C20 H22 [2]H3 N3 O	326.21995	15.451	--	--	327.2276

Continuation of Supplementary Table 2

Compound_ID	Name	Formula	Molecular Weight	RT [min]	Kegg_ID	HMDB_ID	m/z
Com_38437_pos	N1-(4-cyclohexylphenyl)-2-[(4-methylphenyl)thio]acetamide	C21 H25 N O S	361.1501	10.551	--	--	362.15738
Com_39750_pos	Sodium Dehydrocholate	C24 H33 Na O5	424.22279	10.484	--	--	425.23041
Com_42336_pos	3-[4-methyl-1-(2-methylpropanoyl)-3-oxocyclohexyl]butanoic acid	C15 H24 O4	290.14985	11.985	--	--	291.15704
Com_44336_pos	Isobutyryl carnitine	C11 H21 N O4	231.14685	2.272	--	--	232.15413
Com_45173_pos	(1E)-1,7-bis(4-hydroxyphenyl)hept-1-en-3-one	C19 H20 O3	296.14111	9.526	--	--	297.1485
Com_46427_pos	Asp-Phe	C13 H16 N2 O5	280.1069	5.855	--	--	281.11453
Com_48690_pos	Pyridoxine O-Glucoside	C14 H21 N O8	331.12743	6.126	--	--	332.1347
Com_50031_pos	JWH 018 N-(5-hydroxypentyl) β-D-Glucuronide	C30 H31 N O8	555.18568	9.593	--	--	556.1936
Com_53167_pos	N1-[2,4-dimethoxy-5-(trifluoromethyl)phenyl]-2,6-dimethoxybenzamide	C18 H18 F3 N O5	385.116	11.235	--	--	386.12332
Com_53383_pos	Phe-Pro	C14 H18 N2 O3	262.13193	10.421	--	--	263.13934
Com_53764_pos	Gly-Tyr-Ala	C14 H19 N3 O5	309.13217	4.614	--	--	310.13934
Com_56122_pos	L-Cysteine-glutathione gisulfide	C13 H22 N4 O8 S2	426.08927	11.72	--	--	427.09677
Com_64332_pos	N1-methyl-5-methoxy-2-({2-[(methylamino)carbonyl]phenyl}thio)benzamide	C17 H18 N2 O3 S	352.07946	9.614	--	--	353.08664
Com_67768_pos	Taurocholic acid sodium salt hydrate	C26 H45 N Na O7 S	538.28368	10.601	--	--	539.29095
Com_85809_pos	4-((5-(4-Nitrophenyl)oxazol-2-yl)amino)benzonitrile	C16 H10 N4 O3	306.07391	7.312	--	--	307.08087
Com_93568_pos	Salvinorin B	C21 H26 O7	390.1676	10.839	--	--	391.17484
Com_97668_pos	N-Acetyl-Asp-Glu	C11 H16 N2 O8	304.09039	1.382	--	--	305.09781

Continuation of Supplementary Table 2

Compound_ID	Name	Formula	Molecular Weight	RT [min]	Kegg_ID	HMDB_ID	m/z
Com_99697_pos	N epsilon,N epsilon,N epsilon-trimethyllysine	C9 H20 N2 O2	188.15233	6.709	--	--	189.15961
Com_403_neg	DL-4-Hydroxyphenyllactic acid	C9 H10 O4	182.05817	9.62	--	--	181.05083
Com_631_neg	L-Threonic acid	C4 H8 O5	136.03757	1.222	--	--	135.03029
Com_1703_neg	(±)9(10)-DiHOME	C18 H34 O4	314.2463	12.7	--	--	313.23895
Com_2624_neg	D-(-)-Quinic acid	C7 H12 O6	192.06388	1.224	--	--	191.05669
Com_3940_neg	D-α-Hydroxyglutaric acid	C5 H8 O5	148.03759	1.181	--	--	147.03033
Com_7552_neg	Uridine monophosphate (UMP)	C9 H13 N2 O9 P	324.0366	1.376	--	--	323.0293
Com_9308_neg	Guanosine monophosphate (GMP)	C10 H14 N5 O8 P	363.05874	1.38	--	--	362.05145
Com_13655_neg	(2R)-2,3-Dihydroxypropanoic acid	C3 H6 O4	106.02701	1.223	--	--	105.0198
Com_16812_neg	Flavin mononucleotide (FMN)	C17 H21 N4 O9 P	456.10592	7.373	--	--	455.09842
Com_17430_neg	N-Acetyl-D-alloisoleucine	C8 H15 N O3	173.10572	6.683	--	--	172.09843
Com_38545_neg	N2-Methylguanosine	C11 H15 N5 O5	297.10795	5.615	--	--	296.10062
Com_38868_neg	2-(acetylamino)-3-(1H-indol-3-yl)propanoic acid	C13 H14 N2 O3	246.10093	7.395	--	--	245.09372
Com_25469_neg	5-Hydroxyindole	C8 H7 N O	133.05314	8.99	--	--	132.04591
Com_134_neg	FAHFA (24:4/6:0)	C30 H50 O4	474.37138	13.608	--	--	473.36395
Com_171_neg	5,7-Dihydroxy-2-(3-hydroxy-4-methoxyphenyl)chroman-4-one	C16 H14 O6	302.07922	10.732	--	--	301.0719
Com_176_neg	(+/-)9(10)-EpOME	C18 H32 O3	296.23545	13.233	--	--	295.22806
Com_289_neg	(+/-)9-HpODE	C18 H32 O4	312.23058	12.506	--	--	311.22324
Com_296_neg	4-Hydroxyisoleucine	C6 H13 N O3	147.08992	1.398	--	--	146.0826
Com_352_neg	N-Isovalerylglycine	C7 H13 N O3	159.08992	1.348	--	--	158.08258
Com_372_neg	trans-10-Heptadecenoic acid	C17 H32 O2	268.24073	14.523	--	--	267.23343
Com_449_neg	11(E)-Eicosenoic Acid	C20 H38 O2	310.28782	15.139	--	--	309.28049

Continuation of Supplementary Table 2

Compound_ID	Name	Formula	Molecular Weight	RT [min]	Kegg_ID	HMDB_ID	m/z
Com_460_neg	11(Z),14(Z)-Eicosadienoic acid	C20 H36 O2	326.28269	14.852	--	--	325.27545
Com_520_neg	FAHFA (18:1/18:2)	C36 H64 O4	560.48017	14.649	--	--	559.4743
Com_535_neg	15(R)-Prostaglandin E2	C20 H32 O5	352.22557	11.2	--	--	351.2182
Com_657_neg	N-(2,6-difluorophenyl)-2-(4-nitrophenyl)acetamide	C14 H10 F2 N2 O3	347.08339	9.35	--	--	346.07611
Com_732_neg	LPS 15:0	C21 H42 N O9 P	483.26069	13.907	--	--	482.25424
Com_879_neg	LPG 15:0	C21 H43 O9 P	470.2654	13.929	--	--	469.258
Com_941_neg	Prostaglandin K1	C20 H32 O5	334.21496	11.699	--	--	333.20761
Com_1052_neg	5-(sec-butyl)-2-hydroxybenzaldehyde N-phenylhydrazone	C17 H20 N2 O	268.15866	13.619	--	--	267.15134
Com_1457_neg	8-Iso-15-keto-prostaglandin-F2β	C20 H32 O5	334.21498	12.034	--	--	333.20767
Com_1459_neg	13,14-Dihydro-15-keto-tetranor prostaglandin F1α	C16 H28 O5	300.19425	9.808	--	--	299.1868
Com_1485_neg	alpha-D-Glucopyranosyl 2-O-(2-methylbutanoyl)-alpha-D-glucopyranoside	C17 H30 O12	472.17722	11.366	--	--	471.17001
Com_1495_neg	FAHFA (4:0/18:0)	C22 H42 O4	370.30892	14.602	--	--	369.30173
Com_1586_neg	LPG 18:1	C24 H47 O9 P	510.29693	14.254	--	--	509.28952
Com_1647_neg	2-(2-pyridyl)-2-{2-[4-(trifluoromethoxy)phenyl]hydrazono}acetonitrile	C14 H9 F3 N4 O	306.07455	6.85	--	--	305.06729
Com_1736_neg	ethyl 2-[5-(4-chlorophenyl)-1,3,4-oxadiazol-2-yl]acetate	C12 H11 Cl N2 O3	266.04655	3.6	--	--	265.03915
Com_1769_neg	(±)8(9)-DiHET	C20 H34 O4	338.24615	13.722	--	--	337.23813
Com_1787_neg	2,3-Dinor prostaglandin E1	C18 H30 O5	308.19937	11.412	--	--	307.19208
Com_1791_neg	LPG 16:0	C22 H45 O9 P	484.28125	14.106	--	--	483.27405

Continuation of Supplementary Table 2

Compound_ID	Name	Formula	Molecular Weight	RT [min]	Kegg_ID	HMDB_ID	m/z
Com_1823_neg	FAHFA (6:0/18:0)	C24 H46 O4	398.34055	14.932	--	--	397.33325
Com_1980_neg	FAHFA (4:0/22:0)	C26 H50 O4	426.37224	15.604	--	--	425.36478
Com_2010_neg	(±)12(13)-DiHOME	C18 H34 O4	314.24628	12.294	--	--	313.23895
Com_2377_neg	FAHFA (2:0/17:0)	C19 H36 O4	328.26211	14.545	--	--	327.25482
Com_2483_neg	2-{1-[2-(4-benzhydrylpiperazino)-2-oxoethyl]cyclopentyl}acetic acid	C26 H32 N2 O3	420.23686	10.01	--	--	419.22943
Com_2499_neg	FAHFA (18:2/20:4)	C38 H62 O4	582.46369	14.442	--	--	581.4566
Com_2500_neg	methyl 2-[(2-methoxyacetyl)amino]thiophene-3-carboxylate	C9 H11 N O4 S	229.04135	6.12	--	--	228.03403
Com_2581_neg	2,2-Bis(hydroxymethyl)propionic acid	C5 H10 O4	134.05834	1.366	--	--	133.0511
Com_2669_neg	Milbemectin A3	C31 H44 O7	528.31287	13.343	--	--	527.30573
Com_2826_neg	N1-(1,3,4-thiadiazol-2-yl)-2-(benzoylamino)benzamide	C16 H12 N4 O2 S	324.06756	8.853	--	--	323.06021
Com_3136_neg	methyl 7-hydroxy-4-oxo-8-propyl-4H-1-benzothiine-2-carboxylate	C14 H14 O4 S	278.06468	5.68	--	--	277.05753
Com_3152_neg	OxPG (18:1-18:1+2O)	C42 H79 O12 P	806.52086	15.218	--	--	805.51318
Com_3215_neg	FAHFA (5:0/22:0)	C27 H52 O4	440.38774	15.797	--	--	439.38034
Com_3240_neg	Oxazepam-d5	C15 H6 [2]H5 Cl N2 O2	291.08315	7.018	--	--	290.07581
Com_3249_neg	Tetranor-PGDM	C16 H24 O7	310.14235	7.758	--	--	309.13504
Com_3291_neg	PEtOH (18:0-18:2)	C41 H77 O8 P	728.54563	14.432	--	--	727.53839
Com_3303_neg	15(R)-Lipoxin A4	C20 H32 O5	334.21505	13.242	--	--	333.20779
Com_3533_neg	FAHFA (18:1/20:3)	C38 H66 O4	586.49489	14.728	--	--	585.48737

Continuation of Supplementary Table 2

Compound_ID	Name	Formula	Molecular Weight	RT [min]	Kegg_ID	HMDB_ID	m/z
Com_3609_neg	15(R),19(R)-Hydroxy prostaglandin F1α	C20 H36 O6	354.24126	11.951	--	--	353.23407
Com_3641_neg	FAHFA (2:0/18:1)	C20 H36 O4	340.26189	12.872	--	--	339.25458
Com_3706_neg	ethyl 3-cyano-2-hydroxy-6-phenylisonicotinate	C15 H12 N2 O3	268.08134	3.023	--	--	267.07407
Com_3712_neg	FAHFA (18:4/10:0)	C28 H46 O4	446.34078	13.137	--	--	445.33322
Com_4002_neg	Methylgallate	C8 H8 O5	184.0376	15.687	--	--	183.03032
Com_4020_neg	19(R)-Hydroxy prostaglandin F1α	C20 H36 O6	372.25164	10.514	--	--	371.24448
Com_4197_neg	FAHFA (4:0/24:0)	C28 H54 O4	454.40356	15.999	--	--	453.39627
Com_4209_neg	LysoPE 18:2	C23 H44 N O7 P	477.28656	13.935	--	--	476.27914
Com_4233_neg	19(R)-hydroxy Prostaglandin A2	C20 H30 O5	332.19941	11.795	--	--	331.19208
Com_4464_neg	6β-Prostaglandin I1	C20 H34 O5	354.24107	13.462	--	--	353.2338
Com_4536_neg	LPG 16:1	C22 H43 O9 P	482.26588	13.787	--	--	481.25861
Com_4541_neg	6-Ketoprostaglandin F1α	C20 H34 O6	352.2258	10.824	--	--	351.21863
Com_4616_neg	FAHFA (3:0/18:0)	C21 H40 O4	356.29283	14.454	--	--	355.28568
Com_4623_neg	FAHFA (4:0/18:1)	C22 H40 O4	368.29333	14.323	--	--	367.28607
Com_4629_neg	(2-amino-5-chlorophenyl)(phenyl)methanone oxime	C13 H11 Cl N2 O	246.05658	6.944	--	--	245.04938
Com_4663_neg	LPG 17:1	C23 H45 O9 P	496.28137	14.233	--	--	495.27393
Com_4782_neg	FAHFA (4:0/24:1)	C28 H52 O4	452.38795	15.66	--	--	451.38065
Com_4997_neg	15-keto Prostaglandin A1	C20 H30 O4	334.21476	13.648	--	--	333.20767
Com_5002_neg	FAHFA (4:0/24:4)	C28 H46 O4	446.34074	14.823	--	--	445.33368
Com_5119_neg	LPG 19:1	C25 H49 O9 P	524.31272	14.516	--	--	523.3053
Com_5122_neg	8-iso-15-keto Prostaglandin E2	C20 H30 O5	332.19923	13.099	--	--	331.19196
Com_5135_neg	FAHFA (5:0/20:0)	C25 H48 O4	412.35643	15.455	--	--	411.34915
Com_5252_neg	LPG 17:0	C23 H47 O9 P	498.29736	14.253	--	--	497.28998

Continuation of Supplementary Table 2

Compound_ID	Name	Formula	Molecular Weight	RT [min]	Kegg_ID	HMDB_ID	m/z
Com_5327_neg	FAHFA (15:0/17:2)	C32 H58 O4	506.43232	14.313	--	--	505.42505
Com_5469_neg	FAHFA (2:0/24:1)	C26 H48 O4	424.35653	15.41	--	--	423.34903
Com_5607_neg	LPI 16:0	C25 H49 O12 P	572.2979	14.168	--	--	571.29016
Com_5616_neg	LysoPC 20:2	C28 H54 N O7 P	547.36226	11.591	--	--	546.35498
Com_5702_neg	FAHFA (5:0/18:0)	C23 H44 O4	384.32472	14.795	--	--	383.31738
Com_5787_neg	N-(2-hydroxyethyl)-N'-propylthiourea	C6 H14 N2 O S	212.08444	8.877	--	--	211.07721
Com_5788_neg	3-(3-nitrophenyl)-2-phenylacrylic acid	C15 H11 N O4	290.0451	1.134	--	--	289.03763
Com_5794_neg	FAHFA (2:0/18:0)	C20 H38 O4	342.27762	14.236	--	--	341.27042
Com_5842_neg	8-iso Prostaglandin F2β	C20 H34 O5	336.23065	10.677	--	--	335.22324
Com_5905_neg	4-(acetylamino)phenyl 3-chlorobenzoate	C15 H12 Cl N O3	289.04875	1.136	--	--	288.04163
Com_6195_neg	FAHFA (16:0/18:2)	C34 H62 O4	534.4634	14.601	--	--	533.45587
Com_6211_neg	4-(hydroxymethyl)benzoic acid	C8 H8 O3	152.04777	10.457	--	--	151.04059
Com_6261_neg	Hostanox O3	C50 H66 O8	794.47104	12.961	--	--	793.46362
Com_6270_neg	11-Deoxy prostaglandin F2β	C20 H34 O4	338.24636	10.947	--	--	337.23901
Com_6283_neg	PA (2:0/12:0)	C17 H33 O8 P	350.19188	13.518	--	--	349.18439
Com_6310_neg	(+/-)19(20)-DiHDPA	C22 H34 O4	362.24403	14.441	--	--	361.23721
Com_6431_neg	1,5,8-Trihydroxy-9-oxo-9H-xanthen-3-yl beta-D-glucopyranoside	C19 H18 O11	422.08372	1.168	--	--	421.07635
Com_6434_neg	LPG 13:0	C19 H39 O9 P	442.23412	13.269	--	--	441.22693
Com_6449_neg	FAHFA (2:0/16:0)	C18 H34 O4	314.24644	14.221	--	--	313.2392
Com_6473_neg	LPG 18:2	C24 H45 O9 P	508.28125	14.2	--	--	507.27368
Com_6572_neg	D-(+)-Fucose	C6 H12 O5	164.06895	1.368	--	--	163.06168
Com_6618_neg	FAHFA (18:1/7:0)	C25 H46 O4	410.34046	15.117	--	--	409.33316

Continuation of Supplementary Table 2

Compound_ID	Name	Formula	Molecular Weight	RT [min]	Kegg_ID	HMDB_ID	m/z
Com_6670_neg	FAHFA (6:0/18:1)	C24 H44 O4	396.32509	14.664	--	--	395.31787
Com_6671_neg	LPS 18:1	C24 H46 N O9 P	523.29222	14.277	--	--	522.28503
Com_6745_neg	FAHFA (2:0/24:4)	C26 H42 O4	436.32014	14.465	--	--	435.31152
Com_6896_neg	LPS 20:1	C26 H50 N O9 P	551.32375	14.232	--	--	550.31653
Com_6900_neg	1a,1b-Dihomo prostaglandin F2α	C22 H38 O5	418.24783	12.738	--	--	417.24054
Com_6920_neg	4,6-dimethyl-2-(methylamino)-5-nitronicotinonitrile	C9 H10 N4 O2	206.07933	1.208	--	--	205.07208
Com_7058_neg	N-Acetyl-1-aspartylglutamic acid	C11 H16 N2 O8	304.09526	8.992	--	--	303.08801
Com_7140_neg	Estriol 17-sulfate	C18 H24 O6 S	368.12663	14.004	--	--	367.11935
Com_7289_neg	FAHFA (4:0/20:0)	C24 H46 O4	398.34054	15.216	--	--	397.33334
Com_7494_neg	FAHFA (2:0/24:2)	C26 H46 O4	422.34064	15.153	--	--	421.33347
Com_7688_neg	3-(methylsulfanyl)-5H-[1,2,4]triazino[5,6-b]indole	C10 H8 N4 S	216.04626	9.46	--	--	215.03899
Com_7887_neg	FAHFA (15:0/16:0)	C31 H60 O4	496.45052	16.383	--	--	495.44312
Com_7912_neg	FAHFA (3:0/24:1)	C27 H50 O4	438.37217	15.537	--	--	437.3649
Com_8342_neg	5-(3-chloro-4-methylanilino)-1-methyl-1H-pyrazol-3-ol	C11 H12 Cl N3 O	237.06691	8.773	--	--	236.05963
Com_8350_neg	13,14-Dihydro-15-keto Prostaglandin E2	C20 H32 O5	352.22567	13.149	--	--	351.21829
Com_8352_neg	LPA 16:0	C19 H39 O7 P	410.24416	14.21	--	--	409.23694
Com_8353_neg	FAHFA (3:0/24:4)	C27 H44 O4	432.32475	14.639	--	--	431.31744
Com_8361_neg	LPG 18:3	C24 H43 O9 P	506.2695	13.675	--	--	505.26337
Com_8372_neg	13,14-Dihydro-19(R)-hydroxy prostaglandin E1	C20 H36 O6	372.25192	9.4	--	--	371.24469
Com_8742_neg	FAHFA (5:0/24:0)	C29 H56 O4	468.41914	16.148	--	--	467.41177
Com_8962_neg	N-(2-morpholinophenyl)-2,1,3-benzoxadiazole-4-sulfonamide	C16 H16 N4 O4 S	360.08854	6.159	--	--	359.08127

Continuation of Supplementary Table 2

Compound_ID	Name	Formula	Molecular Weight	RT [min]	Kegg_ID	HMDB_ID	m/z
Com_9183_neg	5-[4-(tert-butyl)phenoxy]-6-nitroisoindoline-1,3-dione	C18 H16 N2 O5	340.10109	1.093	--	--	339.09375
Com_9255_neg	4-Hydroxy-3- methoxyphenylglycol sulfate	C9 H12 O7 S	218.02527	1.209	--	--	217.0181
Com_9258_neg	FAHFA (17:0/18:0)	C35 H68 O4	552.51302	14.765	--	--	551.50574
Com_9539_neg	13,14-dihydro Prostaglandin F1α	C20 H38 O5	394.24949	14.283	--	--	393.24176
Com_9576_neg	GlcADG (18:1-18:3)	C45 H76 O11	792.54082	14.182	--	--	791.53369
Com_9742_neg	Ethyl-β-D-glucuronide	C8 H14 O7	222.07443	1.34	--	--	221.06723
Com_9780_neg	FAHFA (4:0/22:1)	C26 H48 O4	424.35634	15.269	--	--	423.34882
Com_9789_neg	8-iso Prostaglandin F2α Ethanolamide	C22 H39 N O5	433.2508	10.958	--	--	432.24356
Com_9799_neg	Lactobionic acid	C12 H22 O12	358.11191	1.211	--	--	357.10461
Com_9829_neg	diethyl 2-[(4-methoxy-2-nitroanilino)methylidene]malonate	C15 H18 N2 O7	338.11374	8.311	--	--	337.10641
Com_9895_neg	LPS 18:0	C24 H48 N O9 P	525.30693	14.65	--	--	524.29962
Com_9954_neg	11-deoxy Corticosterone	C21 H30 O3	376.22534	12.539	--	--	375.21805
Com_9994_neg	N1-(3-amino-4-chlorophenyl)-2-[2,4-di(tert-pentyl)phenoxy]acetamide	C24 H33 Cl N2 O2	416.22426	12.078	--	--	415.21722
Com_10008_neg	N1-[5-(3,5-dimethylpiperidino)-4-fluoro-2-nitrophenyl]acetamide	C15 H20 F N3 O3	309.14546	11.319	--	--	308.13809
Com_10150_neg	Uridine 5'-diphospho-D-glucose	C15 H24 N2 O17 P2	566.05253	10.878	--	--	565.04504
Com_10159_neg	GlcADG (18:0-18:2)	C45 H80 O11	796.57323	14.468	--	--	795.56702
Com_10171_neg	LPC 18:0	C26 H54 N O7 P	583.38672	15.33	--	--	582.37921
Com_10190_neg	FAHFA (4:0/16:0)	C20 H38 O4	342.27745	14.479	--	--	341.27039

Continuation of Supplementary Table 2

Compound_ID	Name	Formula	Molecular Weight	RT [min]	Kegg_ID	HMDB_ID	m/z
Com_10231_neg	L(+)-Asparagine monohydrate	C4 H10 N2 O4	150.0642	10.878	--	--	149.05688
Com_10370_neg	PA (12:0/18:1)	C33 H63 O8 P	618.42237	14.706	--	--	617.41486
Com_10409_neg	FAHFA (2:0/22:0)	C24 H46 O4	398.34054	15.347	--	--	397.33331
Com_10550_neg	LPI 18:1	C27 H51 O12 P	598.31331	14.252	--	--	597.30603
Com_10570_neg	FAHFA (14:0/16:2)	C30 H54 O4	478.40095	14.121	--	--	477.39368
Com_10583_neg	2-({1-[(2-nitrophenyl)sulfonyl]-1H-pyrrol-2-yl}methylene)malononitrile	C14 H8 N4 O4 S	328.02759	9.493	--	--	327.02036
Com_10719_neg	MGDG (8:0/18:3)	C35 H60 O10	700.44174	11.977	--	--	699.43457
Com_10754_neg	5-Aminoimidazole-4-carboxamide-1-beta-d-ribofuranosyl 5-monophosphate	C9 H15 N4 O8 P	338.06239	1.296	--	--	337.05551
Com_10824_neg	FAHFA (2:0/22:1)	C24 H44 O4	396.32471	15.137	--	--	395.31757
Com_10833_neg	LPI 18:2	C27 H49 O12 P	596.29781	14.014	--	--	595.29077
Com_10858_neg	FAHFA (3:0/16:0)	C19 H36 O4	328.26195	14.355	--	--	327.25476
Com_10860_neg	D,L-3,4-Dihydroxymandelic acid	C8 H8 O5	184.03747	11.367	--	--	183.03014
Com_11405_neg	FAHFA (5:0/24:1)	C29 H54 O4	466.40354	15.805	--	--	465.39642
Com_11433_neg	N'-[4-(trifluoromethyl)benzoyl]-6-quinoxalinecarbohydrazide	C17 H11 F3 N4 O2	360.0883	7.343	--	--	359.08084
Com_11540_neg	FAHFA (4:0/26:2)	C30 H54 O4	478.40333	14.535	--	--	477.39615
Com_11664_neg	P-Toluenesulfonic acid	C7 H8 O3 S	172.01989	6.566	--	--	171.01251
Com_11733_neg	[4-(1H-indol-4-yl)piperazino](2-thienyl)methanone	C17 H17 N3 O S	311.10675	9.741	--	--	310.0997
Com_11799_neg	Dactylorhin E	C27 H40 O16	620.23873	11.108	--	--	619.23145
Com_12717_neg	pyridine-2-carbaldehyde 2-(2-quinolyl)hydrazone	C15 H12 N4	248.10518	13.109	--	--	247.09793
Com_12741_neg	6-(4-methoxyphenyl)pyrimidine-2,4-diamine	C11 H12 N4 O	216.10014	2.447	--	--	215.09283

Continuation of Supplementary Table 2

Compound_ID	Name	Formula	Molecular Weight	RT [min]	Kegg_ID	HMDB_ID	m/z
Com_12801_neg	FAHFA (18:0/20:2)	C38 H70 O4	590.52661	15.038	--	--	589.51959
Com_12960_neg	GlcADG (16:1-16:3)	C41 H68 O11	736.47874	13.647	--	--	735.47137
Com_13047_neg	17(S)-HpDHA	C22 H32 O4	360.23078	13.38	--	--	359.22357
Com_13128_neg	N1-piperidinocarbonyl-4-methylbenzene-1-sulfonamide	C13 H18 N2 O3 S	282.101	9.826	--	--	281.09372
Com_13217_neg	FAHFA (18:2/18:2)	C36 H62 O4	558.464	14.556	--	--	557.4566
Com_13246_neg	5-nitro-2-{[5-(trifluoromethyl)-4H-1,2,4-triazol-3-yl]thio}pyridine	C8 H4 F3 N5 O2 S	292.00188	1.012	--	--	290.9946
Com_13770_neg	5-hex-1-ynyl-2-furoic acid	C11 H12 O3	192.07917	7.765	--	--	191.07195
Com_13855_neg	2-[2-(2,3,4-trifluorophenyl)hydrazono]malononitrile	C9 H3 F3 N4	224.03263	8.407	--	--	223.0253
Com_14201_neg	Dl-3,4-Dihydroxymandelic Acid	C8 H8 O5	184.03759	1.094	--	--	183.03041
Com_14213_neg	Hematoxylin	C16 H14 O6	302.07956	12.486	--	--	301.07233
Com_14272_neg	SM (d14:3/12:1)	C31 H57 N2 O6 P	630.39447	14.073	--	--	629.38672
Com_14275_neg	LPA 19:0	C22 H45 O7 P	452.29688	14.06	--	--	451.28778
Com_14294_neg	4,5-diphenyl-2,3-dihydro-1H-pyrazolo[3,4-c]pyridazin-3-one	C17 H12 N4 O	288.10019	11.851	--	--	287.09302
Com_14318_neg	Nitrazepam-d5	C15 H6 [2]H5 N3 O3	286.10714	8.58	--	--	285.09991
Com_14322_neg	N1-(4-fluorophenyl)-2-[(4-methylphenyl)thio]acetamide	C15 H14 F N O S	275.0799	7.013	--	--	274.0726
Com_14610_neg	LPS 20:2	C26 H48 N O9 P	549.30823	13.969	--	--	548.30103
Com_14799_neg	Δ17-6-keto prostaglandin F1α	C20 H32 O6	368.22024	10.761	--	--	367.21292
Com_14822_neg	PA (2:0/3:0)	C8 H15 O8 P	270.05099	1.422	--	--	306.99973

Continuation of Supplementary Table 2

Compound_ID	Name	Formula	Molecular Weight	RT [min]	Kegg_ID	HMDB_ID	m/z
Com_14933_neg	PE (15:0/16:0)	C36 H72 N O8 P	677.50178	16.234	--	--	676.4939
Com_14967_neg	PG (15:0/15:0)	C36 H71 O10 P	694.48113	16.102	--	--	693.47369
Com_15028_neg	LPE 18:3	C23 H42 N O7 P	475.27103	14.255	--	--	474.26389
Com_15177_neg	2-Amino-3-(4-hydroxy-3-methoxyphenyl)propanoic acid	C10 H13 N O4	211.08482	1.86	--	--	210.07755
Com_15357_neg	2-cyano-3-(4-hydroxyphenyl)acrylamide	C10 H8 N2 O2	188.05884	10.094	--	--	187.05167
Com_15723_neg	LPS 18:2	C24 H44 N O9 P	521.27591	14.013	--	--	520.2688
Com_15739_neg	GlcADG (18:3-18:3)	C45 H72 O11	788.51068	15.636	--	--	787.50342
Com_15759_neg	Milbemectin A4	C32 H46 O7	542.32893	13.035	--	--	541.32166
Com_15996_neg	15-Epiprostaglandin E1	C20 H34 O5	708.49614	15.992	--	--	707.48895
Com_16004_neg	1a,1b-Dihomo prostaglandin E1	C22 H38 O5	364.26198	13.987	--	--	363.25458
Com_16038_neg	N1-[1-(3-isopropenylphenyl)-1-methylethyl]-3-oxobutanamide	C16 H21 N O2	259.15348	2.89	--	--	258.14612
Com_16446_neg	N-benzyl-3-(4-chlorophenyl)-4,5-dihydro-5-isoxazolecarboxamide	C17 H15 Cl N2 O2	314.08317	7.32	--	--	313.07587
Com_16546_neg	Uridine 5'-diphosphate (UDP)	C9 H14 N2 O12 P2	404.00286	1.068	--	--	402.99536
Com_16807_neg	FAHFA (7:0/18:0)	C25 H48 O4	412.35621	15.114	--	--	411.34894
Com_16860_neg	15-Deoxy-δ12,14 -Prostaglandin J2	C20 H28 O3	338.1917	13.821	--	--	337.18423
Com_16888_neg	trans-Petroselinic acid	C18 H34 O2	328.26188	12.903	--	--	327.25452
Com_17252_neg	HexCer-NDS (d15:0/12:0)	C33 H65 N O8	663.48398	13.492	--	--	662.47638
Com_17292_neg	13,14-dihydro-15-keto Prostaglandin D1	C20 H34 O5	390.21482	11.373	--	--	389.20743
Com_17326_neg	Thymidine 3,5-cyclic monophosphate	C10 H13 N2 O7 P	304.04632	7.983	--	--	303.03903

Continuation of Supplementary Table 2

Compound_ID	Name	Formula	Molecular Weight	RT [min]	Kegg_ID	HMDB_ID	m/z
Com_17344_neg	LPE 16:2	C21 H40 N O7 P	449.25522	14.231	--	--	448.24783
Com_17359_neg	Y-Aminobutyric acid (GABA)	C4 H9 N O2	103.06362	1.204	--	--	102.0563
Com_17395_neg	N-Phenyl-5-(4-(trifluoromethyl)phenyl)oxazol-2-amine	C16 H11 F3 N2 O	304.0802	1.168	--	--	303.07297
Com_17815_neg	PG (18:1/16:2)	C40 H73 O10 P	744.49888	14.642	--	--	743.49164
Com_18114_neg	6-methylimidazo[2,1-b][1,3]thiazole-5-carbohydrazide	C7 H8 N4 O S	196.04089	5.598	--	--	195.03362
Com_18799_neg	methyl 5-{[2-(ethoxycarbonyl)-3-oxohex-1-enyl]amino}-2-furoate	C15 H19 N O6	309.12194	5.219	--	--	308.11475
Com_19091_neg	2,5-di[4-(trifluoromethyl)benzylidene]cyclopentan-1-one	C21 H14 F6 O	396.09206	9.694	--	--	395.08487
Com_19580_neg	PG (4:0/15:0)	C25 H49 O10 P	540.30786	14.196	--	--	539.30011
Com_19868_neg	Benzyl 6-O-beta-D-glucopyranosyl-beta-D-glucopyranoside	C19 H28 O11	432.16506	11.661	--	--	431.15765
Com_19927_neg	N'-(cyclohexylcarbonyl)-4-methyl-1,2,3-thiadiazole-5-carbohydrazide	C11 H16 N4 O2 S	268.09852	2.58	--	--	267.09125
Com_20067_neg	4-Hexylresorcinol	C12 H18 O2	194.13087	7.997	--	--	193.1236
Com_20261_neg	13,14-Dihydro-15-keto prostaglandin F2α	C20 H34 O5	780.43197	11.378	--	--	389.20871
Com_20280_neg	15(R),19(R)-Hydroxy prostaglandin F2α	C20 H34 O6	370.23615	9.855	--	--	369.22876
Com_20331_neg	DL-α-Methoxyphenylacetic acid	C9 H10 O3	166.06346	6.448	--	--	165.05614
Com_20367_neg	PG (16:0/15:1)	C37 H71 O10 P	706.48076	16.147	--	--	705.47388
Com_20442_neg	2-(benzoylamino)-4,5-dimethoxybenzoic acid	C16 H15 N O5	301.09553	11.969	--	--	300.08817
Com_20489_neg	PEtOH (19:0-20:4)	C44 H79 O8 P	766.56099	14.635	--	--	765.55371

Continuation of Supplementary Table 2

Compound_ID	Name	Formula	Molecular Weight	RT [min]	Kegg_ID	HMDB_ID	m/z
Com_20837_neg	2-Hydroxy-1-(4-methoxyphenyl)propyl hexopyranoside	C16 H24 O8	390.15111	14.209	--	--	389.14383
Com_20897_neg	Carboxybenzaldehyde	C8 H6 O3	150.03207	8.997	--	--	149.02486
Com_21530_neg	LPC 18:1	C26 H52 N O7 P	567.355	14.9	--	--	566.34802
Com_21549_neg	4-(4-cyclohexylphenyl)-4-oxobut-2-enoic acid	C16 H18 O3	258.12594	11.374	--	--	257.11862
Com_21664_neg	19(R)-Hydroxy prostaglandin F2α	C20 H34 O6	306.22009	12.343	--	--	305.21268
Com_21788_neg	nor-6β-Oxycodol	C17 H21 N O4	303.14362	1.39	--	--	302.13614
Com_22076_neg	Lysopc 14:0	C22 H46 N O7 P	467.30206	13.518	--	--	466.29517
Com_22385_neg	FAHFA (17:0/20:3)	C37 H66 O4	574.49489	14.761	--	--	573.48761
Com_22486_neg	2-[(3-methylbenzo[b]thiophen-2-yl)carbonyl]benzoic acid	C17 H12 O3 S	296.05403	7.609	--	--	295.04675
Com_22501_neg	N3-(4-chlorophenyl)-4-(trifluoromethyl)nicotinamide	C13 H8 Cl F3 N2 O	300.03058	6.65	--	--	299.02325
Com_22557_neg	2-chloro-6-[(2-oxoazepan-3-yl)amino]benzonitrile	C13 H14 Cl N3 O	263.07981	8.548	--	--	262.07245
Com_22672_neg	11-keto Testosterone (CRM)	C19 H26 O3	366.20806	14.072	--	--	365.20166
Com_22917_neg	Cer-NDS (d19:0/15:0)	C34 H69 N O3	599.55055	16.178	--	--	598.54327
Com_22980_neg	LPG 5:0	C11 H23 O9 P	330.10933	7.861	--	--	329.10205
Com_23296_neg	N1-(3-cyano-4,6-diphenyl-2-pyridyl)-4-methylbenzamide	C26 H19 N3 O	389.15148	6.494	--	--	388.1442
Com_23304_neg	PG (4:0/18:2)	C28 H51 O10 P	578.3238	14.438	--	--	577.31653
Com_23319_neg	1-methyl-N-(4-piperidinophenyl)-1H-imidazole-4-sulfonamide	C15 H20 N4 O2 S	320.13775	7.994	--	--	319.13062
Com_23402_neg	LPG 12:0	C18 H37 O9 P	428.21857	13.155	--	--	427.21091

Continuation of Supplementary Table 2

Compound_ID	Name	Formula	Molecular Weight	RT [min]	Kegg_ID	HMDB_ID	m/z
Com_23451_neg	(±)10(11)-EpDPA	C22 H32 O3	344.23911	13.425	--	--	343.23163
Com_23479_neg	Pyridoxal 5-phosphate monohydrate	C8 H12 N O7 P	265.03577	1.71	--	--	264.02853
Com_23590_neg	PE (18:2/18:2)	C41 H74 N O8 P	739.51798	15.808	--	--	738.51117
Com_23711_neg	15(R),19(R)-hydroxy Prostaglandin E1	C20 H34 O6	370.23632	9.143	--	--	369.22879
Com_23731_neg	PA (11:0/16:1)	C30 H57 O8 P	576.36685	14.274	--	--	575.35852
Com_23734_neg	Guanethidine Monosulfate	C10 H24 N4 O4 S	296.15308	10.659	--	--	591.29889
Com_23793_neg	FAHFA (3:0/24:2)	C27 H48 O4	436.35635	15.24	--	--	435.34927
Com_23798_neg	FAHFA (14:0/16:0)	C30 H58 O4	482.43478	16.198	--	--	481.42731
Com_24235_neg	1,4-Cyclohexanedicarboxylic acid	C8 H12 O4	126.06848	2.471	--	--	125.0611
Com_24278_neg	N1-cyclohexyl-4-(4-fluorobenzyl)-1,4-diazepane-1-carbothioamide	C19 H28 F N3 S	349.20084	9.787	--	--	348.1936
Com_24568_neg	PG (13:0/16:0)	C35 H69 O10 P	680.46562	15.919	--	--	679.45856
Com_24663_neg	4-amino-2-(4-chlorophenyl)-6-(methylthio)pyrimidine-5-carbonitrile	C12 H9 Cl N4 S	276.01933	11.094	--	--	275.01205
Com_24799_neg	1-(4-methylphenyl)-3-(2-pyridylthio)pyrrolidine-2,5-dione	C16 H14 N2 O2 S	299.08343	9.431	--	--	298.07617
Com_24958_neg	FAHFA (2:0/21:1)	C23 H42 O4	382.30916	14.519	--	--	381.3017
Com_25000_neg	FAHFA (16:0/16:2)	C32 H58 O4	506.43536	14.143	--	--	505.42798
Com_25437_neg	5-amino-1-(4-chlorobenzyl)-1H-1,2,3-triazole-4-carboxamide	C10 H10 Cl N5 O	252.06387	8.613	--	--	251.05652
Com_25539_neg	DL-β-Leucine	C6 H13 N O2	131.09494	7.054	--	--	130.08766
Com_25648_neg	DGDG (18:3/18:3)	C51 H84 O15	996.60515	16.465	--	--	995.59845
Com_25684_neg	Guanosine-3',5'-cyclic monophosphate	C10 H12 N5 O7 P	345.04789	2.215	--	--	344.04071

Continuation of Supplementary Table 2

Compound_ID	Name	Formula	Molecular Weight	RT [min]	Kegg_ID	HMDB_ID	m/z
Com_26327_neg	ethyl 3-cyano-6-methyl-2-(phenylthio)isonicotinate	C16 H14 N2 O2 S	298.07437	7.238	--	--	297.06705
Com_26342_neg	PG (15:0/15:1)	C36 H69 O10 P	692.46505	15.946	--	--	691.45789
Com_26367_neg	LPC 18:2	C26 H50 N O7 P	565.33856	14.584	--	--	564.33148
Com_26682_neg	(+/-)-CP 47,497-C7-Hydroxy metabolite	C21 H34 O3	334.25144	13.548	--	--	333.24414
Com_26774_neg	4-phenylbenzo[4,5]imidazo[1,2-a]pyrimidin-2-amine	C16 H12 N4	260.10868	6.007	--	--	259.10144
Com_26840_neg	FAHFA (18:3/16:2)	C34 H56 O4	528.41729	14.143	--	--	527.4104
Com_27115_neg	5-(4-morpholinoanilino)-5-oxo-3-phenylpentanoic acid	C21 H24 N2 O4	368.18172	6.237	--	--	367.17444
Com_27477_neg	ethyl 1-cyclohexyl-4,5-dioxopyrrolidine-3-carboxylate	C13 H19 N O4	253.13197	8.304	--	--	252.12469
Com_27515_neg	1-[4-mercapto-2-(2-methoxyphenyl)-6-methylpyrimidin-5-yl]ethan-1-one	C14 H14 N2 O2 S	274.0734	8.129	--	--	273.06616
Com_27708_neg	GlcADG (18:2-18:3)	C45 H74 O11	790.52677	15.88	--	--	789.51923
Com_27873_neg	N'-(4-chlorobenzoyl)-3-[4-(trifluoromethoxy)phenyl]acrylohydrazide	C17 H12 Cl F3 N2 O3	384.05229	7.71	--	--	383.04495
Com_28054_neg	FAHFA (6:0/24:1)	C30 H56 O4	480.41923	16.042	--	--	479.41183
Com_28174_neg	2-[(3S)-1-(Cyclohexylmethyl)-3-pyrrolidinyl]-5-fluoro-1H-benzimidazole	C18 H24 F N3	301.20092	7.591	--	--	300.19366
Com_28578_neg	FAHFA (8:0/18:0)	C26 H50 O4	426.37227	15.28	--	--	425.36499
Com_28606_neg	N-[4-cyano-1-(4-fluorophenyl)-1H-pyrazol-5-yl]cyclohexanecarboxamide	C17 H17 F N4 O	312.13689	10.367	--	--	311.12964
Com_28642_neg	MGDG (16:3/18:3)	C43 H70 O10	806.52114	16.381	--	--	805.51392

Continuation of Supplementary Table 2

Compound_ID	Name	Formula	Molecular Weight	RT [min]	Kegg_ID	HMDB_ID	m/z
Com_28686_neg	N1-(4-chlorophenyl)-3-(1H-pyrrol-1-ylmethyl)piperidine-1-carboxamide	C17 H20 Cl N3 O	634.24905	10.18	--	--	316.11725
Com_28943_neg	PA (11:0/18:3)	C32 H57 O8 P	600.36939	14.169	--	--	599.36279
Com_29555_neg	(±)9-HpODE	C18 H32 O4	294.22023	12.616	--	--	293.21295
Com_29672_neg	N,5-Bis(3-nitrophenyl)oxazol-2-amine	C15 H10 N4 O5	326.06773	5.724	--	--	325.06046
Com_30085_neg	PC (2:0/24:0)	C34 H68 N O8 P	695.46292	14.861	--	--	694.45587
Com_30252_neg	Guanosine 5'-diphosphate (GDP)	C10 H15 N5 O11 P2	443.02513	1.13	--	--	442.01785
Com_30283_neg	PA (11:0/18:1)	C32 H61 O8 P	604.39763	14.724	--	--	603.39075
Com_30455_neg	PE (18:2/18:3)	C41 H72 N O8 P	737.50204	15.581	--	--	736.49493
Com_30720_neg	Glycochenodeoxycholic acid sodium salt	C26 H43 N O5 Na	472.30183	10.936	--	--	471.29456
Com_30838_neg	3-(2-naphthyl)-5-(trifluoromethyl)-1H-pyrazole	C14 H9 F3 N2	262.0692	7.376	--	--	261.06198
Com_30954_neg	FAHFA (2:0/22:4)	C24 H38 O4	390.27724	14.826	--	--	389.26996
Com_30985_neg	Cannabichromevarin	C19 H26 O2	286.19565	10.897	--	--	285.18826
Com_31103_neg	N-{6-[4-(tert-butyl)phenoxy]-3-pyridinyl}-4-(trifluoromethyl)benzamide	C23 H21 F3 N2 O2	414.16212	12.249	--	--	413.15485
Com_31478_neg	1-[6-(benzyloxy)-3-(tert-butyl)-2-hydroxyphenyl]ethan-1-one	C19 H22 O3	298.15747	12.917	--	--	297.15021
Com_31651_neg	PA (12:0/18:2)	C33 H61 O8 P	634.42392	14.431	--	--	633.41577
Com_32174_neg	Cetirizine N-oxide	C21 H25 Cl N2 O4	404.14828	9.071	--	--	403.14078
Com_32256_neg	1-methyl-N-(3-methyl-5-cinnolinyl)-1H-imidazole-4-sulfonamide	C13 H13 N5 O2 S	303.07835	7.331	--	--	302.07108
Com_32491_neg	MGDG (18:3/16:4)	C43 H68 O10	804.50502	16.241	--	--	803.49774

Continuation of Supplementary Table 2

Compound_ID	Name	Formula	Molecular Weight	RT [min]	Kegg_ID	HMDB_ID	m/z
Com_33033_neg	2-(2-chlorophenyl)-1H-indole	C14 H10 Cl N	227.04692	1.23	--	--	226.03961
Com_33077_neg	5-heptyl-4-hydroxy-6H-pyrido[3,2,1-jk]carbazol-6-one	C22 H23 N O2	333.17337	7.881	--	--	332.1658
Com_33419_neg	Chelidamic acid hydrate	C7 H5 N O5	183.01733	1.245	--	--	182.00984
Com_34135_neg	AKB48 N-pentanoic acid metabolite	C23 H29 N3 O3	395.22206	12.534	--	--	394.21478
Com_34150_neg	4-{[2-(4-fluorophenyl)-2-oxoethyl]amino}-4-oxobutanoic acid	C12 H12 F N O4	253.07423	12.083	--	--	252.06696
Com_34192_neg	GlcADG (12:0-15:1)	C36 H64 O11	672.44646	13.51	--	--	671.4389
Com_35035_neg	N-[1-methyl-1-(2-phenyldiaz-1-enyl)ethyl]-N'-phenylthiourea	C16 H18 N4 S	298.1244	12.671	--	--	297.11713
Com_35054_neg	Pyridoxal 5'-phosphate hydrate	C8 H10 N O6 P	247.02478	7.508	--	--	246.0175
Com_35152_neg	2-(1,3-dimethyl-1H-pyrazol-5-yl)-1H-isoindole-1,3(2H)-dione	C13 H11 N3 O2	482.16531	9.868	--	--	240.07538
Com_35196_neg	SM (d14:3/15:1)	C34 H63 N2 O6 P	672.44575	15.087	--	--	671.43829
Com_35325_neg	LPE 20:2	C25 H48 N O7 P	505.31848	15.026	--	--	504.31088
Com_35358_neg	PG (13:0/15:0)	C34 H67 O10 P	666.44991	15.707	--	--	665.44244
Com_35842_neg	OxPG (16:0-18:0+2O)	C40 H79 O12 P	782.519	13.435	--	--	781.51172
Com_35844_neg	PEtOH (16:0-16:0)	C37 H73 O8 P	676.49496	14.75	--	--	675.48737
Com_35883_neg	N'-(4-chlorophenyl)-4-ethylbenzohydrazide	C15 H15 Cl N2 O	274.08463	6.486	--	--	273.07739
Com_35901_neg	O1-[4-(tert-butyl)benzoyl]-2-(tert-butylsulfonyl)ethanehydroximamide	C17 H26 N2 O4 S	390.13228	7.051	--	--	389.12503
Com_36093_neg	N-(2,6-Dimethylphenyl)-N-(methoxyacetyl) alanine	C14 H19 N O4	265.1318	6.826	--	--	264.12457
Com_36134_neg	FAHFA (18:2/18:1)	C36 H64 O4	560.48146	16.52	--	--	559.47418

Continuation of Supplementary Table 2

Compound_ID	Name	Formula	Molecular Weight	RT [min]	Kegg_ID	HMDB_ID	m/z
Com_36987_neg	4-(2-((4-Cyanophenyl)amino)oxazol-5-yl)benzonitrile	C17 H10 N4 O	286.08803	8.179	--	--	285.08075
Com_37390_neg	2-[(3-methyl-1-phenyl-1H-pyrazol-5-yl)amino]benzoic acid	C17 H15 N3 O2	586.22818	10.516	--	--	292.10681
Com_37452_neg	Meclocycline	C22 H21 Cl N2 O8	476.10013	7.648	--	--	475.09311
Com_37584_neg	N'-hydroxy-2-[4-(3-nitrophenyl)-1,3-thiazol-2-yl]ethanimidamide	C11 H10 N4 O3 S	278.0464	6.257	--	--	277.03912
Com_38041_neg	3-(5-acetyl-2-chlorophenyl)-2-thioxo-2,3-dihydro-4(1H)-quinazolinone	C16 H11 Cl N2 O2 S	330.02122	2.681	--	--	329.01395
Com_38170_neg	(3R)-4,4-Dimethyl-2-oxotetrahydro-3-furanyl beta-D-glucopyranoside	C12 H20 O8	338.12263	9.626	--	--	337.11536
Com_38835_neg	Prostaglandin A1 ethyl ester	C22 H36 O4	364.26185	12.19	--	--	363.25458
Com_39287_neg	2-Hydroxy-2-methyl-3-buten-1-yl beta-D-glucopyranoside	C11 H20 O7	310.12639	11.608	--	--	309.11911
Com_39494_neg	2-(5-mercapto-4-methyl-4H-1,2,4-triazol-3-yl)acetonitrile	C5 H6 N4 S	154.03039	5.927	--	--	153.02312
Com_39584_neg	PA (11:0/17:0)	C31 H61 O8 P	592.39918	14.824	--	--	591.39124
Com_39958_neg	SM (d14:3/16:2)	C35 H63 N2 O6 P	684.44647	12.309	--	--	683.43842
Com_40134_neg	2-(3,5-difluorophenyl)-3-(3-thienyl)acrylic acid	C13 H8 F2 O2 S	266.02524	11.461	--	--	265.01791
Com_40321_neg	GlcADG (18:2-18:2)	C45 H76 O11	792.54146	16.175	--	--	791.53418
Com_40493_neg	3-(1-cyano-1,2-dihydroisoquinolin-2-yl)-3-oxopropyl propionate	C16 H16 N2 O3	284.11177	7.077	--	--	283.10437
Com_40665_neg	THJ2201 N-pentanoic acid metabolite	C23 H20 N2 O3	372.14564	7.066	--	--	371.13855

Continuation of Supplementary Table 2

Compound_ID	Name	Formula	Molecular Weight	RT [min]	Kegg_ID	HMDB_ID	m/z
Com_40715_neg	N1-phenyl-2-(2-phenylacetyl)hydrazine-1-carbothioamide	C15 H15 N3 O S	570.18112	8.966	--	--	284.08328
Com_40874_neg	16-(Hexopyranosyloxy)-7-hydroxy-8,9-epoxypimaran-18-oic acid	C26 H42 O10	514.27907	7.498	--	--	513.27179
Com_41832_neg	diethyl 1-(2,3,4,5,6-pentamethylphenyl)hydrazine-1,2-dicarboxylate	C17 H26 N2 O4	322.18998	8.873	--	--	321.18268
Com_42020_neg	Cer-EOS (d14:1/16:2-O-18:1)	C48 H87 N O5	803.65108	14.82	--	--	802.6438
Com_42357_neg	ethyl 3-[3,5-di(trifluoromethyl)anilino]-2-nitroacrylate	C13 H10 F6 N2 O4	372.05195	6.224	--	--	371.04468
Com_42446_neg	N-(4-chlorophenyl)-N'-(2-phenoxyphenyl)urea	C19 H15 Cl N2 O2	384.08816	6.976	--	--	383.08087
Com_42525_neg	(+/-)8(9)-DiHETE	C20 H32 O4	672.46211	13.169	--	--	671.45483
Com_42960_neg	2-(2-carboxy-2-methylpropyl)-4,6-dimethylbenzoic acid	C14 H18 O4	250.12093	9.483	--	--	249.11369
Com_43080_neg	FAHFA (18:0/3:0)	C21 H40 O4	356.29361	14.889	--	--	355.28619
Com_43095_neg	ethyl 2-cyano-3-(tetrahydro-3-thiophenylamino)acrylate	C10 H14 N2 O2 S	452.15475	9.802	--	--	225.0701
Com_43160_neg	2-[6-(1H-benzo[d]imidazol-2-yl)-2-pyridyl]-1H-benzo[d]imidazole	C19 H13 N5	312.12172	7.361	--	--	311.11444
Com_43833_neg	N-(2,5-dichlorobenzyl)-5-ethyl-1H-indole-2-carboxamide	C18 H16 Cl2 N2 O	346.06941	8.634	--	--	345.06213
Com_44075_neg	N1-[2-oxo-6-(1H-pyrrol-1-yl)-2H-chromen-3-yl]acetamide	C15 H12 N2 O3	268.0804	3.951	--	--	267.07312

Continuation of Supplementary Table 2

Compound_ID	Name	Formula	Molecular Weight	RT [min]	Kegg_ID	HMDB_ID	m/z
Com_44132_neg	4-(beta-D-Glucopyranosyloxy)-2-methylenebutanoic acid	C11 H18 O8	556.20218	9.599	--	--	277.09381
Com_44461_neg	LPA 16:1	C19 H37 O7 P	408.22898	13.975	--	--	407.22165
Com_44660_neg	PEtOH (19:2-20:5)	C44 H73 O8 P	760.49647	14.552	--	--	759.4892
Com_45466_neg	4-[(3,4-dimethoxyphenethyl)amino]-4-oxobutanoic acid	C14 H19 N O5	281.12678	6.175	--	--	280.11951
Com_45506_neg	3,4,5-trihydroxy-6-methyloxan-2-yl 2-(methylamino)benzoate	C14 H19 N O6	594.25083	10.081	--	--	593.24316
Com_45705_neg	N-(4-bromo-1-methyl-1H-pyrazol-5-yl)-2-morpholinoacetamide	C10 H15 Br N4 O2	302.04335	8.253	--	--	301.03613
Com_45712_neg	2,6-dimethoxy-N-(1-methyl-1H-pyrazol-5-yl)benzamide	C13 H15 N3 O3	261.11181	6.523	--	--	260.10455
Com_45724_neg	tert-butyl 4-{2-[(4-chlorobenzoyl)amino]ethyl}piperazine-1-carboxylate	C18 H26 Cl N3 O3	367.16339	5.935	--	--	366.15622
Com_46206_neg	N-[2-(2-furylsulfanyl)ethyl]-4,6-dimethyl-1H-indole-2-carboxamide	C18 H20 N2 O2 S	328.11997	5.46	--	--	327.11273
Com_47180_neg	FAHFA (6:0/24:2)	C30 H54 O4	478.40376	15.666	--	--	477.39648
Com_47185_neg	3-[2-(4-isobutylphenyl)-2-oxoethyl]-1,3-dihydroisobenzofuran-1-one	C20 H20 O3	308.13748	7.206	--	--	307.13031
Com_47446_neg	N",N'"-di[1-(4-nitrophenyl)ethylidene]carbonic dihydrazide	C17 H16 N6 O5	420.09251	6.758	--	--	419.08524

Continuation of Supplementary Table 2

Compound_ID	Name	Formula	Molecular Weight	RT [min]	Kegg_ID	HMDB_ID	m/z
Com_47502_neg	3-benzyl-1-butyl-4-hydroxy-1,2-dihydroquinolin-2-one	C20 H21 N O2	307.15398	6.74	--	--	306.14651
Com_47836_neg	3-(4,5-diphenyl-1,3-oxazol-2-yl)propanoic acid	C18 H15 N O3	293.10592	9.69	--	--	292.09872
Com_49139_neg	5-(1,3-dioxo-1,3-dihydro-2H-isoindol-2-yl)-1H-indole-3-carbonitrile	C17 H9 N3 O2	241.0591	1.213	--	--	240.0519
Com_49176_neg	2,5-dimethyl-N-(2-oxo-3-azepanyl)-3-thiophenesulfonamide	C12 H18 N2 O3 S2	302.07957	6.047	--	--	301.07233
Com_49678_neg	4-(octyloxy)benzoic acid	C15 H22 O3	250.15742	9.396	--	--	249.15015
Com_50029_neg	PE (18:2e/14:0)	C37 H72 N O7 P	673.50709	16.569	--	--	672.49982
Com_50105_neg	MGDG (16:2/18:3)	C43 H72 O10	808.53712	16.763	--	--	807.52985
Com_50189_neg	Perfluorooctanoic acid (PFOA)	C8 H F15 O2	413.96904	7.144	--	--	412.96182
Com_50388_neg	GlcADG (14:0-15:0)	C38 H70 O11	702.4944	14.246	--	--	701.48712
Com_50630_neg	2-(4-chlorophenoxy)-N-(2-oxo-3-azepanyl)nicotinamide	C18 H18 Cl N3 O3	359.10139	7.898	--	--	358.09396
Com_51294_neg	1-(4-fluorophenyl)-2-(4-methoxyphenyl)-4-(2-naphthyl)butane-1,4-dione	C27 H21 F O3	412.14177	8.93	--	--	411.13449
Com_51642_neg	9-[(2-hydroxyethoxy)methyl]-1,9-dihydro-6H-purin-6-one	C8 H10 N4 O3	210.07571	5.639	--	--	209.06844
Com_51686_neg	2-{4-[(2-aminophenyl)thio]-3-nitrobenzoyl}benzoic acid	C20 H14 N2 O5 S	394.05473	8.165	--	--	393.04745
Com_51917_neg	ethyl 3-[(4-chlorophenethyl)amino]-2-cyanoacrylate	C14 H15 Cl N2 O2	278.08296	7.664	--	--	277.07568
Com_54726_neg	2-({[4-(6-methyl-1,3-benzothiazol-2-yl)phenyl]imino}methyl)phenol	C21 H16 N2 O S	344.10271	8.665	--	--	343.09543

Continuation of Supplementary Table 2

Compound_ID	Name	Formula	Molecular Weight	RT [min]	Kegg_ID	HMDB_ID	m/z
Com_54876_neg	4-(4-nitrophenylazo)aniline	C12 H10 N4 O2	242.08108	7.638	--	--	241.07381
Com_55069_neg	1,3-diphenyl-1H-pyrazole-4-carbaldehyde oxime	C16 H13 N3 O	263.10214	7.356	--	--	262.09497
Com_55482_neg	β-Estradiol 3-(β-D-glucuronide) 17-sulfate	C24 H32 O11 S	528.17525	6.911	--	--	527.16797
Com_55814_neg	Glycohyocholic acid Sodium salt	C26 H42 N Na O6	487.29204	9.494	--	--	486.28476
Com_386_neg	10-Undecenoic acid	C11 H20 O2	184.14625	7.178	--	--	183.13956
Com_1147_neg	gamma-Nonanolactone	C9 H16 O2	156.11536	7.109	--	--	155.10818
Com_32561_neg	4-morpholinobenzoic acid	C11 H13 N O3	207.0899	8.102	--	--	206.08263
Com_21689_neg	5α-Dihydrotestosterone glucuronide	C25 H38 O8	466.25765	11.548	--	--	465.25046
Com_12976_neg	12-Oxo phytodienoic acid	C18 H28 O3	292.20429	12.412	--	--	291.19693
Com_524_neg	All trans-Retinal	C20 H28 O	284.21461	14.889	--	--	283.20724
Com_2522_neg	LPE 14:0	C19 H40 N O7 P	425.25524	14.2	--	--	424.24786
Com_2681_neg	2-Methoxyestradiol (2-MeOE2)	C19 H26 O3	302.18884	10.976	--	--	347.18704
Com_15555_neg	1-(1,8-dihydroxy-3,6-dimethyl-2-naphthyl)ethan-1-one	C14 H14 O3	230.09479	8.598	--	--	229.08757
Com_1755_neg	3-methyl-5-oxo-5-(4-toluidino)pentanoic acid	C13 H17 N O3	235.1213	9.471	--	--	234.11397
Com_2270_neg	Nor-9-carboxy-δ9-THC	C21 H28 O4	344.19921	12.862	--	--	343.19202
Com_4830_neg	1,2,3-Benzenetricarboxylic acid	C9 H6 O6	210.01688	1.09	--	--	209.00958
Com_9223_neg	N-Acetylsphingosine	C20 H39 N O3	341.29356	14.64	--	--	340.28641
Com_4659_neg	Prostaglandin A3	C20 H28 O4	664.39885	13.458	--	--	663.39191
Com_837_neg	MGMG (18:2)	C27 H48 O9	516.33079	11.488	--	--	515.3233
Com_6254_neg	(R)-3-Hydroxy myristic acid	C14 H28 O3	244.20419	12.99	--	--	243.19717
Com_11158_neg	2,3-Dinor-8-epi-prostaglandin F2α	C18 H30 O5	326.20985	10.417	--	--	325.20258
Com_8245_neg	Feruloyl Putrescine	C14 H20 N2 O3	264.14781	7.988	--	--	263.14047

Continuation of Supplementary Table 2

Compound_ID	Name	Formula	Molecular Weight	RT [min]	Kegg_ID	HMDB_ID	m/z
Com_2030_neg	3,8,9-trihydroxy-10-propyl-3,4,5,8,9,10-hexahydro-2H-oxecin-2-one	C12 H20 O5	244.13148	2.107	--	--	243.12419
Com_982_neg	Chenodeoxycholic acid-3-beta-D-glucuronide	C30 H48 O10	568.32649	11.483	--	--	567.31921
Com_4210_neg	23-Nordeoxycholic acid	C23 H38 O4	378.27799	11.467	--	--	377.27051
Com_7898_neg	12-epi Leukotriene B4	C20 H32 O4	318.22003	12.156	--	--	317.21283
Com_20148_neg	(+/-)11(12)-DiHET	C20 H34 O4	338.24645	10.55	--	--	337.23907
Com_8209_neg	(3R)-8-hydroxy-3-(4-methoxyphenyl)-3,4-dihydro-1H-2-benzopyran-1-one	C16 H14 O4	270.08969	13.101	--	--	269.08237
Com_8621_neg	LPE 13:0	C18 H38 N O7 P	411.23942	13.856	--	--	410.23196
Com_10407_neg	Cannabigerolic acid	C22 H32 O4	360.23056	12.877	--	--	359.22318
Com_13189_neg	Orsellinic acid ethyl ester	C10 H12 O4	196.07392	4.768	--	--	195.0667
Com_3405_neg	Dimetghyl 4-Hydroxyisophthalate	C10 H10 O5	210.05332	10.299	--	--	209.04607
Com_5629_neg	6-Methoxy-2-naphthoic acid	C12 H10 O3	202.06337	11.57	--	--	201.05612
Com_2570_neg	all-cis-4,7,10,13,16-Docosapentaenoic acid	C22 H34 O2	330.25642	14.533	--	--	329.24915
Com_17187_neg	4-[2-(2-oxo-1-imidazolidinyl)ethyl]-1lambda~6~,4-thiazinane-1,1-dione	C9 H17 N3 O3 S	248.1012	1.203	--	--	247.09445
Com_1587_neg	Lysopa 16:0	C19 H39 O7 P	410.24444	13.705	--	--	455.24274
Com_10675_neg	N1-isopropyl-2-(1H-2-pyrrolylcarbonyl)-1-hydrazinecarboxamide	C9 H14 N4 O2	420.21923	11.475	--	--	419.21216
Com_3876_neg	Oleoyl-L-alpha-lysophosphatidic acid	C21 H41 O7 P	436.26035	13.979	--	--	481.25861
Com_12921_neg	2,3-Dinor-11β-prostaglandin F2α	C18 H30 O5	326.20986	13.133	--	--	325.20261
Com_46978_neg	3-[3-(beta-D-Glucopyranosyloxy)-2-methoxyphenyl]propanoic acid	C16 H22 O9	358.12742	7.627	--	--	357.12015

Continuation of Supplementary Table 2

Compound_ID	Name	Formula	Molecular Weight	RT [min]	Kegg_ID	HMDB_ID	m/z
Com_21051_neg	LysoPC 18:0	C26 H54 N O7 P	523.36627	13.876	--	--	522.35815
Com_2682_neg	Dl-3-Hydroxynorvaline	C5 H11 N O3	133.07424	1.386	--	--	132.06699
Com_15171_neg	N-(1,3-benzodioxol-5-yl)-7-chloroquinolin-4-amine	C16 H11 Cl N2 O2	298.04831	9.302	--	--	297.04108
Com_5729_neg	(2S)-4-Oxo-2-phenyl-3,4-dihydro-2H-chromen-7-yl beta-D-glucopyranoside	C21 H22 O8	224.07078	8.494	--	--	447.13428
Com_9331_neg	5-(tert-butyl)-2-methyl-N-(5-methyl-3-isoxazolyl)-3-furamide	C14 H18 N2 O3	262.13231	8.443	--	--	261.12524
Com_16407_neg	5-Methyl-dl-tryptophan	C12 H14 N2 O2	218.10602	6.599	--	--	217.09868
Com_15821_neg	DGMG (18:2)	C33 H58 O14	678.38409	14.287	--	--	677.37677
Com_22283_neg	Lysops 22:5	C28 H46 N O9 P	571.29094	11.999	--	--	570.28265
Com_170_neg	7-Hydroxy-3,4-dihydrocarbostyril	C9 H9 N O2	163.06346	9.015	--	--	162.05606
Com_6926_neg	Pyrithioxin	C16 H20 N2 O4 S2	368.0875	9.789	--	--	367.08008
Com_199_neg	1-(2,4-dihydroxyphenyl)-2-(3,5-dimethoxyphenyl)propan-1-one	C17 H18 O5	302.11574	12.859	--	--	301.10825
Com_22714_neg	Epoxomicin	C28 H50 N4 O7	554.36739	14.234	--	--	553.3609
Com_21249_neg	3,5-dimethyl-N'-[4-(trifluoromethyl)benzoyl]isoxazole-4-carbohydrazide	C14 H12 F3 N3 O3	327.08183	7.382	--	--	326.07446
Com_329_neg	Tauroursodeoxycholic acid Dihydrate	C26 H49 N O8 S	535.31935	12.823	--	--	534.31207
Com_23230_neg	2-(Dimethylamino)Guanosine	C12 H17 N5 O5	311.12373	6.219	--	--	310.11661
Com_36526_neg	N1-(6-methyl-4-oxo-3,4-dihydroquinazolin-2-yl)-4-nitrobenzamide	C16 H12 N4 O4	324.08293	7.858	--	--	323.07565

Continuation of Supplementary Table 2

Compound_ID	Name	Formula	Molecular Weight	RT [min]	Kegg_ID	HMDB_ID	m/z
Com_40902_neg	[1,1'-biphenyl]-2,2'-dicarboxylic acid	C14 H10 O4	242.05854	7.674	--	--	241.05119
Com_130_neg	15-Deoxy-Δ12,14-prostaglandin D2	C20 H30 O4	316.20423	14.154	--	--	315.19693
Com_20283_neg	2-cyano-3-(3,4-dimethoxyphenyl)acrylic acid	C12 H11 N O4	233.06928	8.317	--	--	232.06203
Com_14036_neg	4-oxo-4,5,6,7-tetrahydrobenzo[b]furan-3-carboxylic acid	C9 H8 O4	180.04267	6.598	--	--	179.03545
Com_3311_neg	4-chloro-2-(1H-pyrazol-3-yl)phenol	C9 H7 Cl N2 O	194.02533	2.022	--	--	193.01814
Com_28671_neg	1-(2,4-diphenyl-2,3-dihydro-1H-1,5-benzodiazepin-1-yl)propan-1-one	C24 H22 N2 O	354.1716	10.295	--	--	353.1644
Com_20263_neg	P-Coumaroyl Agmatine	C14 H20 N4 O2	276.15762	7.23	--	--	257.1398
Com_26746_neg	Hydroxyglutaric acid	C5 H8 O5	148.03753	1.128	--	--	295.06778
Com_7699_neg	(±)-Abscisic acid	C15 H20 O4	264.13657	9.693	--	--	263.1293
Com_23248_neg	2-Methylbutyl beta-D-glucopyranoside	C11 H22 O6	296.1436	6.876	--	--	295.13626
Com_19286_neg	Dibenzoyl Thiamine	C26 H26 N4 O4 S	490.16981	11.409	--	--	489.16254
Com_10421_neg	Lysopc 16:1	C24 H48 N O7 P	493.31819	14.746	--	--	492.31091
Com_24531_neg	N-[4-(diethylamino)phenyl]-N'-phenylurea	C17 H21 N3 O	284.17161	2.258	--	--	283.16406
Com_28497_neg	3-amino-2,6-diphenyl-4,7-dihydro-2H-pyrazolo[3,4-d]pyrimidin-4-one	C17 H13 N5 O	303.11148	7.44	--	--	302.10391

Supplementary Table 3 Differential analysis of metabolites contained in fatty acids and conjugates

Name	Formula	FC	log2FC	Pvalue	ROC	VIP
Elaidic acid	C18 H34 O2	0.1554118	-2.6858317	1.24E-11	0.975	1.7482436
Suberic acid	C8 H14 O4	3.9597254	1.9854004	2.02E-10	0.975	1.6887793
Sebacic acid	C10 H18 O4	2.8236033	1.4975374	9.41E-10	0.95	1.6582429
20-Carboxy-Leukotriene B4	C20 H30 O6	9.8367116	3.2981761	1.91E-09	0.94	1.6559659
Erucic acid	C22 H42 O2	0.2568261	-1.9611363	1.31E-06	0.9	1.4584031
Undecanoic acid	C11 H22 O2	2.6657504	1.4145417	5.99E-07	0.9125	1.4551805
Eicosapentaenoic acid	C20 H30 O2	17.720387	4.1473382	7.58E-06	0.8925	1.4411521
Undecanedioic acid	C11 H20 O4	2.2712257	1.1834711	2.42E-06	0.865	1.4361134
Docosanoic acid	C22 H44 O2	0.4056603	-1.301656	1.62E-06	0.9175	1.4326934
Capric acid	C10 H20 O2	4.2874205	2.1001099	7.43E-06	0.9325	1.3912172
2-Isopropylmalic acid	C7 H12 O5	4.5065926	2.172037	8.63E-06	0.895	1.3801447
Adipic acid	C6 H10 O4	2.2430606	1.1654686	9.39E-06	0.875	1.324406
2-Isopropylmalate	C7 H12 O5	1.871455	0.9041603	1.74E-05	0.87	1.3010894
Octanedioic acid	C8 H14 O4	5.5210015	2.46493	3.37E-05	0.8575	1.2947152
13Z,16Z-Docosadienoic Acid	C22 H40 O2	0.2065133	-2.2756936	2.79E-05	0.8475	1.2922629
Azelaic acid	C9 H16 O4	6.437702	2.6865458	2.53E-05	0.86	1.2807785
9,10-Dihome	C18 H34 O4	0.5070179	-0.9798913	2.62E-05	0.86	1.2716502
3-Methyladipic acid	C7 H12 O4	4.1359548	2.0482204	4.61E-05	0.825	1.2606964
trans-2-Butene-1,4-dicarboxylic Acid	C6 H8 O4	2.5693821	1.3614215	0.0001898	0.8175	1.1670818
Traumatic acid	C12 H20 O4	0.3836787	-1.3820292	0.0002163	0.815	1.1485497
Tridecylic acid	C13 H26 O2	1.968984	0.9774514	0.0002903	0.8075	1.1303801
Desthiobiotin	C10 H18 N2 O3	2.3342557	1.2229626	0.0004999	0.7825	1.1255675
Lauric acid	C12 H24 O2	1.9686341	0.977195	0.0006331	0.8275	1.0774495
Oleic acid	C18 H34 O2	0.6210359	-0.6872514	0.0010392	0.78	1.0507758
Lignoceric Acid	C24 H48 O2	0.6099884	-0.7131462	0.0011819	0.8325	1.0371879
Mevalonic acid	C6 H12 O4	1.6153811	0.6918746	0.0013996	0.7875	1.0199413
Myristic acid	C14 H28 O2	2.7399676	1.4541588	0.0023113	0.795	1.0049261
Palmitoleic Acid	C16 H30 O2	0.547878	-0.8680735	0.0027706	0.795	1.0004288
2-Hydroxy-2-methylbutanoic acid	C5 H10 O3	1.4579241	0.5439156	0.0029336	0.775	0.9904883
2-Hydroxyisocaproic Acid	C6 H12 O3	1.6375927	0.7115766	0.0040242	0.725	0.9514926

Continuation of Supplementary Table 3

Name	Formula	FC	log2FC	Pvalue	ROC	VIP
Decanoic acid	C10 H20 O2	1.666621	0.736926	0.0045737	0.735	0.9466013
3,3-Dimethylglutaric acid	C7 H12 O4	3.9776166	1.9919042	0.0098843	0.6875	0.9442639
Docosatrienoic acid	C22 H38 O2	0.3986337	-1.3268642	0.004593	0.74	0.9388461
Adrenic acid	C22 H36 O2	0.5975267	-0.742925	0.008825	0.705	0.9028279
Stearic acid	C18 H36 O2	0.3792291	-1.3988582	0.0075025	0.74	0.8885386
Dodecanedioic acid	C12 H22 O4	0.4853727	-1.0428352	0.0482346	0.665	0.770615
Pentadecanoic acid	C15 H30 O2	1.6152748	0.6917796	0.0398527	0.68	0.7500296
Docosapentaenoic acid	C22 H34 O2	1.961585	0.9720198	0.0952022	0.655	0.7098109
Caprylic acid	C8 H16 O2	1.1653257	0.2207332	0.033514	0.72	0.7044789
Nervonic acid	C24 H46 O2	0.4409288	-1.1813824	0.0364432	0.675	0.6977531
20-Hydroxy-(5Z,8Z,11Z,14Z)-eicosatetraenoic acid	C20 H32 O3	0.7469796	-0.4208592	0.0574651	0.71	0.6406
3-Hydroxy-3-methylglutaric acid	C6 H10 O5	1.3675813	0.4516266	0.0682171	0.645	0.6270537
Hexanoic acid	C6 H12 O2	1.6535695	0.7255837	0.0725288	0.6625	0.6106732
Protectin D1	C22 H32 O4	0.6397973	-0.6443133	0.0955188	0.76	0.5986348
2-Hydroxycaproic acid	C6 H12 O3	0.4962574	-1.0108394	0.083289	0.6225	0.5811459
Mupirocin	C26 H44 O9	0.9131447	-0.1310847	0.1163261	0.8075	0.531422
Nonadecanoic acid	C19 H38 O2	0.5149824	-0.9574048	0.132016	0.625	0.5270343
cis-gondoic acid	C20 H38 O2	0.6965985	-0.5216007	0.119537	0.6625	0.5196833
3-Hydroxyvaleric acid	C5 H10 O3	0.7974182	-0.3265916	0.1347753	0.635	0.5109602
2-Methylpentanedioic acid	C6 H10 O4	1.7650324	0.8196947	0.1374325	0.6925	0.5041302
5-Phenylvaleric Acid	C11 H14 O2	0.7013304	-0.5118339	0.172875	0.5525	0.5014709
Tetradecanedioic acid	C14 H26 O4	0.5139662	-0.9602547	0.1775171	0.635	0.4961124

Continuation of Supplementary Table 3

Name	Formula	FC	log2FC	Pvalue	ROC	VIP
16-Hydroxyhexadecanoic acid	C16 H32 O3	1.8580403	0.8937818	0.3595464	0.5475	0.4742817
Arachidic acid	C20 H40 O2	1.300567	0.3791408	0.2193743	0.6275	0.466775
Valeric acid	C5 H10 O2	0.8916826	-0.1653979	0.1988501	0.645	0.4317652
Palmitic acid	C16 H32 O2	1.2186576	0.2852928	0.8629621	0.5125	0.4129001
Docosahexaenoic acid	C22 H32 O2	1.3463856	0.4290917	0.7255104	0.5425	0.3973186
3-hydroxy-3-methylpentanedioic acid	C6 H10 O5	0.501834	-0.9947179	0.3946779	0.6575	0.3876731
5-OxoETE	C20 H30 O3	1.4004409	0.4858811	0.3386234	0.625	0.3864134
4-Methylvaleric Acid	C6 H12 O2	1.2104926	0.2755942	0.2691753	0.5975	0.3747473
Citraconic acid	C5 H6 O4	0.7289405	-0.456127	0.2805973	0.5825	0.3633961
2-Hydroxymyristic acid	C14 H28 O3	0.720246	-0.4734383	0.7921103	0.5075	0.3482532
8Z,11Z,14Z-Eicosatrienoic acid	C20 H34 O2	1.2117973	0.2771484	0.9356734	0.52	0.3390366
2-Hydroxyvaleric acid	C5 H10 O3	0.8792413	-0.1856689	0.4230465	0.4625	0.3186618
Arachidonic acid	C20 H32 O2	1.0389251	0.0550916	0.4069676	0.6175	0.296398
Tiglic acid	C5 H8 O2	0.8716968	-0.1981016	0.4816791	0.5725	0.2574251
Nonanoic acid	C9 H18 O2	0.8711735	-0.198968	0.995144	0.555	0.1992774
Heptanoic acid	C7 H14 O2	0.8730235	-0.1959077	0.7467589	0.485	0.1787354
cis-5,8,11,14,17-Eicosapentaenoic acid	C20 H30 O2	0.6898984	-0.5355441	0.8968509	0.5225	0.1034792
Methylsuccinic acid	C5 H8 O4	1.0468981	0.0661211	0.883582	0.53	0.049603
cis-2-Decenoic acid	C10 H18 O2	0.367685	-1.4434577	0.9986609	0.55	0.01656

Supplementary Table 4. Primers for macaque genes

Primer			Sequence (5′-3′)	Annealing temperature	Length (bp)
Individual identification	D12S372	external	F-GGAAGCAGTCACAACCAGAAAT<br>F-TGGTGCTCTATAAGATGAGGTGTC	58℃	469
		internal	F-AGTCACAACCAGAAATGAACCAC<br>R-TGGTGCTCTATAAGATGAGGTGTC	58℃	463
	D16S403	external	F-CCAAAGCTGGAACTGGTGTCA<br>R-TGAATCACAGAGCACGGCAAG	58℃	429
		internal	F-CCAAAGCTGGAACTGGTGTCA<br>R-AGTGAGGTGGGAATCAAAGGTC	58℃	372
	D5S2497	external	F-GGGTCAATTGGGGATGAGATGT<br>R-CGTGATCCCACCACTGGACTCTA	58℃	413
		internal	F-GGGTCAATTGGGGATGAGATGT<br>R-CTGTAGCCTGGGTGAGAGAGTAAGA	58℃	395
	D3S3697	external	F-TAAGACCTAAGACTGGTGTTCGTG<br>R-CTGAGGCAGGCAGTTTATTTG	58℃	873
		internal	F-CAAGAAAAACCAGCTTCAATGC<br>R-GGGTGTCCACACTAAGTTCAGC	58℃	363
	D1S2878	external	F-TGGGTGGGCATTAGCCATTATC<br>R-GCTCGGAAGTCAAAGAGTTCTAGG	60℃	665
		internal	F-AGCAAACTCTCTACCCTTCCTG<br>R-AGCAATGTCCCTGATGTTTACC	62℃	349
	D9S252	external	F-GCAAAAGGTGCTAATCTCTACG<br>R-GGGTGTGGGTATGTGTGTAGAT	58℃	595
		internal	F-GCAAAAGGTGCTAATCTCTACG<br>R-GACCATGATTTGTCAACTCCTAA	58℃	384
Sex determination	ZFX		F-CTGACCAGCAAAGCAGAGAAGG<br>R-CAAGGGTTACTGAATCGCCAC	59℃	130
	SRY		F-GAACGCATTCATTGTGTGGTCTC<br>R-CTGTGTTGATGGGCGGTAAGTG	60℃	364

Supplementary Table 5 Sample information sheet

High altitude (H)				Low altitude (L)			
Pamulin (HA)		Xi'eluo (HB)		Baidicheng (LA)		Simianshan (LB)	
Female	Male	Female	Male	Female	Male	Female	Male
6	4	5	5	5	5	5	5
20				20			
